# DNA-based floristic survey of red algae (Rhodophyta) growing in the mesophotic coral ecosystems (MCEs) offshore of Tanegashima Island, northern Ryukyu Archipelago, Japan

**DOI:** 10.1371/journal.pone.0316067

**Published:** 2025-03-10

**Authors:** Masahiro Suzuki, Ryuta Terada

**Affiliations:** 1 United Graduate School of Agricultural Sciences, Kagoshima University, Korimoto, Kagoshima, Japan; 2 Kobe University Research Center for Inland Seas, Iwaya, Awaji, Japan; King Abdulaziz University, SAUDI ARABIA

## Abstract

A molecular-based floristic survey of marine red algal biodiversity was conducted offshore Tanegashima Island, which is located at the northern end of mesophotic coral ecosystems (MCEs), in the Ryukyu Archipelago, Japan. This study provides the first comprehensive catalog of red algae comprising the sublittoral marine flora of offshore Tanegashima Island, Japan, and represents the first exhaustive molecular-assisted survey of red algal marine flora in Japan. Morphological and molecular analyses using plastid-encoded *rbc*L and mitochondrion-encoded *cox*1 genes revealed a total of 129 species, which included nine newly recognized species in Japan. Morphologically, 82 species were assigned to known species. Among the 82 species, 17 included cryptic species, and 25 appeared to have misapplied names. The remaining 47 species could not be identified to the species level, which indicates the necessity of a detailed reference library containing validated DNA barcodes and further taxonomic studies based on morpho-molecular analyses.

## Introduction

In tropical and subtropical regions, the highly productive ecosystems include mesophotic coral ecosystems (MCEs), which are characterized by light-dependent corals, sponges, algae, and other associated communities that are typically found at depths ranging from 30–40 m to over 150 m [1]. Concerns have recently been documented and changes to such ecosystems have been projected as a result of climate change and its associated impacts on productivity. The stability, resistance to disturbance, and resilience of such ecosystems depend on the diversity and relationships among their components, including seaweeds. Marine macroalgae are essential components of such ecosystems and play a significant role in their productivity and restoration following disturbance [2]. A thorough understanding of marine macroalgae diversity and biogeographic composition of flora within such communities is essential for predicting further MCE changes and safeguarding against such changes. Unfortunately, such knowledge is lacking, and genetic verification of species composition of floras is required in many areas of the world.

One of these underexplored areas is the vicinity of Tanegashima Island. This island is located at the northern end of the Ryukyu Archipelago in the northwestern Pacific Ocean and the northern end of the MCEs in Japan. Tanegashima Island and its vicinity, including Mageshima Island and Yakushima Island, belong to a subtropical region and are known as the northern limit of true coral reef distribution [3]. High-latitude coral reefs are localized and scattered around the Tanegashima, Mageshima, and Yakushima Islands [4]. Additionally, the seafloor around the western area of Tanegashima Island forms a flat submarine topography at depths of approximately 30–60 m.

The macroalgal flora of this water has been studied since the early 20th century. As a pioneer in this area, Dr. Takeshi Tanaka (1907–1997) conducted continuous research voyages offshore Tanegashima Island (formerly called offshore Mageshima Island) from the 1950s to the 1960s, and 12 sublittoral algae, including seven new species, have been reported from Tanaka’s collection [5–9]. Additionally, four sublittoral algae, including a new species, were reported from 1969 to 1977 [10–15]. Since 2007, three species new for Japan were found here [16,17], and a new species was described [18] based on recent collection from offshore Tanegashima Island (as offshore Mageshima Island). To date, the total list of marine macroalgae from Tanegashima Island and its vicinity includes 271 species, among which 225 are red algae [5,19]. Most species were collected from intertidal to shallow subtidal zones (up to 20 m in depth). Sublittoral algae collected offshore Tanegashima Island have been recorded in scattered publications [5–18]; however, a comprehensive catalog of sublittoral marine flora from this island has never been reported.

Until now, data on the composition of local floras have mostly been based on species that were morphologically identified. However, analyzing morphology alone has been shown to result in numerous examples of misidentification or underestimation of species diversity because morphological identification often cannot overcome issues related to cryptic species diversity or convergent evolution. These issues can be resolved using sequence data.

Sequence data are critical for determining the diversity of local macroalgal floras and distribution of seaweeds. DNA barcoding is a useful tool for inferring algal diversity and distribution. The genetic marker sequence of a specimen can be compared with a database of sequences specific to a particular species to provide new verified data on species diversity. The plastid-encoded ribulose bisphosphate carboxylase gene (*rbc*L) and mitochondria-encoded cytochrome c oxidase I gene (*cox*1) have been used as standard markers for the DNA barcoding of red algae [20–24].

This study provides an inventory of red algae offshore Tanegashima Island based on a combination of morphological and DNA-based floristic surveys using *rbc*L and *cox*1 sequences. This study also discusses the characteristics of sublittoral flora offshore Tanegashima Island.

## Materials and methods

### Study area

**Fig 1:**
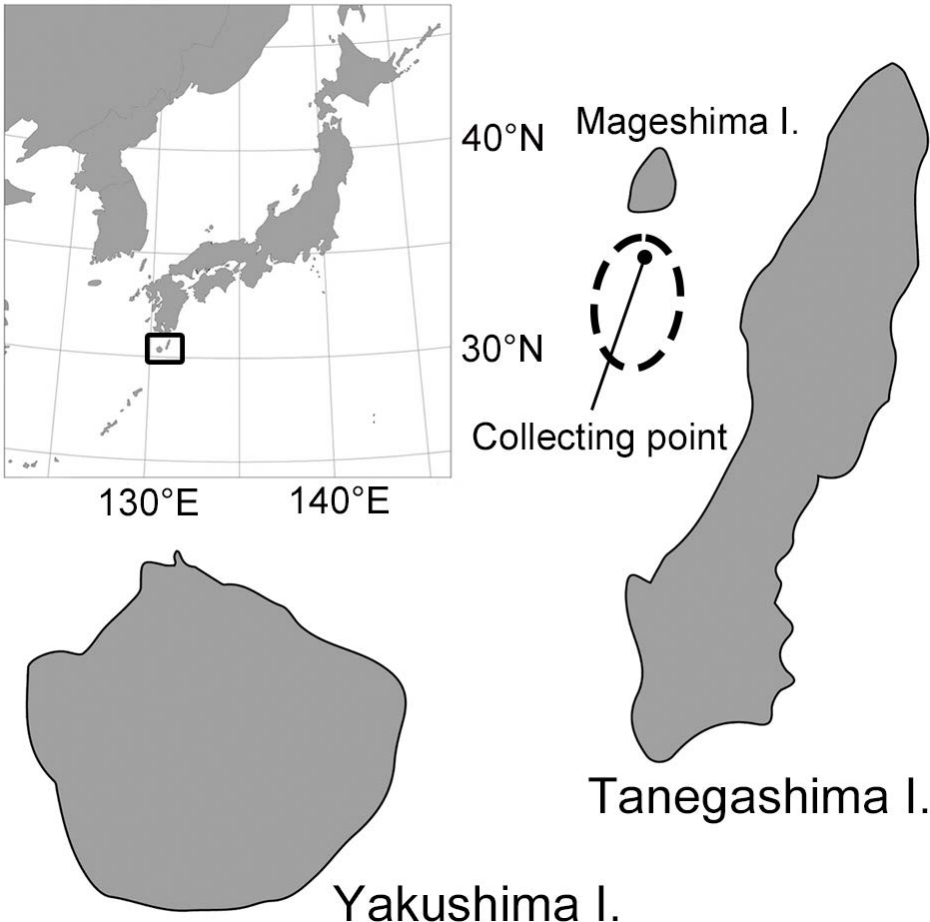
Map showing the sampling collection point offshore Tanegashima Island. The dashed line circle indicates the area of the MCEs.

The study area was outside of the coral reef and located approximately 10 km from the western coast of Tanegashima Island and 5 km from the southern coast of Mageshima Island (Fig 1). The seafloor at the study area was flat and composed of cobbles with corals, sponges, and nongeniculate coralline algae. The seawater temperature around the study area at a depth of approximately 35 m ranged from 19°C to 25°C and irradiance was around 200 μmol photons m^ − 2^ s^ − 1^ (March to October) [25].

### Sampling

Samples were collected from the seafloor at a depth of approximately 35 m off the coast of Tanegashima Island, Japan using a dredge on the T/S *Nansei-Maru* (Faculty of Fisheries, Kagoshima University) between May 26, 2015, and September 29, 2022. Since the collection site is not a protected area, such as a natural reserve or a fisherman’s cooperative reserve, we confirmed with the Fisheries Promotion Division of the Kagoshima Prefectural Government that no specific permission was required. The specimens were either quickly frozen on the vessel or transported as fresh material in plastic bottles (20L) filled with seawater at 24°C for DNA extraction and morpho-anatomical observations. Pieces of fresh and defrosted specimens were dried in silica gel for DNA extraction. After DNA extraction and morpho-anatomical observations, the defrosted specimens were pressed into herbarium specimens. Voucher specimens were deposited at the National Museum of Nature and Science, Tokyo, Japan (TNS). The nongeniculate coralline algae and some small epiphytic red algae were excluded from this study because we could not extract DNA and failed PCR.

### Morphological observations and identifications

For morpho-anatomical observations, specimens were first thawed. The habits of the specimens were observed, and photographs were taken using a Tough TG-6 digital camera (Olympus, Tokyo, Japan). The specimens except for filamentous species were sectioned by hand or using a freezing microtome (MA-101, Komatsu Electronics, Komatsu, Japan) for the anatomical observations. Several sections were stained with Lactophenol blue solution (Sigma-Aldrich®, Tokyo, Japan), acidified with 10% HCl, and mounted in 50% aqueous Karo syrup. Photomicrographs were taken using a BX50 microscope (Olympus, Tokyo, Japan) with a WRAYCAM-NOA630B digital camera (WRAYMER, Osaka, Japan). Drawings were made using the U-DA Drawing Attachment (Olympus, Tokyo, Japan) with a BX50 microscope.

Morphology-based identifications were largely based on comparisons with previously published literature on all species belonging to Nemaliales and Rhodymeniophycidae recorded in Japan, South Korea, Hawaii, the USA, and Australia [26–43], original descriptions of each species, and several taxonomic studies conducted on related genera collected from offshore Tanegashima Island.

### DNA extraction, PCR amplification, and sequencing

Partial *rbc*L and *cox*1 were sequenced for molecular phylogenetic analyses. The specimens used in the molecular analyses are listed in S1–S13 Tables in S1 File. Genomic DNA was extracted using GenCheck® DNA Extraction Reagent (FASMAC Co., Ltd., Atsugi, Japan). Total DNA was used as a template for the polymerase chain reaction (PCR) amplification of the *rbc*L and *cox*1 sequences using a KOD FX Neo (TOYOBO CO. LTD., Osaka, Japan) and TaKaRa PCR Thermal Cycler Dice Gradient (TaKaRa Bio, Kusatsu, Japan). The primers used for PCR amplification were as follows: *rbc*L: F8 – R1150 and Rh3 – R1381 [44,45]; *cox*1: GazF1 – GazR1 [46] or GazF1 – C880R [47]. The temperature cycling protocol for both *rbc*L and *cox*1 sequences was as follows: 2 min at 94°C for an initial denaturation step, followed by 35 cycles of 15 s of denaturation at 94°C, 30 s of primer annealing at 46°C, 1 min extension at 68°C, a final 7 min extension at 72°C, and then a hold at 4°C. The amplified DNA fragments were purified using an IlluminaTM ExoProStar (Cytiva, Tokyo, Japan). The PCR products were sequenced by a DNA sequencing service (FASMAC, Atsugi, Japan). Reverse and direct chromatograms were assembled using the GeneStudioTM Professional Ver. 2.2. (GeneStudio, Inc.). The *rbc*L and *cox*1 sequences of 159 and 138 specimens collected from offshore Tanegashima Island were sequenced (S1–S13 Tables in S1 File). For comparison, the *rbc*L and *cox*1 sequences of 104 and 107 specimens collected from various parts of Japan and Taiwan were also sequenced (S1–S13 Tables in S1 File). The determined sequences were deposited in the DNA Data Bank of Japan (DDBJ) under accession numbers LC820897 to LC821382.

### Genetic identification

The newly determined sequences were identified through the Basic Local Alignment Search Tool (BLAST) of National Center for Biotechnology Information (NCBI). Based on the results of BLAST, we compiled sequence data available from INSD; International Nucleotide Sequence Database (DDBJ/EMBL/GenBank) and BOLD for each family, subfamily, or genus (S1–S13 Tables in S1 File). In total, 77 datasets were subjected to maximum likelihood (ML) and Bayesian inference (BI) phylogenetic analyses. Descriptions of the ML and BI analyses are presented in S2 File and S1–S34 Tables in S3 File.

The specimens were genetically identified based on the results of BLAST, phylogenetic analyses, and divergence of *rbc*L and *cox*1. Typically, approximately 1% (to 2%) and 1% to 2% (to 3%) divergence for *rbc*L [48] and *cox*1 [46], respectively, were used as thresholds to define taxa.

### Accurate application of species name to specimens

The specimens were finally identified using combined morphological and genetic methods. The results were separated based on the following identification patterns (Id). C1: completely identified. The identification of species was supported by both molecular and morphological data. C2: completely identified. The identification of species was supported only by morphological data. C3: completely identified. The identification of species was supported only by molecular data. T: tentatively identified. The species was morphologically identified; however, molecular data revealed that it includes several cryptic species. U1: unidentified. The DNA sequences did not closely match the INSD data or matched sequences that were unidentified at the species or genus level. In addition, the specimens did not morphologically match any species recorded in Japan or its vicinity. U2: unidentified. The specimen was morphologically similar to a known species; however, molecular data indicated that it was distinct from a known species. U3: unidentified. The DNA sequences did not closely match the INSD data and did not exhibit distinguishable or reliable morphological characteristics. The numbers of species associated with each identification pattern are presented in Table 1.

**Table 1:** The species number for identification pattern. C1: completely identified. The identification of species was supported by both molecular and morphological data. C2: completely identified. The identification of species was supported only by morphological data. C3: completely identified. The identification of species was supported only by molecular data. T: tentatively identified. The species was morphologically identified; however, molecular data revealed that it includes several cryptic species. U1: unidentified. The DNA sequences did not closely match the INSD data or matched sequences that were unidentified at the species or genus level. In addition, the specimens did not morphologically match any species recorded in Japan or its vicinity. U2: unidentified. The specimen was morphologically similar to a known species; however, molecular data indicated that it was distinct from a known species. U3: unidentified. The DNA sequences did not closely match the INSD data and did not exhibit distinguishable or reliable morphological characteristics.

Identification pattern	Number of species
C1	22
C2	18
C3	3
T	17
U1	27
U2	25
U3	17

## Results

### Identification

**Fig 2:**
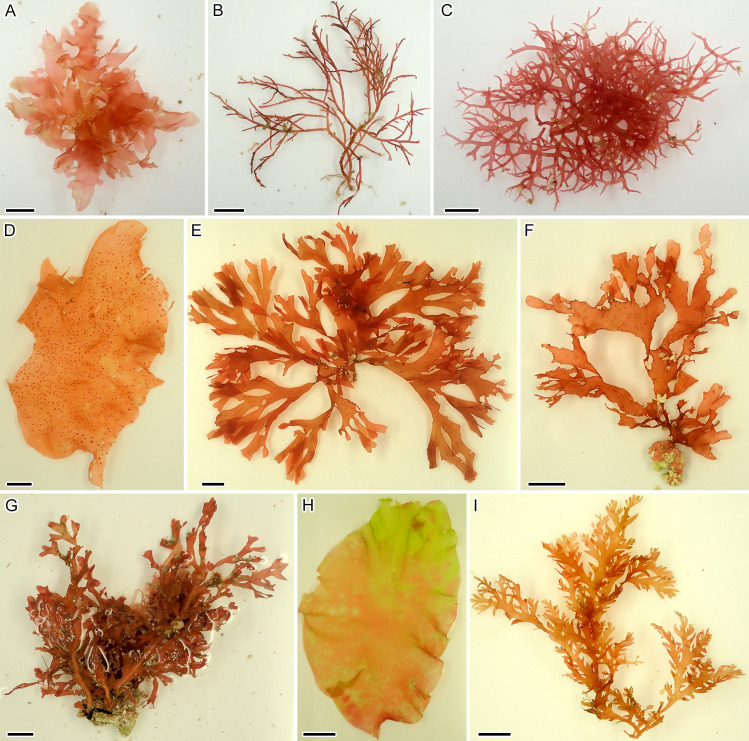
Newly recorded species in Japan collected offshore Tanegashima Island. (A) *Pseudopolyneura hyacinthina* (TNS AL-222210). (B) *Acanthophora dendroides* (TNS AL-222201). (C) *Calliblepharis yasutakei* (TNS AL-220757). (D) *Croisettea kalaukapuae* (TNS AL-220766). (E) *Stenogramma guleopoense* (TNS AL-209832). (F) *Stenogramma lamyi* (TNS AL-209842). (G) *Yonagunia taiwani-borealis* (TNS AL-214473). (H) *Amalthea rubida* (TNS AL-220704). (I) *Plocamium brasiliense* (TNS AL-215770). Scale bar =  1 cm (A–G), 3 cm (H), 5 mm (I).

A total of 129 species were detected offshore Tanegashima Island based on molecular data and morphological observations (Fig 2, Table 2, S1–S22 Figs in S4 File, S23–S56 Figs in S5 File). The identification details are presented in S1 Table. Morphologically, 82 species corresponded to known species (Id: C1, C2, T, U2). Among the 82 species, 25 were not identified because molecular data indicated that they were distinct from known species (Id: U2). Among the remaining 57 species, 17 exhibited large intraspecific variation in the sequences assigned to the species in INSD, suggesting that these species have been previously misidentified or include cryptic species (Id: T).

**Table 2:** List of red algae collected from offshore Tanegashima Island.

Species	Identification	Past records on Tanegashima Island and its vicinity	Depth range based on data from our study and the literature	Remarks
Nemaliophycidae, Nemaliales, Galaxauraceae				
*Dichotomaria latifolia* (Tak.Tanaka) S.Fontana, W.L.Wang & Sh.L. Liu (S1 Fig in S4 File)	C1	[19 as *Galaxaura latifolia*]	Near the low-tide line in Taiwan [49 as *G*. *latifolia*]	
*Dichotomaria* sp. 2 TNE (S1 Fig in S4 File)	U3		N/A	The *cox*1 sequence did not closely match the INSD data and was distant from various *Dichotomaria* species (S23 Fig in S5 File).
Nemaliophycidae, Nemaliales, Scinaiaceae				
*Scinaia hormoides* Setchell (S1 Fig in S4 File)	C3	[3,19 as *S*. *moniliformis*]	Subtidal [50 as *S*. *moniliformis*]; 3 m depth in Taiwan [this study]	Morphologically, this species is similar to *S*. *moniliformis*.
*Scinaia* sp. 1 TNE (*S*. cf. *latifrons;* S1 Fig in S4 File)	T1	[3 as *S*. *cottonii*,19 as *S*. *latifrons*]	Lower subtidal [26 as *S*. *cottonii*]; 10–15 m depth on Jeju Island, South Korea [51 as *S*. *latifrons*]	In the *rbc*L analyses, *S*. *latifrons* includes cryptic species (S24 Fig in S5 File).
*Scinaia* sp. 2 TNE (S1 Fig in S4 File)	U3	[3,20 as *S*. *japonica*]	N/A	The *rbc*L and *cox*1 sequences did not closely match the INSD data and were distant from various *Scinaia* species (S24 Fig in S5 File).
Rhodymeniophycidae, Bonnemaisoniales, Bonnemaisoniaceae				
*Delisea japonica* Okamura (S1 Fig in S4 File)	C1	[5 as *D*. *pulchra*,19 as *D*. *fimbriata*]	Low-tide line to lower subtidal [50 as *D*. *fimbriata*]; 10–20 m depth on Jeju Island, South Korea [51]	
Rhodymeniophycidae, Ceramiales, Callithamniaceae				
*Euptilota* sp. JP (S1 Fig in S4 File)	U2	[5,19 as *E*. *articulata*]	Subtidal [50 as *E*. *articulata*]; Approximately 5–10 m depth on Tokunoshima Island, Japan [this study]	In the *rbc*L analyses, *E*. *articulata* recorded in Japan appear to be different species (S26 Fig in S5 File).
Rhodymeniophycidae, Ceramiales, Ceramiaceae				
“*Ceramium*” *nakamurae* E.Y.Dawson (S1 Fig in S4 File)	C2	[14]	N/A	The *rbc*L analyses indicated that *Ceramium nakamurae* belongs to *Ceramothamnion* (S27 Fig in S5 File).
*Delesseriopsis elegans* Okamura (S1 Fig in S4 File)	C2	[8,19]	50 m depth [8]	
*Pterothamnion* sp. TNE (*P*. cf. *yezoense*; S1 Fig in S4 File)	T	[14 as *Antithamnion plumula*, 19 as *Platythamnion yezoense*]	N/A	In the *rbc*L and *cox*1 analyses, *P*. *yezoense* recorded in Japan and South Korea includes cryptic species (S27 Fig in S5 File).
Rhodymeniophycidae, Ceramiales, Delesseriaceae				
*Dasya* sp. TNE (S2 Fig in S4 File)	U3		N/A	The *rbc*L and *cox*1 sequences did not closely match the INSD data and were distant from various *Dasya* species (S28 Fig in S5 File).
Delesseriaceae sp. 1 TNE (S2 Fig in S4 File)	U1		N/A	The *cox*1 sequence did not closely match the INSD data and was distant from various delesseriacean genera (S29 Fig in S5 File).
Delesseriaceae sp. 2 TNE (S2 Fig in S4 File)	U1		N/A	The morphological characteristics and *cox*1 analyses indicated that this species is closely related to Delesseriaceae sp. 1 TNE; however, the *cox*1 sequence divergence indicated that it is distinct from Delesseriaceae sp. 1 TNE (S29 Fig in S5 File).
“*Hypoglossum*” *nipponicum* Yamada (S3 Fig in S4 File)^a^	C2		Subtidal [50 as *H*. *nipponicum*]	The *rbc*L and *cox*1 analyses indicated that *H*. *nipponicum* is not included in the *Hypoglossum* clade and is distant from various delesseriacean genera (S29 Fig in S5 File).
“*Hypoglossum*” *serratifolium* Okamura (S2 Fig in S4 File)^a^	C2		Lower subtidal [26]	The *rbc*L and *cox*1 analyses revealed that this species is not included in the *Hypoglossum* clade and is distant from various delesseriacean genera (S29 Fig in S5 File).
*Martensia* sp. 1 TNE (S2 Fig in S4 File)	U2	[5,19 as *M*. *denticulata*]	Low-tide line to subtidal [26 as *M*. *fragilis*]	The morphological characteristics and *rbc*L analyses indicated that this species is closely related to *M. tsudae*; however, the *cox*1 sequence divergence indicated that it is distinct from *M. tsudae* (S30 Fig in S5 File).
*Martensia* sp. 2 TNE (S2 Fig in S4 File)	U2	[5,19 as *M*. *denticulata*]	Low-tide line to subtidal [26 as *M*. *fragilis*]	The morphological characteristics and *rbc*L analyses indicated that this species is closely related to *M*. sp. 1 TNE; however, the *cox*1 sequence divergence indicated that it is distinct from *M*. sp. 1 TNE (S30 Fig in S5 File).
Nitophylloideae sp. 1 TNE (S2 Fig in S4 File)	U1		N/A	The *rbc*L and *cox*1 sequences did not closely match the INSD data and were distant from various delesseriacean genera (S29 Fig in S5 File).
Nitophylloideae sp. 2 TNE (S2 Fig in S4 File)	U1		N/A	The morphological characteristics and *rbc*L analyses indicated that this species is closely related to Nitophylloideae sp. 1 TNE; however, the *cox*1 sequence divergence indicated that it is distinct from Nitophylloideae sp. 1 TNE (S30 Fig in S5 File).
*Nitophyllum* sp. TNE (S2 Fig in S4 File)	U1		N/A	The *rbc*L and *cox*1 sequences did not closely match the INSD data (S30 Fig in S5 File).
Phycodryoideae sp. 1 TNE (S3 Fig in S4 File)	U3		N/A	The *rbc*L and *cox*1 sequences did not closely match the INSD data (S31 Fig in S5 File).
Phycodryoideae sp. 2 TNE (S3 Fig in S4 File)	U3		N/A	The *rbc*L and *cox*1 sequences did not closely match the INSD data (S31 Fig in S5 File).
Phycodryoideae sp. 3 TNE (S3 Fig in S4 File)	U2		1–10 m depth in Taiwan [53 as *Drachiella liaoi*]	The morphological characteristics and *rbc*L analyses indicated that this species is related to *D*. *liaoi*; however, the *rbc*L sequence divergence indicated that it is distinct from *D*. *liaoi* (S31 Fig in S5 File).
Phycodryoideae sp. 4 TNE (S3 Fig in S4 File)	U2		1–10 m depth in Taiwan [53 as *Drachiella liaoi*]	The morphological characteristics and *rbc*L analyses indicated that this species is related to *D*. *liaoi* and Phycodryoideae sp. 3 TNE; however, the *rbc*L sequence divergence indicated that it is distinct from *D*. *liaoi* and Phycodryoideae sp. 3 TNE (S31 Fig in S5 File).
Phycodryoideae sp. 5 TNE (S3 Fig in S4 File)	U2		1–10 m depth in Taiwan [53 as *Drachiella liaoi*]	The morphological characteristics and *rbc*L analyses indicated that this species is related to *D*. *liaoi* and Phycodryoideae sp. 3 TNE; however, the *rbc*L sequence divergence indicated that it is distinct from *D*. *liaoi* and Phycodryoideae sp. 3 TNE (S31 Fig in S5 File).
*Pseudopolyneura hyacinthina* (J.C.Kang & M.S.Kim) M. J. Wynne (S4 Fig in S4 File and S6 File)^b^	C1		10–30 m depth on Jeju province, South Korea [54 as *Erythroglossum hyacinthinum*]	
“*Sorella*” *pulchra* (Yamada) T.Yoshida & Mikami (S2 Fig in S4 File)^a^	C2		11–16 m depth in Sagami Bay, Japan [52 as *E*. *pulchrum*]	The *rbc*L and *cox*1 analyses indicated that *Sorella pulchra* belongs to *Erythroglossum* (S31 Fig in S5 File).
*Sympodothamnion leptophyllum* (Tak.Tanaka) Itono (S3 Fig in S4 File)^c^	C2	[8,19]	40 m depth [8]	
“*Vanvoorstia*” *coccinea* Harvey ex J.Agardh (S3 Fig in S4 File)	C2	[5,19 as *V*. *coccinea*]	Low-tide line to subtidal [50 as *V*. *coccinea*]	The *rbc*L and *cox*1 analyses indicated that *V*. *coccinea* recorded in Japan is distant form *V*. *spectabilis* and various delesseriacean genera (S29 Fig in S5 File).
*Yoshidaphycus ciliatus* (Okamura) Mikami (S3 Fig in S4 File)^a^	C2		Subtidal in Honshu, the Seto Inland Sea and Kyushu, Japan [26]; 10–20 m depth on Jeju Island, South Korea [51]	
Zinovaeeae sp. TNE (S3 Fig in S4 File)	U1		N/A	The *rbc*L and *cox*1 sequences did not closely match the INSD data and were distant from various delesseriacean genera (S29 Fig in S5 File).
Rhodymeniophycidae, Ceramiales, Rhodomelaceae				
*Acanthophora dendroides* Harvey (S5 Fig in S4 File and S6 File)^b^	C1		27–28 m depth in Dampier Archipelago, Australia [43]	
Amansieae sp. TNE (S6 Fig in S4 File)	U1		N/A	The *cox*1 sequence did not closely match the INSD data and was distant from various amansieaean genera (S32 Fig in S5 File).
*Aneurianna lorentzii* (Weber-van Bosse) L.E.Phillips (S6 Fig in S4 File)	C2	[9 as *Aneuria lorentzii*]	10–30 (–60) m depth [9 as *Aneuria lorentzii*]	
*Aneurianna* sp. TNE (S6 Fig in S4 File)	U2		35 m depth [this study]	The morphological characteristics and molecular analyses indicated that this species is closely related to *A*. *lorentzii*; however, the *rbc*L and *cox*1 sequence divergence indicated that it is distinct from *A*. *lorentzii* (S32 Fig in S5 File).
*Chondria intertexta* P.C.Silva (S6 Fig in S4 File)	C1	[19]	Upper subtidal [26]	
*Chondria mageshimensis* Tak.Tanaka & K.Nozawa (S6 Fig in S4 File)	C2	[8]	30 m depth [8]; Near the low-tide line in the Seto Inland Sea [55]	
*Chondria* sp. 1 TNE (S6 Fig in S4 File)	U2	[8 as *C*. *mageshimensis*]	35 m depth [this study]	The morphological characteristics and *rbc*L analyses indicated that this species is closely related to *C*. *mageshimensis*; however, the *cox*1 sequence divergence indicated that it is distinct from *C*. *mageshimensis* (S33 Fig in S5 File).
*Chondria* sp. 2 TNE (S6 Fig in S4 File)	U2		Shallow subtidal [26 as *C*. *expansa*]; Intertidal in South Korea [28 as *C*. *expansa*]	Morphological characteristics of this species is similar to those of *C. expansa*; however, the *rbc*L and *cox*1 analyses indicated that it is distant from various *Chondria* species, including *C*. *expansa*. (S33 Fig in S5 File).
*Chondria* sp. 3 TNE (S6 Fig in S4 File)	U1		N/A	The *rbc*L and *cox*1 sequences did not closely match the INSD data and were distant from various *Chondria* species (S33 Fig in S5 File).
*Chondria* sp. 4 TNE (S6 Fig in S4 File)	U2		Upper intertidal in South Korea [28 as *C*. *arcuata*]; Lower intertidal to shallow subtidal in Hawaii [38 as *C*. *arcuata*]	Morphological characteristics of this species is similar to those of *C. arcuata*; however, the *rbc*L and *cox*1 analyses indicated that it is distant from various *Chondria* species, including *C*. *arcuata*. (S33 Fig in S5 File).
*Chondrophycus* sp. 1 TNE (S6 Fig in S4 File)	U1		N/A	The *cox*1 sequence did not closely match the INSD data and was distant from various *Chondrophycus* species (S34 Fig in S5 File).
*Chondrophycus* sp. 2 TNE (S7 Fig in S4 File)	U1		N/A	The morphological characteristics and *cox*1 analyses indicated that this species is related to *C*. sp. 1 TNE; however, the *cox*1 sequence divergence indicated that it is distinct from *C*. sp. 1 TNE (S34 Fig in S5 File).
*Laurencia* sp. TNE (S7 Fig in S4 File)	U1		N/A	The *rbc*L and *cox*1 sequence did not closely match the INSD data and were distant from various *Laurencia* species (S34 Fig in S5 File).
*Lophocladia japonica* Yamada (S7 Fig in S4 File)^a^	C2		16 m depth [52]	
*Neurymenia nigricans* Tak.Tanaka & Itono (S7 Fig in S4 File)	C2	[55]	Low-tide line to 15 m [56]	
*Tolypiocladia* sp. TNE (S22 Fig in S4 File)	U2	[5 as *Roschera glomerulata*,20]	Near the low-tide line [50 as *T*. *glomerulata*]	In the *rbc*L analyses, *T*. *glomerulata* recorded in Japan appear to be different species (S36 Fig in S5 File).
*Wrightiella* sp. TNE (S7 Fig in S4 File)	U1		N/A	The *rbc*L and *cox*1 sequence did not closely match the INSD data (S35 Fig in S5 File).
Rhodymeniophycidae, Ceramiales, Wrangeliaceae				
*Anotrichium* sp. TNE (S7 Fig in S4 File)	U3		N/A	The *rbc*L and *cox*1 sequence did not closely match the INSD data (S37 Fig in S5 File).
*Griffithsia venusta* Yamada (S7 Fig in S4 File)	C2	[14,19]	13 m depth [52]; 5–15 m depth on Jeju Island, South Korea [51]	
“*Griffithsia*” sp. 1 TNE (*G*. cf. *subcylindrica*; S7 Fig in S4 File)	T	[14,19 as *G*. *subcylindrica*]	Low-tide line to subtidal [50 as *G*. *subcylindrica*]	In the *cox*1 analyses, *G*. *subcylindrica* includes cryptic species (S37 Fig in S5 File).
“*Griffithsia*” sp. 2 TNE (S7 Fig in S4 File)	U2	[5,14,19 as *G*. *japonica*]	Intertidal to subtaidal [50 as *G*. *japonica*]	The morphological characteristics and *rbc*L analyses indicated that this species is closely related to *G*. *japonica*; however, the *cox*1 sequence divergence indicated that it is distinct from *G*. *japonica* (S37 Fig in S5 File).
*Pleonosporium* sp. TNE (S7 Fig in S4 File)	U3		N/A	The *rbc*L and *cox*1 sequence did not closely match the INSD data and were distant from various *Pleonosporium* species (S37 Fig in S5 File).
*Wrangelia tagoi* (Okamura) Okamura & Segawa (S8 Fig in S4 File)	C2	[14,19]	Low-tide line to subtidal [50]	
*Wrangelia* sp. TNE (*W*. cf. *tanegana*; S8 Fig in S4 File)	T1	[5 as *W*. *argus*], [14,19 as *W*. *tayloriana*]	Near the low-tide line [50 as *W*. *argus*]	In the *rbc*L analyses, *W*. *tanegata* includes cryptic species (S37 Fig in S5 File).
Rhodymeniophycidae, Gigartinales, Cystocloniaceae				
*Calliblepharis saidana* (Holmes) M.Y.Yang & M.S.Kim (S8 Fig in S4 File)^a^	C1		Intertidal to subtidal in middle part of Honshu, Japan and South Korea [36,50].	
*Calliblepharis yasutakei* Paiano & A.R.Sherwood (S9 Fig in S4 File and S6 File)^b^	C1		98 m depth in Hawaii, U.S.A. [57]	
*Calliblepharis* sp. 1 TNE (S8 Fig in S4 File)	U1		N/A	The *rbc*L and *cox*1 sequence did not closely match the INSD data and were distant from various *Calliblepharis* species (S38 Fig in S5 File).
*Calliblepharis* sp. 2 TNE (S8 Fig in S4 File)	U1		N/A	The *rbc*L and *cox*1 sequence did not closely match the INSD data and were distant from various *Calliblepharis* species (S38 Fig in S5 File).
*Hypnea yamadae* Tak.Tanaka (S8 Fig in S4 File)^a^	C1		Upper subtidal [6]	
Rhodymeniophycidae, Gigartinales, Furcellariaceae				
*Halarachnion latissimum* Okamura (S8 Fig in S4 File)^a^	C1		Lower subtidal [26]; Near the low-tide line on Awaji Island, Japan [this study]	
Rhodymeniophycidae, Gigartinales, Gigartinaceae				
*Chondracanthus saundersii* C.W.Schneider & C.E.Lane (S8 Fig in S4 File)	C1	[17]	0.5–6 m depth in Bermuda [58]; intertidal to 15 m depth in Brazil [59]	This specimen was recorded from offshore of Tanegashima Island (as Mageshima Island) as a new record for Japan [17].
Rhodymeniophycidae, Gigartinales, Kallymeniaceae				
*Austrokallymenia* sp. 1 (*Kallymenia* cf. *sessilis*; S8 Fig in S4 File)^a^	T		Subtidal [26 as *K*. *sessilis*]; 5–10 m depth on Jeju Island, South Korea [51 as *K*. *sessilis*]	In the *rbc*L and *cox*1 analyses, *K*. *sessilis* includes cryptic species and is included in the *Austrokallymenia* clade (S40 Fig in S5 File).
*Callophyllis* sp. 1 TNE (*C*. cf. *adhaerens*; S8 Fig in S4 File)^a^	T		Subtidal [50 as *C*. *adhaerens*]; 15 m depth on Jeju Island, South Korea [51 as *C*. *adhaerens*]	The morphological characteristics and *rbc*L analyses indicated that this species is closely related to *C*. *adhaerens*; however, the *cox*1 sequence divergence indicated that it is distinct from *C*. *adhaerens* (S41 Fig in S5 File).
*Callophyllis* sp. 2 TNE (S10 Fig in S4 File)	U3		N/A	The *rbc*L and *cox*1 sequence did not closely match the INSD data and were distant from various *Callophyllis* species (S41 Fig in S5 File).
*Croisettea kalaukapuae* F.P.Cabrera & A.R.Sherwood (S11 Fig in S4 File and S6 File)^b^	C1		83–85 m depth in Hawaii, U.S.A. [60]	
*Croisettea* sp. TNE (S10 Fig in S4 File)	U2		35 m depth [this study]	Morphological characteristics of this species is similar to those of *C. kalaukapuae*; however, the *rbc*L and *cox*1 analyses indicated that it is distant from various *Croisettea* species. (S42 Fig in S5 File).
“*Kallymenia*” *perfolata* J.Agardh (S10 Fig in S4 File)	C2	[5,19]	Near the low-tide line [26]	In the *rbc*L and *cox*1 analyses, *K*. *perfolata* recorded in Japan was included in the *Leiomenia* clade (S40 Fig in S5 File).
Kallymeniaceae sp. TNE (S10 Fig in S4 File)	U1		N/A	The *rbc*L and *cox*1 sequence did not closely match the INSD data and were distant from various kallymeniacean genera (S40 Fig in S5 File).
*Psaromenia* sp. 1 JP (*Kallymenia* cf. *crassiuscula*; S10 Fig in S4 File)^a^	T		Lower subtidal [26 as *K*. *crassiuscula*]	In the *rbc*L and *cox*1 analyses, *K*. *crassiuscula* includes cryptic species and is included in the *Psaromenia* clade (S40 Fig in S5 File).
*Psaromenia* sp. 2 TNE (*Kallymenia* cf. *crassiuscula*; S10 Fig in S4 File)^a^	T		Lower subtidal [26 as *K*. *crassiuscula*]	Morphological characteristics of this species is similar to those of *P.* sp. 1 JP and *K*. *crassiuscula*; however, the *rbc*L and *cox*1 analyses indicated that it is distant from *P.* sp. 1 JP and various *Psaromenia* species. (S40 Fig in S5 File).
Rhodymeniophycidae, Gigartinales, Phyllophoraceae				
*Stenogramma guleopoense* M.S.Calderón & S.M.Boo (S12 Fig in S4 File and S7 File)^b^	C1		5–10 m depth on Guleopdo Isle, South Korea [61]	
*Stenogramma lamyi* L.Le Gall (S13 Fig in S4 File and S6 File)^b^	C3		6 m depth in Madagascar [62]	Vegetative anatomy of Japanese specimens was different from that of Malagasy specimens.
Rhodymeniophycidae, Gigartinales, Solieriaceae				
*Solieria pacifica* (Yamada) T.Yoshida (S10 Fig in S4 File)	C1	[5,19 as *S*. *robusta*]	Near the low-tide line to 35 m depth [26,63]	
Rhodymeniophycidae, Gracilariales, Gracilariaceae				
*Gracilaria punctata* (Okamura) Yamada (S10 Fig in S4 File)^a^	C1		Tide pools and subtidal [64]; Tide pools and 1–2 m depth in Taiwan [65]	
*Gracilaria sublittoralis* Yamada & Segawa ex H.Yamamoto (S10 Fig in S4 File)	C2	[7,19]	Subtidal, up to 50 m depth [7,26]	
*Gracilaria* sp. 1 TNE (S10 Fig in S4 File)	U3		N/A	The *rbc*L and *cox*1 sequences did not closely match the INSD data and were distant from various *Gracilaria* species (S45 Fig in S5 File).
*Gracilaria* sp. 2 TNE (S14 Fig in S4 File)	U1		N/A	The *rbc*L sequence did not closely match the INSD data and was distant from various *Gracilaria* species, whereas the *cox*1 sequence was 99.5% identical to that of *G*. sp. ARS 03323 from Hawaii (S45 Fig in S5 File).
*Gracilaria* sp. 3 TNE (*G*. cf. *articulata*; S14 Fig in S4 File)^a^	T		Lower intertidal [66 as *G*. *articulata*]	In the *rbc*L analyses, *G*. *articulata* includes cryptic species (S45 Fig in S5 File).
*Gracilaria* sp. 4 TNE (S14 Fig in S4 File)	U1		N/A	The *rbc*L and *cox*1 sequences did not closely match the INSD data and were distant from various *Gracilaria* species (S45 Fig in S5 File).
*Gracilariopsis mageshimensis* Mas.Suzuki & R.Terada (S14 Fig in S4 File)^c^	C1	[18]	35 m depth [18]	This species was described from offshore of Tanegashima Island (as Mageshima Island) [18].
Rhodymeniophycidae, Halymeniales, Grateloupiaceae				
*Yonagunia taiwani-borealis* Showe M.Lin, Y.C.Chuang & De Clerck (S15 Fig in S4 File and S6 File)^b^	C1		Shallow subtidal in Taiwan [67]	
*Yonagunia* sp. TNE (S14 Fig in S4 File)	U2		35 m depth [this study]	The morphological characteristics and *rbc*L analyses indicated that this species is closely related to *Y. taiwani-borealis*; however, the *cox*1 sequence divergence indicated that it is distinct from *Y. taiwani-borealis* (S47 Fig in S5 File).
Rhodymeniophycidae, Halymeniales, Halymeniaceae				
*Amalthea rubida* H.W.Lee & M.S.Kim (S16 Fig in S4 File and S6 File)^b^	C1		10–20 m depth on Jeju Island, South Korea [51,68]	
*Amalthea* sp. 1 Tane (S14 Fig in S4 File)	U2		35 m depth [this study]	Morphological characteristics of this species is similar to those of *A. rubida*; however, the *rbc*L analyses indicated that it is distant from various *Amalthea* species. (S47 Fig in S5 File).
*Amalthea* sp. 2 TNE (S14 Fig in S4 File)	U2		35 m depth [this study]	Morphological characteristics of this species is similar to those of *A. rubida*; however, the *rbc*L analyses indicated that it is distant from various *Amalthea* species. (S47 Fig in S5 File).
*Amalthea* sp. 3 TNE (S14 Fig in S4 File)	U2		35 m depth [this study]	Morphological characteristics of this species is similar to those of *A. rubida*; however, the *rbc*L analyses indicated that it is distant from various *Amalthea* species. (S47 Fig in S5 File).
*Amalthea* sp. 4 TNE (S14 Fig in S4 File)	U1		35 m depth [this study]	The *rbc*L sequence did not closely match the INSD data and were distant from various *Amalthea* species (S47 Fig in S5 File).
*Cryptonemia semiprocumbens* Tak.Tanaka (S14 Fig in S4 File)^a^	C2		30–60 m depth [69]	
*Cryptonemia* sp. TNE (S17 Fig in S4 File)	U2	[35 as *C*. *luxurians*]	Subtidal [69 as *C*. *luxurians*]	Morphological characteristics of this species is similar to those of *C. asiatica*; however, the *rbc*L analyses indicated that it is distant from various *Cryptonemia* species, including *C. asiatica*. (S47 Fig in S5 File).
*Galene* sp. 1 TNE (S17 Fig in S4 File)	U1		N/A	The *rbc*L and *cox*1 sequences did not closely match the INSD data and were distant from various *Galene* species (S47 Fig in S5 File).
*Galene* sp. 2 TNE (S17 Fig in S4 File)	U3		N/A	The *rbc*L sequence was 99.2% identical to that of *G*. sp. 2 LH from Lord Howe Island, Australia.
*Galene* sp. 3 TNE (S17 Fig in S4 File)	U1		N/A	The *rbc*L and *cox*1 sequences were 99.0% amd 97.7% identical to those of *G*. sp. 1 WA from Australia.
*Halymenia* sp. TNE (*H*. cf. *durvillei*; S17 Fig in S4 File)	T	[5,19 as *H*. *durvillei* var. *formosa*]	Subtidal [26 as *H*. *floresii*]	In the *rbc*L and *cox*1 analyses, *H*. *durvillei* includes cryptic species (S47 Fig in S5 File).
Halymeniaceae sp. 1 TNE (S17 Fig in S4 File)	U1		N/A	The *rbc*L sequence did not closely match the INSD data and was distant from various halymeniaceaen genera (S47 Fig in S5 File).
Halymeniaceae sp. 2 TNE (S17 Fig in S4 File)	U3		N/A	The *rbc*L sequence did not closely match the INSD data and was distant from various halymeniaceaen genera (S47 Fig in S5 File).
Halymeniaceae sp. 3 TNE (S17 Fig in S4 File)	U1		N/A	The *rbc*L and *cox*1 sequences did not closely match the INSD data and were distant from various halymeniaceaen genera (S47 Fig in S5 File).
Rhodymeniophycidae, Nemastomatales, Schizymeniaceae				
*Platoma* sp. TNE (S18 Fig in S4 File)	U1			The *rbc*L and *cox*1 sequences did not closely match the INSD data and were distant from various *Platoma* species (S48 Fig in S5 File).
Rhodymeniophycidae, Peyssonneliales, Peyssonneliaceae				
*Agissea* sp. 1 TNE (S18 Fig in S4 File)	U2		5–10 m depth [70 as *Peyssonnelia conchicola*]; 36–45 m depth in Hawaii [38 as *P*. *conchicola*]	Morphological characteristics of this species is similar to those of *P*. *conchicola*; however, the *rbc*L analyses indicated that it is included in the *Agissea* clade (S49 Fig in S5 File).
*Agissea* sp. 2 TNE (*A*. cf. *orientalis*; S18 Fig in S4 File)^a^	T		60 m depth [70 as *Peyssonnelia orientalis*]	In the *rbc*L analyses, *A*. *orientalis* includes cryptic species (S49 Fig in S5 File).
*Incendia* sp. TNE (S18 Fig in S4 File)	U3		N/A	The *rbc*L and *cox*1 sequences did not closely match the INSD data and were distant from various *Incendia* species (S49 Fig in S5 File).
Rhodymeniophycidae, Plocamiales, Plocamiaceae				
*Plocamium brasiliense* (Greville) M.Howe & W.R.Taylor (S19 Fig in S4 File and S6 File)^b^	C1		32 m depth in Enseada do Flamengo, Brazil [71]	
*Plocamium luculentum* M.Y.Yang & M.S.Kim (S18 Fig in S4 File)	C1	[5,19 as *P*. *telfairiae*]	Lower intertidal to subtidal [50 as *P*. *telfairiae*]; intertidal to 20 m depth on Jeju Island, South Korea [51 as *P*. *telfairiae*]	
*Plocamium ovicorne* Okamura (S18 Fig in S4 File)	C1	[19]	Subtidal [26]; 10–20 m depth on Jeju Island, South Korea [51]	
*Plocamium* sp. TNE (S18 Fig in S4 File)	U3		N/A	The *rbc*L sequences was 99.3% identical to those of *P*. sp. Asia from South Korea (S50 Fig in S5 File).
*Sarcodia* sp. JP1 (S18 Fig in S4 File)	U2	[19 as *S*. *ceylanica*]	Lower subtidal [50 as *S*. *ceylanica*]	In the *rbc*L analyses, *S*. *ceylanica* recorded in Japan appear to be different species (S50 Fig in S5 File).
Rhodymeniophycidae, Rhodymeniales, Champiaceae				
*Champia expansa* Yendo (S18 Fig in S4 File)^a^	C3		Lower subtidal in Honshu and Kyushu, Japan [26]; 10–15 m depth on Jeju Island, South Korea [51]	Habit of specimen collected from offshore Tanegashima Island differs from that of *C*. *expansa*.
*Champia* sp. 1 TNE (S18 Fig in S4 File)	U2	[5,19 as *C*. *parvula*]	Intertidal [50 as *C*. *parvula*]	The morphological characteristics and molecular analyses indicated that this species is related to *C*. *recta*; however, the *rbc*L and *cox*1 sequence divergence indicated that it is distinct from *C*. *recta* (S51 Fig in S5 File).
*Champia* sp. 2 TNE (S20 Fig in S4 File)	U3		N/A	The *rbc*L and *cox*1 sequences did not closely match the INSD data and were distant from various *Champia* species (S51 Fig in S5 File).
*Champia* sp. 3 TNE (*C*. cf. *vieillardii*; S20 Fig in S4 File)^a^	T		N/A	In the *rbc*L analyses, *C*. *vieillardii* includes cryptic species (S49 Fig in S5 File).
*Champia* sp. 4 TNE (S20 Fig in S4 File)	U3		N/A	The *rbc*L and *cox*1 sequences did not closely match the INSD data and were distant from various *Champia* species (S51 Fig in S5 File).
*Champia* sp. 5 TNE (S20 Fig in S4 File)	U1		N/A	The *rbc*L and *cox*1 sequences did not closely match the INSD data and were distant from various *Champia* species (S51 Fig in S5 File).
Rhodymeniophycidae, Rhodymeniales, Faucheaceae				
*Gloiocladia* sp. 1 TNE (S20 Fig in S4 File)	U2		3–12 m depth in Australia [40 as *G*. *polycarpa*]	Morphological characteristics of this species is similar to those of *G*. *polycarpa*; however, the *rbc*L and *cox*1 analyses indicated that it is distant from various *Gloiocladia* species. (S52 Fig in S5 File).
*Gloiocladia* sp. 2 TNE (S20 Fig in S4 File)	U2		Lower subtidal [50 as *Gloioderma iyoensis*]; Deep subtidal in South Korea [33 as *G*. *iyoensis*]	Morphological characteristics of this species is similar to those of *G*. *iyoensis*; however, the *rbc*L and *cox*1 analyses indicated that it is distant from various *Gloiocladia* species, including *G*. *iyoensis*. (S52 Fig in S5 File). The *rbc*L sequence is 99.3% identical to that of *G*. sp. GiyoGF2005 from Australia.
Rhodymeniophycidae, Rhodymeniales, Rhodymeniaceae				
Lomentariaceae sp. TNE (S20 Fig in S4 File)	U1		N/A	The *rbc*L and *cox*1 sequences did not closely match the INSD data and were distant from various lomentariacean genera (S53 Fig in S5 File).
Rhodymeniophycidae, Rhodymeniales, Rhodymeniaceae				
*Botryocladia leptopoda* (J.Agardh) Kylin (S20 Fig in S4 Fig)	C1	[43]	Intertidal and subtidal to a depth of 14 m in Australia [42]	
*Botryocladia* sp. TNE (*B*. cf. *kuckuckii*; S20 Fig in S4 File)^a^	T		Lower intertidal [50 as *B*. *kuckuckii*]	In the *rbc*L and *cox*1 analyses, *B*. *kuckuckii* includes cryptic species (S54 Fig in S5 File).
*“Chamaebotrys” lomentariae* (Tak.Tanaka & K.Nozawa) Huisman (S20 Fig in S4 File)^c^	C2	[7]	30–45 m depth [7,72]	The *rbc*L and *cox*1 analyses revealed that this species is included in the *Halopeltis* clade (S54 Fig in S5 File).
*Chamaebotrys* sp. 1 TNE (*C.* cf. *boergesenii*; S21 Fig in S4 File) ^a^	T		Subtidal [50 as *C. boergesenii*]	In the *rbc*L and *cox*1 analyses, *C. boergesenii* includes cryptic species (S54 Fig in S5 File).
*Chamaebotrys* sp. 2 TNE (*C.* cf. *boergesenii*; S21 Fig in S4 File)^a^	T		Subtidal [50 as *C. boergesenii*]	Morphological characteristics of this species is similar to those of *C.* sp. 1 TNE and *C*. *boergesenii*; however, the *rbc*L and *cox*1 analyses indicated that it is distant from *C.* sp. 1 TNE and *C*. *boergesenii*. (S54 Fig in S5 File).
*Chrysymenia* sp. TNE (S21 Fig in S4 File)	U1		N/A	The *rbc*L and *cox*1 sequences did not closely match the INSD data and were distant from various *Chrysymenia* species (S54 Fig in S5 File).
*Drouetia* sp. TNE (S21 Fig in S4 File)	U2		N/A	Morphological characteristics of this species is similar to those of *D*. *viridescens*; however, the *rbc*L and *cox*1 analyses indicated that it is distant from various *Drouetia* species, including **D*. viridescens*. (S54 Fig in S5 File).
*Halichrysis* sp. TNE (S21 Fig in S4 File)	U3		N/A	The *rbc*L and *cox*1 sequences did not closely match the INSD data and were distant from various *Halichrysis* species (S54 Fig in S5 File).
*Halopeltis tanakae* Mas.Suzuki & R.Terada (S21 Fig in S4 File)^c^	C1	[8 as *Rhodymenia prostrata*,^72^]	35–50 m depth [6 as *R*. *prostrata*,^73^]	
*Halopeltis* sp. 1 TNE (S21 Fig in S4 File)	U2		35 m depth [this study]	The morphological characteristics and molecular analyses indicated that this species is related to *H*. *tanakae*; however, the *rbc*L and *cox*1 sequence divergence indicated that it is distinct from *H*. *tanakae* (S54 Fig in S5 File).
*Halopeltis* sp. 2 TNE (*H*. cf. *adnata*; S21 Fig in S4 File)^a^	T		Lower subtidal [74 as *Rhodymenia adnata*]; 5–20 m depth on Jeju Island, South Korea [51 as *H*. *adnata*]	In the *rbc*L and *cox*1 analyses, *H. adnata* includes cryptic species (S54 Fig in S5 File).
Rhodymeniophycidae, Sebdeniales, Sebdeniaceae				
*Sebdenia* sp. TNE (*S*. cf. *flabellata*; S22 Fig in S4 File)	T	[5,19 as *Halymenia agardii*]	Lower subtidal [5 as *H*. *agardii*,50 as *H*. *agardii*]; 5–15 m depth on Jeju Island, South Korea [51 as *S*. *flabellata*]	In the *rbc*L and *cox*1 analyses, *S. flabellata* includes cryptic species (S55 Fig in S5 File).
Sebdeniaceae sp. 1 TNE (S22 Fig in S4 File)	U1		N/A	The *rbc*L and *cox*1 sequences were 99.8% amd 98.9% identical to those of Sebdeniaceae sp. 1 GWS-2011 from Australia.
Rhodymeniophycidae, Incertae sedis, Calosiphoniaceae				
*Schmitzia* sp. TNE (S22 Fig in S4 File)	U3		N/A	The *rbc*L sequence did not closely match the INSD data (S56 Fig in S5 File).

^a^New record for offshore Tanegashima Island and its vicinity.

^b^New record for Japan.

^c^Endemic to offshore Tanegashima Island.

Three species were identified based on the molecular data (Id: C3); however, they did not morphologically match the compared species and further investigations, including more specimens from various regions, are required to clarify the morphological variability among the specimens. We could not identify 17 species because the *rbc*L and *cox*1 sequences did not closely match the INSD data and they did not exhibit distinguishable or reliable morphological characteristics (Id: U3). The remaining 27 taxa lacked similar data in INSD and did not morphologically match any species currently recorded in the northwestern Pacific (Id: U1). In total, 43 species were identified completely (Id: C1–C3).

### Diversity

Among the 60 species identified or tentatively identified in the present study (Id: C1–C3, T), 21 species were new records for the offshore Tanegashima Island and its vicinity, and four species were endemic to the offshore Tanegashima Island (Table 2). Nine species were newly recorded in Japan (Fig 2). The detailed morphological observations and identification of newly recorded species in Japan are presented in S6 File.

## Discussion

### Identification

Morphologically, 82 species were assigned to known species in this study. However, molecular data revealed that there were issues with identification of 42 species. Among the 42, 17 included cryptic species. We tentatively identified these species until taxonomic problems were clarified based on type specimens or samples from a type locality. According to the molecular data, the names of the remaining 25 species recorded in Japan appeared to have misapplied names. In addition, at least 27 species did not morphologically match any species recorded in Japan. These results suggest that 52 species may represent new species or species recorded outside Japan for which DNA sequence data are not yet available. Further taxonomic studies based on morphological and DNA-based approaches are needed to confirm the novelty of these species.

### DNA barcoding

Although 129 species were recognized, less than half were identified to the species level. Furthermore, the identification of the 17 species was incomplete; thus, further taxonomic studies are needed. It appears that the available DNA sequences in INSD were not sufficient to complete the DNA barcoding of the marine flora of offshore Tanegashima Island and the Japanese marine flora. Comparing the number of species for which *rbc*L or *cox*1 sequences were available in INSD to the 976 red algal species recorded in Japan [75], we found that 590 *rbc*L or *cox*1 sequences assigned to Japanese species were available in INSD. Among them, 348 sequences were determined from Japanese specimens. Although approximately 60% of the species assigned to Japanese species had sequences available in INSD, we could only identify approximately 19% to the species level based on high BLAST scores. Additionally, the sequences of seven other taxa most closely matched sequences that were unidentified at the species or genus level, labeled as “sp.,” and the sequences of four other taxa most closely matched temporarily misidentified sequences (S50 Table). These results indicate that the DNA barcoding reference for Japanese red algae is incomplete, and DNA barcoding failed to identify a large amount of their diversity.

Vieira et al. [23] conducted a DNA-based floristic survey of marine macroalgae in northern Madagascar and identified 89 unique taxa. Among the 89 taxa, approximately 36% could be identified to the species level. They also noted the necessity of a detailed reference library containing validated DNA barcodes. Further taxonomic studies will increase the number of validated sequences in public databases and are needed to explore the identity of samples with low BLAST scores.

### Diversity

This study provides the first comprehensive catalog of red algae identified the sublittoral marine flora of offshore Tanegashima Island, Japan, and the first exhaustive molecular-assisted survey of red algal marine flora in Japan. Among the 60 species identified in this study, four were considered endemic. The number of species recognized in this study was much higher than that of MCEs in Hawaii and Ryukyu Island, Japan. In total, 72 species, including 31 red algae, have been recorded in Hawaiian MCEs [76], whereas 102 species, including 56 red algae, have been recorded in the MCEs around Ryukyu Island [77]. The identification of macroalgae recorded on Hawaii and Ryukyu Island was morphologically based. Therefore, the number of species on Hawaii and Ryukyu Island will most likely increase with additional morpho-molecular analyses.

We recognize that a floristic survey on offshore Tanegashima Island has not yet been completed. We could not extract DNA from small-sized and epiphytic algae such as the species belonging to Acrochaetiales, Ceramiales, Colaconematales, Erythropeltidales, and Stylonematales. The addition of these species in the flora via a molecular approach would require unialgal culture strains. At least two nongeniculate coralline species and a small fragment of *Meristotheca* species have also been collected. The number of species offshore Tanegashima Island will increase with further collections and morpho-molecular analyses.

The mesophotic flora of offshore Tanegashima Island includes species distributed throughout Hawaii and Australia. *Calliblepharis yasutakei*, *Croisettea kalaukapuae*, *Scinaia hormoides*, and *Gracilaria* sp. 2 TNE are distributed offshore Tanegashima Island and in Hawaii, whereas *Botryocladia leptopoda*, *Galene* sp. 2 TNE, *Galene* sp. 3 TNE, *Gloiocladia* sp. 2 TNE, and Sebdeniaceae sp. TNE are distributed offshore Tanegashima Island and in Australia. Given that Kawai et al. [16] reported the widespread distribution of *Ryuguphycus kuaweuweu*, a deep-water green algal species, in Hawaii, Japan, New Zealand, and Australia, it is possible that these deep-water species might be widely distributed in the MCEs of the Pacific Ocean.

Notably, some species that were described are distributed in regions distinct from Japan. *Stenogramma lamyi* was originally described by Le Gall et al. [63] in Manantenina, Madagascar. *Plocamium brasiliense* was recorded in the western Atlantic [72]. Soares and Fuji [78] and Campbell et al. [79] reported *Calliblepharis saidana*, which is distributed in Japan, is from North Carolina (USA) and Brazil. Suzuki et al. [17] recorded *Chondracanthus saundersii*, which is distributed in the western Atlantic, from offshore Tanegashima Island. Unfortunately, we do not yet have a hypothesis that can clearly explain the geographic factors influencing the distribution of these species because of the limited number of records. Further investigations, including those that use more specimens from various regions in the Indian and Atlantic Oceans, are needed to clarify the distribution patterns of these species.

Among the 85 species identified or corresponding to known species recorded in the northwestern Pacific in this study, 84% were the same as those usually found growing from the lower intertidal to shallow subtidal zones (up to 20 m depth) in various parts of Japan and in the vicinity of Japan, whereas 16% were only found at depths below 30 m (Table 2). Many species that appear offshore Tanegashima Island do indeed grow in shallower depths. The seawater temperature is consistently 2–5°C lower at the sea floor compared with the sea surface in spring to summer, but the thermocline disappears in autumn [25]. These temperature environments may allow the shallow water species to flourish below 30 m.

Alternatively, species growing offshore Tanegashima Island may undergo adaptation to low-light environments [25]. In fact, Borlongan et al. [63] reported that *Solieria pacifica* collected at a depth of 35 m offshore Tanegashima Island showed different temperature optima for photosynthesis compared with the species collected at a depth of 5 m. Additional investigations of other species are necessary to clarify adaptive strategies against deep-water conditions.

In contrast, for species flourishing in MCEs in Hawaii, approximately 45% are unique to their environments [76]. As the occurrence depth of seaweeds in Hawaiian MCEs is much deeper than offshore Tanegashima Island (up to 120 m), it appears that the characteristics of MCEs may differ by region and depth environment. Further comprehensive floristic studies based on molecular data for MCEs from various regions are needed to elucidate the marine flora characteristics of each MCE.

## Conclusions

This study revealed the cryptic diversity of sublittoral algae in Japan based on exhaustive molecular-assisted surveys and contributes to increasing the sequences available for DNA barcoding from offshore Tanegashima Island. However, it also revealed the necessity of taxonomically validated reference libraries in INSD for DNA barcoding. Further taxonomic studies based on morphological and DNA-based approaches will be required to mature the reference libraries and assess marine algal biodiversity.

## Supporting information

S1 File Collection locations and details, and INSD (DDBJ/EMBL/GenBank) and BOLD accession numbers of the samples used in the *rbc*L and *cox*1 sequence analyses. Accession numbers in bold were determined for this study.
(XLSX)

S2 File Descriptions of maximum likelihood (ML) and Bayesian inference (BI) phylogenetic analyses. (DOCX)

S3 File Summary of maximum likelihood and Bayesian phylogenetic analyses on 77 datasets. (XLSX)

S4 File Supplementary figures. Habits and herbarium specimens of red algae collected from offshore Tanegashima Island.
(PDF)

S5 File Supplementary figures. Maximum likelihood phylogeny of red algae collected from offshore Tanegashima Island.
(ZIP)

S6 File Taxonomic information and morpho-anatomical observations of newly recorded species for Japan. (DOCX)

S7 File List of red algae from offshore of Tanegashima Island and the identification details. (DOCX)
