# Correction: DNA-based floristic survey of red algae (Rhodophyta) growing in the mesophotic coral ecosystems (MCEs) offshore of Tanegashima Island, northern Ryukyu Archipelago, Japan

**DOI:** 10.1371/journal.pone.0325606

**Published:** 2025-05-27

**Authors:** The PLOS One Staff

## Notice of Republication

This article was republished on May 16, 2025, to correct incorrect supporting information files that were originally included. The publisher apologizes for the errors. Please download the article again to view the corrected version.
