# Supplementary material for: DNA-based floristic survey of red algae (Rhodophyta) growing in the mesophotic coral ecosystems (MCEs) offshore of Tanegashima Island, northern Ryukyu Archipelago, Japan
Source: PLoS One. 2025 Mar 10;20(3):e0316067. doi: 10.1371/journal.pone.0316067 (PMC11893125; doi:10.1371/journal.pone.0316067)
Supplement: S2 File — (DOCX) [file pone.0316067.s002.docx]

**S2 File. Descriptions of maximum likelihood (ML) and Bayesian inference (BI) phylogenetic analyses.**

The *rbc*L and *cox*1 sequences were aligned using ClustalW [1]. The ML analyses were performed using RAxML-NG v1.0.1 [2]. To find the best tree, 500 random and 500 parsimony-based starting trees. Bootstrap values (BP) for the ML analyses were calculated based on 1,000 pseudoreplicates. The BI analyses were performed using MrBayes 3.2.7a [3]. The BI analyses were initiated with a random starting tree and four chains of Markov chain Monte Carlo iterations were run for 20,000,000 to 70,000,000 generations, with one tree kept every 500 generations. The convergence of the loglikelihood and parameter values was assessed using Tracer ver.1.7.1 [4]. The first 25% of the generations were discarded as burn-in before constructing the majority rule consensus tree; the remaining trees were used to calculate a 50% majority rule tree and determine the posterior probabilities (PPs) of individual branches. The number of taxa, the selection of outgroups, the length of alignments, the number of MCMC iterations for BI analyses, and the substitution models for ML and BI analyses for each dataset were summarized in S14–S47 Tables in S3 File. The *p*-distances for each pair of specimens were calculated using PAUP 4.0b10 software [5].

References

1. Larkin MA, Blackshields G, Brown NP, Chenna R, McGettigan PA, McWilliam H, et al. Clustal W and Clustal X version 2.0. Bioinfomatics. 2007; 23: 2947–2948. https://doi.org/10.1093/bioinformatics/btm404
2. Kozlov AM, Darriba D, Flouri T, Morel B, Stamatakis A. RAxML-NG: a fast, scalable and user-friendly tool for maximum likelihood phylogenetic inference. Bioinformatics. 2019; 35: 4453–4455. https://doi.org/10.1093/bioinformatics/btz305
3. Ronquist, F., Teslenko, M., van der Mark, P., Ayres, D.L., Darling, A., Höhna, S., Larget B, Liu L, Suchard MA, Huelsenbeck JP. MrBayes 3.2: Effient Bayesian phylogenetic inference and model choice across a large model space. Syst Biol. 2012; 61: 539–542. https://doi.org/10.1093/sysbio/sys029
4. Rambaut A, Drummond AJ, Xie D, Baele G, Suchard MA. Posterior summarisation in Bayesian phylogenetics using Tracer 1.7. Syst Biol. 2018; 67: 901–904. https://doi.org/10.1093/sysbio/syy032
5. Swofford DL. PAUP*. Phylogenetic analysis using parsimony (*and other methods), Version 4.0 Beta 10. Sunderland: Sinauer Associate; 2002.
