# Supplementary material for: DNA-based floristic survey of red algae (Rhodophyta) growing in the mesophotic coral ecosystems (MCEs) offshore of Tanegashima Island, northern Ryukyu Archipelago, Japan
Source: PLoS One. 2025 Mar 10;20(3):e0316067. doi: 10.1371/journal.pone.0316067 (PMC11893125; doi:10.1371/journal.pone.0316067)
Supplement: S4 File — Habits and herbarium specimens of red algae collected from offshore Tanegashima Island. (PDF) [file pone.0316067.s004.pdf]

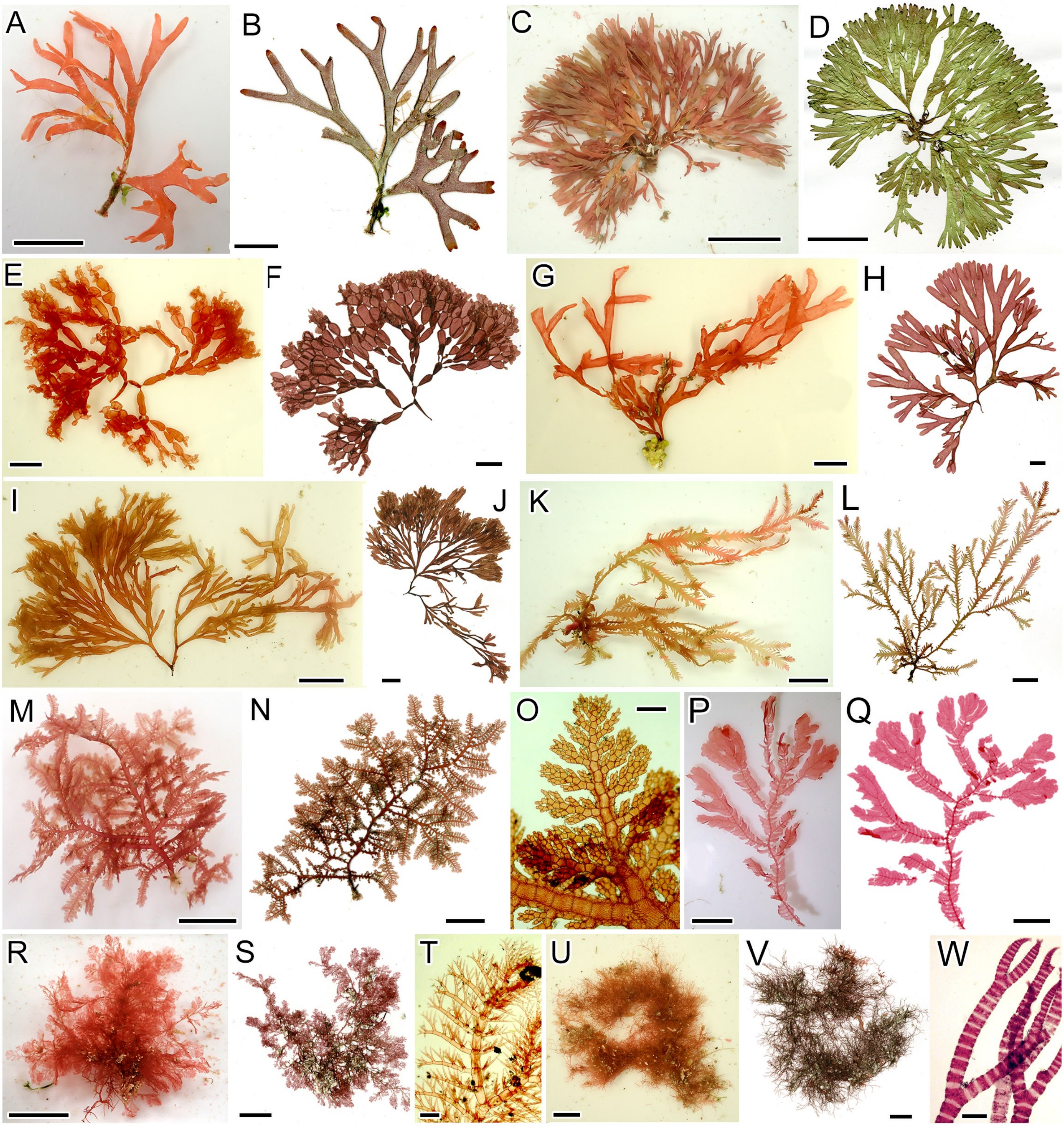

S1 Fig. Habits and herbarium specimens of red algae collected from offshore Tanegashima Island. (A, B) *Dichotomaria latifolia* (TNS AL-215728). (C, D) *Dichotomaria* sp. TNE (TNS AL-222169). (E, F) *Scinaia hormoides* (TNS AL-209756). (G, H) *Scinaia* sp.1 TNE (*S*. cf. *latifrons*; TNS AL-209755). (I, J) *Scinaia* sp.2 TNE (TNS AL-209760). (K, L) *Delisea japonica* (TNS AL-215738). (M–O) *Euptilota* sp. JP (TNS AL-209879). (P, Q) *Delesseriopsis elegans* (TNS AL-200146). (R–T) *Pterothamnion* sp. TNE (*P*. cf. *yezoense*; TNS AL-220687). (U–W) "*Ceramium*" *nakamurae* (TNS AL-220685). Scale bar = 1 cm (A, B, E–L, R, S, U, V), 5 mm (C, D, M, N, P, Q), 200 µm (O, W), 50 µm (T).

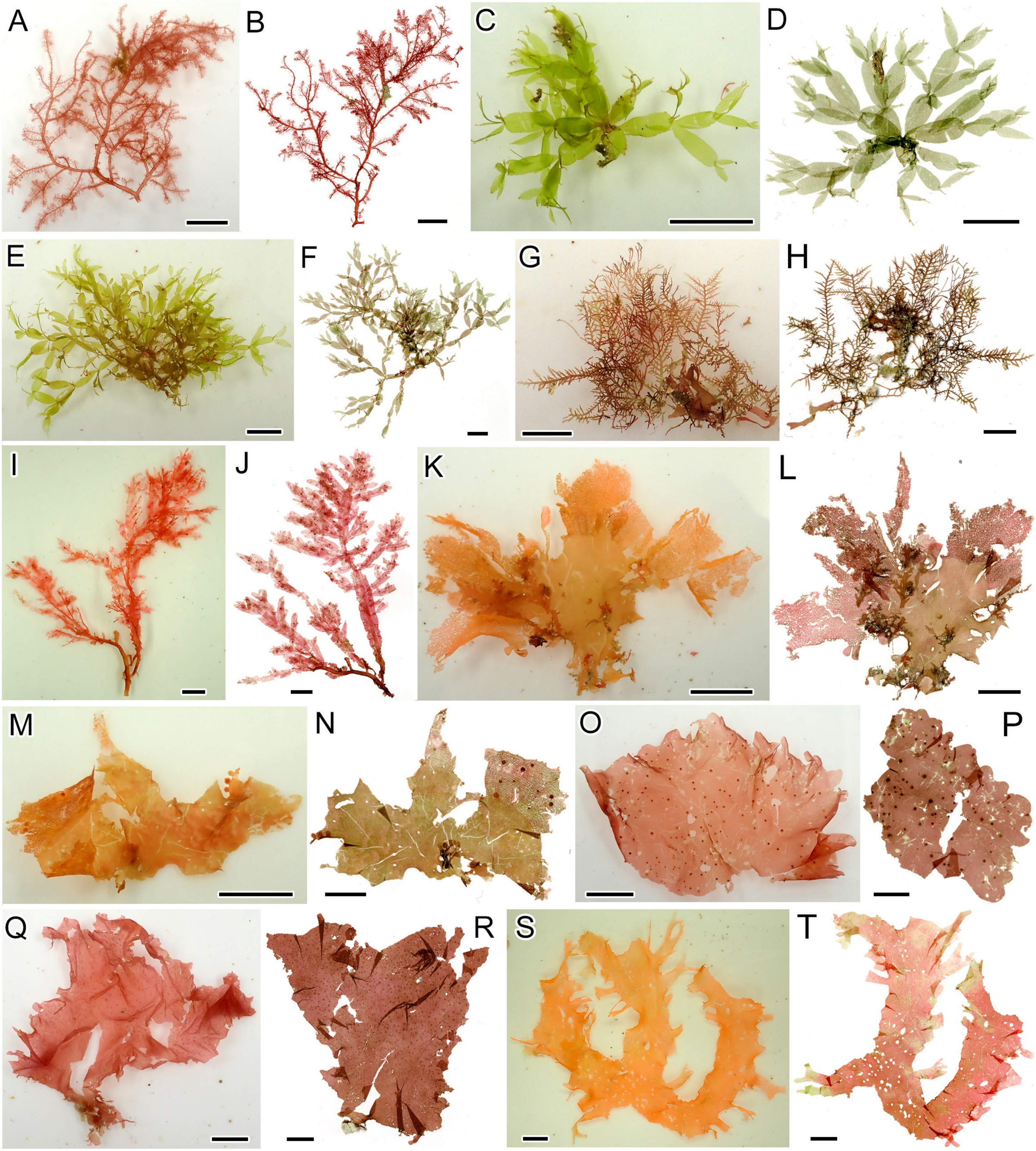

S2 Fig. Habits and herbarium specimens of red algae collected from offshore Tanegashima Island. (A, B) *Dasya* sp. TNE (TNS AL-222154). (C, D) Delesseriaceae sp.1 TNE (TNS AL-220666). (E, F) Delesseriaceae sp.2 TNE (TNS AL -220667). (G, H) “*Sorella*” *pulchra* (TNS AL-220665). (I, J) “*Hypoglossum*” *serratifolium* (TNS AL-215844). (K, L) *Martensia* sp.1 TNE (TNS AL-222060). (M, N) *Martensia* sp.2 TNE (TNS AL-222068). (O, P) Nitophylloideae sp.1 TNE (TNS AL-222145). (Q, R) Nitophylloideae sp.2 TNE (TNS AL-222090). (S, T) *Nitophyllum* sp. TNE (TNS AL-222074). Scale bar = 1 cm (A–F, I–N, Q–T), 5 mm (G, H, O, P).

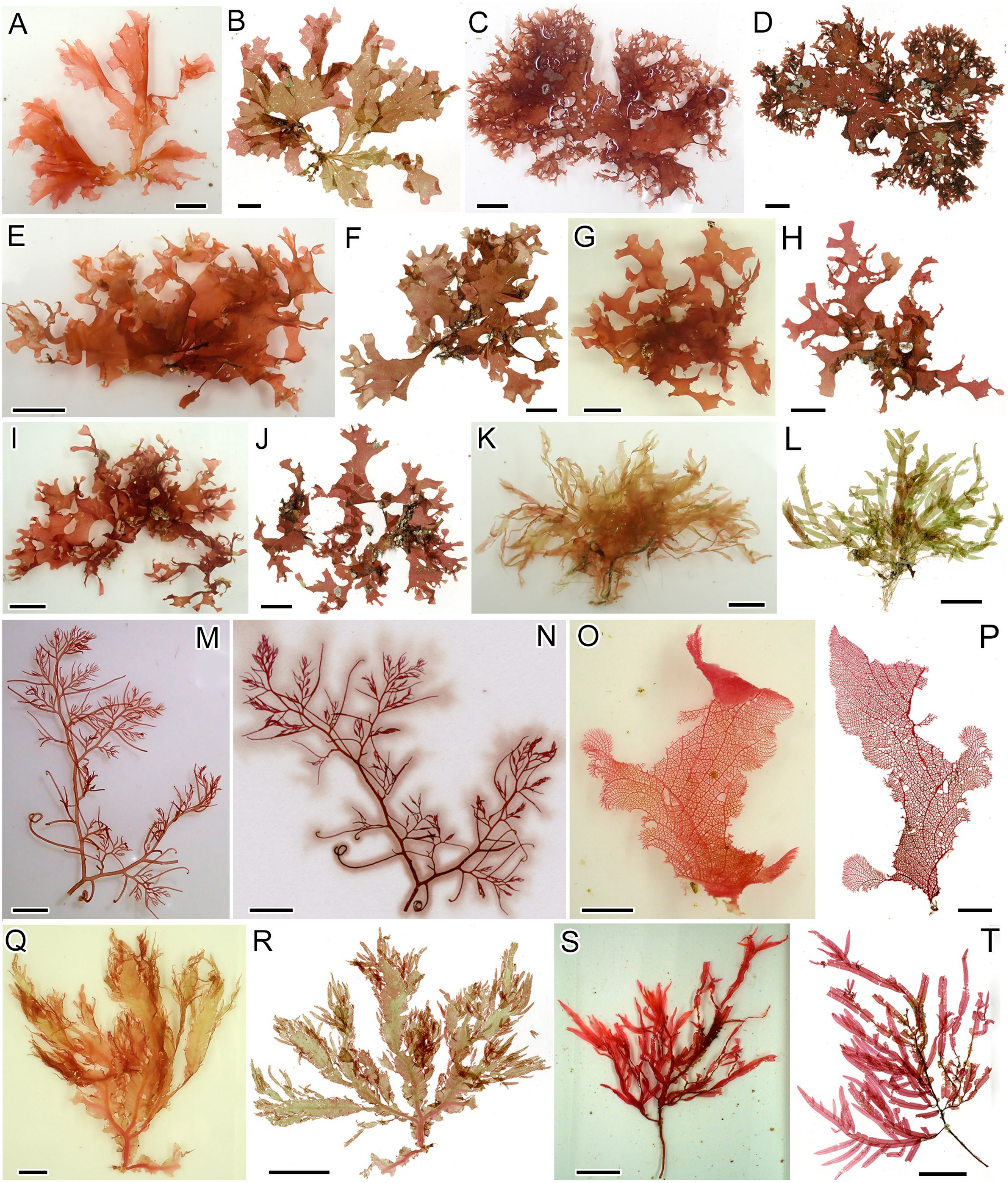

S3 Fig. Habits and herbarium specimens of red algae collected from offshore Tanegashima Island. (A, B) Phycodryoideae sp.1 TNE (TNS AL-222212). (C, D) Phycodryoideae sp.2 TNE (TNS AL-222110). (E, F) Phycodryoideae sp.3 TNE (TNS AL-220684). (G, H) Phycodryoideae sp.4 TNE (TNS AL-222160). (I, J) Phycodryoideae sp.5 TNE (TNS AL -222166). (K, L) "*Hypoglossum*" *nipponicum* (TNS AL-222181). (M, N) *Sympodothamnion leptophyllum* (TNS AL-200169). (O, P) "*Vanvoorstia*" *coccinea* (TNS AL-209889). (Q, R) *Yoshidaphycus ciliatus* (TNS AL-215852). (S, T) Zinovaeeae sp. TNE (TNS AL-220678). Scale bar = 1 cm (A–F, I–L, Q), 5 mm (G, H, M, N, O, P), 3 cm (R–T).

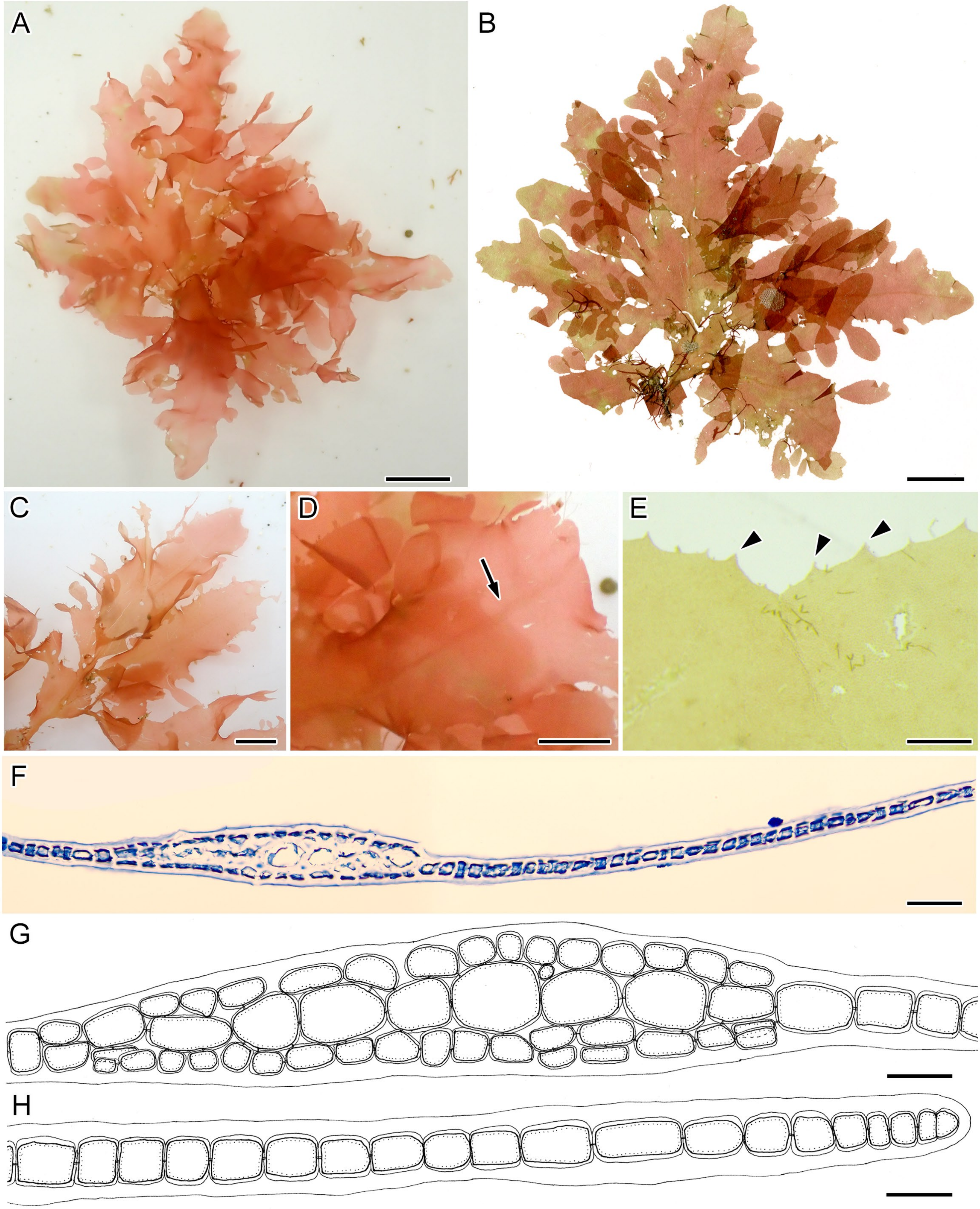

S4 Fig. *Pseudopolyneura hyacinthina* (TNS AL-222210) collected from offshore Tanegashima Island. (A) Habit. (B) Herbarium specimen. (C) Close-up of blades. (D) Close-up of blade showing midrib (arrow). (E) Magin of blade showing microscopic dentations (arrowheads). (F) Transverse section of blade. (G) Transverse section of midrib. (H) Transverse section of marginal part of blade. Scale bar = 1 cm (A, B), 500 μm (C–E), 100 μm (F–H).

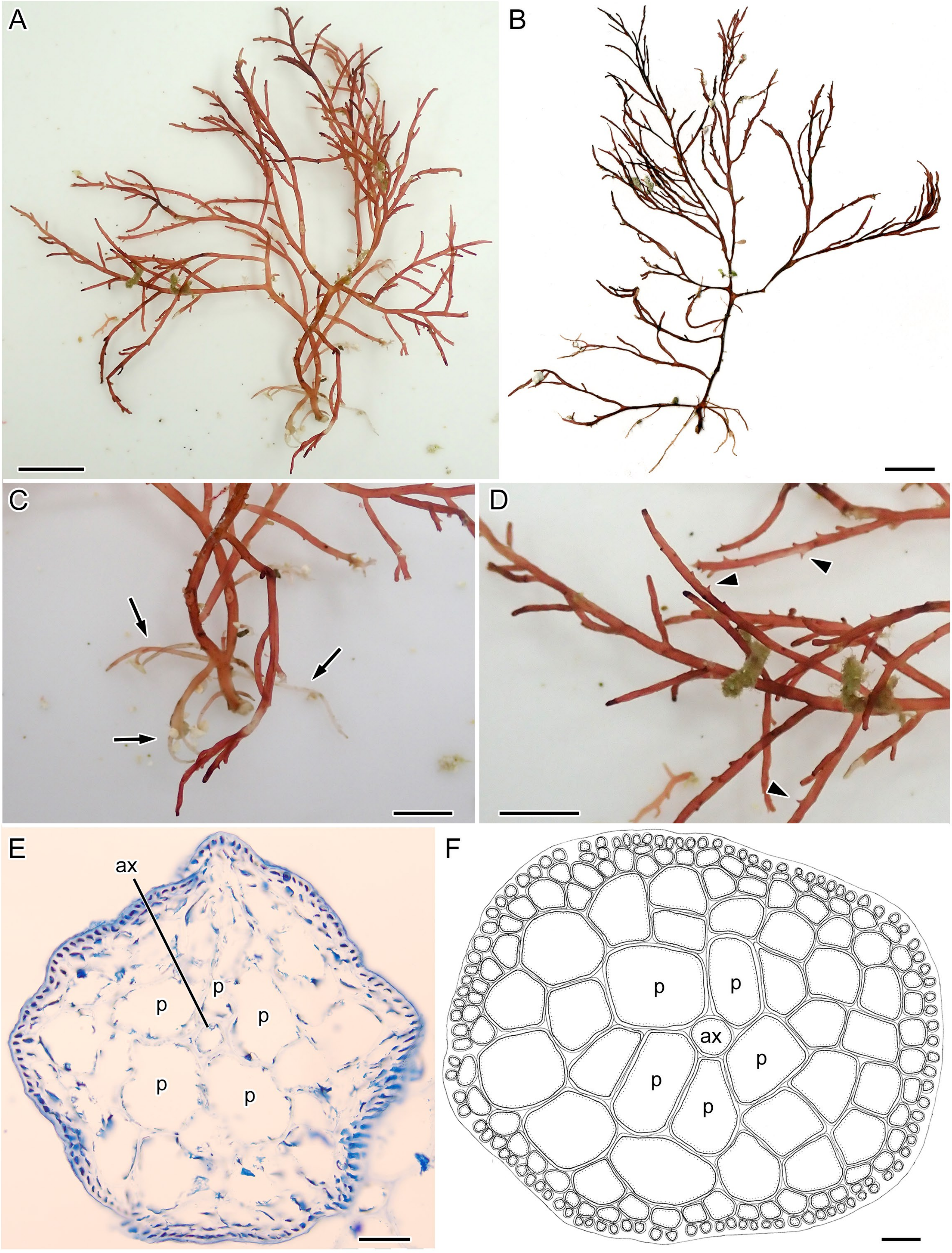

S5 Fig. *Acanthophora dendroides* (TNS AL-222201) collected from offshore Tanegashima Island. (A) Habit. (B) Herbarium specimen. (C) Close-up of the basal part of thallus showing stoloniferous axes (arrows) occurring near the holdfast. (D) Close-up of the upper part of thallus showing short spines (arrowheads). Note that lateral branches are not constricted at the base. (E, F) Transverse section of thallus showing axial cell (ax) surrounded by five periaxial cells (p). Scale bar = 1 cm (A, B), 5 mm (C, D), 100 μm (E, F).

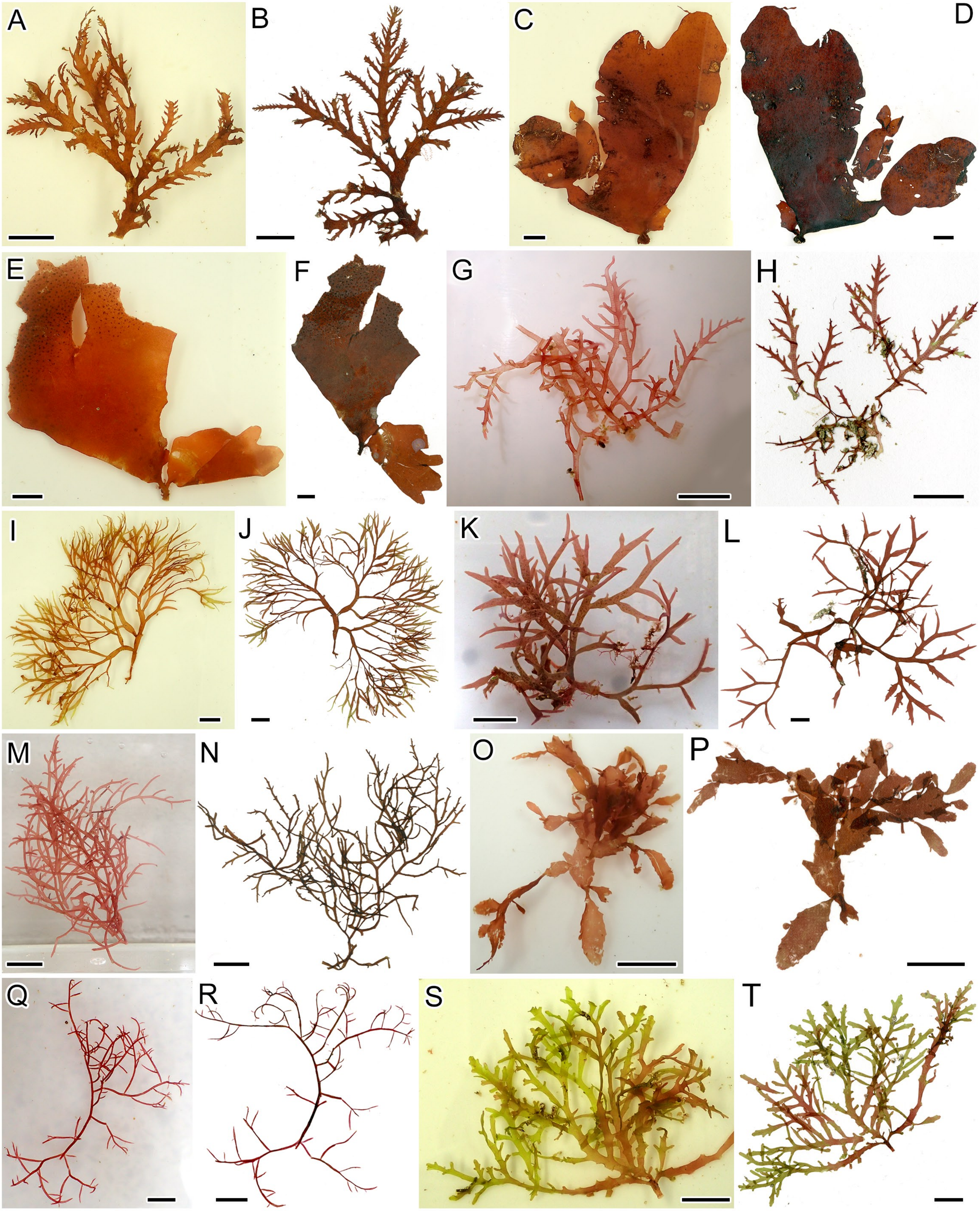

S6 Fig. Habits and herbarium specimens of red algae collected from offshore Tanegashima Island. (A, B) Amansieae sp. TNE (TNS AL-222201). (C, D) *Aneurianna lorentzii* (TNS AL-209898). (E, F) *Aneurianna* sp. TNE (TNS AL -220645). (G, H) *Chondria intertexta* (TNS AL-213817). (I, J) *Chondria mageshimensis* (TNS AL-209904). (K, L) *Chondria* sp.1 TNE (TNS AL-209903). (M, N) *Chondria* sp.2 TNE (TNS AL-220649). (O, P) *Chondria* sp.3 TNE (TNS AL-222062). (Q, R) *Chondria* sp.4 TNE (TNS AL-220650). (S, T) *Chondrophycus* sp.1 TNE (TNS AL-220652). Scale bar = 1 cm (A–J, M–T), 5 mm (K, L).

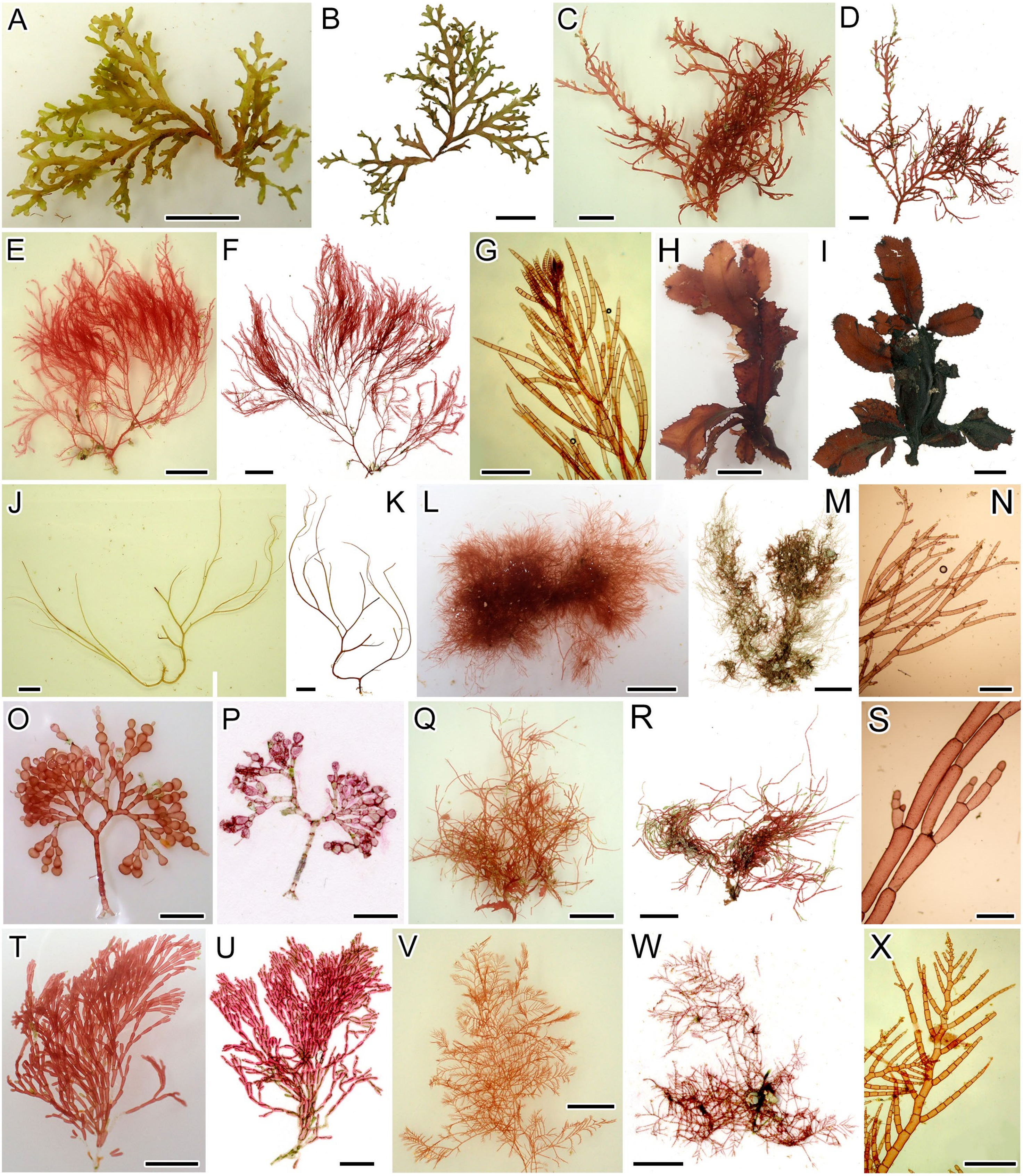

S7 Fig. Habits and herbarium specimens of red algae collected from offshore Tanegashima Island. (A, B) *Chondrophycus* sp.2 TNE (TNS AL-220653). (C, D) *Laurencia* sp. TNE (TNS AL-220657). (E–G) *Lophocladia japonica* (TNS AL-220660). (H, I) *Neurymenia nigricans* (TNS AL-215872). (J, K) *Wrightiella* sp. TNE (TNS AL-220662). (L–N) *Anotrichium* sp. TNE (TNS AL-220628). (O, P) *Griffithsia venusta* (TNS AL-200149). (Q–S) *Griffithsia* sp.1 TNE (*G*. cf. *subcylindrica*; TNS AL-222167). (T, U) *Griffithsia* sp.2 TNE (TNS AL-222108). (V–X) *Pleonosporium* sp. TNE (TNS AL -220689). Scale bar = 1 cm (A-F, H-M, Q, R), 5 mm (O, P, T–W), 500 µm (G, N, S, X).

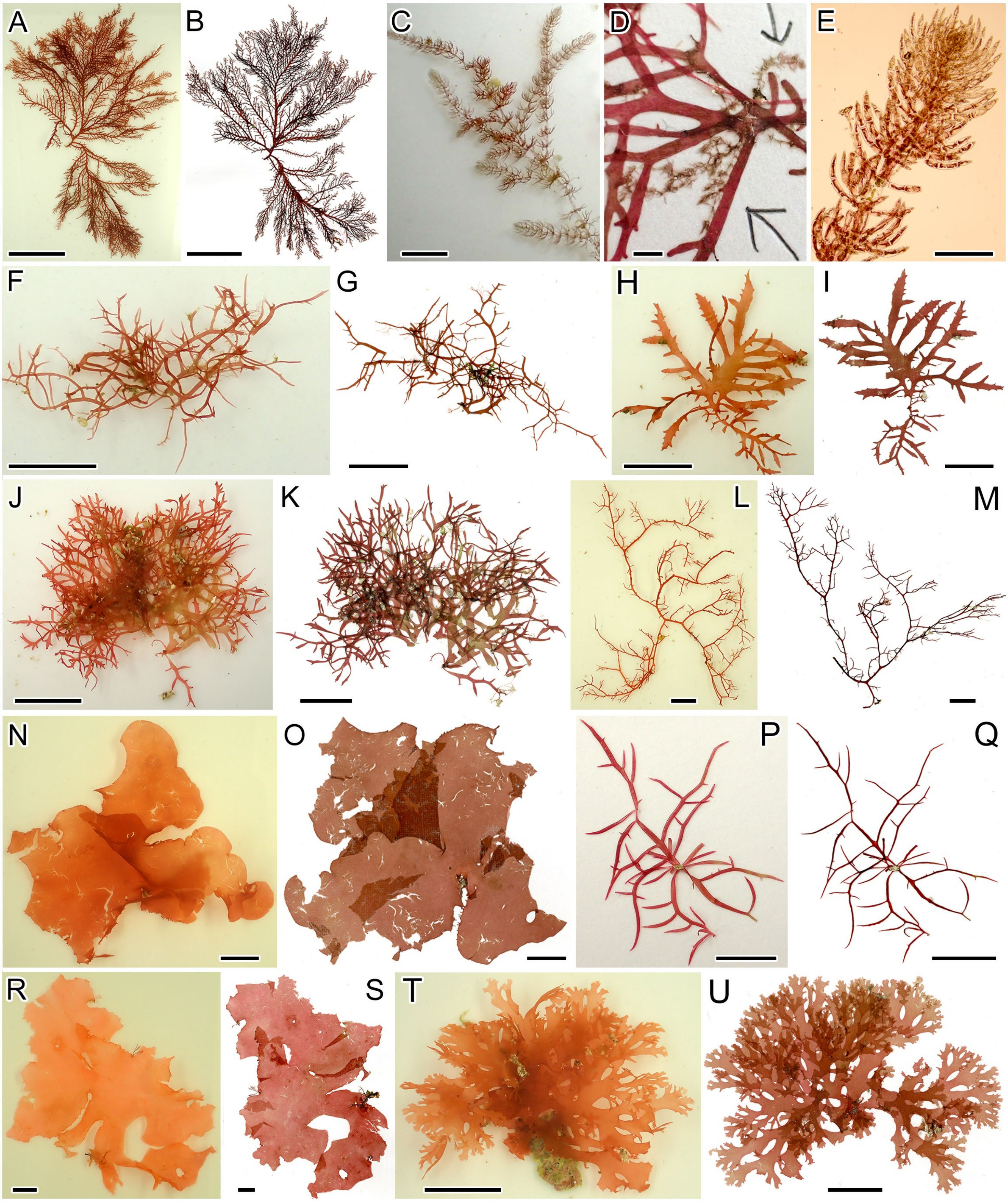

S8 Fig. Habits and herbarium specimens of red algae collected from offshore Tanegashima Island. (A, B) *Wrangelia tagoi* (TNS AL-222220). (C–E) *Wrangelia* sp. TNE (*W*. cf. *tanegata*; TNS AL-222176). (F, G) *Calliblepharis saidana* (TNS AL-220759). (H, I) *Calliblepharis* sp.1 TNE (TNS AL-220755). (J, K) *Calliblepharis* sp.2 TNE (TNS AL-220758). (L, M) *Hypnea yamadae* (TNS AL-209788). (N, O) *Halarachnion latissimum* (TNS AL-220760). (P, Q) *Chondracanthus saundersii* (TNS AL-209799). (R, S) *Austrokallymenia* sp.1 (*Kallymenia* cf. *sessilis*) (TNS AL-213790). (T, U) *Callophyllis* sp.1 TNE (*C*. cf. *adhaerens*) (TNS AL-209807). Scale bar = 5 cm (A, B), 1 cm (F–U), 2 mm (C, D), 500 μm (E).

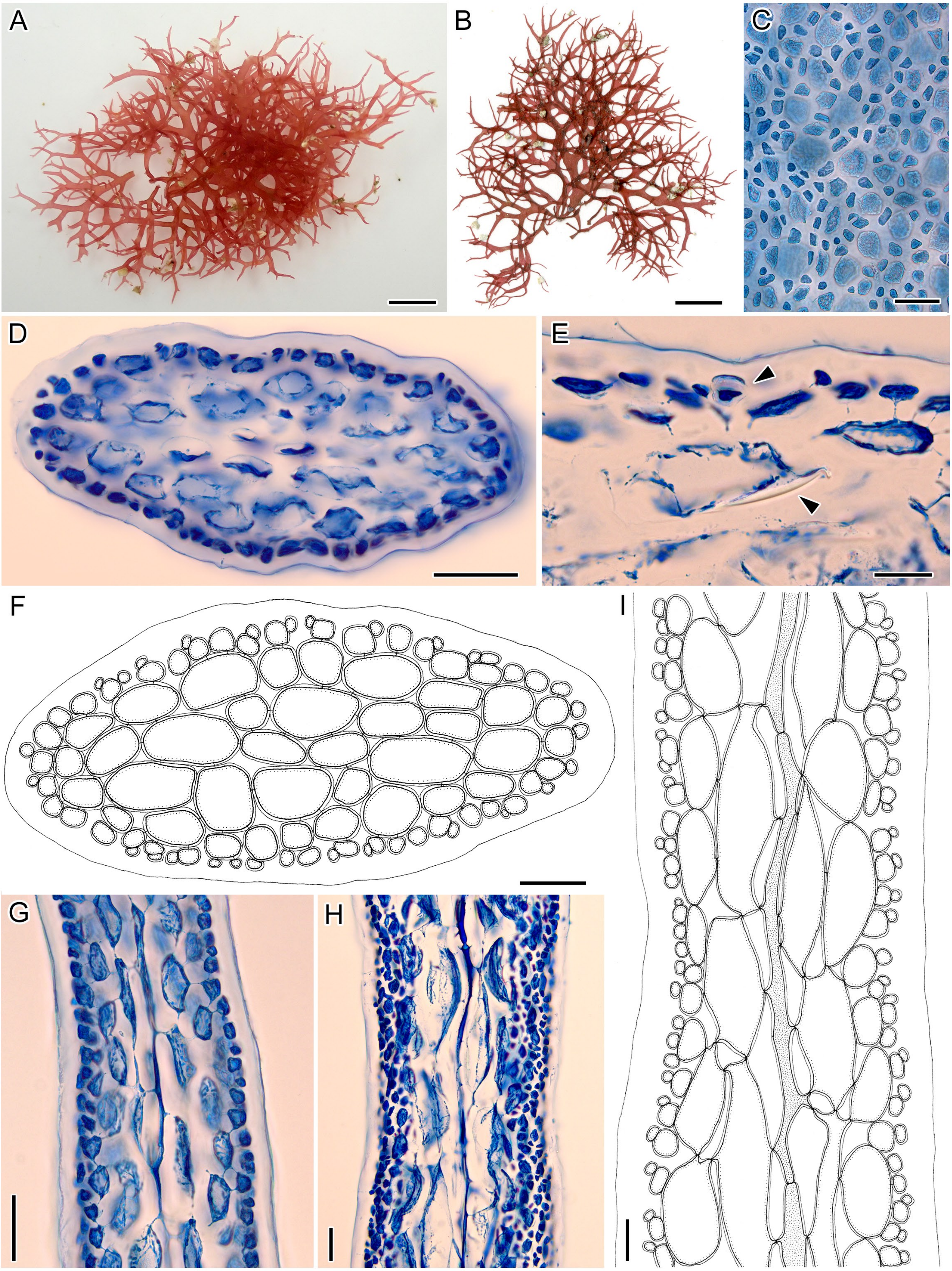

S9 Fig. *Calliblepharis yasutakei* (TNS AL-220757) collected from offshore Tanegashima Island. (A) Habit. (B) Herbarium specimen. (C) Surface view of blade. (D) Transverse section of upper part of thallus. (E) Close-up of Transverse section showing lenticular thickenings in the medullary and cortical cells (arrowheads). (F) Detail of transverse section of upper part of thallus. (G) Longitudinal section of upper part of thallus. (H) Longitudinal section of middle part of thallus. (I) Detail of longitudinal section of middle part of thallus. Scale bar = 5 mm (A, B), 50 μm (C–I).

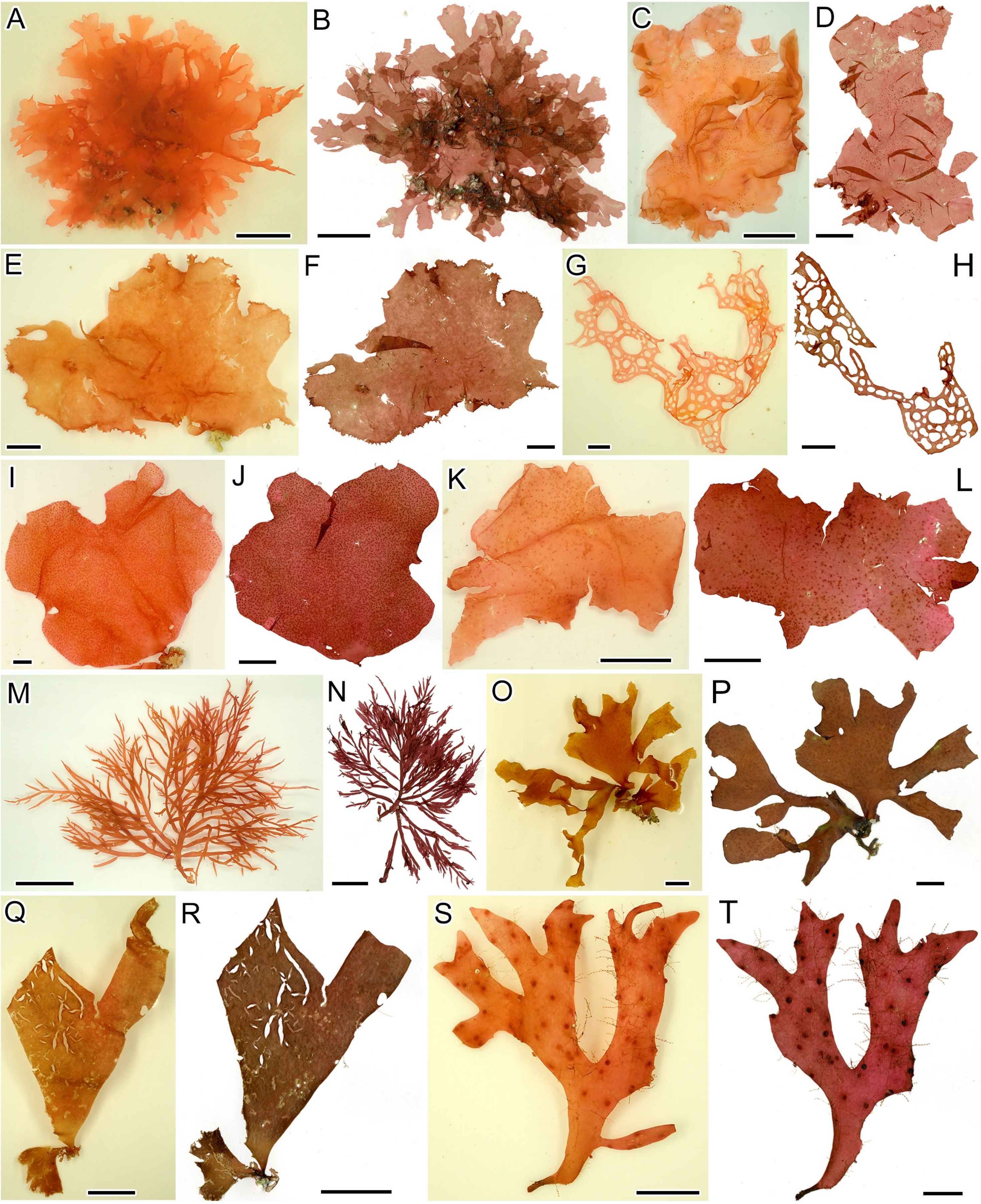

S10 Fig. Habits and herbarium specimens of red algae collected from offshore Tanegashima Island. (A, B) *Callophyllis* sp.2 TNE (TNS AL-222049). (C, D) *Croisettea* sp. TNE (TNS AL-222077). (E, F) Kallymeniaceae sp. TNE (TNS AL-220767). (G, H) "*Kallymenia*" *perfolata* (TNS AL-209813). (I, J) *Psaromenia* sp.1 (*Kallymenia* cf. *crassiuscula*) (TNS AL-209816). (K, L) *Psaromenia* sp.2 (*Kallymenia* cf. *crassiuscula*) (TNS AL-220762). (M, N) *Solieria pacifica* (TNS AL-215754). (O, P) *Gracilaria punctata* (TNS AL-209781). (Q, R) *Gracilaria sublittoralis* (TNS AL-220696). (S, T) *Gracilaria* sp.1 TNE (TNS AL-220692). Scale bar = 1 cm (A, B, E–I, S, T), 3 cm (C, D, J, K–N, O, R).

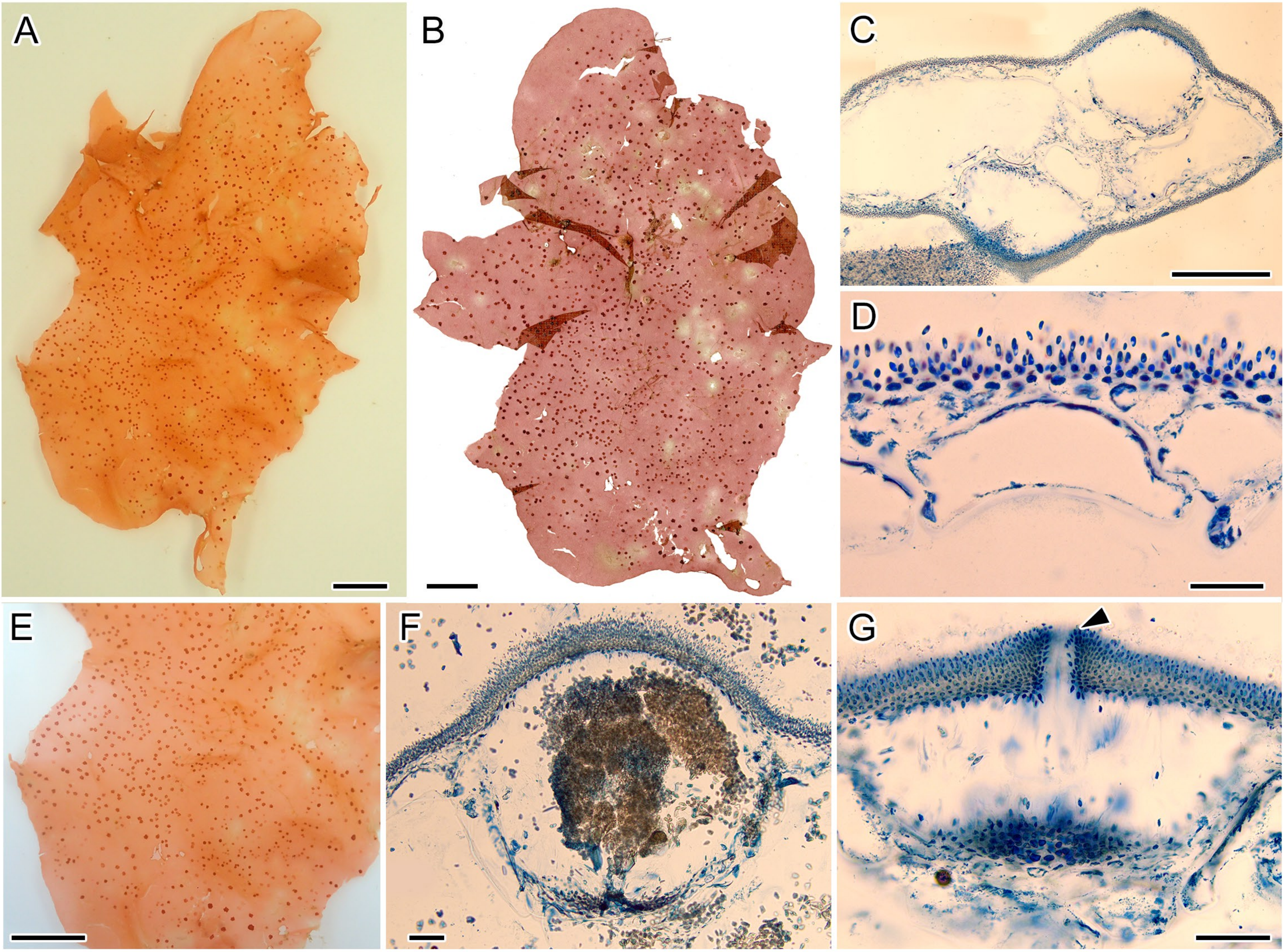

S11 Fig. *Croisettea kalaukapuae* (TNS AL-220766) collected from offshore Tanegashima Island. (A) Habit. (B) Herbarium specimen. (C) Cross section of blade showing elongated and ruptured medulla. (D) Close-up of cortex. (E) Close-up of blade showing cystocarps scattered on the blade. (F) Cross section of cystocarp. (G) Cross section of cystocarp showing an ostiole (arrowhead). Scale bar = 1 cm (A, B, E), 500 μm (C), 50 μm (D), 100 μm (F, G).

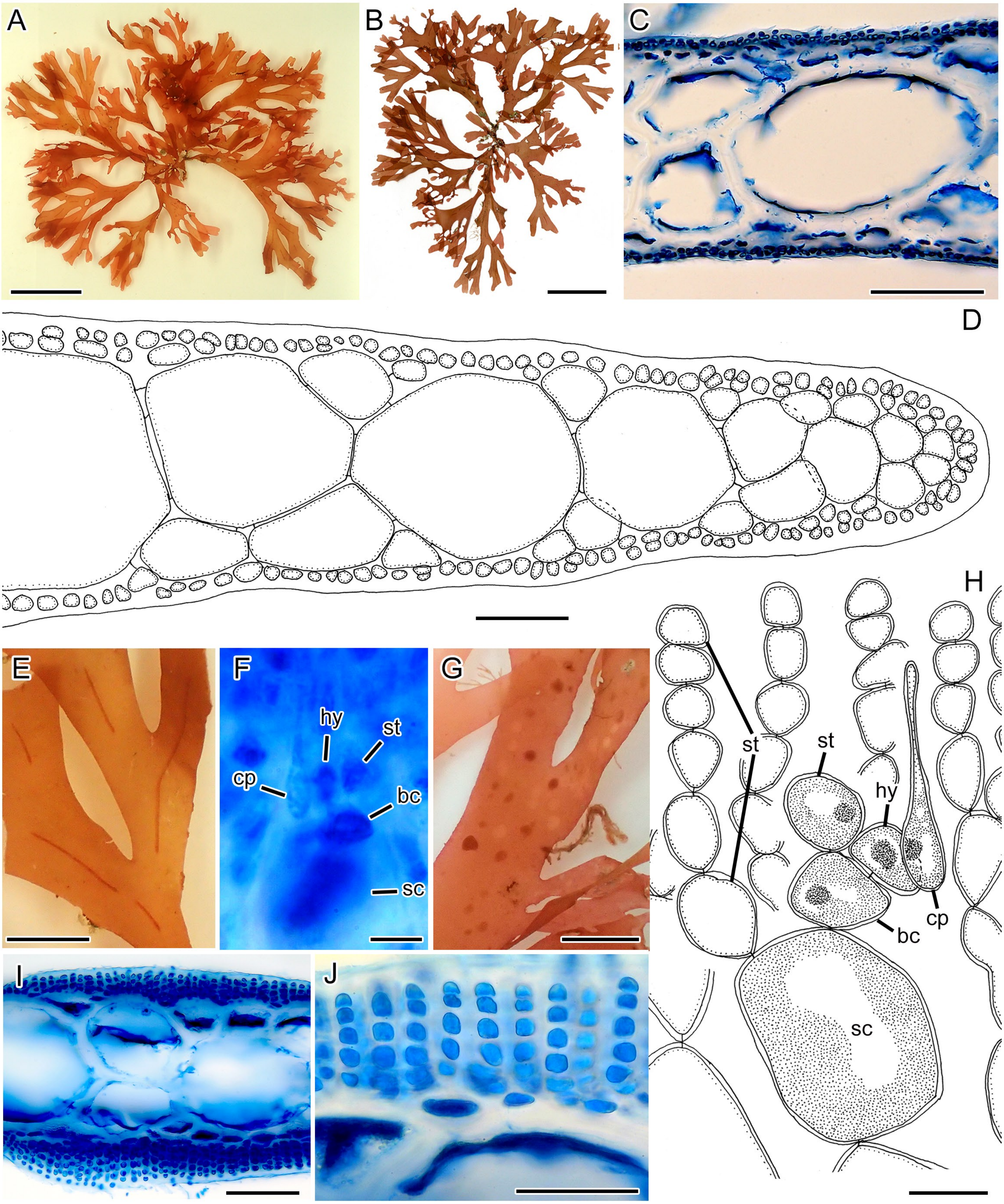

S12 Fig. *Stenogramma guleopoense* (TNS AL-209830: A–F, H; TNS AL-209832: G, I, J) collected from offshore Tanegashima Island. (A) Habit. (B) Herbarium specimen. (C, D) Transverse section of thallus. (E) Close-up of a blade showing the interrupted median thickened line. (F) Longitudinal view of a procarp showing a three-celled carpogonial branch with a sterile lateral from the basal cell borne on the supporting cell. bc, basal cell; cp, carpogonium; hy, hypogenous cell; sc, supporting cell; st, sterile cell. (G) Close-up of a blade showing tetrasporangial nemathecia. (H) Longitudinal view of a procarp showing a three-celled carpogonial branch with a sterile lateral from the basal cell borne on the supporting cell. A six-celled sterile branch borne on the supporting cell. (I) Transverse section of immature nemathecia. (J) Close-up of nemathecia showing rows of undivided tetrasporangia. Scale bar = 3 cm (A, B), 100 μm (C, I), 50 μm (D, J), 5 mm (E, G), 10 μm (F, H).

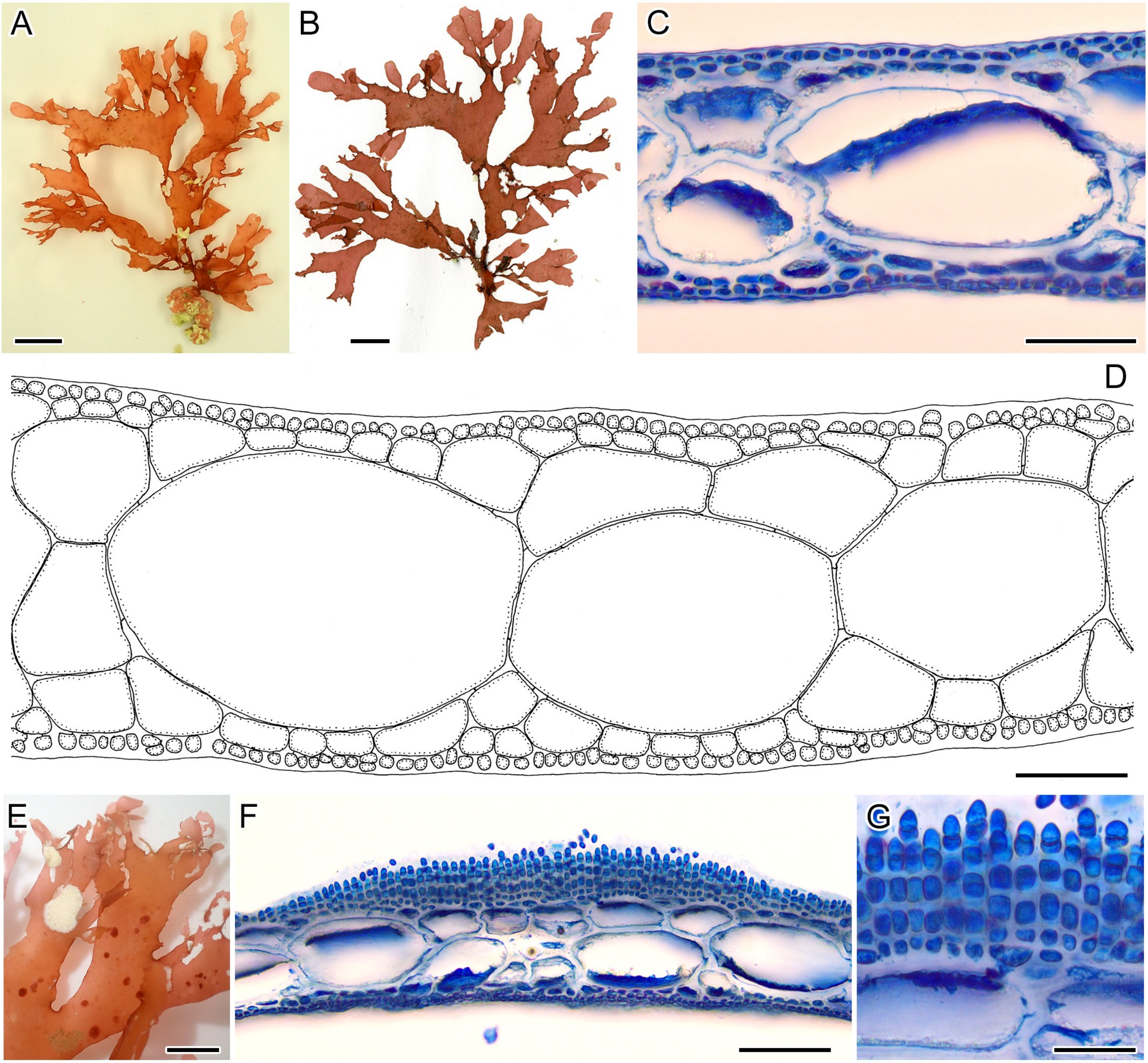

S13 Fig. *Stenogramma lamyi* (TNS AL-209842: A, B, D; TNS AL-222185: C, E–G) collected from offshore Tanegashima Island. (A) Habit. (B) Herbarium specimen. (C, D) Transverse section of thallus. (E) Close-up of a blade showing tetrasporangial nemathecia. (F) Transverse section of immature nemathecia. (G) Close-up of nemathecia showing rows of undivided tetrasporangia. Scale bar = 1 cm (A, B), 50 μm (C, D), 5 mm (E), 100 μm (F), 30 μm (G).

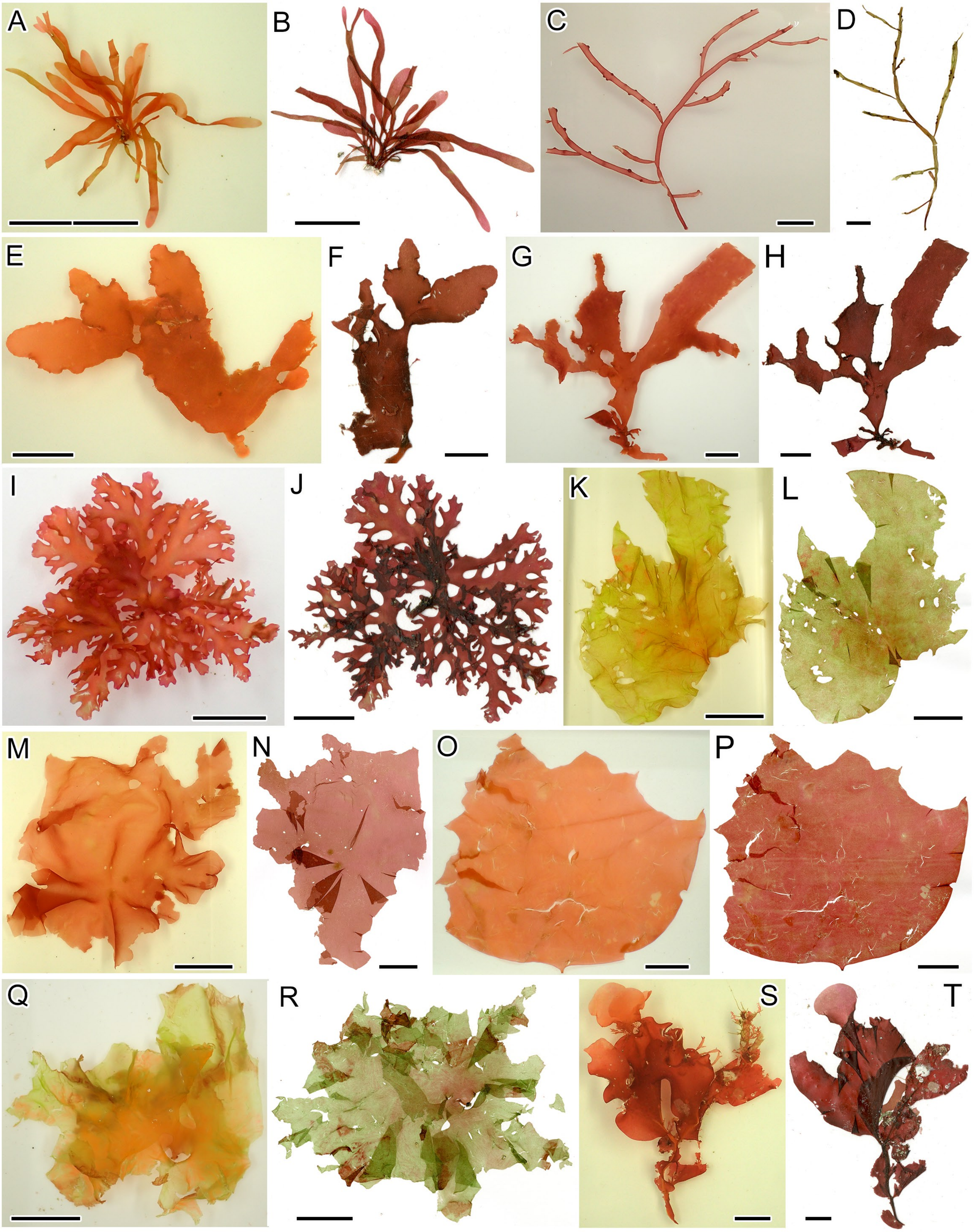

S14 Fig. Habits and herbarium specimens of red algae collected from offshore Tanegashima Island. (A, B) *Gracilaria* sp.3 TNE (TNS AL-222698). (C, D) *Gracilaria* sp.4 TNE (*G.* cf. *articulata*; TNS AL-209783). (E, F) *Gracilaria* sp.5 TNE (TNS AL-222050). (G, H) *Gracilariopsis mageshimensis* (TNS AL-215793). (I, J) *Yonagunia* sp. TNE (TNS AL -220700). (K, L) *Amalthea* sp.1 TNE (TNS AL-220705). (M, N) *Amalthea* sp.2 TNE (TNS AL-220711). (O, P) *Amalthea* sp.3 TNE (TNS AL-220724). (Q, R) *Amalthea* sp.4 TNE (TNS AL-222081). (S, T) *Cryptonemia semiprocumbens* (TNS AL -213356). Scale bar = 1 cm (A–J, S, T), 3 cm (M–R), 5 cm (K, L).

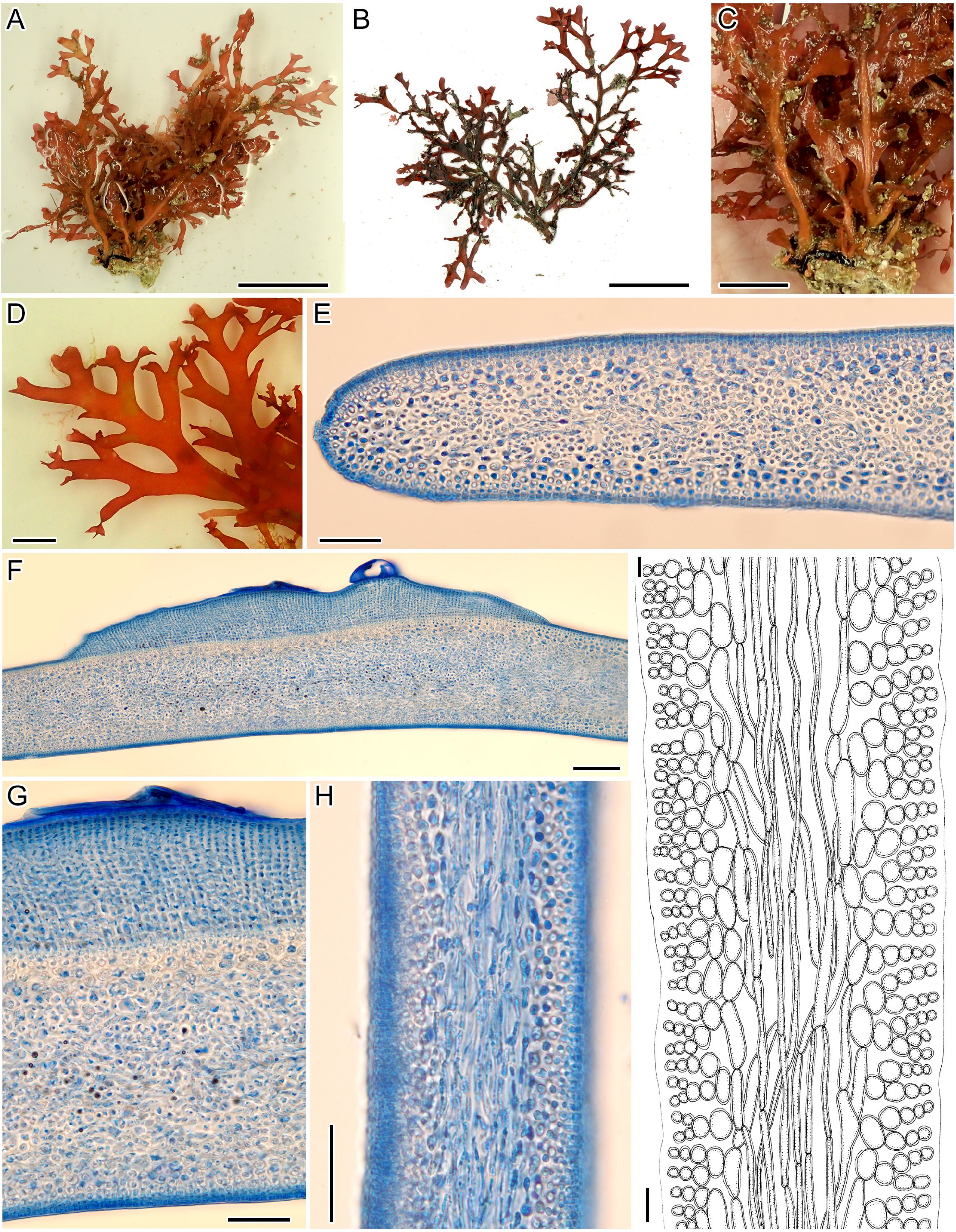

S15 Fig. *Yonagunia taiwani-borealis* (TNS AL-214473: A–C, E–I; TNS AL-214474: D) collected from offshore Tanegashima Island. (A) Habit . (B) Herbarium specimen. (C) Close-up of the middle to basal part of thallus. (D) Close-up of the upper part of thallus. (E) Transverse section of marginal part of blade. (F, G) Transverse section of middle part of blade. (H, I) Longitudinal section of young blade. Scale bar = 3 cm (A, B), 1 cm (C), 5 mm (D), 50 μm (E, G), 100 μm (F), 10 μm (I).

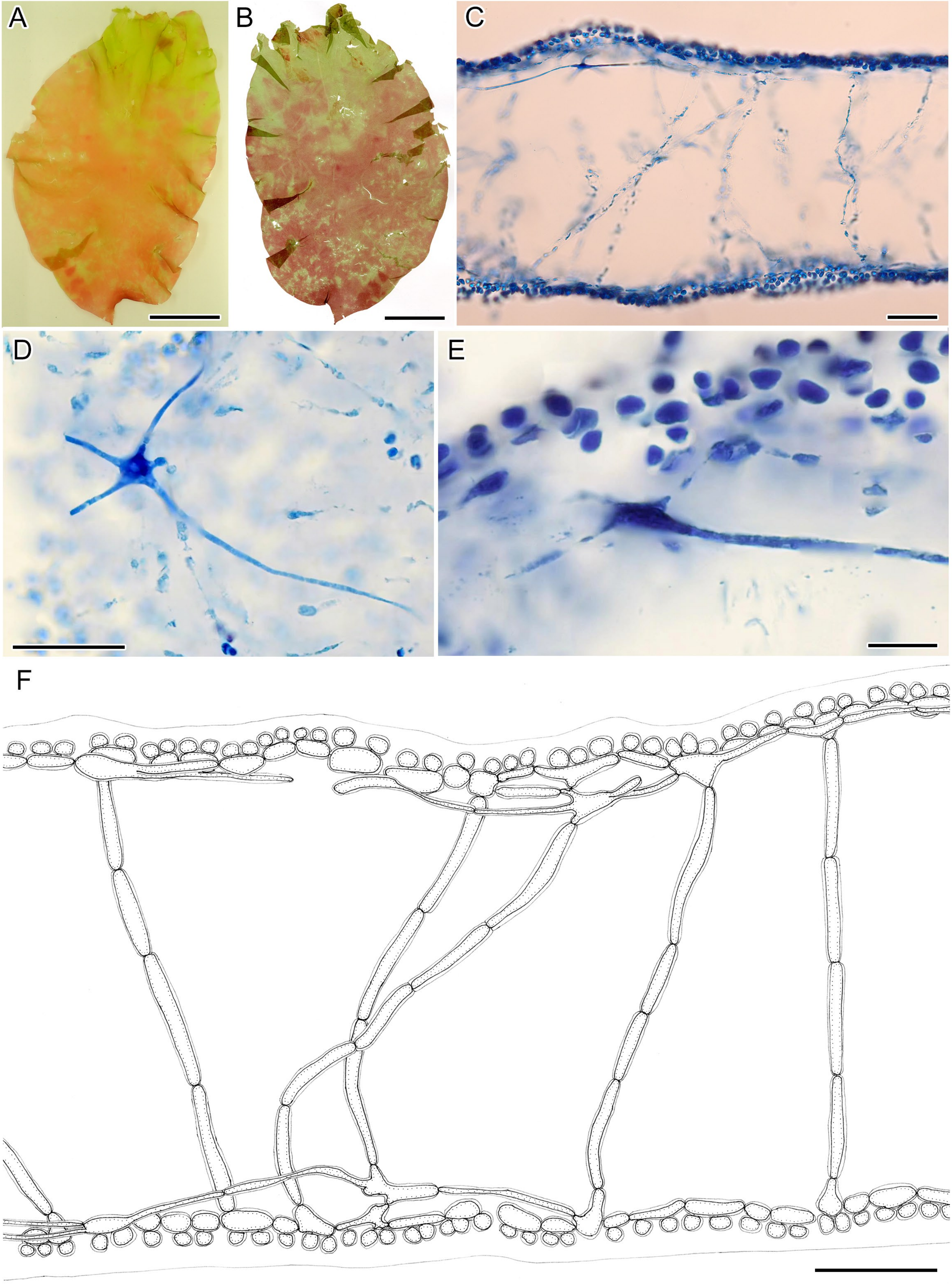

S16 Fig. *Amalthea rubida* (TNS AL-220704) collected from offshore Tanegashima Island. (A) Habit. (B) Herbarium specimen. (C) Transverse section of thallus. (D) Surface view of medullary stellate cell. (E) Transverse view of medullary stellate cell. (F) Transverse section of the upper part of thallus. Scale bar = 5 cm (A, B), 50 μm (C, D, F), 10 μm (E).

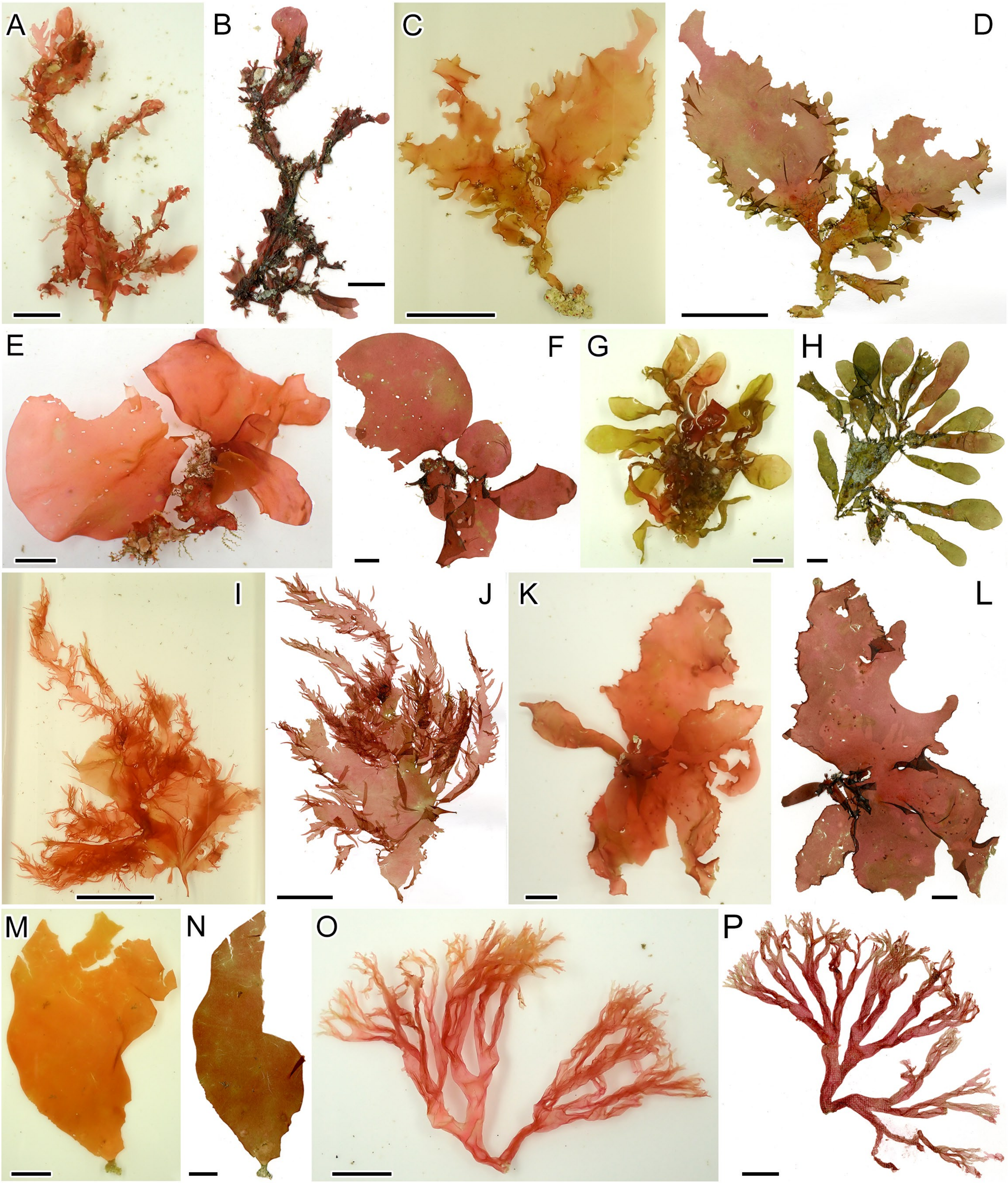

S17 Fig. Habits and herbarium specimens of red algae collected from offshore Tanegashima Island. (A, B) *Cryptonemia* sp. TNE (TNS AL-215796). (C, D) *Galene* sp.1 TNE (TNS AL-214462). (E, F) *Galene* sp.2 TNE (TNS AL -220729). (G, H) *Galene* sp.3 TNE (TNS AL-222143). (I, J) *Halymenia* sp. TNE (*H.* cf. *durvillei*; TNS AL-215802). (K, L) Halymeniaceae sp.1 TNE (TNS AL-222205). (M, N) Halymeniaceae sp.2 TNE (TNS AL-222124). (O, P) Halymeniaceae sp.3 TNE (TNS AL-220737). Scale bar = 1 cm (A, B, E–H, K–P), 5 cm (C, D, I, J).

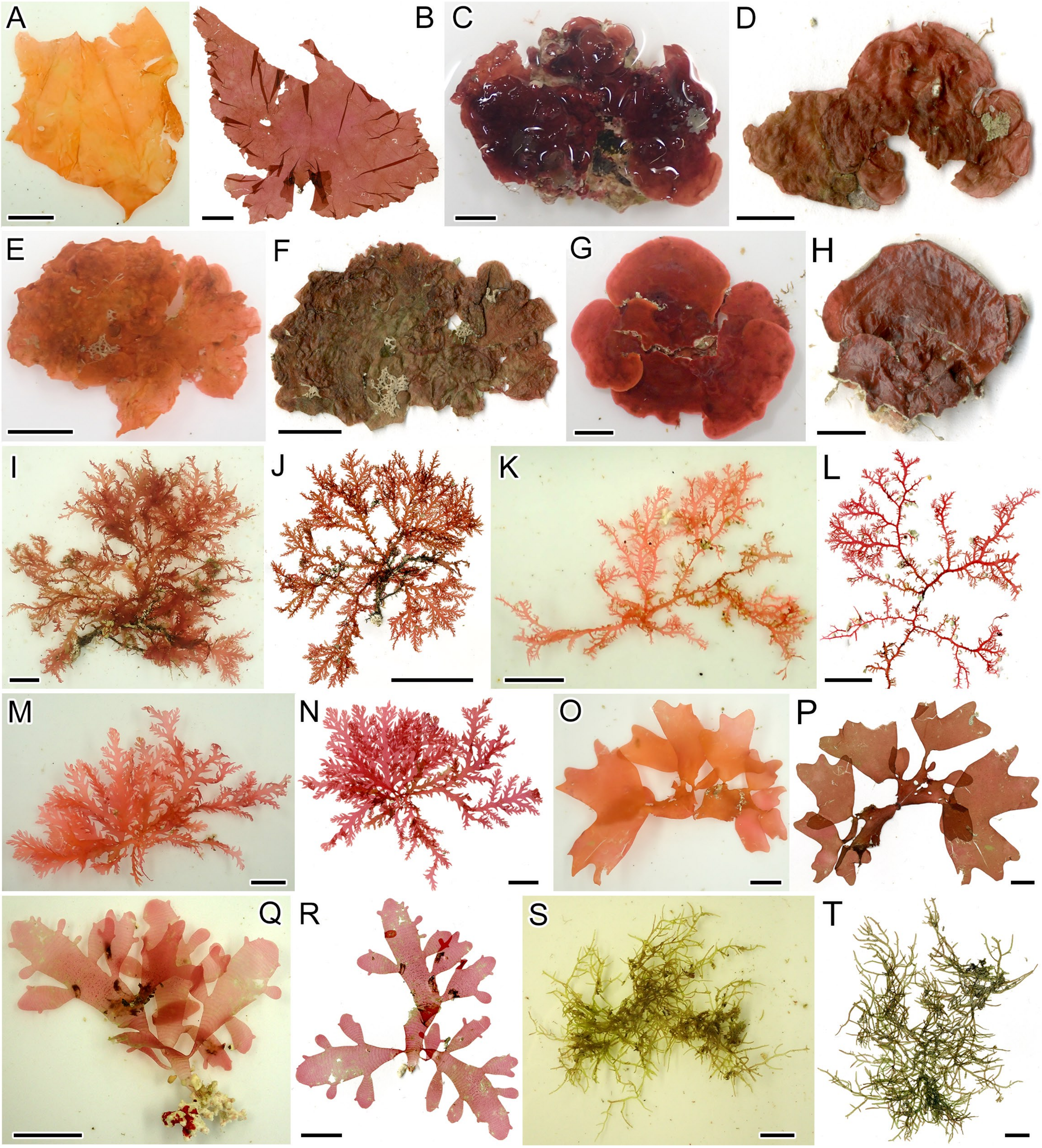

S18 Fig. Habits and herbarium specimens of red algae collected from offshore Tanegashima Island. (A, B) *Platoma* sp. TNE (TNS AL-220735). (C, D) *Agissea* sp.1 TNE (TNS AL-222096). (E, F) *Agissea* sp.2 TNE (*A.* cf. *orientalis*; TNS AL -222187). (G, H) *Incendia* sp. TNE (TNS AL-222186). (I, J) *Plocamium luculentum* (TNS AL-222170). (K, L) *Plocamium ovicorne* (TNS AL-215771). (M, N) *Plocamium* sp. TNE (TNS AL-222095). (O, P) *Sarcodia* sp. JP1 (TNS AL-222148). (Q, R) *Champia expansa* (TNS AL-209851). (S, T) *Champia* sp.1 TNE (TNS AL-220739). Scale bar = 1 cm (A, B, E, F, I, K–T), 5 mm (C, D, G, H), 5 cm (J).

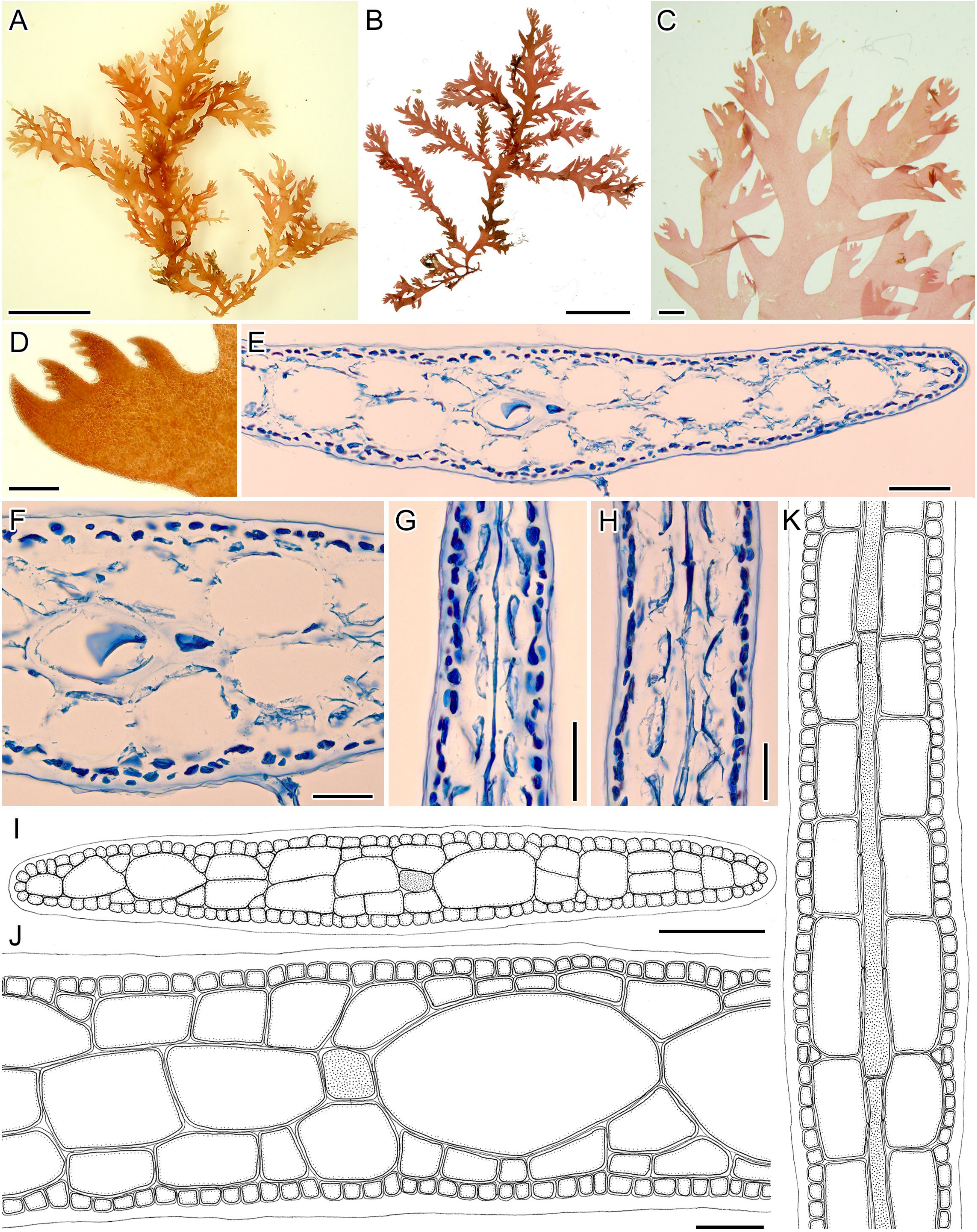

S19 Fig. *Plocamium brasiliense* (TNS AL-209776: A, B, D; TNS AL-215770: C, E–K) collected from offshore Tanegashima Island. (A) Habit. (B) Herbarium specimen. (C) Close-up of upper part of thalus. (D) Close-up of ramuli. (E, F) Transverse section of thallus. (G) Longitudinal section of upper part of thallus. (H) Longitudinal section of middle part of thallus. (I, J) Detail of transverse section of thallus. (K) Detail of longitudinal section of thallus. Scale bar = 1 cm (A, B), 1 mm (C), 100 μm (D, E, I), 50 μm (F–H, J, K).

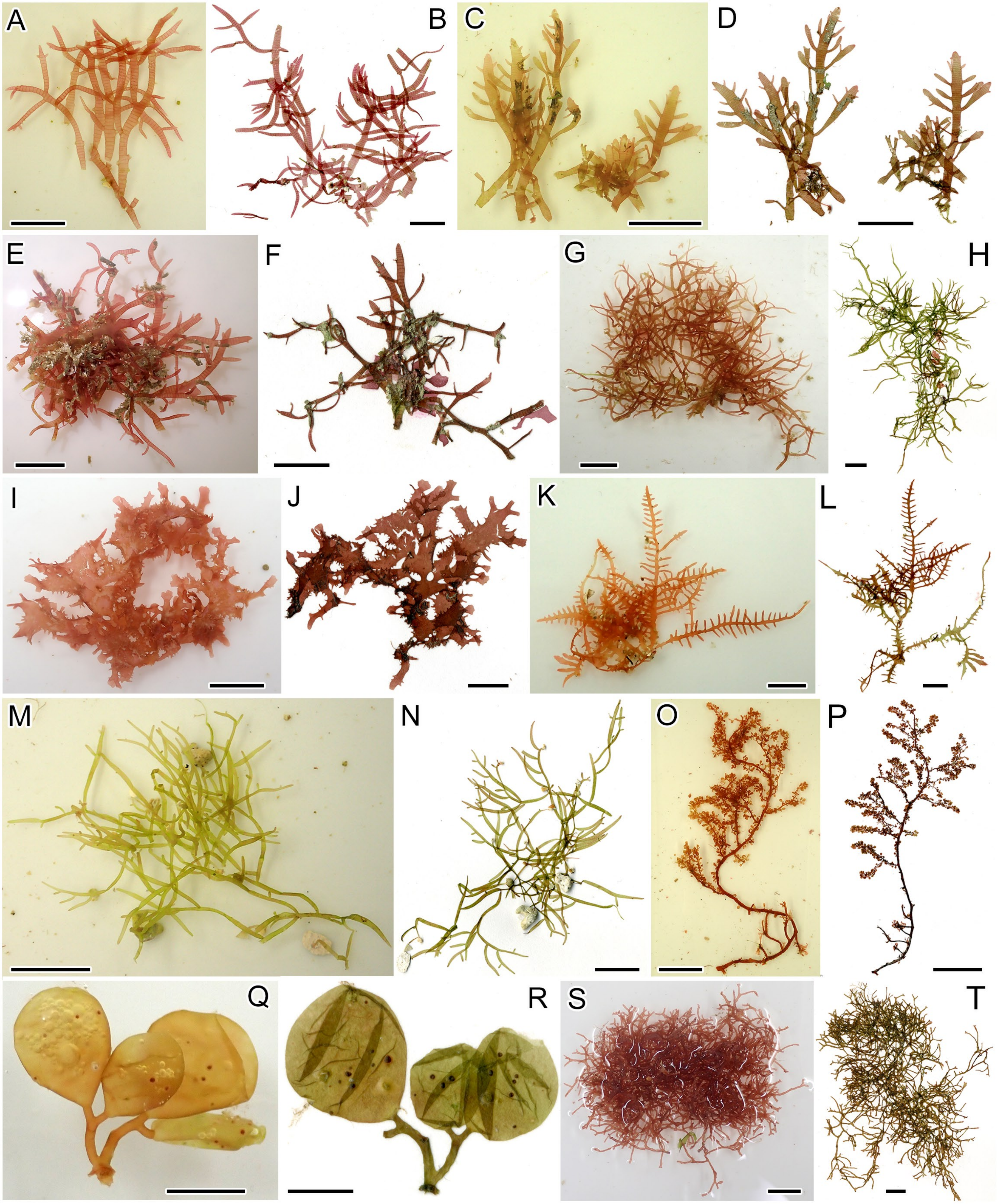

S20 Fig. Habits and herbarium specimens of red algae collected from offshore Tanegashima Island. (A, B) *Champia* sp.2 TNE (TNS AL-220743). (C, D) *Champia* sp.3 TNE (*C.* cf. *vieillardii*; TNS AL-220745). (E, F) *Champia* sp.4 TNE (TNS AL-220746). (G, H) *Champia* sp.5 TNE (TNS AL-222066). (I, J) *Gloiocladia* sp.1 TNE (TNS AL-222213). (K, L) *Gloiocladia* sp.2 TNE (TNS AL-215771). (M, N) Lomentariaceae sp. TNE (TNS AL-220748). (O, P) *Botryocladia leptopoda* (TNS AL-209858). (Q, R) *Botryocladia* sp. TNE (*B.* cf. *kuckuckii*; TNS AL-222137). (S, T) "*Chamaebotrys*" *lomentariae* (TNS AL-222103). Scale bar = 1 cm (A–J, M, N, S, T), 5 mm (K, L, Q, R), 3 cm (O), 5 cm (P).

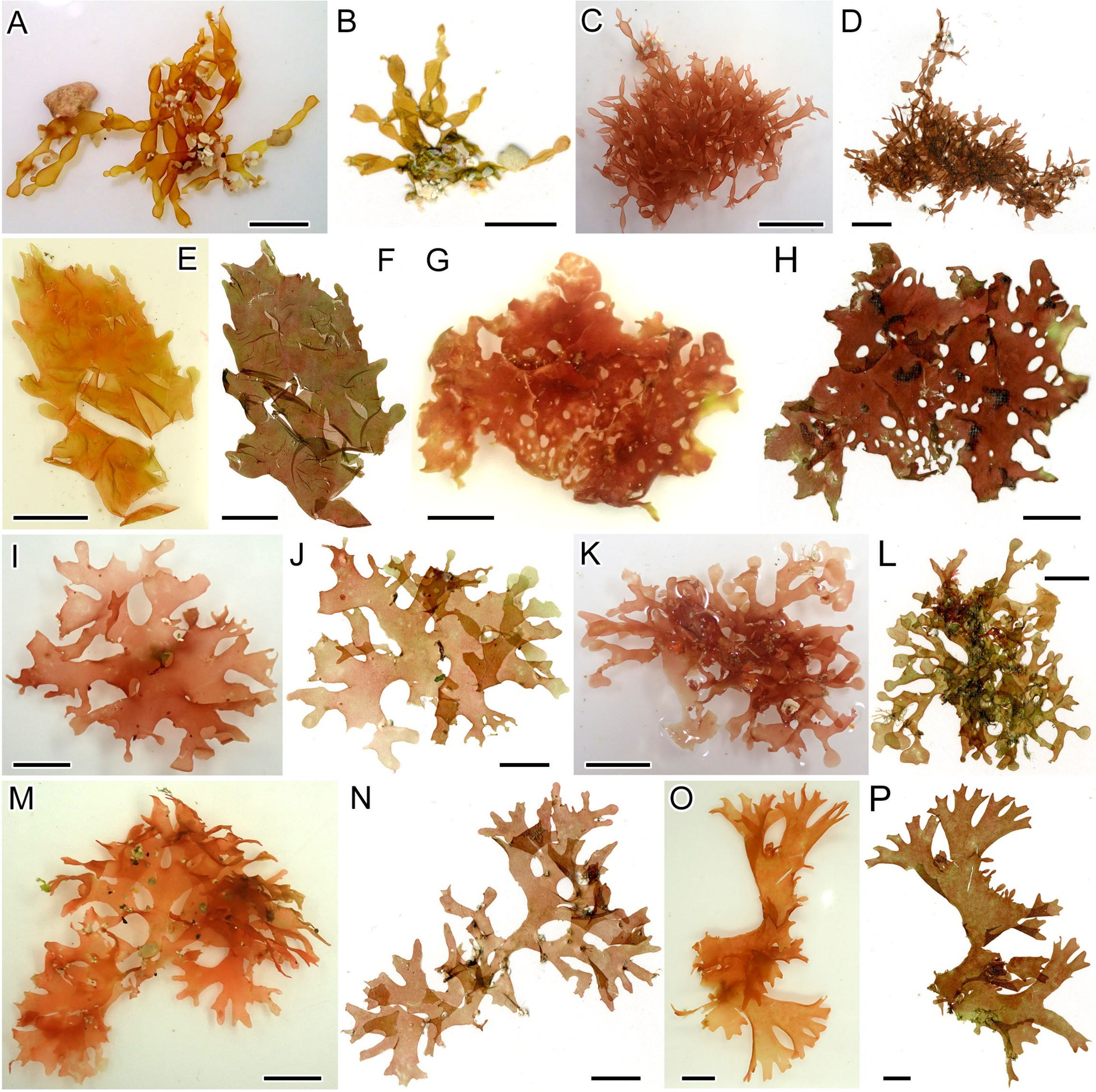

S21 Fig. Habits and herbarium specimens of red algae collected from offshore Tanegashima Island. (A, B) *Chamaebotrys* sp.1 TNE (*C.* cf. *boergesenii*; TNS AL-209864). (C, D) *Chamaebotrys* sp.2 TNE (*C.* cf. *boergesenii*; TNS AL-209865). (E, F) *Chrysymenia* sp. TNE (TNS AL-213811). (G, H) *Drouetia* sp. TNE (TNS AL-222162). (I, J) *Halichrysis* sp. TNE (TNS AL-222164). (K, L) *Halopeltis tanakae* (TNS AL-222105). (M, N) *Halopeltis* sp.1 TNE (TNS AL-222194). (O, P) *Halopeltis* sp.2 TNE (*H.* cf. *adnata*; TNS AL-222193). Scale bar = 5 mm (A, B), 1 cm (C, D, G–P), 3 cm (E, F).

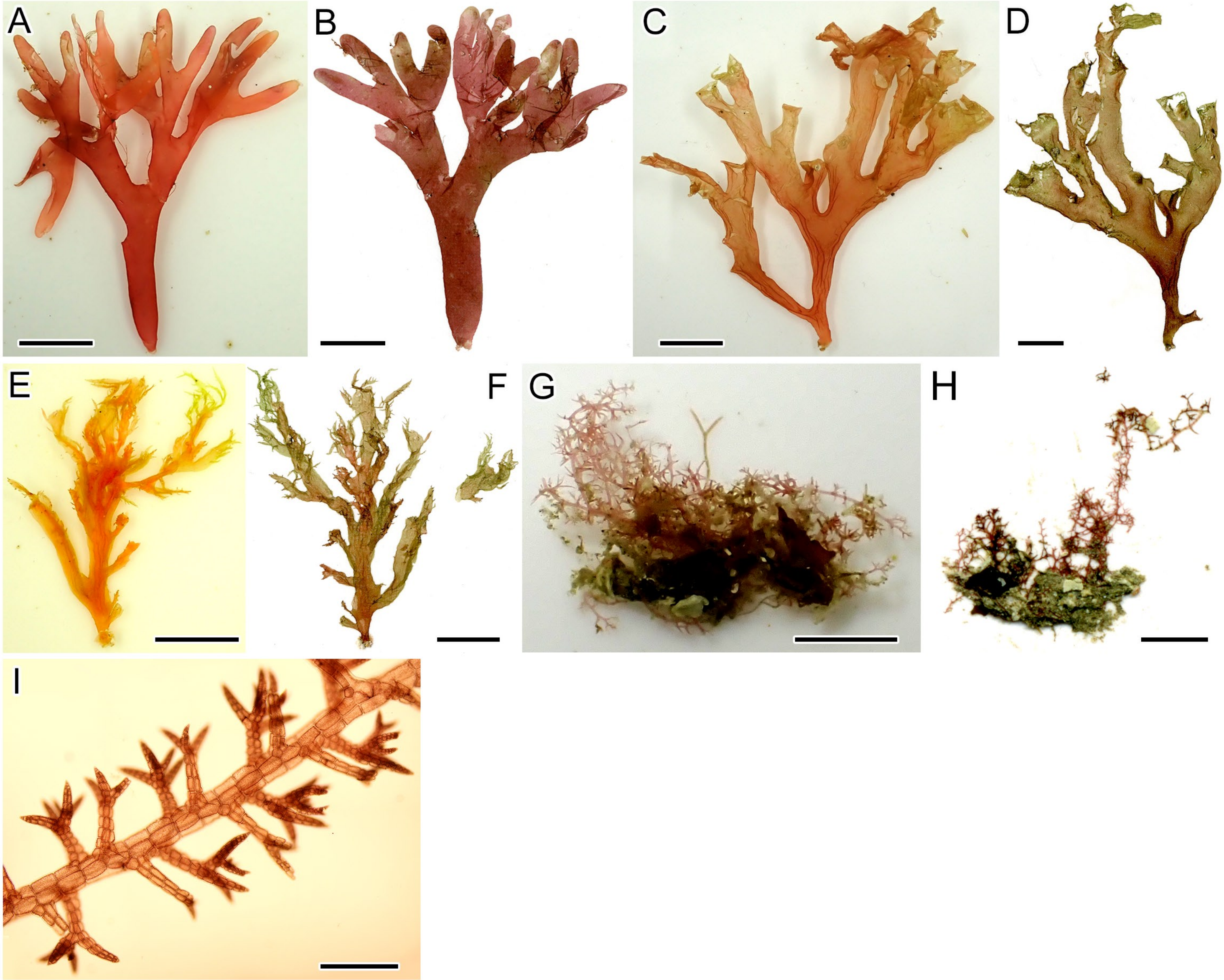

S22 Fig. Habits and herbarium specimens of red algae collected from offshore Tanegashima Island. (A, B) *Sebdenia* sp. TNE (*S*. cf. *flabellata*; TNS AL-215817). (C, D) Sebdeniaceae sp. TNE (TNS AL-222135). (E, F) *Schmitzia* sp. TNE (TNS AL-220738). (G–I) *Tolypiocladia* sp. TNE (TNS AL-213823). Scale bar = 1 cm (A–F), 3 mm (G, H), 500 µm (I).
