# Supplementary material for: DNA-based floristic survey of red algae (Rhodophyta) growing in the mesophotic coral ecosystems (MCEs) offshore of Tanegashima Island, northern Ryukyu Archipelago, Japan
Source: PLoS One. 2025 Mar 10;20(3):e0316067. doi: 10.1371/journal.pone.0316067 (PMC11893125; doi:10.1371/journal.pone.0316067)
Supplement: S5 File — Maximum likelihood phylogeny of red algae collected from offshore Tanegashima Island. (ZIP) [file pone.0316067.s005.zip › S5_File/S29_Fig.pdf]

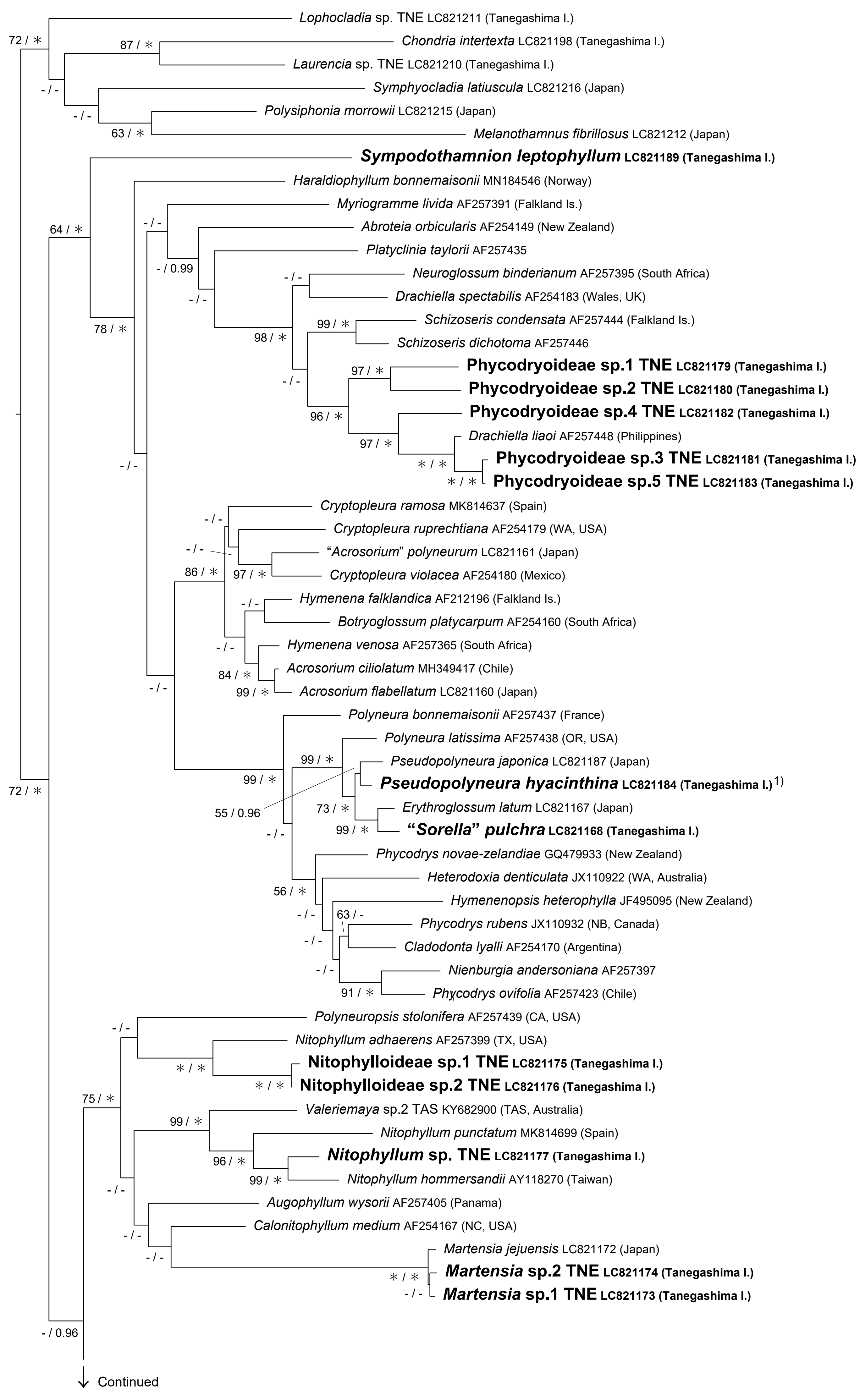

Lophocladia sp. TNE LC821211 (Tanegashima I.)
72 / *
87 / *
Chondria intertexta LC821198 (Tanegashima I.)
Laurencia sp. TNE LC821210 (Tanegashima I.)
- / -
Symphyocladia latiuscula LC821216 (Japan)
- / -
Polysiphonia morrowii LC821215 (Japan)
63 / *
Melanothamnus fibrillosus LC821212 (Japan)
Sympodothamnion leptophyllum LC821189 (Tanegashima I.)
Haraldiophyllum bonnemaisonii MN184546 (Norway)
Myriogramme livida AF257391 (Falkland Is.)
- / -
Abroteia orbicularis AF254149 (New Zealand)
64 / *
Platyclinia taylorii AF257435
- / 0.99
- / -
Neuroglossum binderianum AF257395 (South Africa)
- / -
Drachiella spectabilis AF254183 (Wales, UK)
78 / *
99 / *
Schizoseris condensata AF257444 (Falkland Is.)
98 / *
Schizoseris dichotoma AF257446
97 / *
Phycodryoideae sp.1 TNE LC821179 (Tanegashima I.)
- / -
Phycodryoideae sp.2 TNE LC821180 (Tanegashima I.)
96 / *
Phycodryoideae sp.4 TNE LC821182 (Tanegashima I.)
Drachiella liaoi AF257448 (Philippines)
97 / *
* / *
Phycodryoideae sp.3 TNE LC821181 (Tanegashima I.)
- / -
* / *
Phycodryoideae sp.5 TNE LC821183 (Tanegashima I.)
Cryptopleura ramosa MK814637 (Spain)
- / -
Cryptopleura ruprechtiana AF254179 (WA, USA)
- / -
"Acrosorium" polyneurum LC821161 (Japan)
86 / *
97 / *
Cryptopleura violacea AF254180 (Mexico)
- / -
Hymenena falklandica AF212196 (Falkland Is.)
Botryoglossum platycarpum AF254160 (South Africa)
- / -
Hymenena venosa AF257365 (South Africa)
84 / *
Acrosorium ciliolatum MH349417 (Chile)
- / -
99 / *
Acrosorium flabellatum LC821160 (Japan)
Polyneura bonnemaisonii AF257437 (France)
Polyneura latissima AF257438 (OR, USA)
99 / *
Pseudopolyneura japonica LC821187 (Japan)
99 / *
Pseudopolyneura hyacinthina LC821184 (Tanegashima I.) 1)
72 / *
55 / 0.96
73 / *
Erythroglossum latum LC821167 (Japan)
- / -
99 / *
"Sorella" pulchra LC821168 (Tanegashima I.)
Phycodrys novae-zelandiae GQ479933 (New Zealand)
Heterodoxia denticulata JX110922 (WA, Australia)
56 / *
Hymenenopsis heterophylla JF495095 (New Zealand)
- / -
63 / -
Phycodrys rubens JX110932 (NB, Canada)
- / -
Cladodonta lyalli AF254170 (Argentina)
- / -
Nienburgia andersoniana AF257397
91 / *
Phycodrys ovifolia AF257423 (Chile)
Polyneuropsis stolonifera AF257439 (CA, USA)
- / -
Nitophyllum adhaerens AF257399 (TX, USA)
* / *
Nitophylloideae sp.1 TNE LC821175 (Tanegashima I.)
* / *
Nitophylloideae sp.2 TNE LC821176 (Tanegashima I.)
75 / *
Valeriemaya sp.2 TAS KY682900 (TAS, Australia)
99 / *
Nitophyllum punctatum MK814699 (Spain)
96 / *
Nitophyllum sp. TNE LC821177 (Tanegashima I.)
99 / *
Nitophyllum hommersandii AY118270 (Taiwan)
- / -
Augophyllum wysorii AF257405 (Panama)
Calonitophyllum medium AF254167 (NC, USA)
- / -
Martensia jejuensis LC821172 (Japan)
- / -
* / *
Martensia sp.2 TNE LC821174 (Tanegashima I.)
- / -
Martensia sp.1 TNE LC821173 (Tanegashima I.)
- / 0.96
Continued

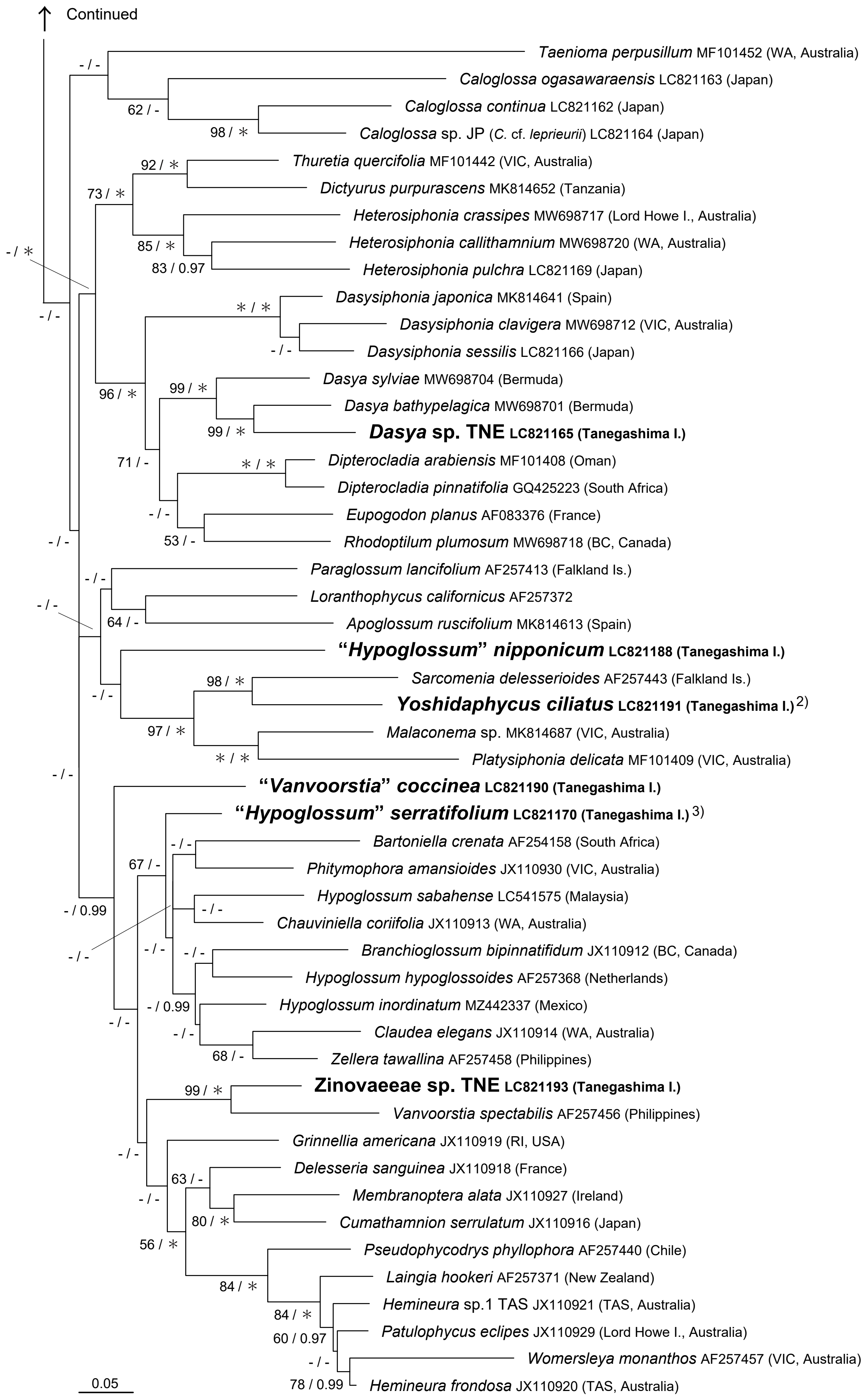

[1]KF305299 (South Korea *Holotype), LC821185, LC821186 (Tanegashima I.) had identical sequences. [2]LC821192 (Tanegashima I.) had identical sequence. [3]LC821171 (Tanegashima I.) had identical sequence.

S29A Fig. Maximum likelihood phylogeny for Delesseriaceae species based on *rbc*L DNA sequences. Values are indicated at the branches: bootstrap (BP; ≥ 50%) and Bayesian posterior probabilities (PP; ≥ 0.95). Asterisks (*) indicate 100% BP and 1.00 PP.

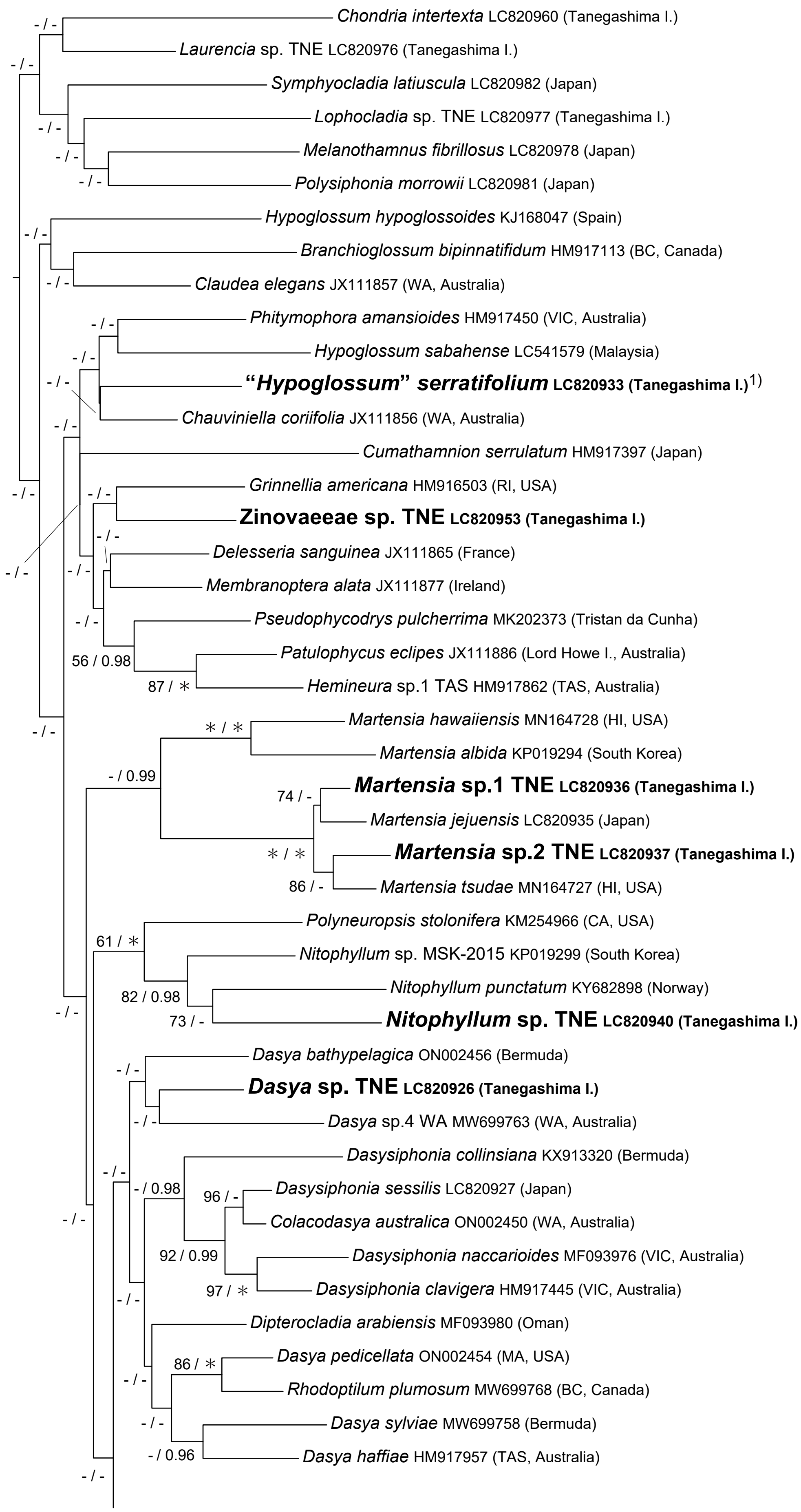

Chondria intertexta LC820960 (Tanegashima I.)
Laurencia sp. TNE LC820976 (Tanegashima I.)
Symphyocladia latiuscula LC820982 (Japan)
Lophocladia sp. TNE LC820977 (Tanegashima I.)
Melanothamnus fibrillosus LC820978 (Japan)
Polysiphonia morrowii LC820981 (Japan)
Hypoglossum hypoglossoides KJ168047 (Spain)
Branchioglossum bipinnatifidum HM917113 (BC, Canada)
Claudea elegans JX111857 (WA, Australia)
Phitymophora amansioides HM917450 (VIC, Australia)
Hypoglossum sabahense LC541579 (Malaysia)
"Hypoglossum" serratifolium LC820933 (Tanegashima I.)1)
Chauviniella coriifolia JX111856 (WA, Australia)
Cumathamnion serrulatum HM917397 (Japan)
Grinnellia americana HM916503 (RI, USA)
Zinovaeeae sp. TNE LC820953 (Tanegashima I.)
Delesseria sanguinea JX111865 (France)
Membranoptera alata JX111877 (Ireland)
Pseudophycodrys pulcherrima MK202373 (Tristan da Cunha)
Patulophycus eclipes JX111886 (Lord Howe I., Australia)
Hemineura sp.1 TAS HM917862 (TAS, Australia)
Martensia hawaiiensis MN164728 (HI, USA)
Martensia albida KP019294 (South Korea)
Martensia sp.1 TNE LC820936 (Tanegashima I.)
Martensia jejuensis LC820935 (Japan)
Martensia sp.2 TNE LC820937 (Tanegashima I.)
Martensia tsudae MN164727 (HI, USA)
Polyneuropsis stolonifera KM254966 (CA, USA)
Nitophyllum sp. MSK-2015 KP019299 (South Korea)
Nitophyllum punctatum KY682898 (Norway)
Nitophyllum sp. TNE LC820940 (Tanegashima I.)
Dasya bathypelagica ON002456 (Bermuda)
Dasya sp. TNE LC820926 (Tanegashima I.)
Dasya sp.4 WA MW699763 (WA, Australia)
Dasysiphonia collinsiana KX913320 (Bermuda)
Dasysiphonia sessilis LC820927 (Japan)
Colacodasya australica ON002450 (WA, Australia)
Dasysiphonia naccarioides MF093976 (VIC, Australia)
Dasysiphonia clavigera HM917445 (VIC, Australia)
Dipterocladia arabiensis MF093980 (Oman)
Dasya pedicellata ON002454 (MA, USA)
Rhodoptilum plumosum MW699768 (BC, Canada)
Dasya sylviae MW699758 (Bermuda)
Dasya haffiae HM917957 (TAS, Australia)
56 / 0.98
87 / *
* / *
- / 0.99
74 / -
* / *
86 / -
61 / *
82 / 0.98
73 / -
- / 0.98
96 / -
92 / 0.99
97 / *
86 / *
- / 0.96

↓ Continued

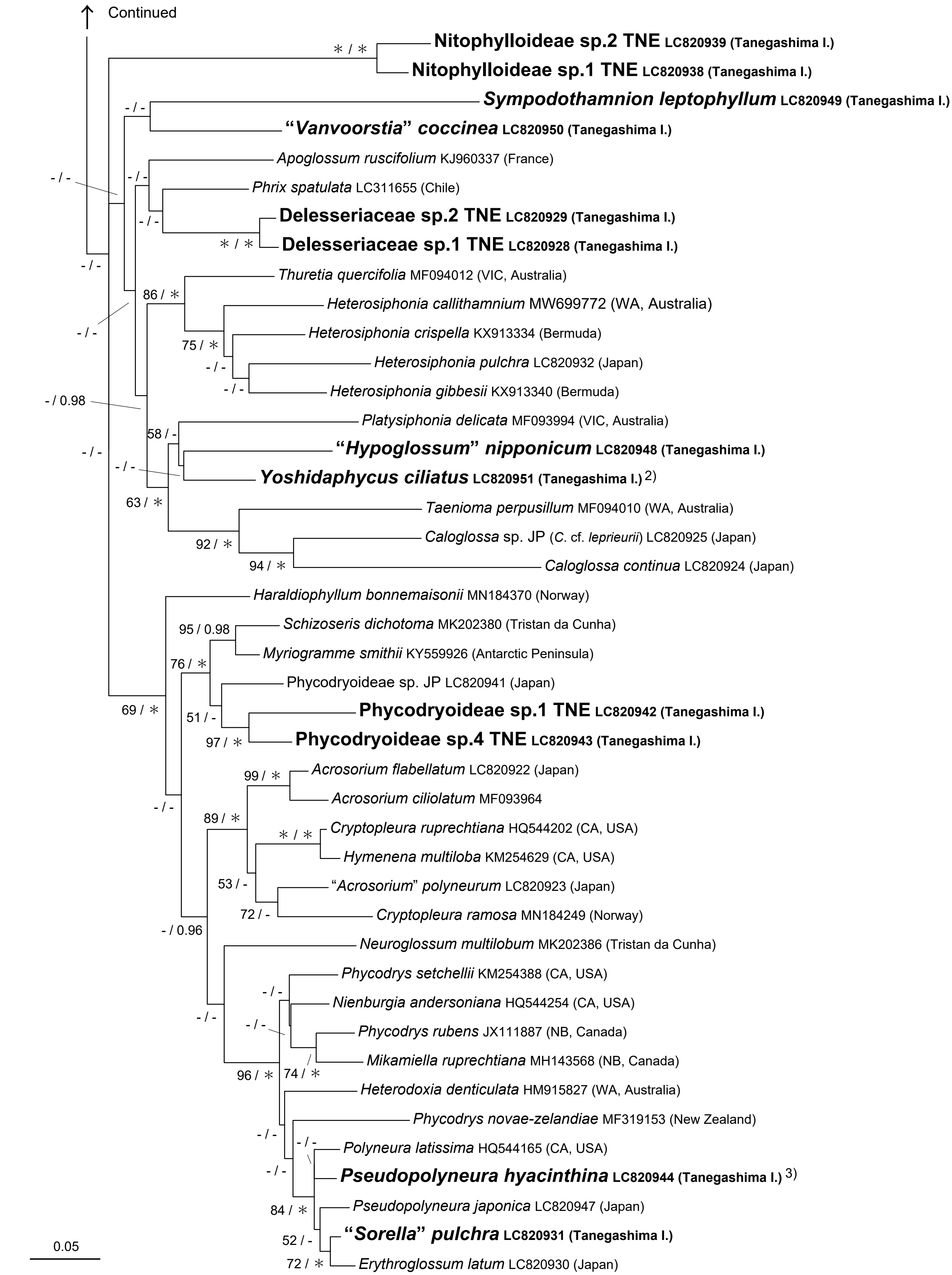

[1)]LC820934 (Tanegashima I.) had identical sequence. [2)]LC820952 (Tanegashima I.) had identical sequence. [3)]LC820945, LC820946 (Tanegashima I.) had identical sequences.

S29B Fig. Maximum likelihood phylogeny for Delesseriaceae species based on *cox*1 DNA sequences. Values are indicated at the branches: bootstrap (BP; ≥ 50%) and Bayesian posterior probabilities (PP; ≥ 0.95). Asterisks (∗) indicate 100% BP and 1.00 PP.

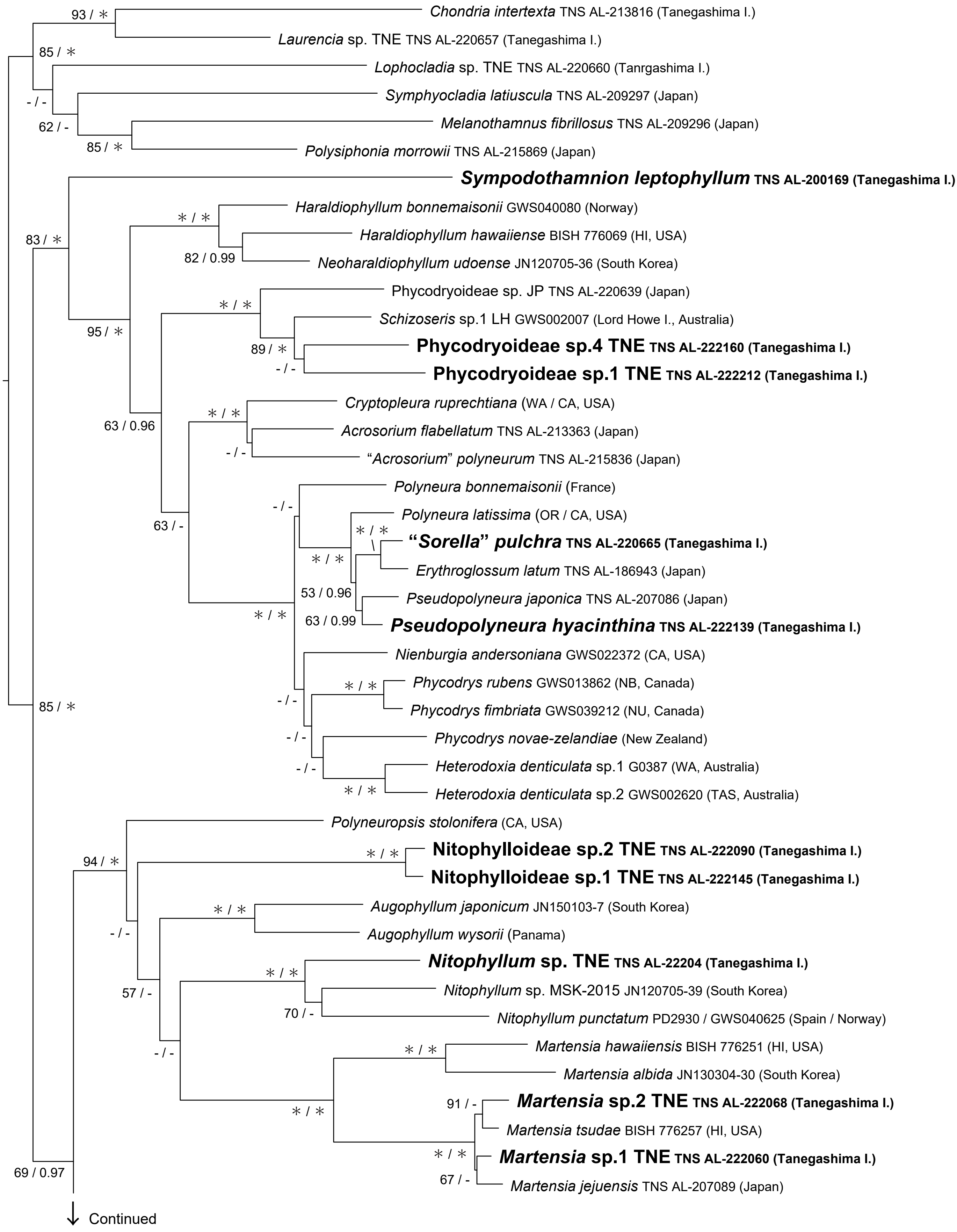
Chondria intertexta TNS AL-213816 (Tanegashima I.)
Laurencia sp. TNE TNS AL-220657 (Tanegashima I.)
Lophocladia sp. TNE TNS AL-220660 (Tanrgashima I.)
Symphyocladia latiuscula TNS AL-209297 (Japan)
Melanothamnus fibrillosus TNS AL-209296 (Japan)
Polysiphonia morrowii TNS AL-215869 (Japan)
Sympodothamnion leptophyllum TNS AL-200169 (Tanegashima I.)
Haraldiophyllum bonnemaisonii GWS040080 (Norway)
Haraldiophyllum hawaiiense BISH 776069 (HI, USA)
Neoharaldiophyllum udoense JN120705-36 (South Korea)
Phycodryoideae sp. JP TNS AL-220639 (Japan)
Schizoseris sp.1 LH GWS002007 (Lord Howe I., Australia)
Phycodryoideae sp.4 TNE TNS AL-222160 (Tanegashima I.)
Phycodryoideae sp.1 TNE TNS AL-222212 (Tanegashima I.)
Cryptopleura ruprechtiana (WA / CA, USA)
Acrosorium flabellatum TNS AL-213363 (Japan)
"Acrosorium" polyneurum TNS AL-215836 (Japan)
Polyneura bonnemaisonii (France)
Polyneura latissima (OR / CA, USA)
"Sorella" pulchra TNS AL-220665 (Tanegashima I.)
Erythroglossum latum TNS AL-186943 (Japan)
Pseudopolyneura japonica TNS AL-207086 (Japan)
Pseudopolyneura hyacinthina TNS AL-222139 (Tanegashima I.)
Nienburgia andersoniana GWS022372 (CA, USA)
Phycodrys rubens GWS013862 (NB, Canada)
Phycodrys fimbriata GWS039212 (NU, Canada)
Phycodrys novae-zelandiae (New Zealand)
Heterodoxia denticulata sp.1 G0387 (WA, Australia)
Heterodoxia denticulata sp.2 GWS002620 (TAS, Australia)
Polyneuropsis stolonifera (CA, USA)
Nitophylloideae sp.2 TNE TNS AL-222090 (Tanegashima I.)
Nitophylloideae sp.1 TNE TNS AL-222145 (Tanegashima I.)
Augophyllum japonicum JN150103-7 (South Korea)
Augophyllum wysorii (Panama)
Nitophyllum sp. TNE TNS AL-22204 (Tanegashima I.)
Nitophyllum sp. MSK-2015 JN120705-39 (South Korea)
Nitophyllum punctatum PD2930 / GWS040625 (Spain / Norway)
Martensia hawaiiensis BISH 776251 (HI, USA)
Martensia albida JN130304-30 (South Korea)
Martensia sp.2 TNE TNS AL-222068 (Tanegashima I.)
Martensia tsudae BISH 776257 (HI, USA)
Martensia sp.1 TNE TNS AL-222060 (Tanegashima I.)
Martensia jejuensis TNS AL-207089 (Japan)
93 / *
85 / *
- / -
62 / -
85 / *
83 / *
* / *
82 / 0.99
95 / *
* / *
89 / *
- / -
63 / 0.96
* / *
- / -
63 / -
- / -
* / *
* / *
53 / 0.96
63 / 0.99
* / *
- / -
* / *
- / -
- / -
* / *
85 / *
94 / *
* / *
* / *
- / -
* / *
57 / -
70 / -
- / -
* / *
* / *
91 / -
* / *
67 / -
69 / 0.97
Continued

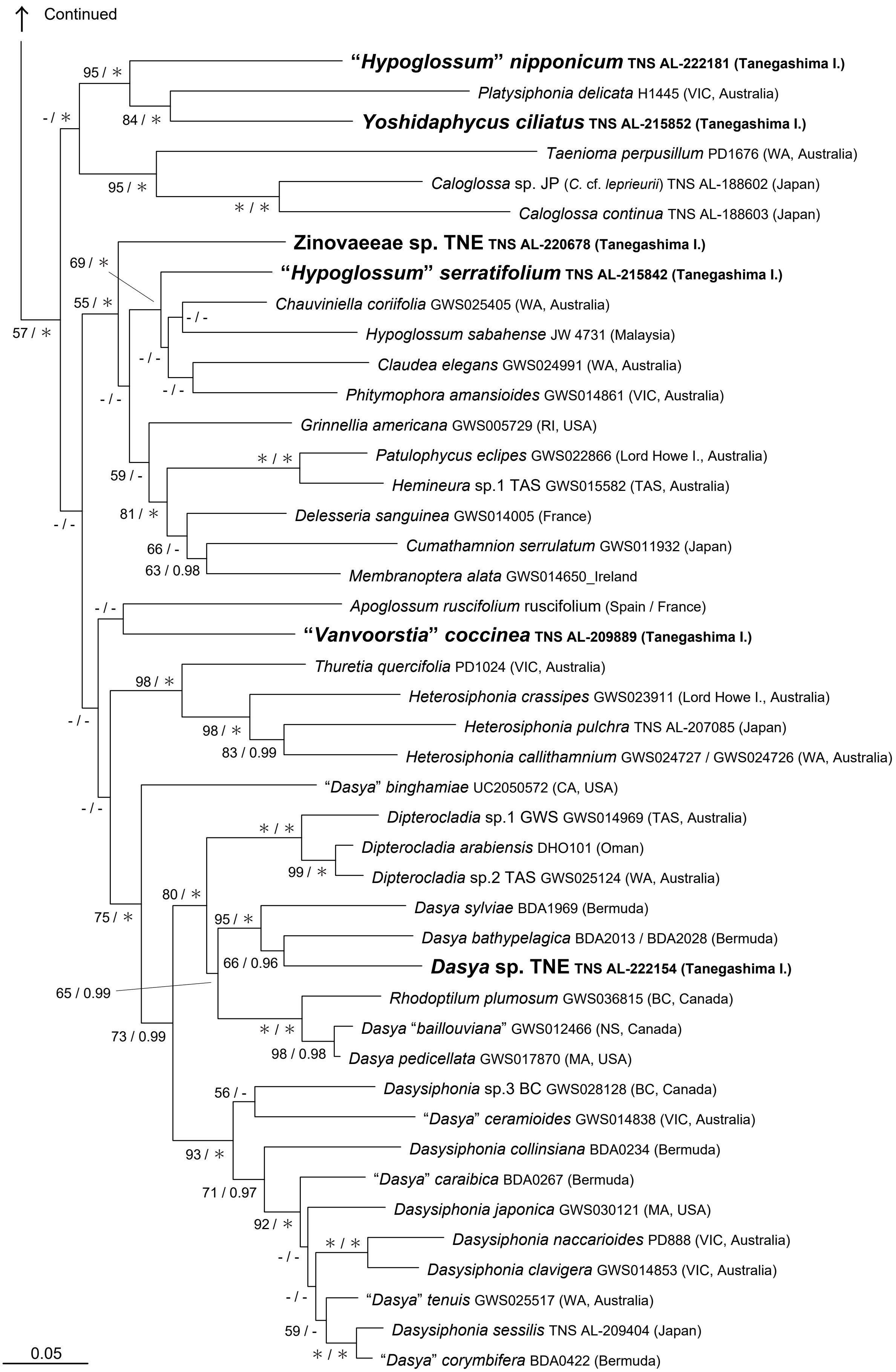

S29C Fig. Maximum likelihood phylogeny for Delesseriaceae species based on combined *rbc*L and *cox*1 DNA sequences. Values are indicated at the branches: bootstrap (BP; ≥ 50%) and Bayesian posterior probabilities (PP; ≥ 0.95). Asterisks (∗) indicate 100% BP and 1.00 PP.
