# Supplementary material for: DNA-based floristic survey of red algae (Rhodophyta) growing in the mesophotic coral ecosystems (MCEs) offshore of Tanegashima Island, northern Ryukyu Archipelago, Japan
Source: PLoS One. 2025 Mar 10;20(3):e0316067. doi: 10.1371/journal.pone.0316067 (PMC11893125; doi:10.1371/journal.pone.0316067)
Supplement: S5 File — Maximum likelihood phylogeny of red algae collected from offshore Tanegashima Island. (ZIP) [file pone.0316067.s005.zip › S5_File/S37_Fig.pdf]

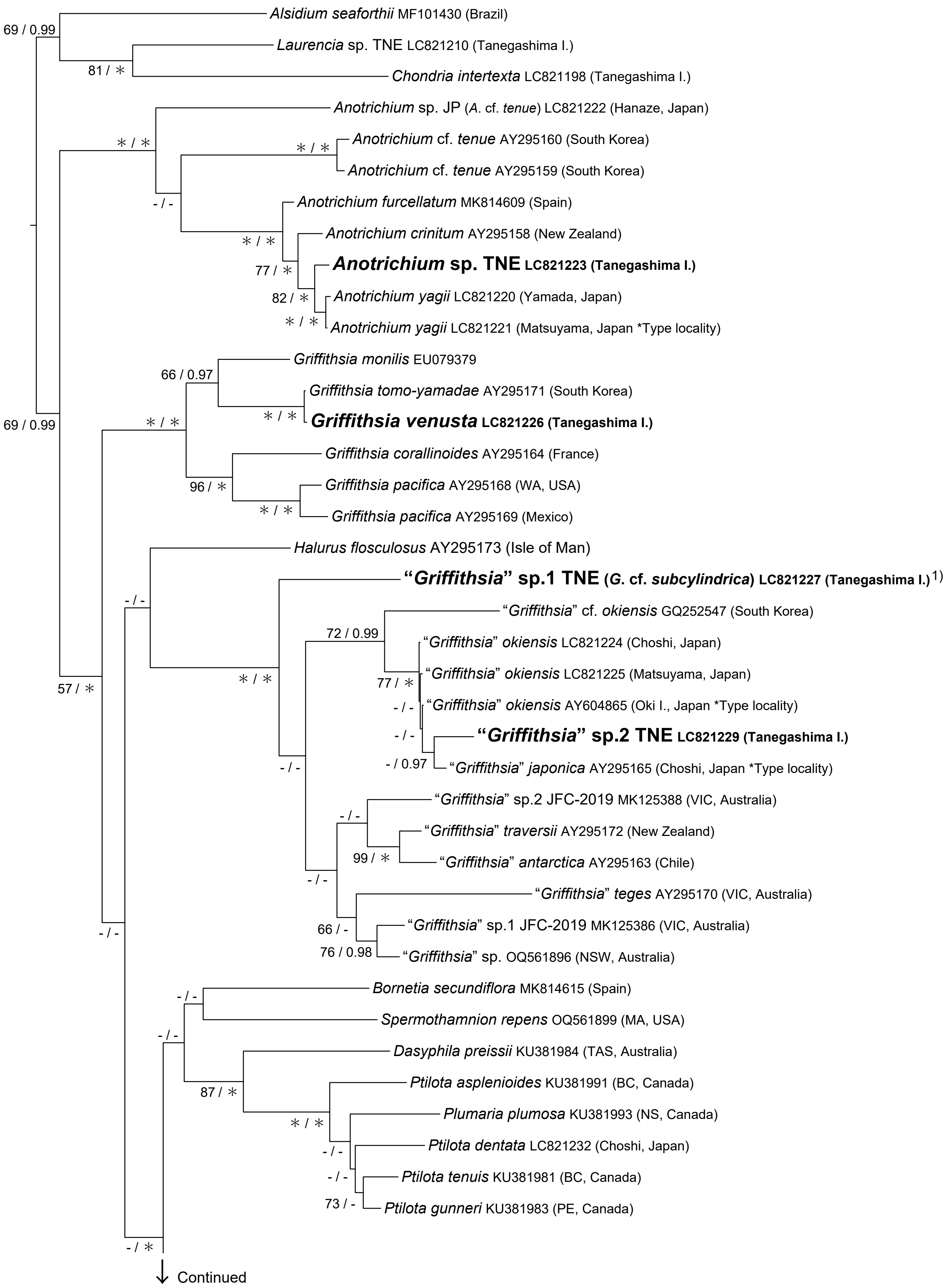

69 / 0.99
Alsidium seaforthii MF101430 (Brazil)
Laurencia sp. TNE LC821210 (Tanegashima I.)
81 / *
Chondria intertexta LC821198 (Tanegashima I.)
Anotrichium sp. JP (A. cf. tenue) LC821222 (Hanaze, Japan)
* / *
Anotrichium cf. tenue AY295160 (South Korea)
* / *
Anotrichium cf. tenue AY295159 (South Korea)
- / -
Anotrichium furcellatum MK814609 (Spain)
Anotrichium crinitum AY295158 (New Zealand)
* / *
Anotrichium sp. TNE LC821223 (Tanegashima I.)
77 / *
82 / *
Anotrichium yagii LC821220 (Yamada, Japan)
* / *
Anotrichium yagii LC821221 (Matsuyama, Japan *Type locality)
Griffithsia monilis EU079379
66 / 0.97
Griffithsia tomo-yamadae AY295171 (South Korea)
69 / 0.99
* / *
* / *
Griffithsia venusta LC821226 (Tanegashima I.)
Griffithsia corallinoides AY295164 (France)
96 / *
Griffithsia pacifica AY295168 (WA, USA)
* / *
Griffithsia pacifica AY295169 (Mexico)
Halurus flosculosus AY295173 (Isle of Man)
"Griffithsia" sp.1 TNE (G. cf. subcylindrica) LC821227 (Tanegashima I.) 1)
- / -
"Griffithsia" cf. okiensis GQ252547 (South Korea)
72 / 0.99
"Griffithsia" okiensis LC821224 (Choshi, Japan)
"Griffithsia" okiensis LC821225 (Matsuyama, Japan)
* / *
77 / *
57 / *
- / -
"Griffithsia" okiensis AY604865 (Oki I., Japan *Type locality)
- / -
"Griffithsia" sp.2 TNE LC821229 (Tanegashima I.)
- / -
- / 0.97
"Griffithsia" japonica AY295165 (Choshi, Japan *Type locality)
"Griffithsia" sp.2 JFC-2019 MK125388 (VIC, Australia)
- / -
"Griffithsia" traversii AY295172 (New Zealand)
99 / *
"Griffithsia" antarctica AY295163 (Chile)
- / -
"Griffithsia" teges AY295170 (VIC, Australia)
- / -
66 / -
"Griffithsia" sp.1 JFC-2019 MK125386 (VIC, Australia)
76 / 0.98
"Griffithsia" sp. OQ561896 (NSW, Australia)
Bornetia secundiflora MK814615 (Spain)
- / -
Spermothamnion repens OQ561899 (MA, USA)
- / -
Dasyphila preissii KU381984 (TAS, Australia)
87 / *
Ptilota asplenioides KU381991 (BC, Canada)
Plumaria plumosa KU381993 (NS, Canada)
* / *
Ptilota dentata LC821232 (Choshi, Japan)
- / -
- / -
Ptilota tenuis KU381981 (BC, Canada)
73 / -
Ptilota gunneri KU381983 (PE, Canada)
- / *
Continued

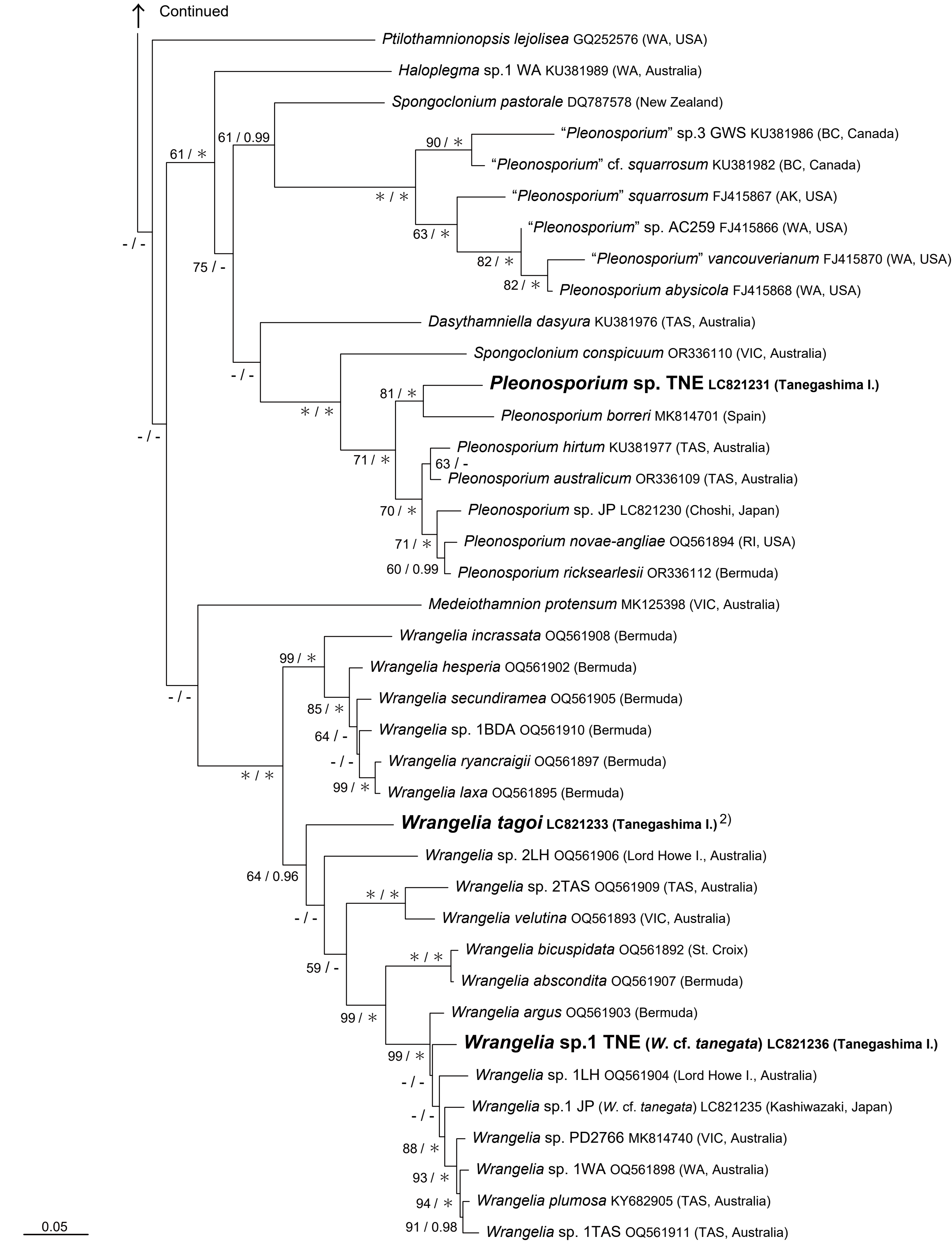

1) LC821228 (Tanegashima I.) had identical sequence. 2) LC821234 (Tanegashima I.) had identical sequence.

S37A Fig. Maximum likelihood phylogeny for Wrangeliaceae species based on *rbc*L DNA sequences.
Values are indicated at the branches: bootstrap (BP; ≥ 50%) and Bayesian posterior probabilities (PP; ≥ 0.95). Asterisks (∗) indicate 100% BP and 1.00 PP.

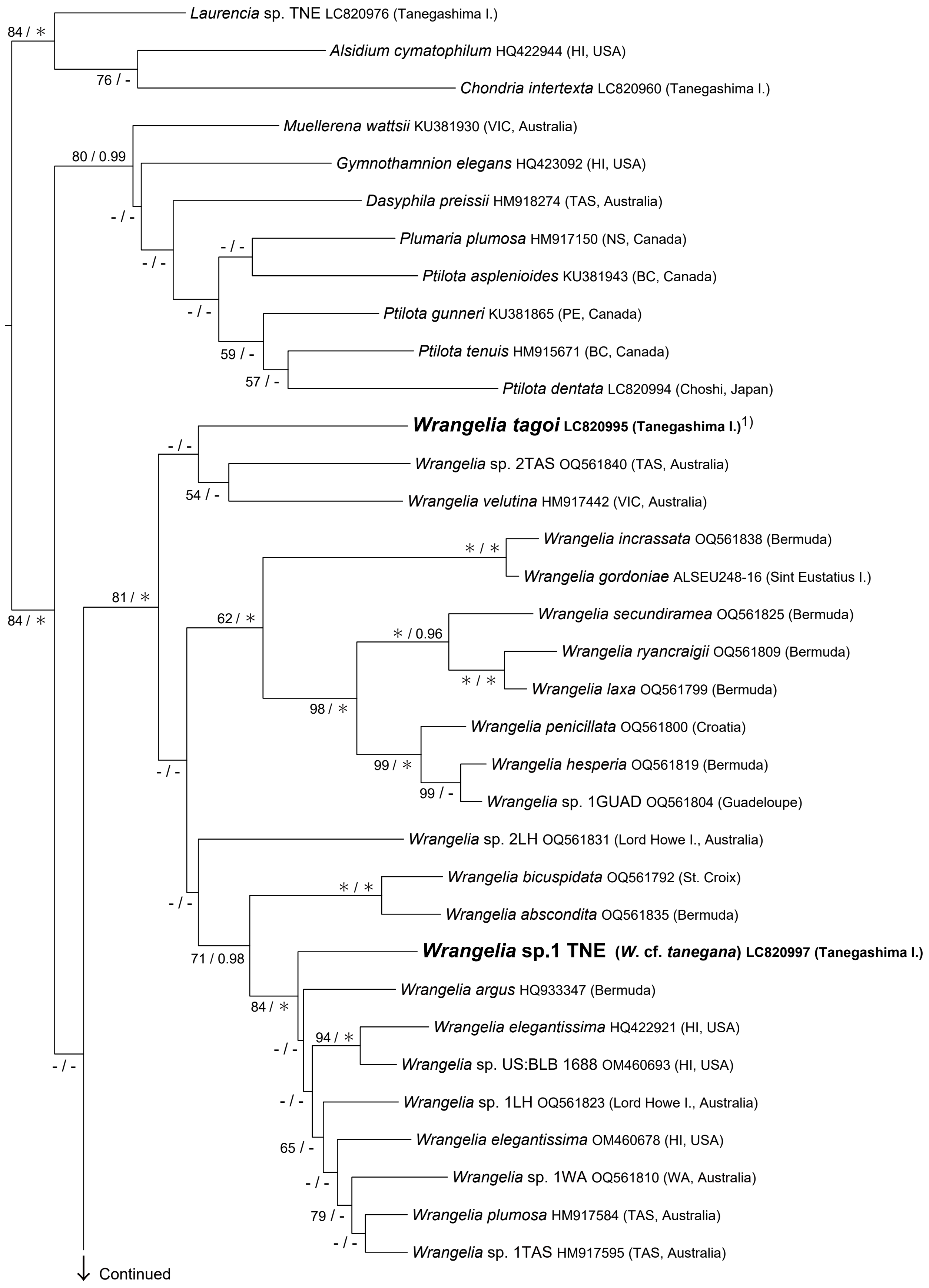

Laurencia sp. TNE LC820976 (Tanegashima I.)
84 / ∗
Alsidium cymatophilum HQ422944 (HI, USA)
76 / -
Chondria intertexta LC820960 (Tanegashima I.)
Muellerena wattsii KU381930 (VIC, Australia)
80 / 0.99
Gymnothamnion elegans HQ423092 (HI, USA)
Dasyphila preissii HM918274 (TAS, Australia)
- / -
Plumaria plumosa HM917150 (NS, Canada)
- / -
Ptilota asplenioides KU381943 (BC, Canada)
- / -
- / -
Ptilota gunneri KU381865 (PE, Canada)
Ptilota tenuis HM915671 (BC, Canada)
59 / -
57 / -
Ptilota dentata LC820994 (Choshi, Japan)
Wrangelia tagoi LC820995 (Tanegashima I.)1)
- / -
Wrangelia sp. 2TAS OQ561840 (TAS, Australia)
54 / -
Wrangelia velutina HM917442 (VIC, Australia)
∗ / ∗
Wrangelia incrassata OQ561838 (Bermuda)
Wrangelia gordoniae ALSEU248-16 (Sint Eustatius I.)
81 / ∗
84 / ∗
62 / ∗
Wrangelia secundiramea OQ561825 (Bermuda)
∗ / 0.96
Wrangelia ryancraigii OQ561809 (Bermuda)
∗ / ∗
Wrangelia laxa OQ561799 (Bermuda)
98 / ∗
Wrangelia penicillata OQ561800 (Croatia)
- / -
99 / ∗
Wrangelia hesperia OQ561819 (Bermuda)
99 / -
Wrangelia sp. 1GUAD OQ561804 (Guadeloupe)
Wrangelia sp. 2LH OQ561831 (Lord Howe I., Australia)
∗ / ∗
Wrangelia bicuspidata OQ561792 (St. Croix)
- / -
Wrangelia abscondita OQ561835 (Bermuda)
71 / 0.98
Wrangelia sp.1 TNE (W. cf. tanegana) LC820997 (Tanegashima I.)
Wrangelia argus HQ933347 (Bermuda)
84 / ∗
94 / ∗
Wrangelia elegantissima HQ422921 (HI, USA)
- / -
Wrangelia sp. US:BLB 1688 OM460693 (HI, USA)
- / -
- / -
Wrangelia sp. 1LH OQ561823 (Lord Howe I., Australia)
65 / -
Wrangelia elegantissima OM460678 (HI, USA)
- / -
Wrangelia sp. 1WA OQ561810 (WA, Australia)
79 / -
Wrangelia plumosa HM917584 (TAS, Australia)
- / -
Wrangelia sp. 1TAS HM917595 (TAS, Australia)
Continued

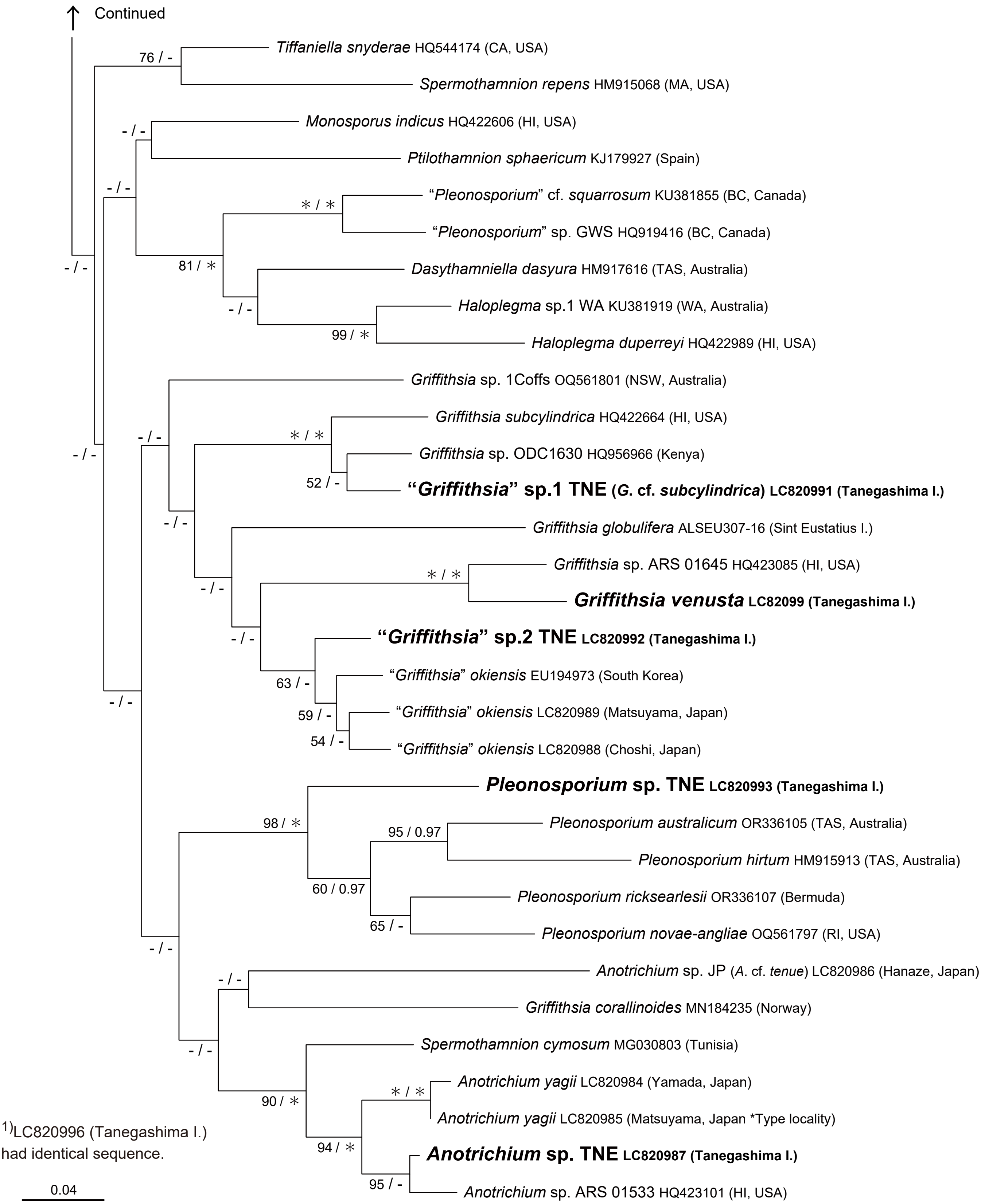

S37B Fig. Maximum likelihood phylogeny for Wrangeliaceae species based on *cox*1 DNA sequences.
Values are indicated at the branches: bootstrap (BP; ≥ 50%) and Bayesian posterior probabilities (PP; ≥ 0.95). Asterisks (∗) indicate 100% BP and 1.00 PP.

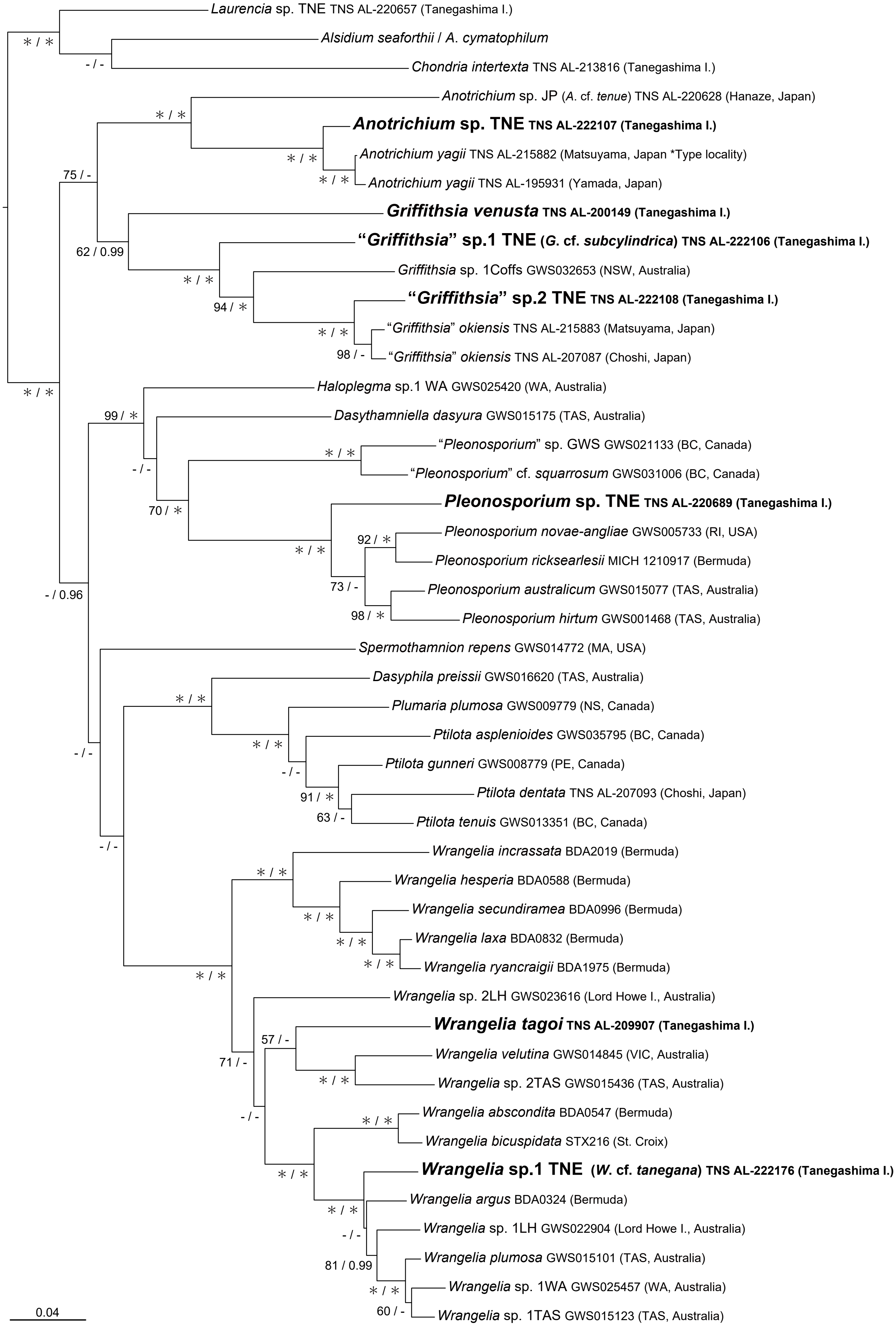

S37C Fig. Maximum likelihood phylogeny for Wrangeliaceae species based on combined *rbc*L and *cox*1 DNA sequences. Values are indicated at the branches: bootstrap (BP; ≥ 50%) and Bayesian posterior probabilities (PP; ≥ 0.95). Asterisks (∗) indicate 100% BP and 1.00 PP.
