# Supplementary material for: DNA-based floristic survey of red algae (Rhodophyta) growing in the mesophotic coral ecosystems (MCEs) offshore of Tanegashima Island, northern Ryukyu Archipelago, Japan
Source: PLoS One. 2025 Mar 10;20(3):e0316067. doi: 10.1371/journal.pone.0316067 (PMC11893125; doi:10.1371/journal.pone.0316067)
Supplement: S5 File — Maximum likelihood phylogeny of red algae collected from offshore Tanegashima Island. (ZIP) [file pone.0316067.s005.zip › S5_File/S38_Fig.pdf]

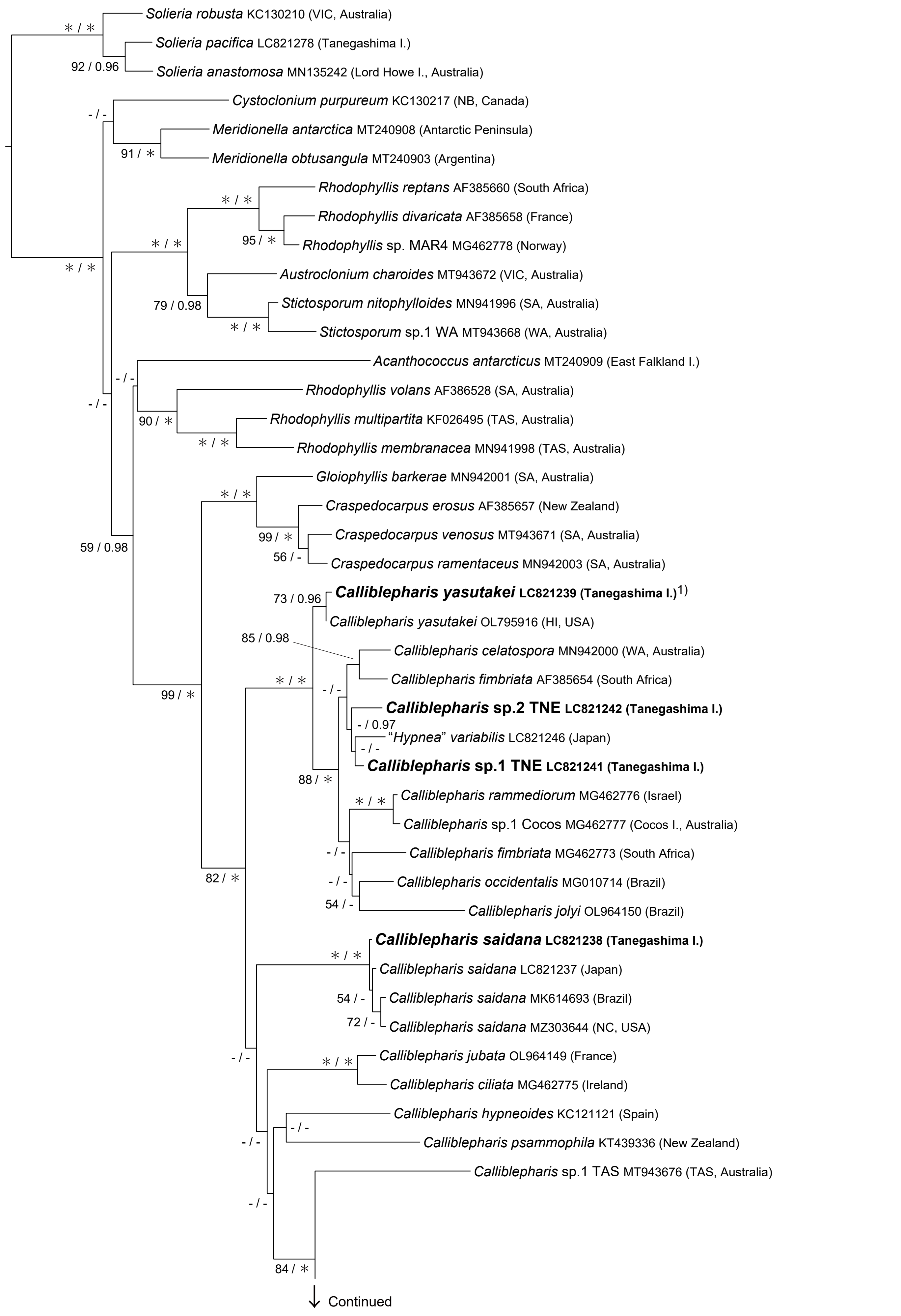
Solieria robusta KC130210 (VIC, Australia)
Solieria pacifica LC821278 (Tanegashima I.)
Solieria anastomosa MN135242 (Lord Howe I., Australia)
Cystoclonium purpureum KC130217 (NB, Canada)
Meridionella antarctica MT240908 (Antarctic Peninsula)
Meridionella obtusangula MT240903 (Argentina)
Rhodophyllis reptans AF385660 (South Africa)
Rhodophyllis divaricata AF385658 (France)
Rhodophyllis sp. MAR4 MG462778 (Norway)
Austroclonium charoides MT943672 (VIC, Australia)
Stictosporum nitophylloides MN941996 (SA, Australia)
Stictosporum sp.1 WA MT943668 (WA, Australia)
Acanthococcus antarcticus MT240909 (East Falkland I.)
Rhodophyllis volans AF386528 (SA, Australia)
Rhodophyllis multipartita KF026495 (TAS, Australia)
Rhodophyllis membranacea MN941998 (TAS, Australia)
Gloiophyllis barkerae MN942001 (SA, Australia)
Craspedocarpus erosus AF385657 (New Zealand)
Craspedocarpus venosus MT943671 (SA, Australia)
Craspedocarpus ramentaceus MN942003 (SA, Australia)
Calliblepharis yasutakei LC821239 (Tanegashima I.)1)
Calliblepharis yasutakei OL795916 (HI, USA)
Calliblepharis celatospora MN942000 (WA, Australia)
Calliblepharis fimbriata AF385654 (South Africa)
Calliblepharis sp.2 TNE LC821242 (Tanegashima I.)
"Hypnea" variabilis LC821246 (Japan)
Calliblepharis sp.1 TNE LC821241 (Tanegashima I.)
Calliblepharis rammediorum MG462776 (Israel)
Calliblepharis sp.1 Cocos MG462777 (Cocos I., Australia)
Calliblepharis fimbriata MG462773 (South Africa)
Calliblepharis occidentalis MG010714 (Brazil)
Calliblepharis jolyi OL964150 (Brazil)
Calliblepharis saidana LC821238 (Tanegashima I.)
Calliblepharis saidana LC821237 (Japan)
Calliblepharis saidana MK614693 (Brazil)
Calliblepharis saidana MZ303644 (NC, USA)
Calliblepharis jubata OL964149 (France)
Calliblepharis ciliata MG462775 (Ireland)
Calliblepharis hypneoides KC121121 (Spain)
Calliblepharis psammophila KT439336 (New Zealand)
Calliblepharis sp.1 TAS MT943676 (TAS, Australia)
Continued

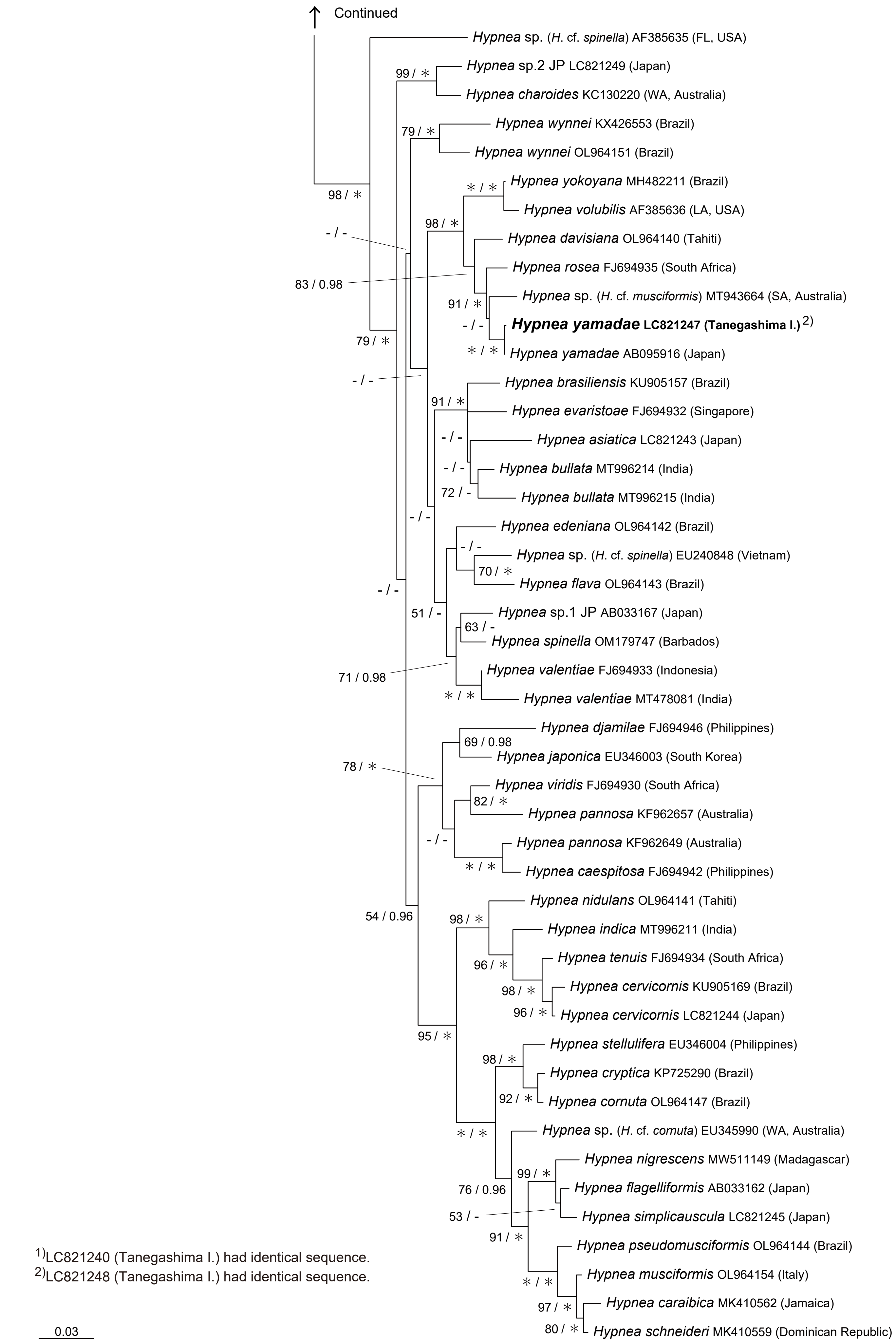

1)LC821240 (Tanegashima I.) had identical sequence.
2)LC821248 (Tanegashima I.) had identical sequence.

S38A Fig. Maximum likelihood phylogeny for Cystocloniaceae species based on *rbc*L DNA sequences.
Values are indicated at the branches: bootstrap (BP; ≥ 50%) and Bayesian posterior probabilities (PP; ≥ 0.95). Asterisks (∗) indicate 100% BP and 1.00 PP.

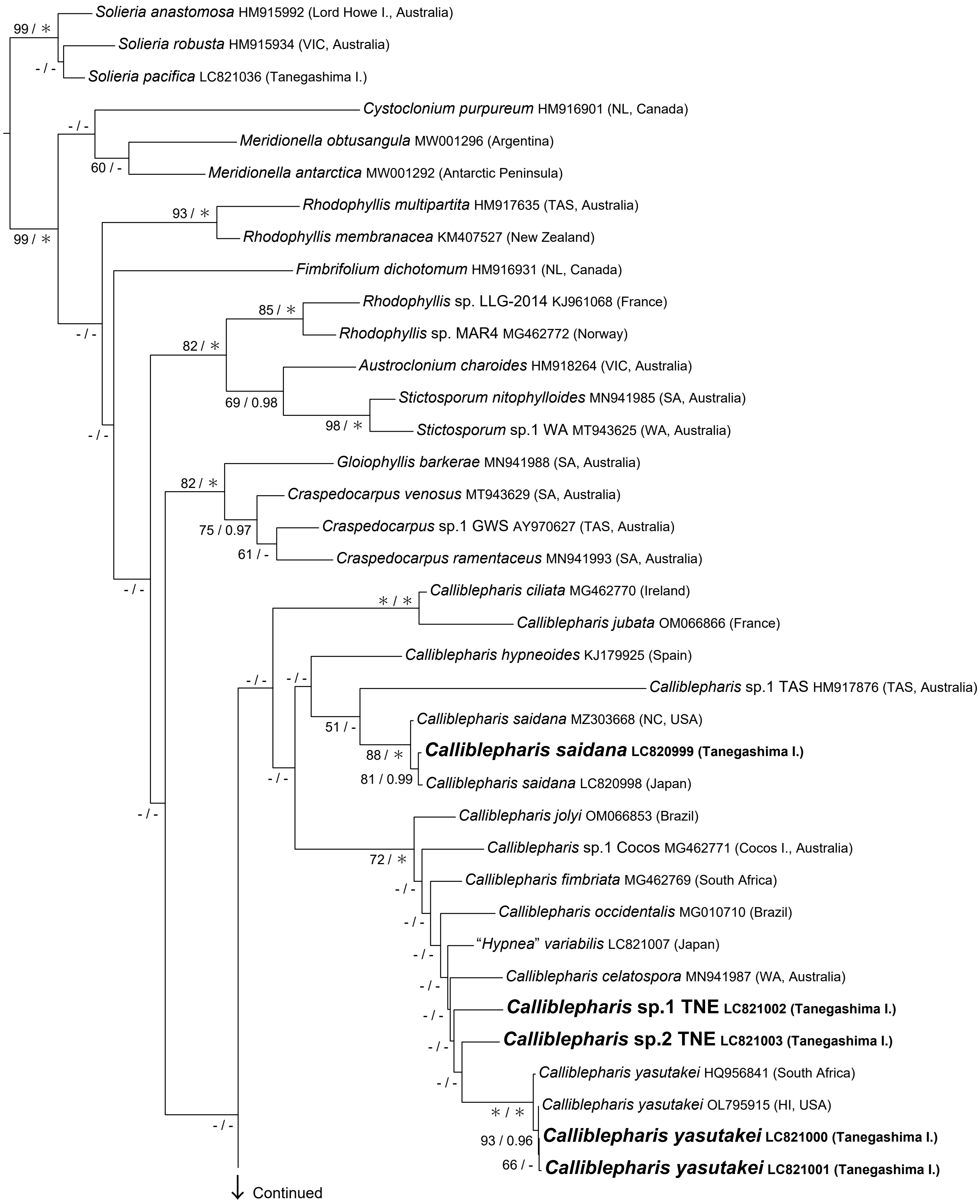

Solieria anastomosa HM915992 (Lord Howe I., Australia)
Solieria robusta HM915934 (VIC, Australia)
Solieria pacifica LC821036 (Tanegashima I.)
Cystoclonium purpureum HM916901 (NL, Canada)
Meridionella obtusangula MW001296 (Argentina)
Meridionella antarctica MW001292 (Antarctic Peninsula)
Rhodophyllis multipartita HM917635 (TAS, Australia)
Rhodophyllis membranacea KM407527 (New Zealand)
Fimbrifolium dichotomum HM916931 (NL, Canada)
Rhodophyllis sp. LLG-2014 KJ961068 (France)
Rhodophyllis sp. MAR4 MG462772 (Norway)
Austroclonium charoides HM918264 (VIC, Australia)
Stictosporum nitophylloides MN941985 (SA, Australia)
Stictosporum sp.1 WA MT943625 (WA, Australia)
Gloiophyllis barkerae MN941988 (SA, Australia)
Craspedocarpus venosus MT943629 (SA, Australia)
Craspedocarpus sp.1 GWS AY970627 (TAS, Australia)
Craspedocarpus ramentaceus MN941993 (SA, Australia)
Calliblepharis ciliata MG462770 (Ireland)
Calliblepharis jubata OM066866 (France)
Calliblepharis hypneoides KJ179925 (Spain)
Calliblepharis sp.1 TAS HM917876 (TAS, Australia)
Calliblepharis saidana MZ303668 (NC, USA)
Calliblepharis saidana LC820999 (Tanegashima I.)
Calliblepharis saidana LC820998 (Japan)
Calliblepharis jolyi OM066853 (Brazil)
Calliblepharis sp.1 Cocos MG462771 (Cocos I., Australia)
Calliblepharis fimbriata MG462769 (South Africa)
Calliblepharis occidentalis MG010710 (Brazil)
"Hypnea" variabilis LC821007 (Japan)
Calliblepharis celatospora MN941987 (WA, Australia)
Calliblepharis sp.1 TNE LC821002 (Tanegashima I.)
Calliblepharis sp.2 TNE LC821003 (Tanegashima I.)
Calliblepharis yasutakei HQ956841 (South Africa)
Calliblepharis yasutakei OL795915 (HI, USA)
Calliblepharis yasutakei LC821000 (Tanegashima I.)
Calliblepharis yasutakei LC821001 (Tanegashima I.)
99 / *
- / -
- / -
60 / -
99 / *
93 / *
- / -
85 / *
82 / *
69 / 0.98
98 / *
- / -
82 / *
75 / 0.97
61 / -
- / -
* / *
- / -
- / -
51 / -
88 / *
81 / 0.99
- / -
- / -
72 / *
- / -
- / -
- / -
- / -
- / -
- / -
- / -
* / *
93 / 0.96
66 / -
- / -
Continued

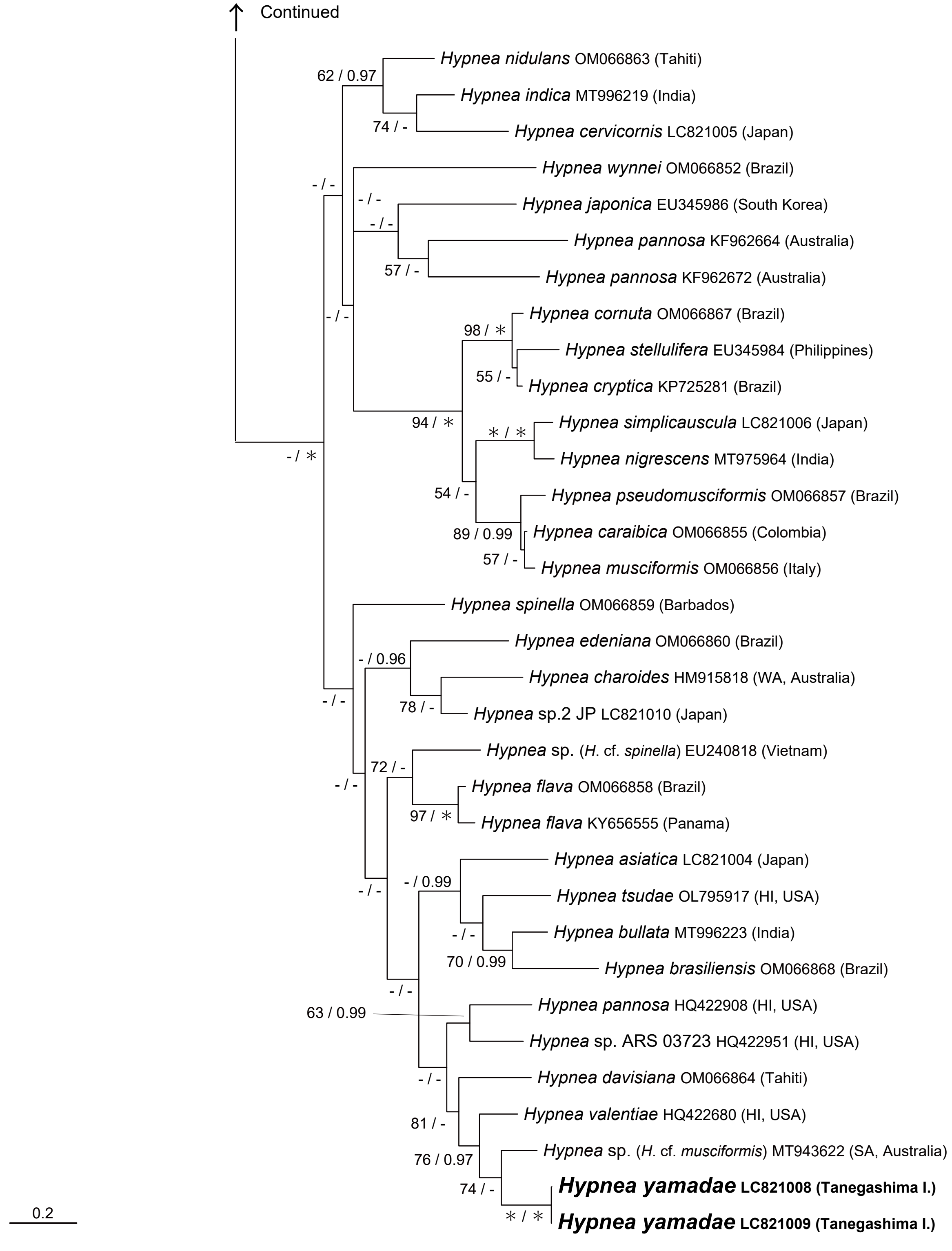

S38B Fig. Maximum likelihood phylogeny for Cystocloniaceae species based on *cox*1 DNA sequences. Values are indicated at the branches: bootstrap (BP; ≥ 50%) and Bayesian posterior probabilities (PP; ≥ 0.95). Asterisks (∗) indicate 100% BP and 1.00 PP.

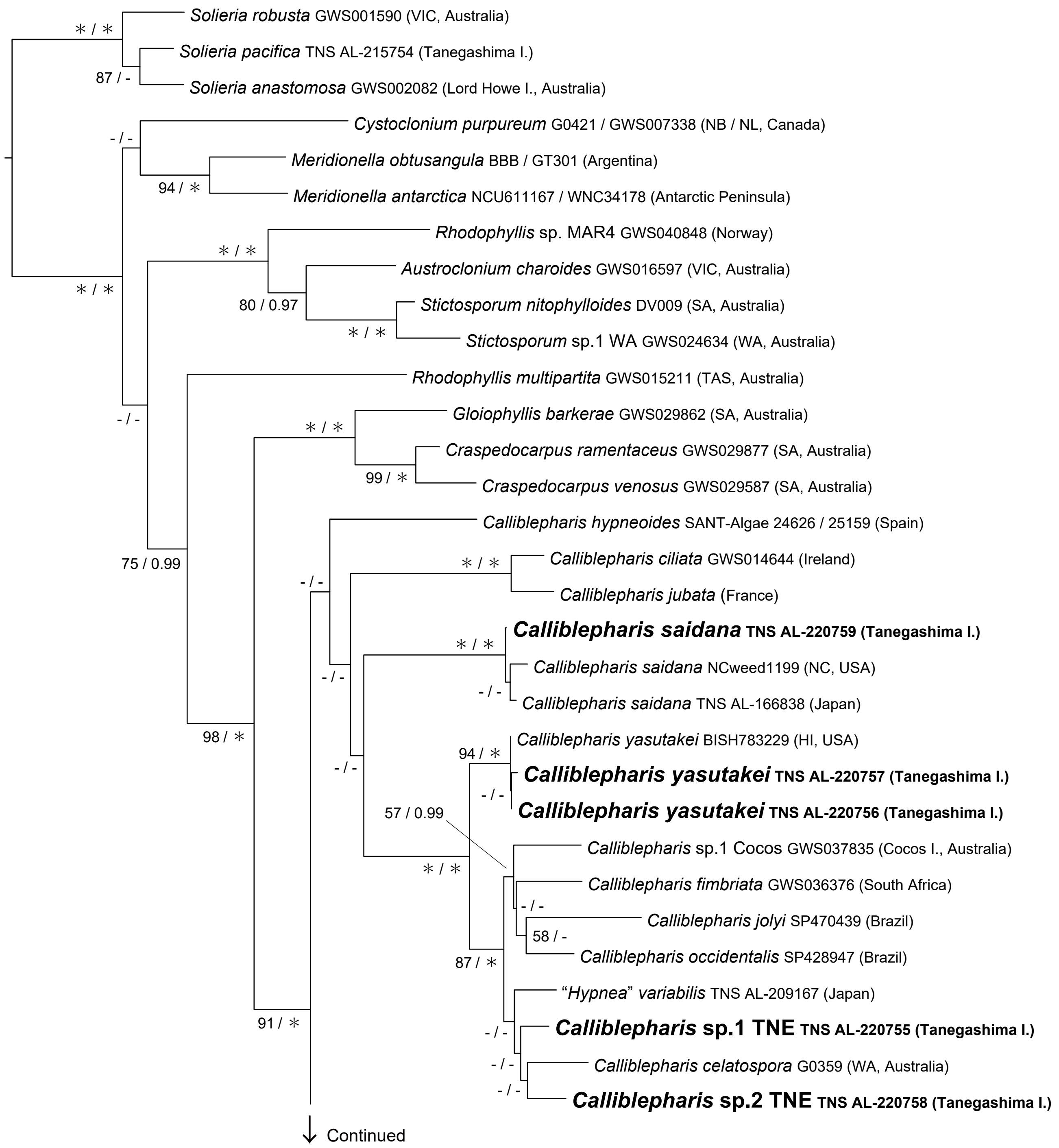
Solieria robusta GWS001590 (VIC, Australia)
Solieria pacifica TNS AL-215754 (Tanegashima I.)
Solieria anastomosa GWS002082 (Lord Howe I., Australia)
Cystoclonium purpureum G0421 / GWS007338 (NB / NL, Canada)
Meridionella obtusangula BBB / GT301 (Argentina)
Meridionella antarctica NCU611167 / WNC34178 (Antarctic Peninsula)
Rhodophyllis sp. MAR4 GWS040848 (Norway)
Austroclonium charoides GWS016597 (VIC, Australia)
Stictosporum nitophylloides DV009 (SA, Australia)
Stictosporum sp.1 WA GWS024634 (WA, Australia)
Rhodophyllis multipartita GWS015211 (TAS, Australia)
Gloiophyllis barkerae GWS029862 (SA, Australia)
Craspedocarpus ramentaceus GWS029877 (SA, Australia)
Craspedocarpus venosus GWS029587 (SA, Australia)
Calliblepharis hypneoides SANT-Algae 24626 / 25159 (Spain)
Calliblepharis ciliata GWS014644 (Ireland)
Calliblepharis jubata (France)
Calliblepharis saidana TNS AL-220759 (Tanegashima I.)
Calliblepharis saidana NCweed1199 (NC, USA)
Calliblepharis saidana TNS AL-166838 (Japan)
Calliblepharis yasutakei BISH783229 (HI, USA)
Calliblepharis yasutakei TNS AL-220757 (Tanegashima I.)
Calliblepharis yasutakei TNS AL-220756 (Tanegashima I.)
Calliblepharis sp.1 Cocos GWS037835 (Cocos I., Australia)
Calliblepharis fimbriata GWS036376 (South Africa)
Calliblepharis jolyi SP470439 (Brazil)
Calliblepharis occidentalis SP428947 (Brazil)
"Hypnea" variabilis TNS AL-209167 (Japan)
Calliblepharis sp.1 TNE TNS AL-220755 (Tanegashima I.)
Calliblepharis celatospora G0359 (WA, Australia)
Calliblepharis sp.2 TNE TNS AL-220758 (Tanegashima I.)
* / *
87 / -
- / -
94 / *
* / *
80 / 0.97
* / *
* / *
- / -
* / *
99 / *
75 / 0.99
- / -
* / *
- / -
* / *
- / -
98 / *
- / -
94 / *
- / -
57 / 0.99
* / *
- / -
58 / -
87 / *
- / -
- / -
- / -
91 / *
Continued

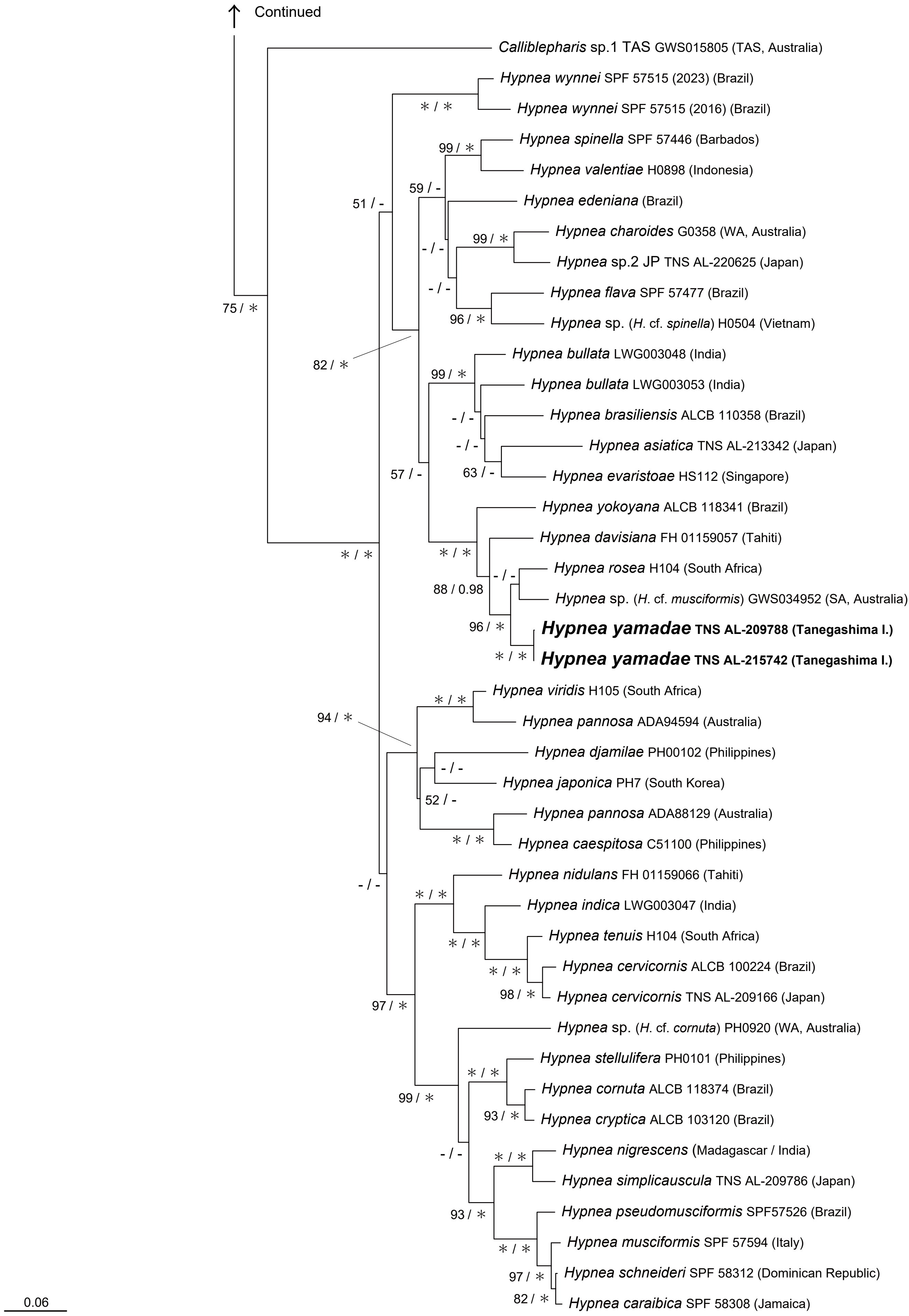

S38C Fig. Maximum likelihood phylogeny for Cystocloniaceae species based on combined *rbc*L and *cox*1 DNA sequences. Values are indicated at the branches: bootstrap (BP; ≥ 50%) and Bayesian posterior probabilities (PP; ≥ 0.95). Asterisks (∗) indicate 100% BP and 1.00 PP.
