# Supplementary material for: DNA-based floristic survey of red algae (Rhodophyta) growing in the mesophotic coral ecosystems (MCEs) offshore of Tanegashima Island, northern Ryukyu Archipelago, Japan
Source: PLoS One. 2025 Mar 10;20(3):e0316067. doi: 10.1371/journal.pone.0316067 (PMC11893125; doi:10.1371/journal.pone.0316067)
Supplement: S5 File — Maximum likelihood phylogeny of red algae collected from offshore Tanegashima Island. (ZIP) [file pone.0316067.s005.zip › S5_File/S39_Fig.pdf]

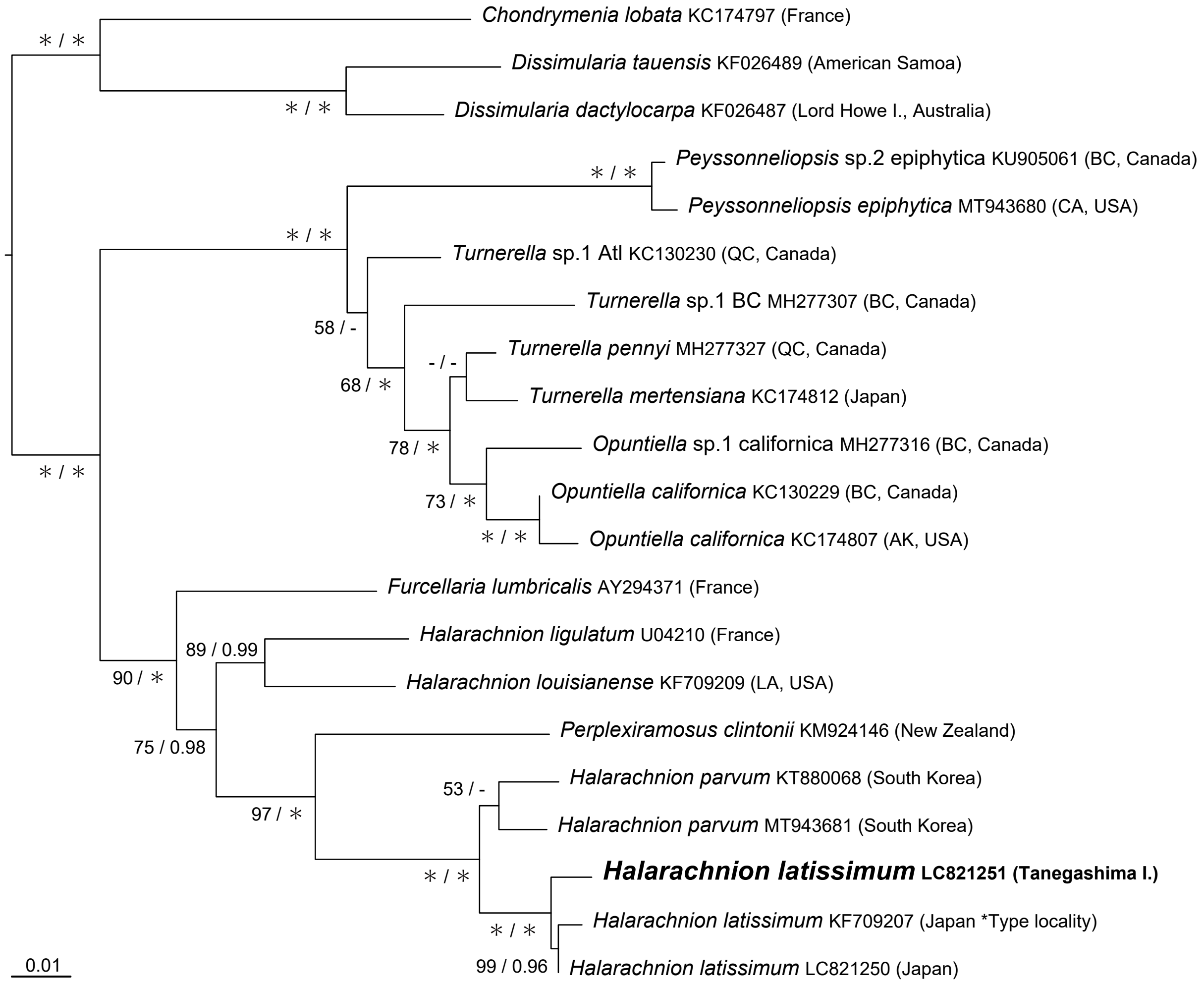


**S39A Fig. Maximum likelihood phylogeny for Furcellariaceae species based on *rbc*L DNA sequences.** Values are indicated at the branches: bootstrap (BP; ≥ 50%) and Bayesian posterior probabilities (PP; ≥ 0.95). Asterisks (*) indicate 100% BP and 1.00 PP.

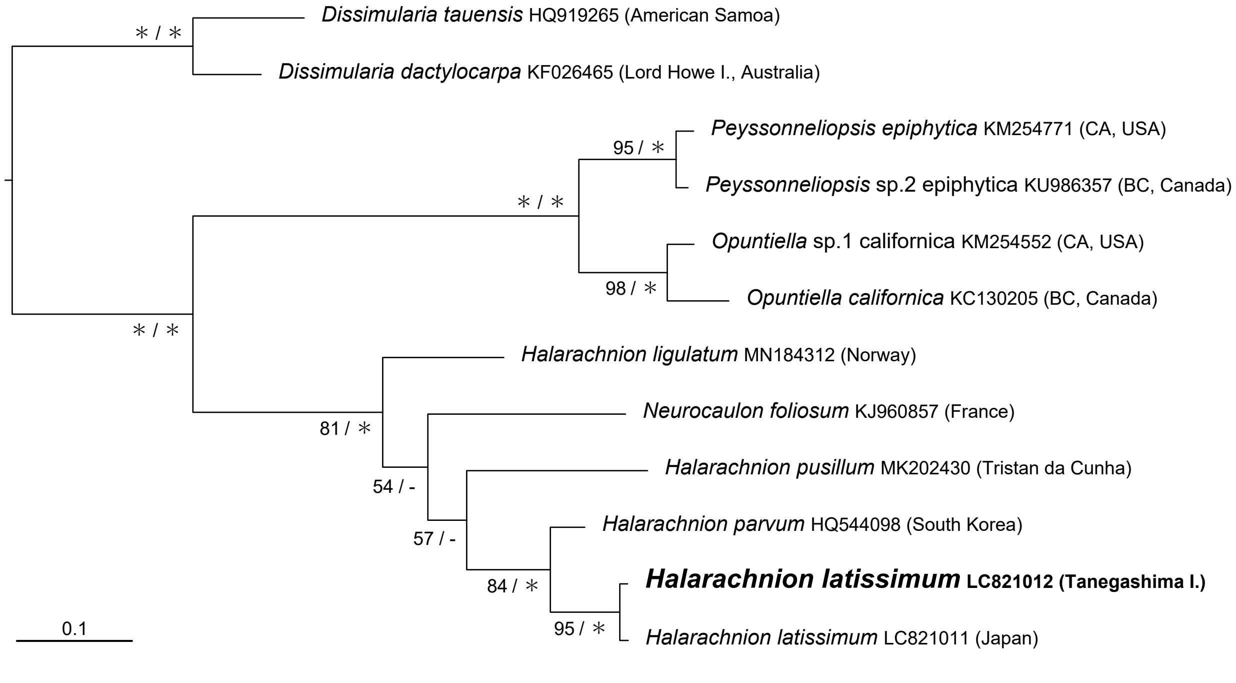


**S39B Fig. Maximum likelihood phylogeny for Furcellariaceae species based on *cox*1 DNA sequences.** Values are indicated at the branches: bootstrap (BP; ≥ 50%) and Bayesian posterior probabilities (PP; ≥ 0.95). Asterisks (*) indicate 100% BP and 1.00 PP.
