# Supplementary material for: DNA-based floristic survey of red algae (Rhodophyta) growing in the mesophotic coral ecosystems (MCEs) offshore of Tanegashima Island, northern Ryukyu Archipelago, Japan
Source: PLoS One. 2025 Mar 10;20(3):e0316067. doi: 10.1371/journal.pone.0316067 (PMC11893125; doi:10.1371/journal.pone.0316067)
Supplement: S5 File — Maximum likelihood phylogeny of red algae collected from offshore Tanegashima Island. (ZIP) [file pone.0316067.s005.zip › S5_File/S40_Fig.pdf]

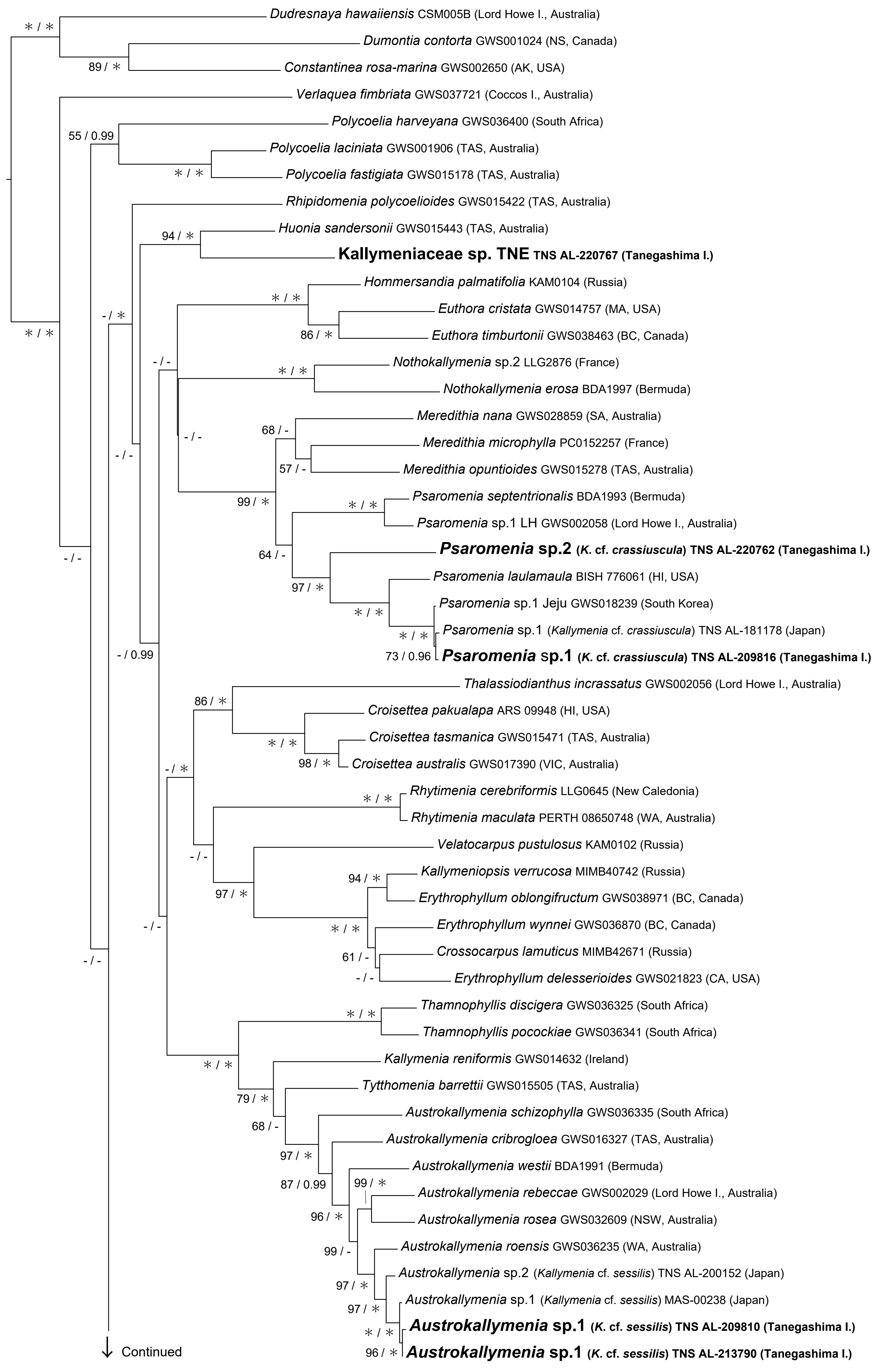

Dudresnaya hawaiiensis CSM005B (Lord Howe I., Australia)
Dumontia contorta GWS001024 (NS, Canada)
Constantinea rosa-marina GWS002650 (AK, USA)
Verlaquea fimbriata GWS037721 (Coccos I., Australia)
Polycoelia harveyana GWS036400 (South Africa)
Polycoelia laciniata GWS001906 (TAS, Australia)
Polycoelia fastigiata GWS015178 (TAS, Australia)
Rhipidomenia polycoelioides GWS015422 (TAS, Australia)
Huonia sandersonii GWS015443 (TAS, Australia)
Kallymeniaceae sp. TNE TNS AL-220767 (Tanegashima I.)
Hommersandia palmatifolia KAM0104 (Russia)
Euthora cristata GWS014757 (MA, USA)
Euthora timburtonii GWS038463 (BC, Canada)
Nothokallymenia sp.2 LLG2876 (France)
Nothokallymenia erosa BDA1997 (Bermuda)
Meredithia nana GWS028859 (SA, Australia)
Meredithia microphylla PC0152257 (France)
Meredithia opuntioides GWS015278 (TAS, Australia)
Psaromenia septentrionalis BDA1993 (Bermuda)
Psaromenia sp.1 LH GWS002058 (Lord Howe I., Australia)
***Psaromenia* sp.2 (*K.* cf. *crassiuscula*) TNS AL-220762 (Tanegashima I.)**
Psaromenia laulamaula BISH 776061 (HI, USA)
Psaromenia sp.1 Jeju GWS018239 (South Korea)
Psaromenia sp.1 (*Kallymenia* cf. *crassiuscula*) TNS AL-181178 (Japan)
***Psaromenia* sp.1 (*K.* cf. *crassiuscula*) TNS AL-209816 (Tanegashima I.)**
Thalassiodianthus incrassatus GWS002056 (Lord Howe I., Australia)
Croisettea pakualapa ARS 09948 (HI, USA)
Croisettea tasmanica GWS015471 (TAS, Australia)
Croisettea australis GWS017390 (VIC, Australia)
Rhytimenia cerebriformis LLG0645 (New Caledonia)
Rhytimenia maculata PERTH 08650748 (WA, Australia)
Velatocarpus pustulosus KAM0102 (Russia)
Kallymeniopsis verrucosa MIMB40742 (Russia)
Erythrophyllum oblongifructum GWS038971 (BC, Canada)
Erythrophyllum wynnei GWS036870 (BC, Canada)
Crossocarpus lamuticus MIMB42671 (Russia)
Erythrophyllum delesserioides GWS021823 (CA, USA)
Thamnophyllis discigera GWS036325 (South Africa)
Thamnophyllis pocockiae GWS036341 (South Africa)
Kallymenia reniformis GWS014632 (Ireland)
Tytthomenia barrettii GWS015505 (TAS, Australia)
Austrokallymenia schizophylla GWS036335 (South Africa)
Austrokallymenia cribrogloea GWS016327 (TAS, Australia)
Austrokallymenia westii BDA1991 (Bermuda)
Austrokallymenia rebeccae GWS002029 (Lord Howe I., Australia)
Austrokallymenia rosea GWS032609 (NSW, Australia)
Austrokallymenia roensis GWS036235 (WA, Australia)
Austrokallymenia sp.2 (*Kallymenia* cf. *sessilis*) TNS AL-200152 (Japan)
Austrokallymenia sp.1 (*Kallymenia* cf. *sessilis*) MAS-00238 (Japan)
***Austrokallymenia* sp.1 (*K.* cf. *sessilis*) TNS AL-209810 (Tanegashima I.)**
***Austrokallymenia* sp.1 (*K.* cf. *sessilis*) TNS AL-213790 (Tanegashima I.)**
Continued

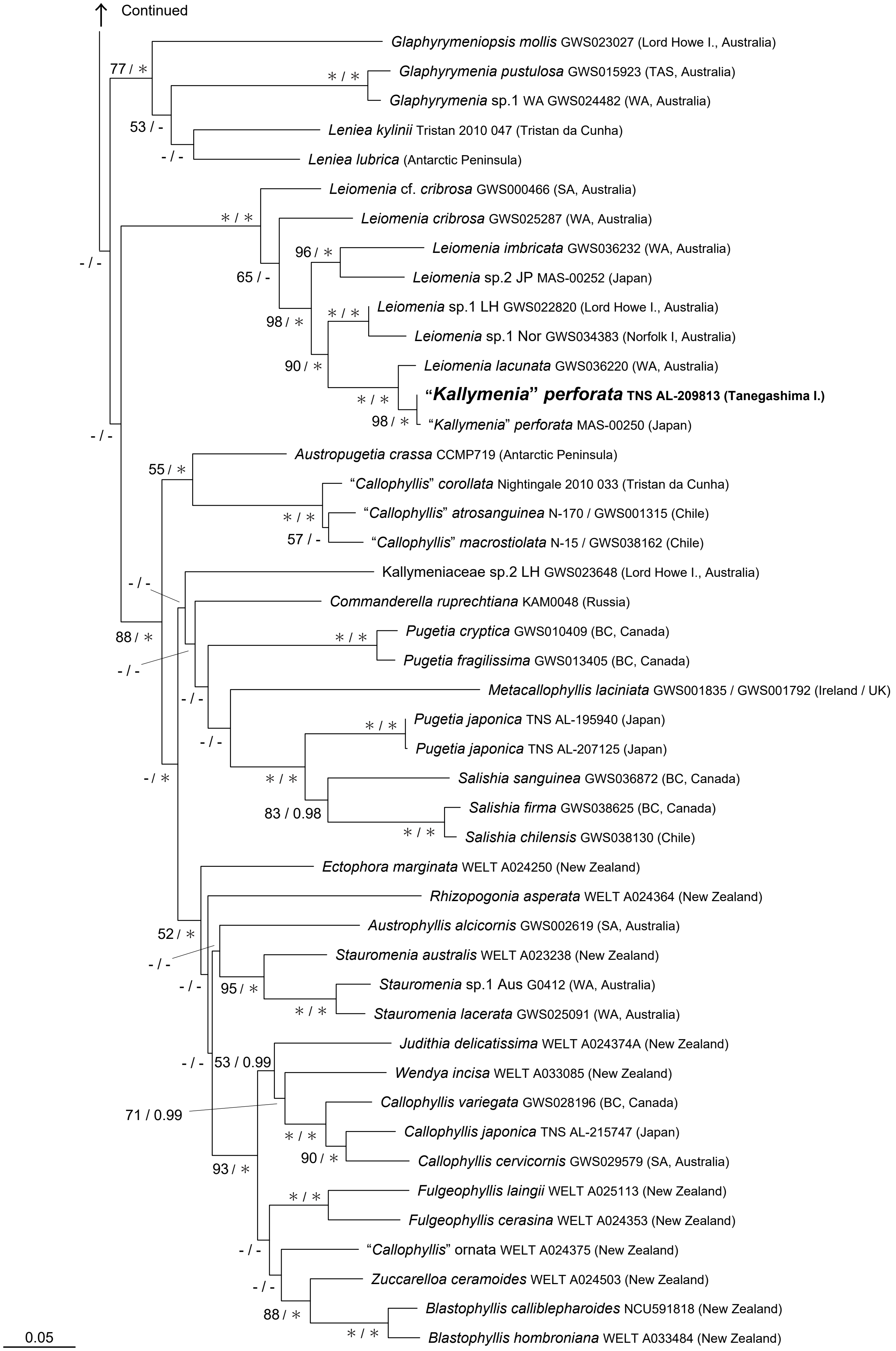

S40 Fig. Maximum likelihood phylogeny for Kallymeniaceae species based on combined *rbc*L and *cox*1 DNA sequences. Values are indicated at the branches: bootstrap (BP; ≥ 50%) and Bayesian posterior probabilities (PP; ≥ 0.95). Asterisks (∗) indicate 100% BP and 1.00 PP.
