# Supplementary material for: DNA-based floristic survey of red algae (Rhodophyta) growing in the mesophotic coral ecosystems (MCEs) offshore of Tanegashima Island, northern Ryukyu Archipelago, Japan
Source: PLoS One. 2025 Mar 10;20(3):e0316067. doi: 10.1371/journal.pone.0316067 (PMC11893125; doi:10.1371/journal.pone.0316067)
Supplement: S5 File — Maximum likelihood phylogeny of red algae collected from offshore Tanegashima Island. (ZIP) [file pone.0316067.s005.zip › S5_File/S43_Fig.pdf]

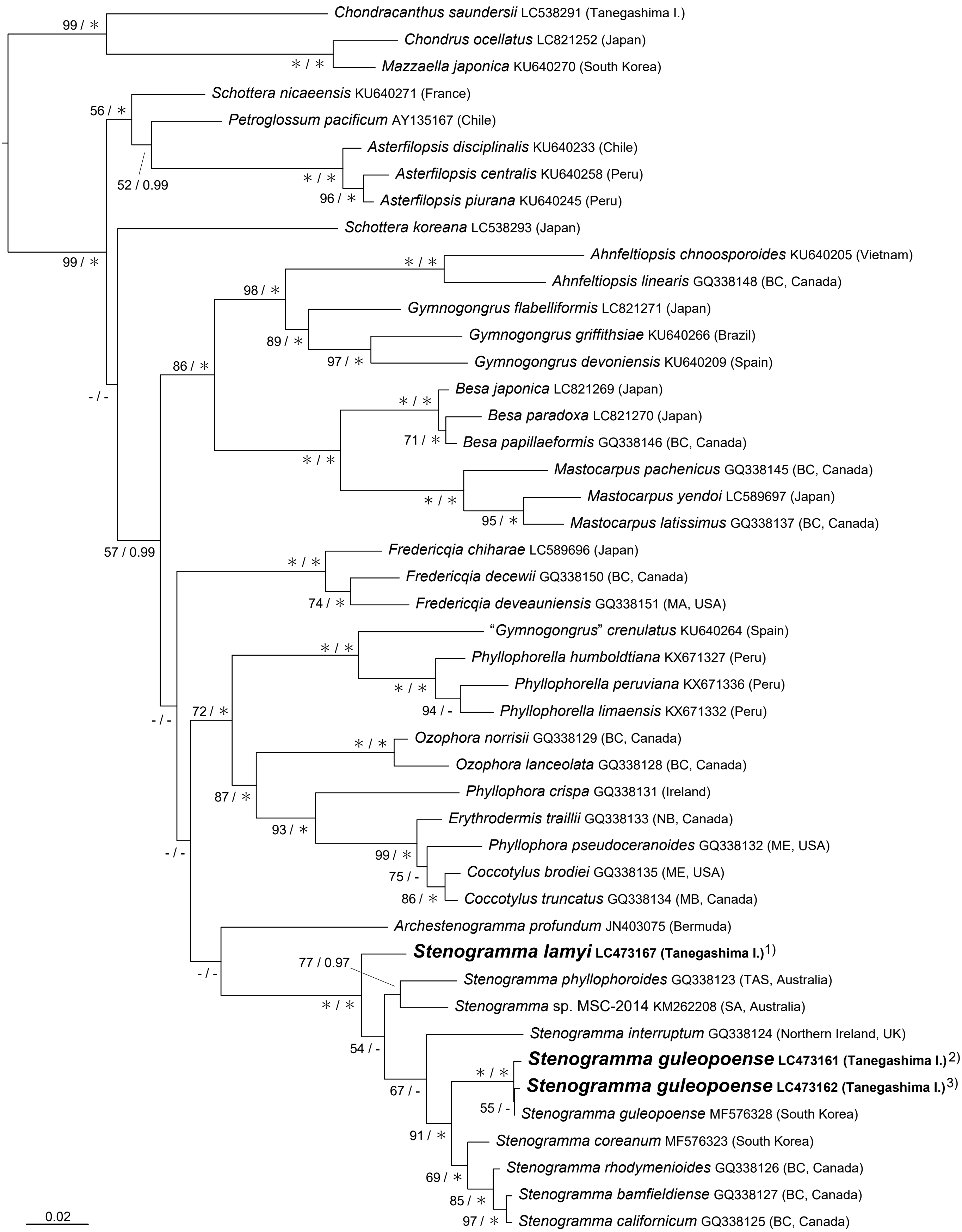

1) KR733113 (Madagascar) *Holotype, LC821272-LC821276 (Tanegashima I.) had identical sequences. 2) LC473163, LC473164 (Tanegashima I.) had identical sequences. 3) LC473165 (Tanegashima I.) had identical sequence.

S43A Fig. Maximum likelihood phylogeny for Phyllophoraceae species based on *rbc*L DNA sequences. Values are indicated at the branches: bootstrap (BP; ≥ 50%) and Bayesian posterior probabilities (PP; ≥ 0.95). Asterisks (∗) indicate 100% BP and 1.00 PP.

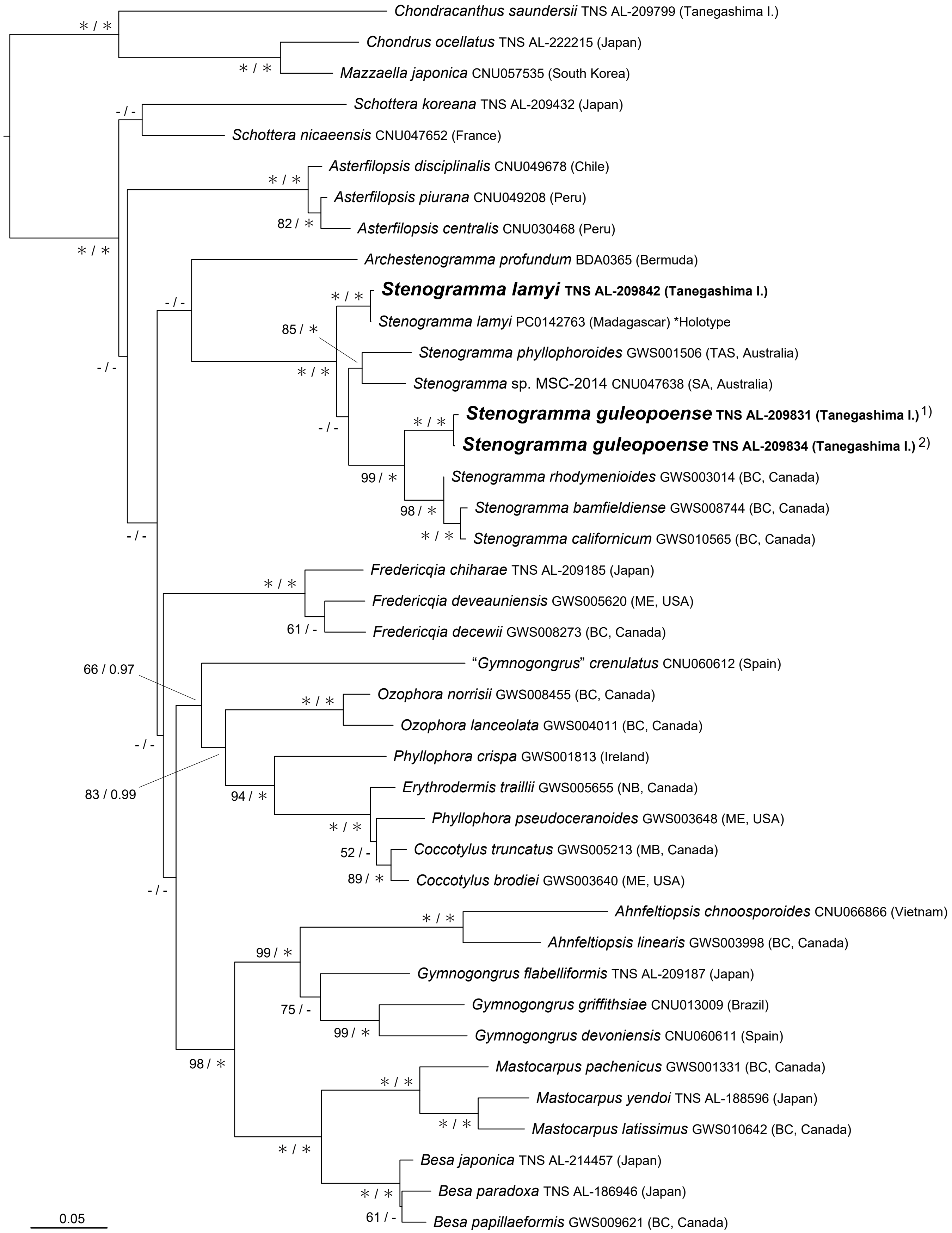

[1)]TNS AL-209835, 209830 (Tanegashima I.) had identical sequences. [2)]TNS AL-209832 (Tanegashima I.) had identical sequence.

S43B Fig. Maximum likelihood phylogeny for Phyllophoraceae species based on combined *rbc*L and *cox*1 DNA sequences. Values are indicated at the branches: bootstrap (BP; ≥ 50%) and Bayesian posterior probabilities (PP; ≥ 0.95). Asterisks (∗) indicate 100% BP and 1.00 PP.
