# Supplementary material for: DNA-based floristic survey of red algae (Rhodophyta) growing in the mesophotic coral ecosystems (MCEs) offshore of Tanegashima Island, northern Ryukyu Archipelago, Japan
Source: PLoS One. 2025 Mar 10;20(3):e0316067. doi: 10.1371/journal.pone.0316067 (PMC11893125; doi:10.1371/journal.pone.0316067)
Supplement: S5 File — Maximum likelihood phylogeny of red algae collected from offshore Tanegashima Island. (ZIP) [file pone.0316067.s005.zip › S5_File/S45_Fig.pdf]

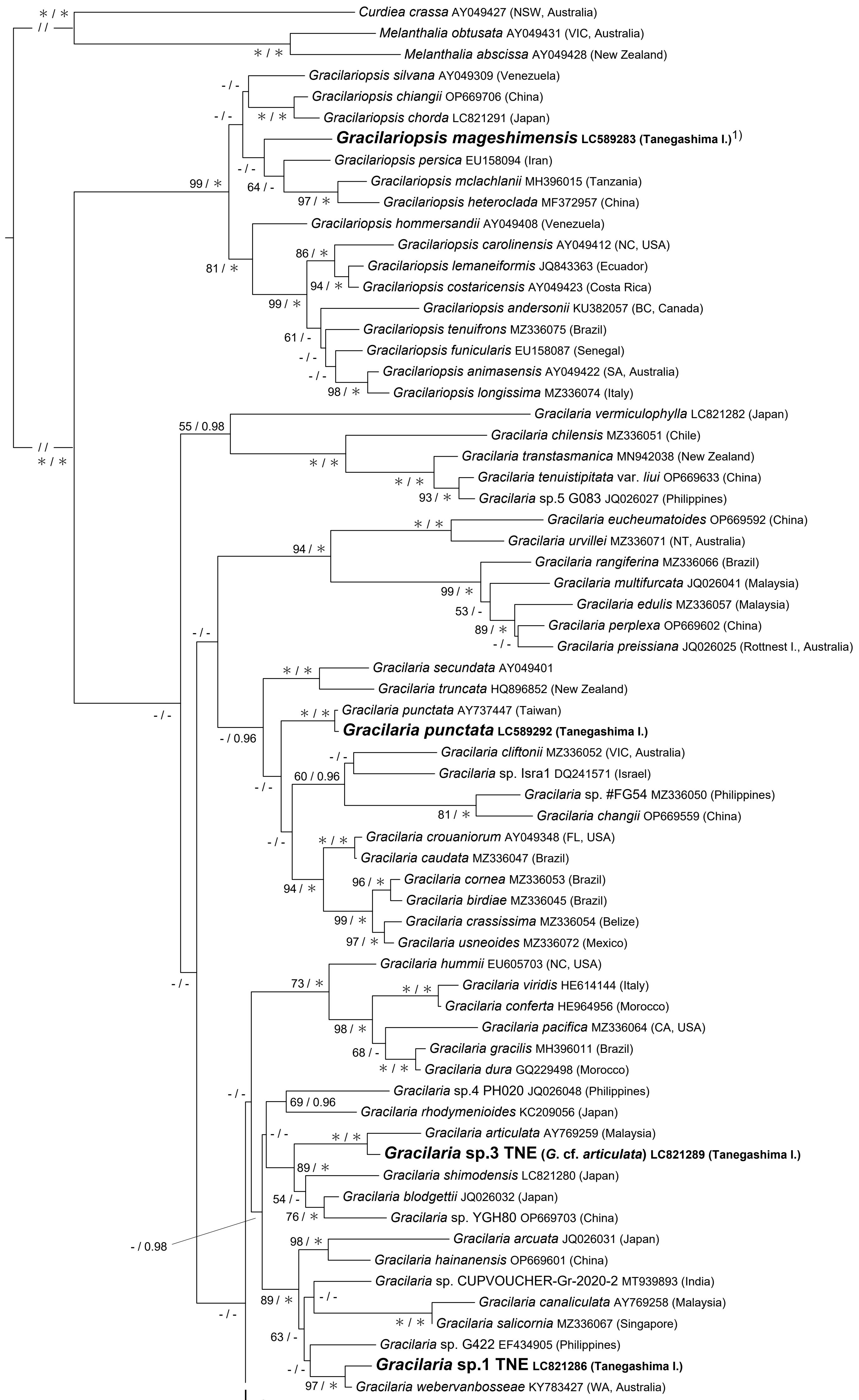
Curdiea crassa AY049427 (NSW, Australia)
Melanthalia obtusata AY049431 (VIC, Australia)
Melanthalia abscissa AY049428 (New Zealand)
Gracilariopsis silvana AY049309 (Venezuela)
Gracilariopsis chiangii OP669706 (China)
Gracilariopsis chorda LC821291 (Japan)
Gracilariopsis mageshimensis LC589283 (Tanegashima I.)1)
Gracilariopsis persica EU158094 (Iran)
Gracilariopsis mclachlanii MH396015 (Tanzania)
Gracilariopsis heteroclada MF372957 (China)
Gracilariopsis hommersandii AY049408 (Venezuela)
Gracilariopsis carolinensis AY049412 (NC, USA)
Gracilariopsis lemaneiformis JQ843363 (Ecuador)
Gracilariopsis costaricensis AY049423 (Costa Rica)
Gracilariopsis andersonii KU382057 (BC, Canada)
Gracilariopsis tenuifrons MZ336075 (Brazil)
Gracilariopsis funicularis EU158087 (Senegal)
Gracilariopsis animasensis AY049422 (SA, Australia)
Gracilariopsis longissima MZ336074 (Italy)
Gracilaria vermiculophylla LC821282 (Japan)
Gracilaria chilensis MZ336051 (Chile)
Gracilaria transtasmanica MN942038 (New Zealand)
Gracilaria tenuistipitata var. liui OP669633 (China)
Gracilaria sp.5 G083 JQ026027 (Philippines)
Gracilaria eucheumatoides OP669592 (China)
Gracilaria urvillei MZ336071 (NT, Australia)
Gracilaria rangiferina MZ336066 (Brazil)
Gracilaria multifurcata JQ026041 (Malaysia)
Gracilaria edulis MZ336057 (Malaysia)
Gracilaria perplexa OP669602 (China)
Gracilaria preissiana JQ026025 (Rottnest I., Australia)
Gracilaria secundata AY049401
Gracilaria truncata HQ896852 (New Zealand)
Gracilaria punctata AY737447 (Taiwan)
Gracilaria punctata LC589292 (Tanegashima I.)
Gracilaria cliftonii MZ336052 (VIC, Australia)
Gracilaria sp. Isra1 DQ241571 (Israel)
Gracilaria sp. #FG54 MZ336050 (Philippines)
Gracilaria changii OP669559 (China)
Gracilaria crouaniorum AY049348 (FL, USA)
Gracilaria caudata MZ336047 (Brazil)
Gracilaria cornea MZ336053 (Brazil)
Gracilaria birdiae MZ336045 (Brazil)
Gracilaria crassissima MZ336054 (Belize)
Gracilaria usneoides MZ336072 (Mexico)
Gracilaria hummii EU605703 (NC, USA)
Gracilaria viridis HE614144 (Italy)
Gracilaria conferta HE964956 (Morocco)
Gracilaria pacifica MZ336064 (CA, USA)
Gracilaria gracilis MH396011 (Brazil)
Gracilaria dura GQ229498 (Morocco)
Gracilaria sp.4 PH020 JQ026048 (Philippines)
Gracilaria rhodymenioides KC209056 (Japan)
Gracilaria articulata AY769259 (Malaysia)
Gracilaria sp.3 TNE (G. cf. articulata) LC821289 (Tanegashima I.)
Gracilaria shimodensis LC821280 (Japan)
Gracilaria blodgettii JQ026032 (Japan)
Gracilaria sp. YGH80 OP669703 (China)
Gracilaria arcuata JQ026031 (Japan)
Gracilaria hainanensis OP669601 (China)
Gracilaria sp. CUPVOUCHER-Gr-2020-2 MT939893 (India)
Gracilaria canaliculata AY769258 (Malaysia)
Gracilaria salicornia MZ336067 (Singapore)
Gracilaria sp. G422 EF434905 (Philippines)
Gracilaria sp.1 TNE LC821286 (Tanegashima I.)
Gracilaria webervanbosseae KY783427 (WA, Australia)

↓ Continued

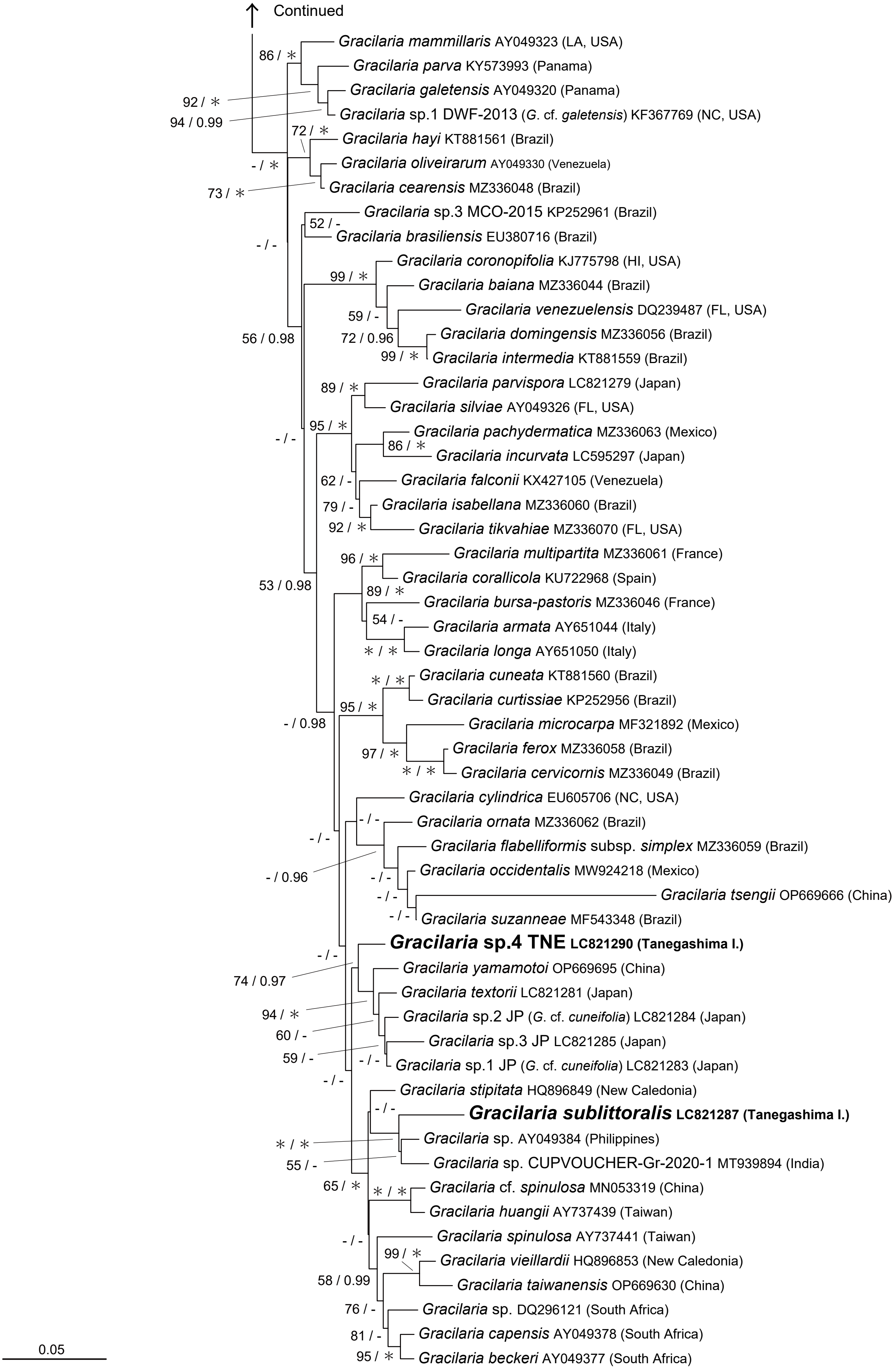

[1)]LC589284–LC589289, LC821292–LC821295 (Tanegashima I.) had identical sequences.

S45A Fig. Maximum likelihood phylogeny for Gracilarioideae species based on *rbc*L DNA sequences. Values are indicated at the branches: bootstrap (BP; ≥ 50%) and Bayesian posterior probabilities (PP; ≥ 0.95). Asterisks (*) indicate 100% BP and 1.00 PP.

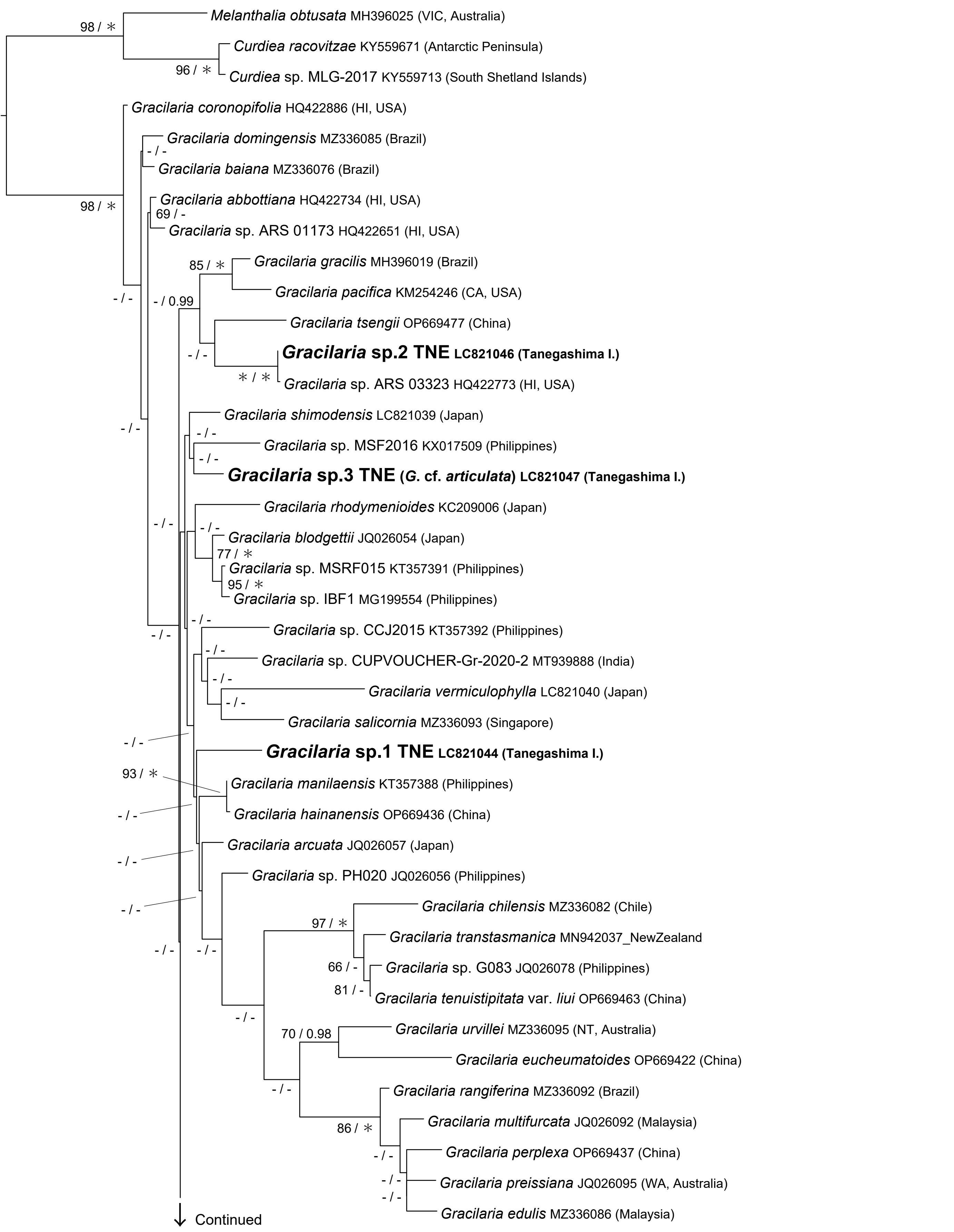

Melanthalia obtusata MH396025 (VIC, Australia)
98 / ∗
Curdiea racovitzae KY559671 (Antarctic Peninsula)
96 / ∗
Curdiea sp. MLG-2017 KY559713 (South Shetland Islands)
Gracilaria coronopifolia HQ422886 (HI, USA)
Gracilaria domingensis MZ336085 (Brazil)
- / -
Gracilaria baiana MZ336076 (Brazil)
98 / ∗
Gracilaria abbottiana HQ422734 (HI, USA)
69 / -
Gracilaria sp. ARS 01173 HQ422651 (HI, USA)
85 / ∗
Gracilaria gracilis MH396019 (Brazil)
Gracilaria pacifica KM254246 (CA, USA)
- / -
- / 0.99
Gracilaria tsengii OP669477 (China)
- / -
Gracilaria sp.2 TNE LC821046 (Tanegashima I.)
∗ / ∗
Gracilaria sp. ARS 03323 HQ422773 (HI, USA)
Gracilaria shimodensis LC821039 (Japan)
- / -
- / -
Gracilaria sp. MSF2016 KX017509 (Philippines)
- / -
Gracilaria sp.3 TNE (G. cf. articulata) LC821047 (Tanegashima I.)
Gracilaria rhodymenioides KC209006 (Japan)
- / -
- / -
Gracilaria blodgettii JQ026054 (Japan)
77 / ∗
Gracilaria sp. MSRF015 KT357391 (Philippines)
95 / ∗
Gracilaria sp. IBF1 MG199554 (Philippines)
- / -
Gracilaria sp. CCJ2015 KT357392 (Philippines)
- / -
- / -
Gracilaria sp. CUPVOUCHER-Gr-2020-2 MT939888 (India)
- / -
Gracilaria vermiculophylla LC821040 (Japan)
- / -
Gracilaria salicornia MZ336093 (Singapore)
- / -
Gracilaria sp.1 TNE LC821044 (Tanegashima I.)
93 / ∗
Gracilaria manilaensis KT357388 (Philippines)
- / -
Gracilaria hainanensis OP669436 (China)
- / -
Gracilaria arcuata JQ026057 (Japan)
- / -
Gracilaria sp. PH020 JQ026056 (Philippines)
Gracilaria chilensis MZ336082 (Chile)
97 / ∗
Gracilaria transtasmanica MN942037_NewZealand
- / -
- / -
66 / -
Gracilaria sp. G083 JQ026078 (Philippines)
81 / -
Gracilaria tenuistipitata var. liui OP669463 (China)
- / -
70 / 0.98
Gracilaria urvillei MZ336095 (NT, Australia)
Gracilaria eucheumatoides OP669422 (China)
- / -
Gracilaria rangiferina MZ336092 (Brazil)
Gracilaria multifurcata JQ026092 (Malaysia)
86 / ∗
- / -
Gracilaria perplexa OP669437 (China)
- / -
Gracilaria preissiana JQ026095 (WA, Australia)
- / -
Gracilaria edulis MZ336086 (Malaysia)
Continued

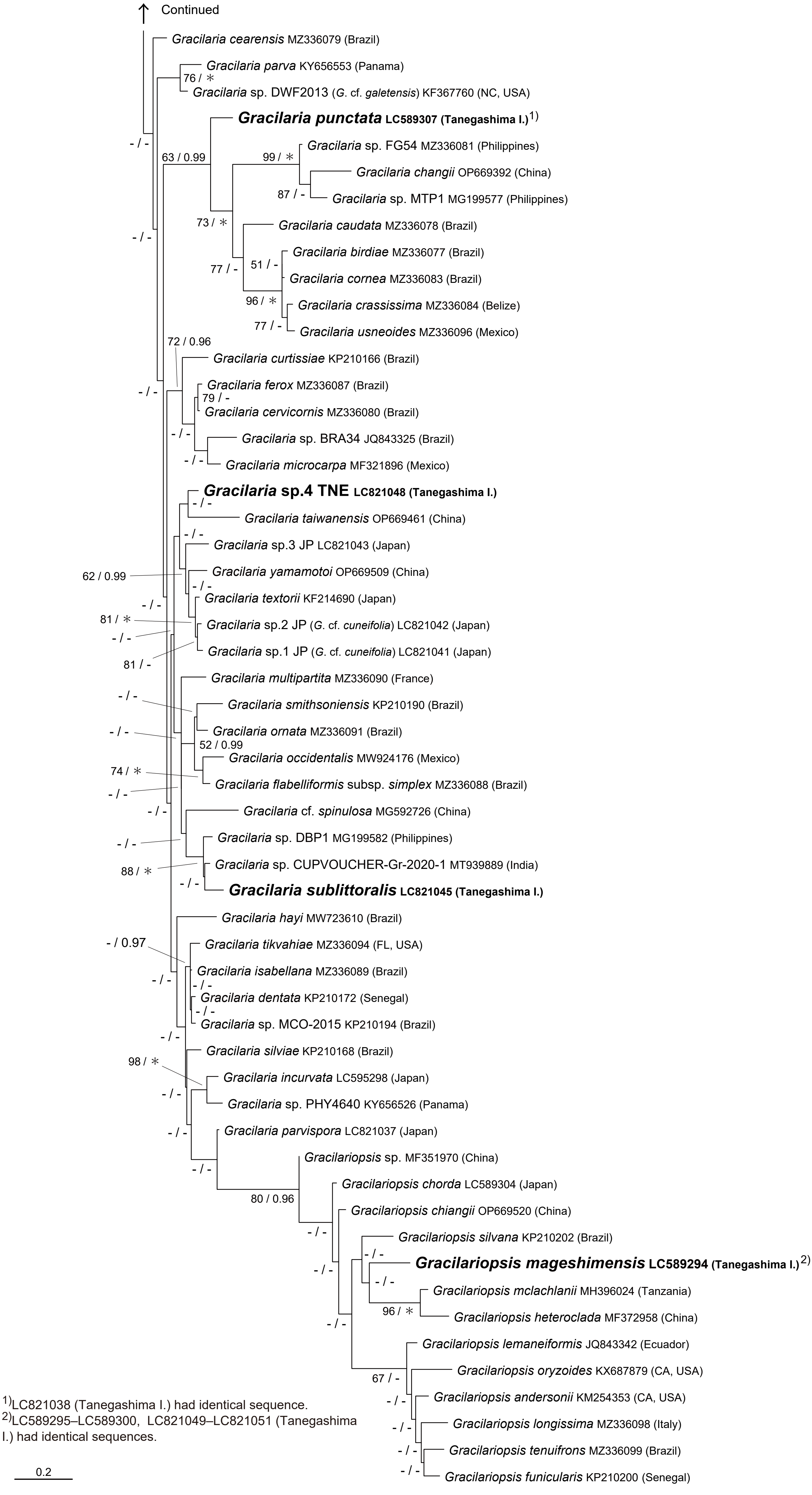

S45B Fig. Maximum likelihood phylogeny for Gracilarioideae species based on *cox*1 DNA sequences. Values are indicated at the branches: bootstrap (BP; ≥ 50%) and Bayesian posterior probabilities (PP; ≥ 0.95). Asterisks (∗) indicate 100% BP and 1.00 PP.

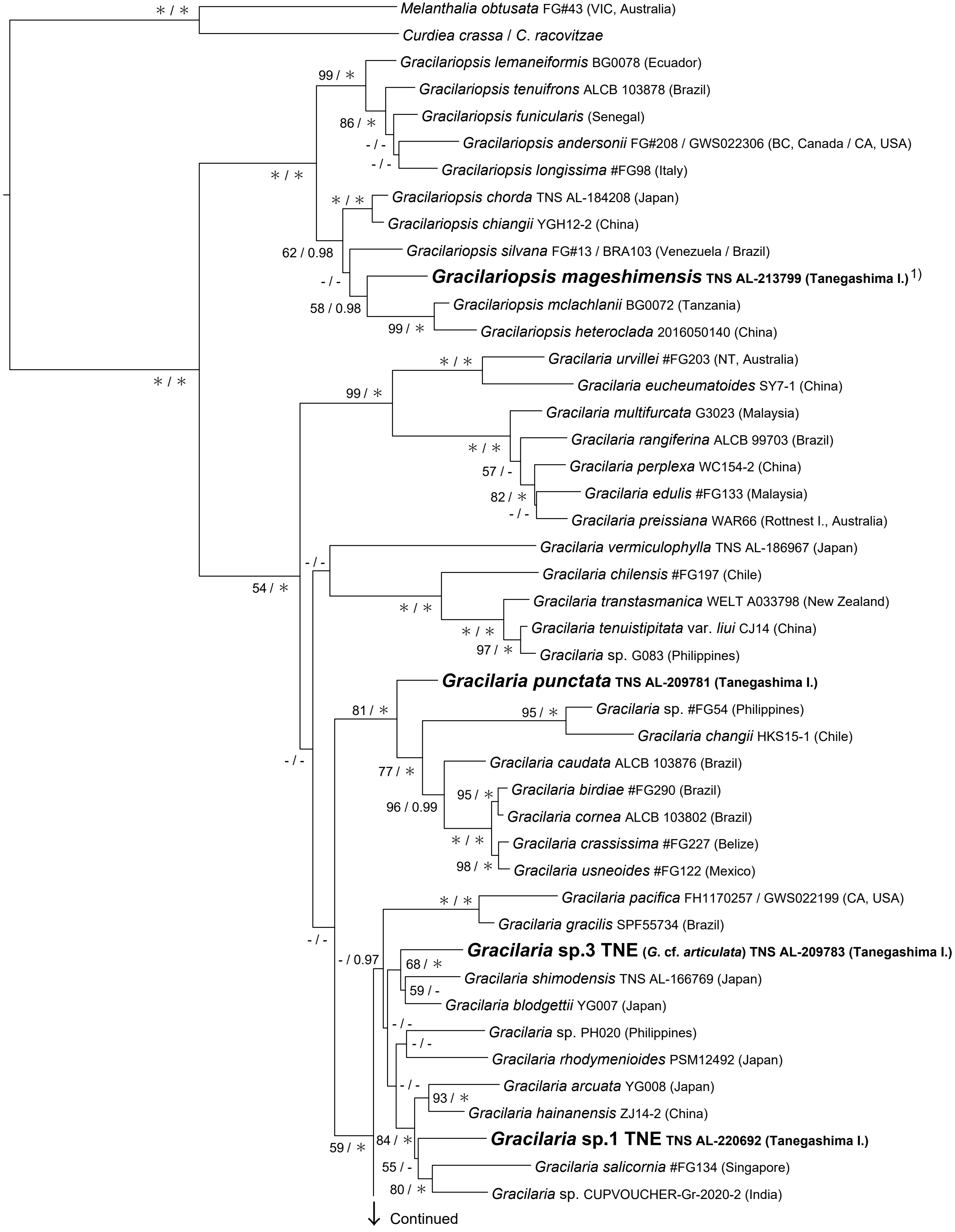

/
Melanthalia obtusata FG#43 (VIC, Australia)
Curdiea crassa / C. racovitzae
Gracilariopsis lemaneiformis BG0078 (Ecuador)
99 / *
Gracilariopsis tenuifrons ALCB 103878 (Brazil)
Gracilariopsis funicularis (Senegal)
86 / *
Gracilariopsis andersonii FG#208 / GWS022306 (BC, Canada / CA, USA)
- / -
- / -
Gracilariopsis longissima #FG98 (Italy)
* / *
Gracilariopsis chorda TNS AL-184208 (Japan)
* / *
Gracilariopsis chiangii YGH12-2 (China)
62 / 0.98
Gracilariopsis silvana FG#13 / BRA103 (Venezuela / Brazil)
- / -
Gracilariopsis mageshimensis TNS AL-213799 (Tanegashima I.) 1)
58 / 0.98
Gracilariopsis mclachlanii BG0072 (Tanzania)
99 / *
Gracilariopsis heteroclada 2016050140 (China)
* / *
* / *
Gracilaria urvillei #FG203 (NT, Australia)
Gracilaria eucheumatoides SY7-1 (China)
99 / *
Gracilaria multifurcata G3023 (Malaysia)
Gracilaria rangiferina ALCB 99703 (Brazil)
* / *
Gracilaria perplexa WC154-2 (China)
57 / -
82 / *
Gracilaria edulis #FG133 (Malaysia)
- / -
Gracilaria preissiana WAR66 (Rottnest I., Australia)
Gracilaria vermiculophylla TNS AL-186967 (Japan)
- / -
Gracilaria chilensis #FG197 (Chile)
54 / *
* / *
Gracilaria transtasmanica WELT A033798 (New Zealand)
* / *
Gracilaria tenuistipitata var. liui CJ14 (China)
97 / *
Gracilaria sp. G083 (Philippines)
Gracilaria punctata TNS AL-209781 (Tanegashima I.)
81 / *
95 / *
Gracilaria sp. #FG54 (Philippines)
Gracilaria changii HKS15-1 (Chile)
- / -
Gracilaria caudata ALCB 103876 (Brazil)
77 / *
95 / *
Gracilaria birdiae #FG290 (Brazil)
96 / 0.99
Gracilaria cornea ALCB 103802 (Brazil)
* / *
Gracilaria crassissima #FG227 (Belize)
98 / *
Gracilaria usneoides #FG122 (Mexico)
* / *
Gracilaria pacifica FH1170257 / GWS022199 (CA, USA)
Gracilaria gracilis SPF55734 (Brazil)
- / -
- / 0.97
Gracilaria sp.3 TNE (G. cf. articulata) TNS AL-209783 (Tanegashima I.)
68 / *
Gracilaria shimodensis TNS AL-166769 (Japan)
59 / -
Gracilaria blodgettii YG007 (Japan)
- / -
Gracilaria sp. PH020 (Philippines)
- / -
Gracilaria rhodymenioides PSM12492 (Japan)
- / -
Gracilaria arcuata YG008 (Japan)
93 / *
Gracilaria hainanensis ZJ14-2 (China)
84 / *
Gracilaria sp.1 TNE TNS AL-220692 (Tanegashima I.)
59 / *
55 / -
Gracilaria salicornia #FG134 (Singapore)
80 / *
Gracilaria sp. CUPVOUCHER-Gr-2020-2 (India)
↓ Continued

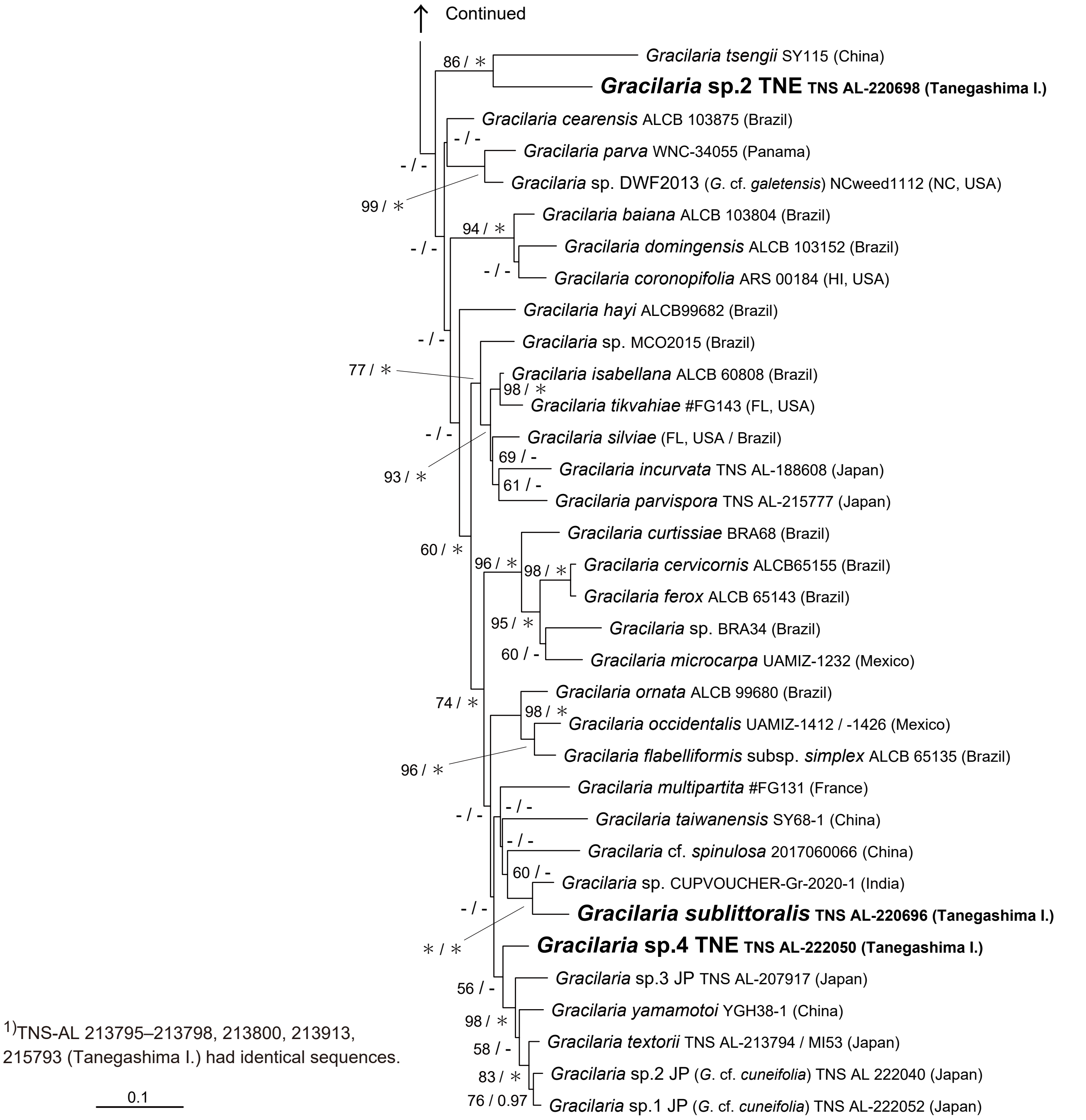

S45C Fig. Maximum likelihood phylogeny for Gracilarioideae species based on combined *rbc*L and *cox*1 DNA sequences. Values are indicated at the branches: bootstrap (BP; ≥ 50%) and Bayesian posterior probabilities (PP; ≥ 0.95). Asterisks (∗) indicate 100% BP and 1.00 PP.
