# Supplementary material for: DNA-based floristic survey of red algae (Rhodophyta) growing in the mesophotic coral ecosystems (MCEs) offshore of Tanegashima Island, northern Ryukyu Archipelago, Japan
Source: PLoS One. 2025 Mar 10;20(3):e0316067. doi: 10.1371/journal.pone.0316067 (PMC11893125; doi:10.1371/journal.pone.0316067)
Supplement: S5 File — Maximum likelihood phylogeny of red algae collected from offshore Tanegashima Island. (ZIP) [file pone.0316067.s005.zip › S5_File/S47_Fig.pdf]

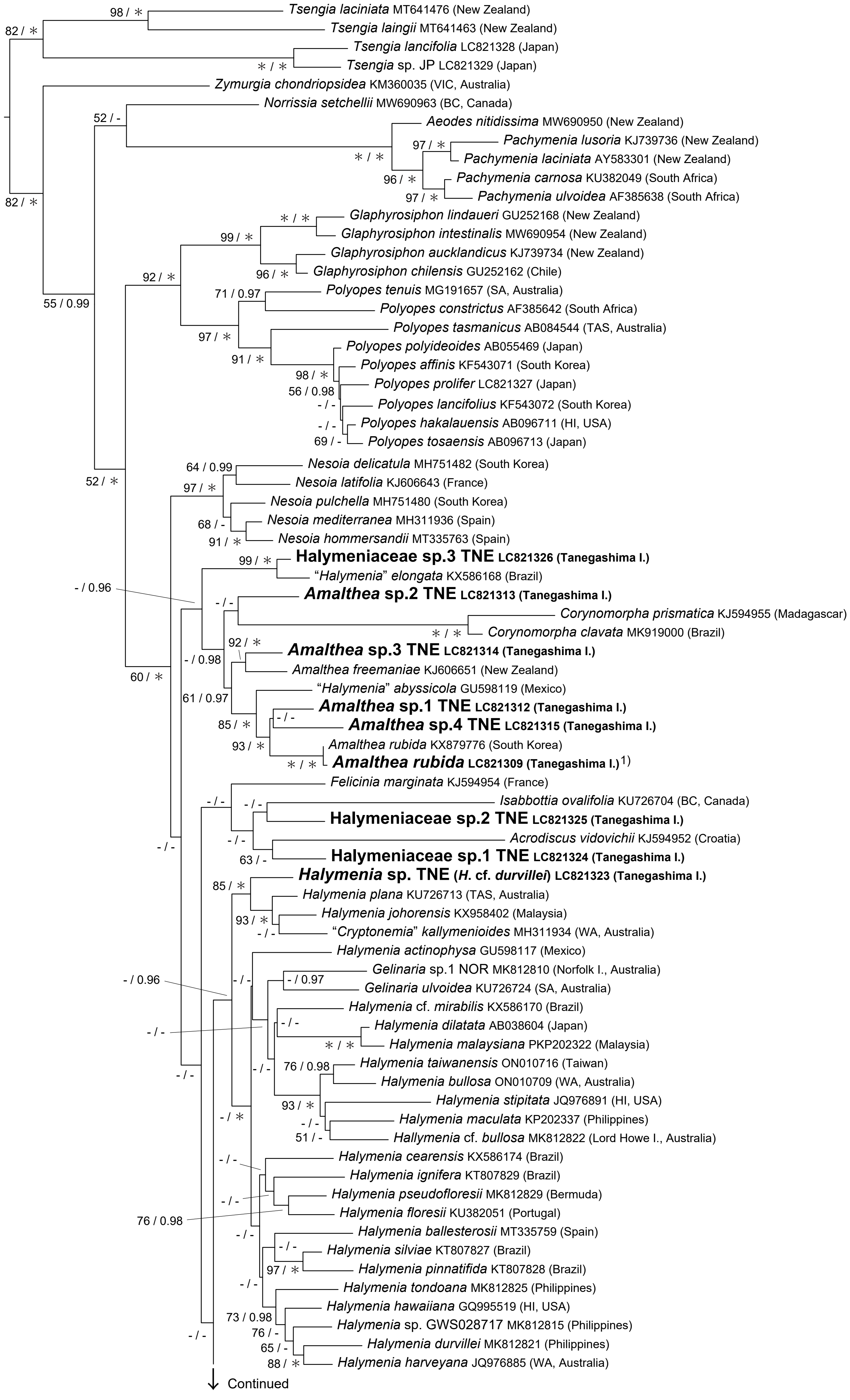

Tsengia laciniata MT641476 (New Zealand)
Tsengia laingii MT641463 (New Zealand)
Tsengia lancifolia LC821328 (Japan)
Tsengia sp. JP LC821329 (Japan)
Zymurgia chondriopsidea KM360035 (VIC, Australia)
Norrissia setchellii MW690963 (BC, Canada)
Aeodes nitidissima MW690950 (New Zealand)
Pachymenia lusoria KJ739736 (New Zealand)
Pachymenia laciniata AY583301 (New Zealand)
Pachymenia carnosa KU382049 (South Africa)
Pachymenia ulvoidea AF385638 (South Africa)
Glaphyrosiphon lindaueri GU252168 (New Zealand)
Glaphyrosiphon intestinalis MW690954 (New Zealand)
Glaphyrosiphon aucklandicus KJ739734 (New Zealand)
Glaphyrosiphon chilensis GU252162 (Chile)
Polyopes tenuis MG191657 (SA, Australia)
Polyopes constrictus AF385642 (South Africa)
Polyopes tasmanicus AB084544 (TAS, Australia)
Polyopes polyideoides AB055469 (Japan)
Polyopes affinis KF543071 (South Korea)
Polyopes prolifer LC821327 (Japan)
Polyopes lancifolius KF543072 (South Korea)
Polyopes hakalauensis AB096711 (HI, USA)
Polyopes tosaensis AB096713 (Japan)
Nesoia delicatula MH751482 (South Korea)
Nesoia latifolia KJ606643 (France)
Nesoia pulchella MH751480 (South Korea)
Nesoia mediterranea MH311936 (Spain)
Nesoia hommersandii MT335763 (Spain)
Halymeniaceae sp.3 TNE LC821326 (Tanegashima I.)
"Halymenia" elongata KX586168 (Brazil)
Amalthea sp.2 TNE LC821313 (Tanegashima I.)
Corynomorpha prismatica KJ594955 (Madagascar)
Corynomorpha clavata MK919000 (Brazil)
Amalthea sp.3 TNE LC821314 (Tanegashima I.)
Amalthea freemaniae KJ606651 (New Zealand)
"Halymenia" abyssicola GU598119 (Mexico)
Amalthea sp.1 TNE LC821312 (Tanegashima I.)
Amalthea sp.4 TNE LC821315 (Tanegashima I.)
Amalthea rubida KX879776 (South Korea)
Amalthea rubida LC821309 (Tanegashima I.)1)
Felicinia marginata KJ594954 (France)
Isabbottia ovalifolia KU726704 (BC, Canada)
Halymeniaceae sp.2 TNE LC821325 (Tanegashima I.)
Acrodiscus vidovichii KJ594952 (Croatia)
Halymeniaceae sp.1 TNE LC821324 (Tanegashima I.)
Halymenia sp. TNE (H. cf. durvillei) LC821323 (Tanegashima I.)
Halymenia plana KU726713 (TAS, Australia)
Halymenia johorensis KX958402 (Malaysia)
"Cryptonemia" kallymenioides MH311934 (WA, Australia)
Halymenia actinophysa GU598117 (Mexico)
Gelinaria sp.1 NOR MK812810 (Norfolk I., Australia)
Gelinaria ulvoidea KU726724 (SA, Australia)
Halymenia cf. mirabilis KX586170 (Brazil)
Halymenia dilatata AB038604 (Japan)
Halymenia malaysiana PKP202322 (Malaysia)
Halymenia taiwanensis ON010716 (Taiwan)
Halymenia bullosa ON010709 (WA, Australia)
Halymenia stipitata JQ976891 (HI, USA)
Halymenia maculata KP202337 (Philippines)
Hallymenia cf. bullosa MK812822 (Lord Howe I., Australia)
Halymenia cearensis KX586174 (Brazil)
Halymenia ignifera KT807829 (Brazil)
Halymenia pseudofloresii MK812829 (Bermuda)
Halymenia floresii KU382051 (Portugal)
Halymenia ballesterosii MT335759 (Spain)
Halymenia silviae KT807827 (Brazil)
Halymenia pinnatifida KT807828 (Brazil)
Halymenia tondoana MK812825 (Philippines)
Halymenia hawaiiana GQ995519 (HI, USA)
Halymenia sp. GWS028717 MK812815 (Philippines)
Halymenia durvillei MK812821 (Philippines)
Halymenia harveyana JQ976885 (WA, Australia)
Continued

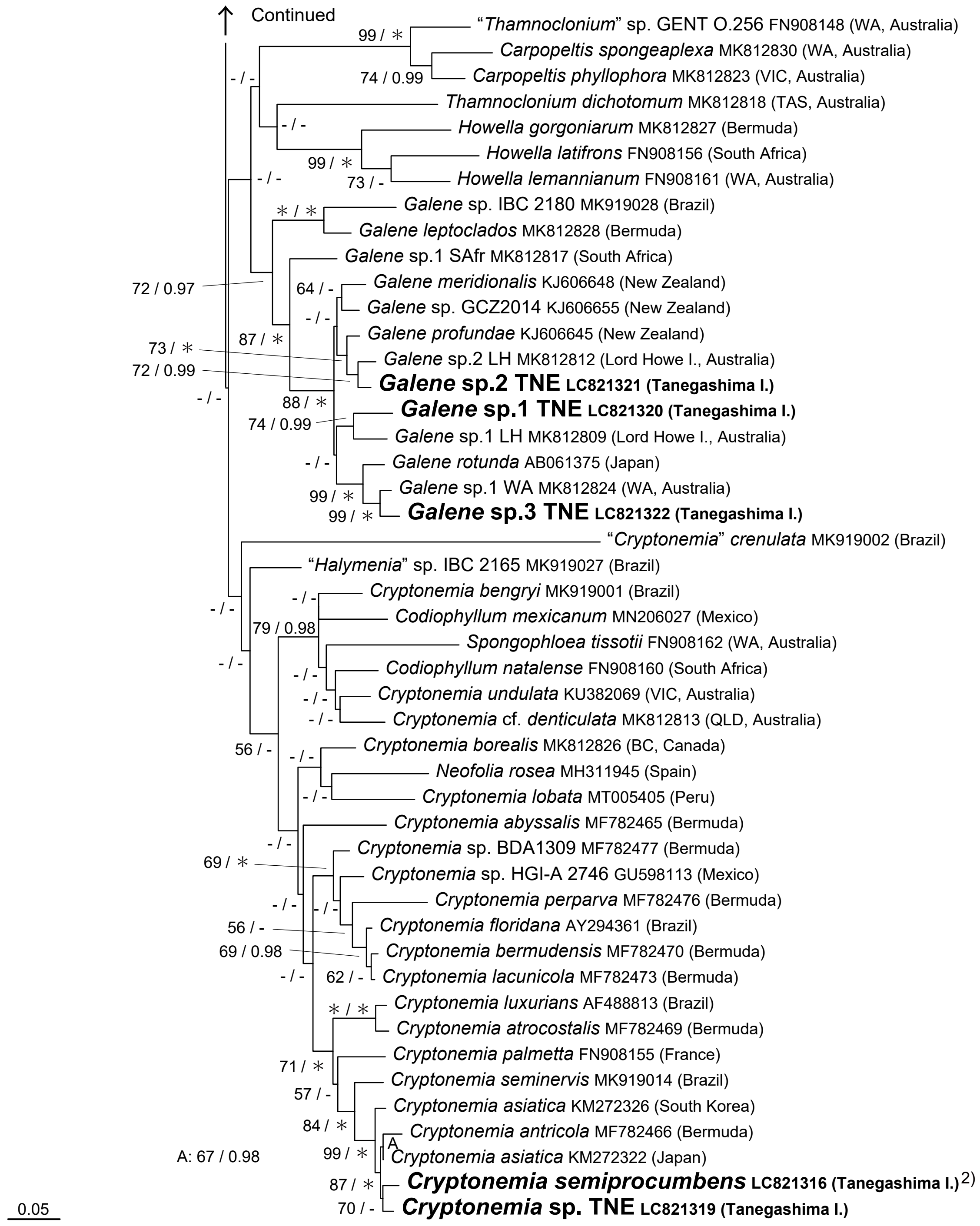

[1)]LC821310, LC821311 (Tanegashima I.) had identical sequences. [2)]LC821317, LC821318 (Tanegashima I.) had identical sequences.

S47A Fig. Maximum likelihood phylogeny for Halymeniaceae species based on *rbc*L DNA sequences. Values are indicated at the branches: bootstrap (BP; ≥ 50%) and Bayesian posterior probabilities (PP; ≥ 0.95). Asterisks (∗) indicate 100% BP and 1.00 PP.

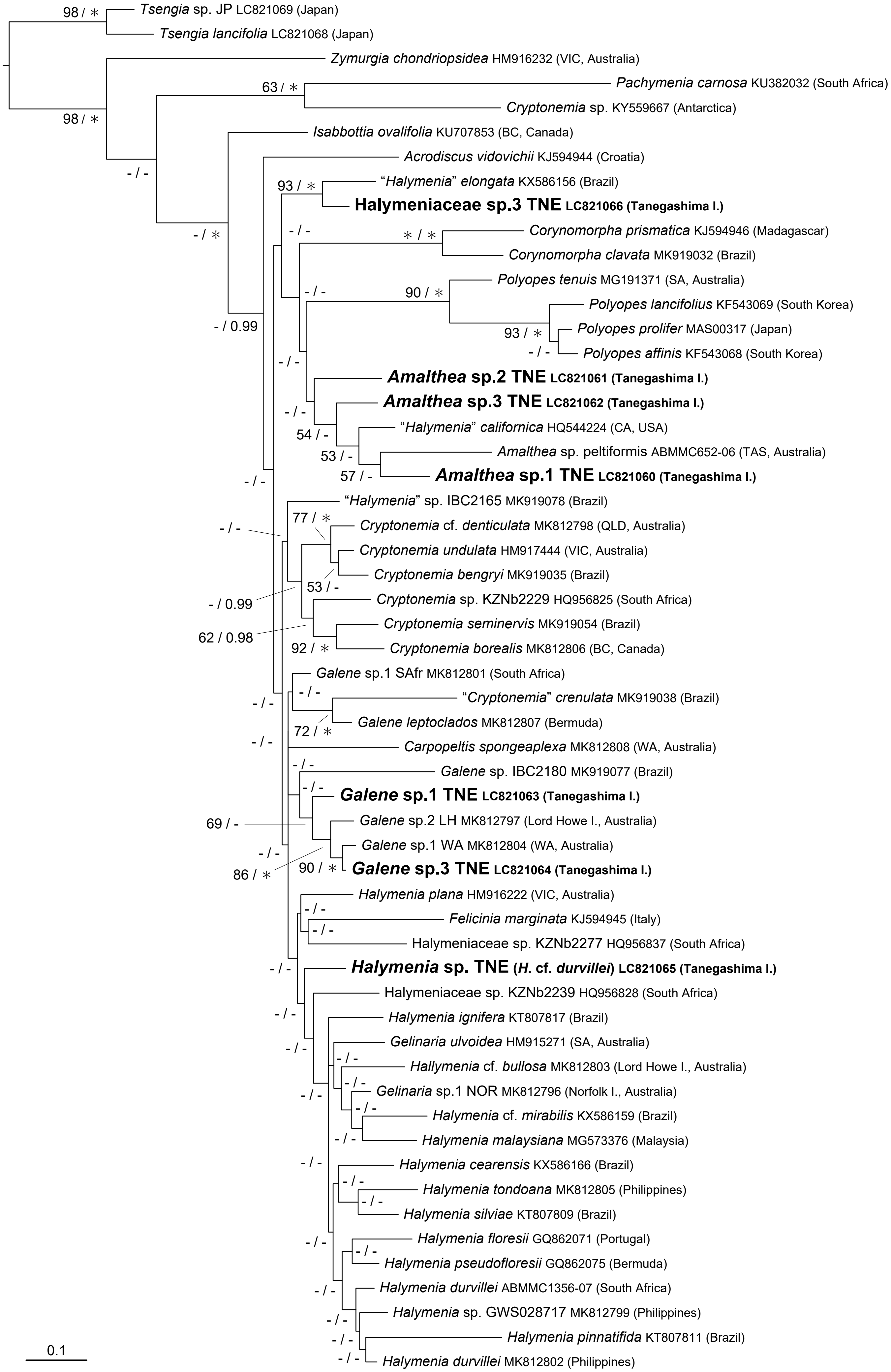

S47B Fig. Maximum likelihood phylogeny for Halymeniaceae species based on *cox*1 DNA sequences.
Values are indicated at the branches: bootstrap (BP; ≥ 50%) and Bayesian posterior probabilities (PP; ≥ 0.95). Asterisks (∗) indicate 100% BP and 1.00 PP.

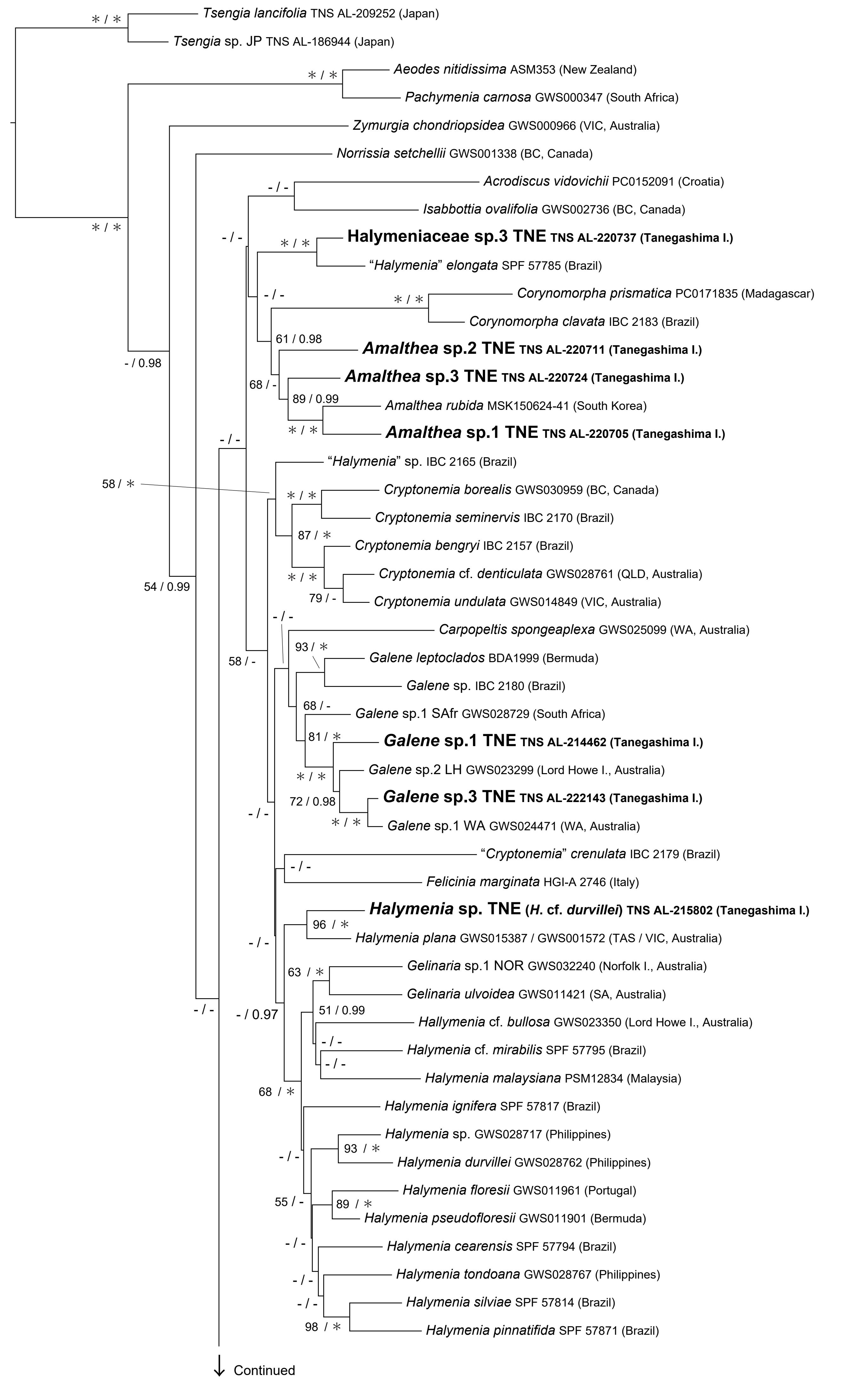

Tsengia lancifolia TNS AL-209252 (Japan)
Tsengia sp. JP TNS AL-186944 (Japan)
Aeodes nitidissima ASM353 (New Zealand)
Pachymenia carnosa GWS000347 (South Africa)
Zymurgia chondriopsidea GWS000966 (VIC, Australia)
Norrissia setchellii GWS001338 (BC, Canada)
Acrodiscus vidovichii PC0152091 (Croatia)
Isabbottia ovalifolia GWS002736 (BC, Canada)
Halymeniaceae sp.3 TNE TNS AL-220737 (Tanegashima I.)
"Halymenia" elongata SPF 57785 (Brazil)
Corynomorpha prismatica PC0171835 (Madagascar)
Corynomorpha clavata IBC 2183 (Brazil)
Amalthea sp.2 TNE TNS AL-220711 (Tanegashima I.)
Amalthea sp.3 TNE TNS AL-220724 (Tanegashima I.)
Amalthea rubida MSK150624-41 (South Korea)
Amalthea sp.1 TNE TNS AL-220705 (Tanegashima I.)
"Halymenia" sp. IBC 2165 (Brazil)
Cryptonemia borealis GWS030959 (BC, Canada)
Cryptonemia seminervis IBC 2170 (Brazil)
Cryptonemia bengryi IBC 2157 (Brazil)
Cryptonemia cf. denticulata GWS028761 (QLD, Australia)
Cryptonemia undulata GWS014849 (VIC, Australia)
Carpopeltis spongeaplexa GWS025099 (WA, Australia)
Galene leptoclados BDA1999 (Bermuda)
Galene sp. IBC 2180 (Brazil)
Galene sp.1 SAfr GWS028729 (South Africa)
Galene sp.1 TNE TNS AL-214462 (Tanegashima I.)
Galene sp.2 LH GWS023299 (Lord Howe I., Australia)
Galene sp.3 TNE TNS AL-222143 (Tanegashima I.)
Galene sp.1 WA GWS024471 (WA, Australia)
"Cryptonemia" crenulata IBC 2179 (Brazil)
Felicinia marginata HGI-A 2746 (Italy)
Halymenia sp. TNE (H. cf. durvillei) TNS AL-215802 (Tanegashima I.)
Halymenia plana GWS015387 / GWS001572 (TAS / VIC, Australia)
Gelinaria sp.1 NOR GWS032240 (Norfolk I., Australia)
Gelinaria ulvoidea GWS011421 (SA, Australia)
Hallymenia cf. bullosa GWS023350 (Lord Howe I., Australia)
Halymenia cf. mirabilis SPF 57795 (Brazil)
Halymenia malaysiana PSM12834 (Malaysia)
Halymenia ignifera SPF 57817 (Brazil)
Halymenia sp. GWS028717 (Philippines)
Halymenia durvillei GWS028762 (Philippines)
Halymenia floresii GWS011961 (Portugal)
Halymenia pseudofloresii GWS011901 (Bermuda)
Halymenia cearensis SPF 57794 (Brazil)
Halymenia tondoana GWS028767 (Philippines)
Halymenia silviae SPF 57814 (Brazil)
Halymenia pinnatifida SPF 57871 (Brazil)
Continued

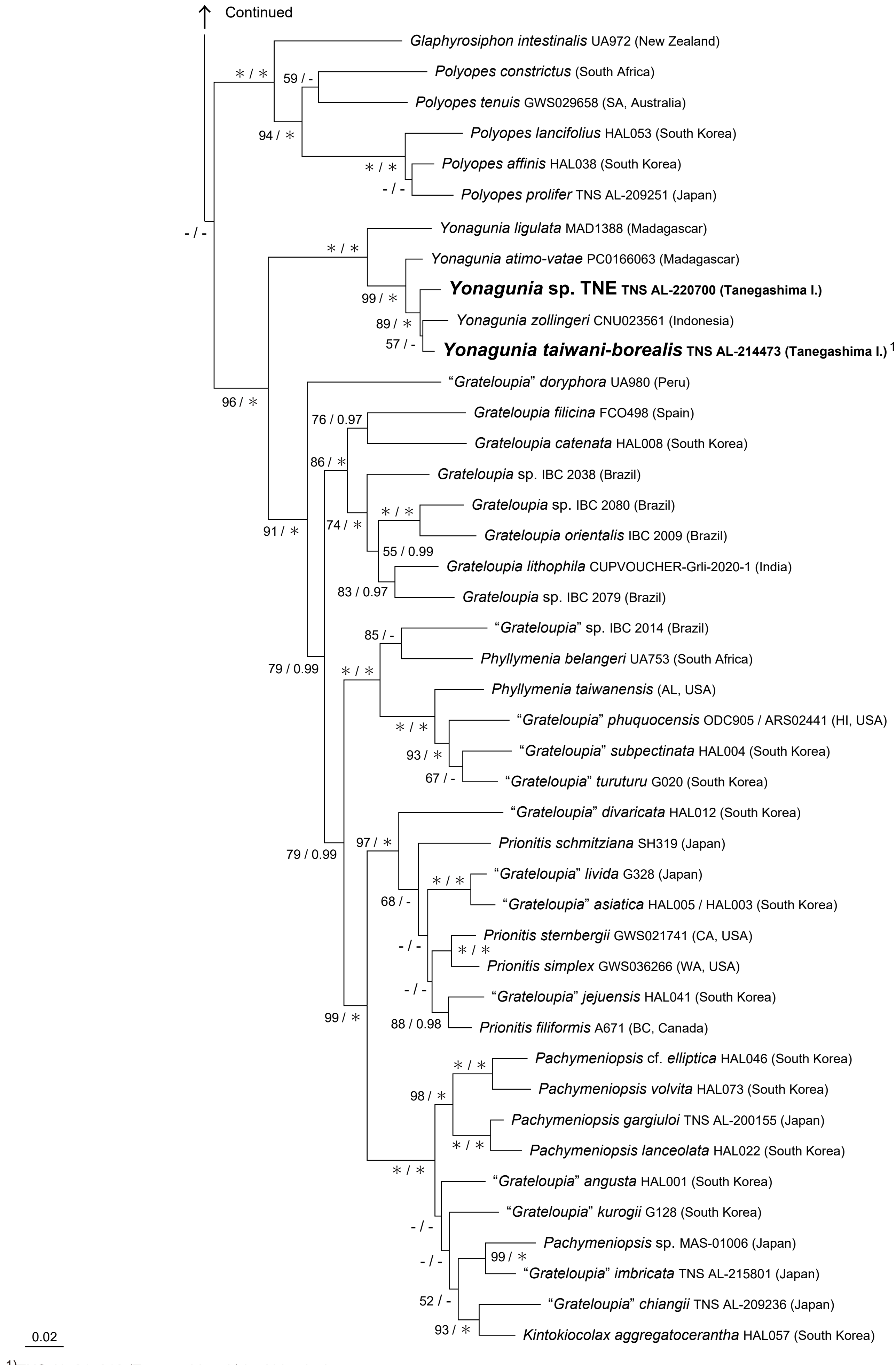

[1]TNS AL-215816 (Tanegashima I.) had identical sequence.

S47C Fig. Maximum likelihood phylogeny for Halymeniaceae species based on combined *rbc*L and *cox*1 DNA sequences. Values are indicated at the branches: bootstrap (BP; ≥ 50%) and Bayesian posterior probabilities (PP; ≥ 0.95). Asterisks (∗) indicate 100% BP and 1.00 PP.
