# Supplementary material for: DNA-based floristic survey of red algae (Rhodophyta) growing in the mesophotic coral ecosystems (MCEs) offshore of Tanegashima Island, northern Ryukyu Archipelago, Japan
Source: PLoS One. 2025 Mar 10;20(3):e0316067. doi: 10.1371/journal.pone.0316067 (PMC11893125; doi:10.1371/journal.pone.0316067)
Supplement: S5 File — Maximum likelihood phylogeny of red algae collected from offshore Tanegashima Island. (ZIP) [file pone.0316067.s005.zip › S5_File/S49_Fig.pdf]

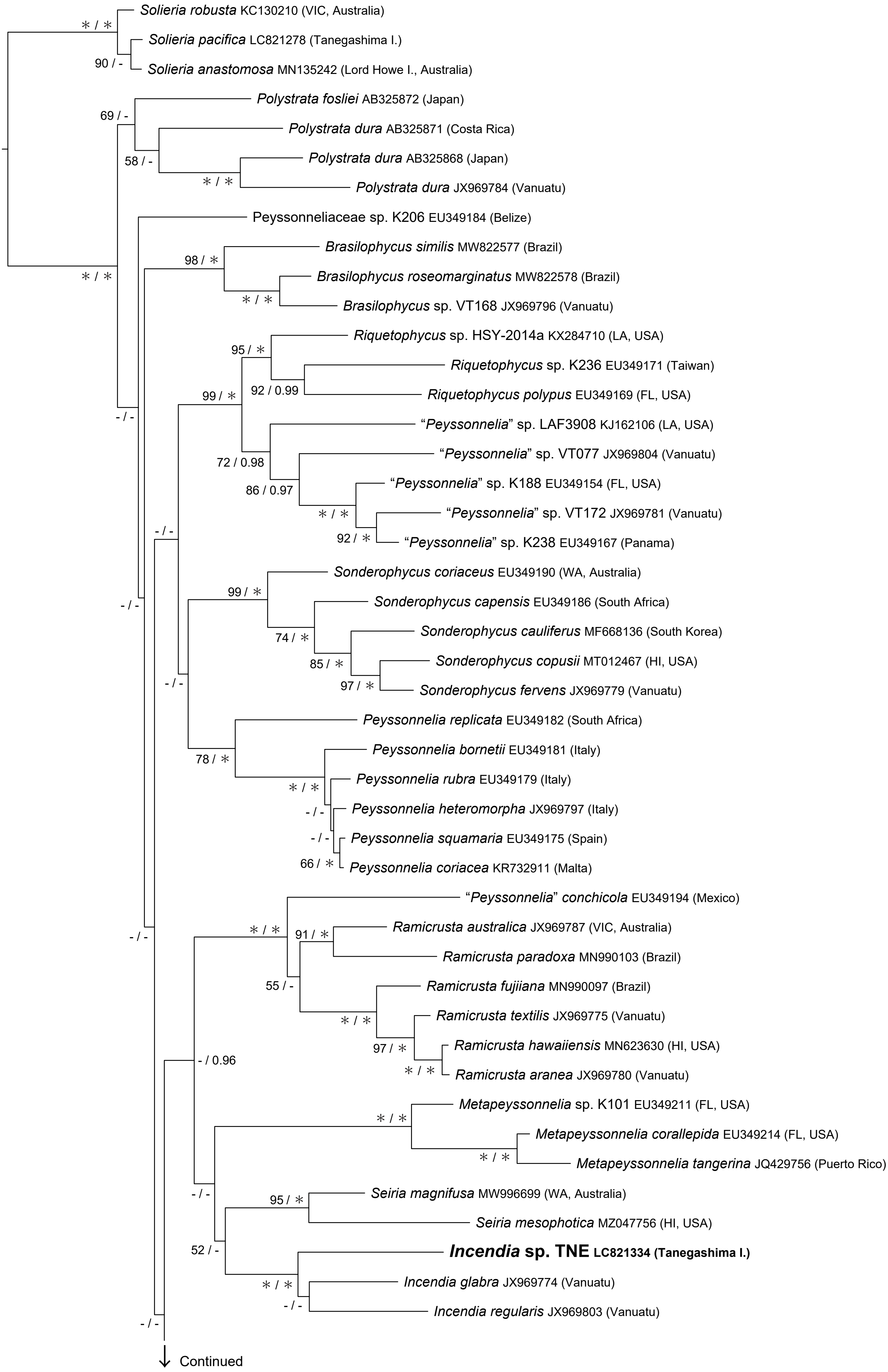

Solieria robusta KC130210 (VIC, Australia)
Solieria pacifica LC821278 (Tanegashima I.)
Solieria anastomosa MN135242 (Lord Howe I., Australia)
Polystrata fosliei AB325872 (Japan)
Polystrata dura AB325871 (Costa Rica)
Polystrata dura AB325868 (Japan)
Polystrata dura JX969784 (Vanuatu)
Peyssonneliaceae sp. K206 EU349184 (Belize)
Brasilophycus similis MW822577 (Brazil)
Brasilophycus roseomarginatus MW822578 (Brazil)
Brasilophycus sp. VT168 JX969796 (Vanuatu)
Riquetophycus sp. HSY-2014a KX284710 (LA, USA)
Riquetophycus sp. K236 EU349171 (Taiwan)
Riquetophycus polypus EU349169 (FL, USA)
"Peyssonnelia" sp. LAF3908 KJ162106 (LA, USA)
"Peyssonnelia" sp. VT077 JX969804 (Vanuatu)
"Peyssonnelia" sp. K188 EU349154 (FL, USA)
"Peyssonnelia" sp. VT172 JX969781 (Vanuatu)
"Peyssonnelia" sp. K238 EU349167 (Panama)
Sonderophycus coriaceus EU349190 (WA, Australia)
Sonderophycus capensis EU349186 (South Africa)
Sonderophycus cauliferus MF668136 (South Korea)
Sonderophycus copusii MT012467 (HI, USA)
Sonderophycus fervens JX969779 (Vanuatu)
Peyssonnelia replicata EU349182 (South Africa)
Peyssonnelia bornetii EU349181 (Italy)
Peyssonnelia rubra EU349179 (Italy)
Peyssonnelia heteromorpha JX969797 (Italy)
Peyssonnelia squamaria EU349175 (Spain)
Peyssonnelia coriacea KR732911 (Malta)
"Peyssonnelia" conchicola EU349194 (Mexico)
Ramicrusta australica JX969787 (VIC, Australia)
Ramicrusta paradoxa MN990103 (Brazil)
Ramicrusta fujiiana MN990097 (Brazil)
Ramicrusta textilis JX969775 (Vanuatu)
Ramicrusta hawaiiensis MN623630 (HI, USA)
Ramicrusta aranea JX969780 (Vanuatu)
Metapeyssonnelia sp. K101 EU349211 (FL, USA)
Metapeyssonnelia corallepida EU349214 (FL, USA)
Metapeyssonnelia tangerina JQ429756 (Puerto Rico)
Seiria magnifusa MW996699 (WA, Australia)
Seiria mesophotica MZ047756 (HI, USA)
Incendia sp. TNE LC821334 (Tanegashima I.)
Incendia glabra JX969774 (Vanuatu)
Incendia regularis JX969803 (Vanuatu)
Continued

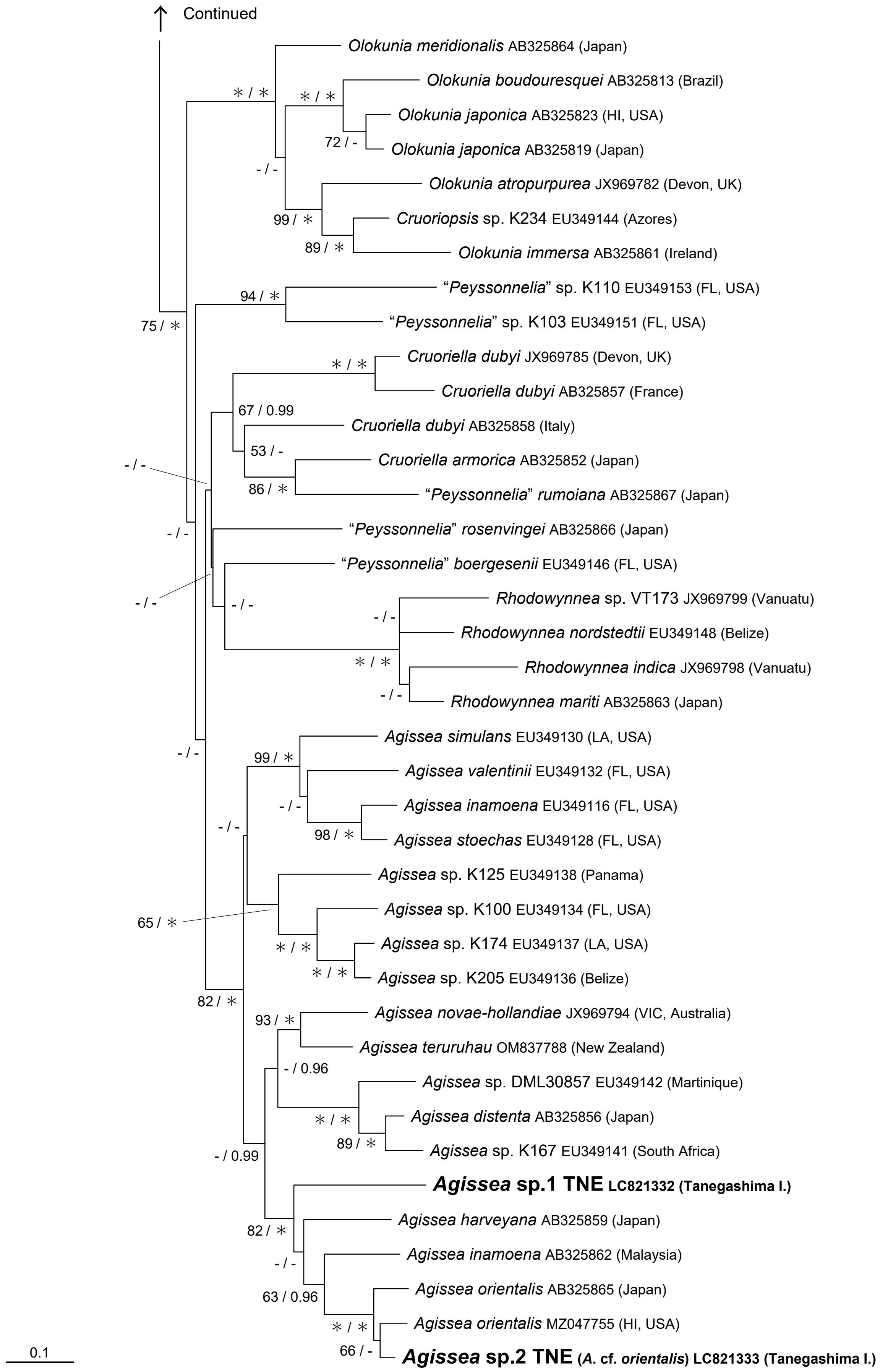

S49A Fig. Maximum likelihood phylogeny for Peyssonneliaceae species based on *rbc*L DNA sequences. Values are indicated at the branches: bootstrap (BP; ≥ 50%) and Bayesian posterior probabilities (PP; ≥ 0.95). Asterisks (*) indicate 100% BP and 1.00 PP.

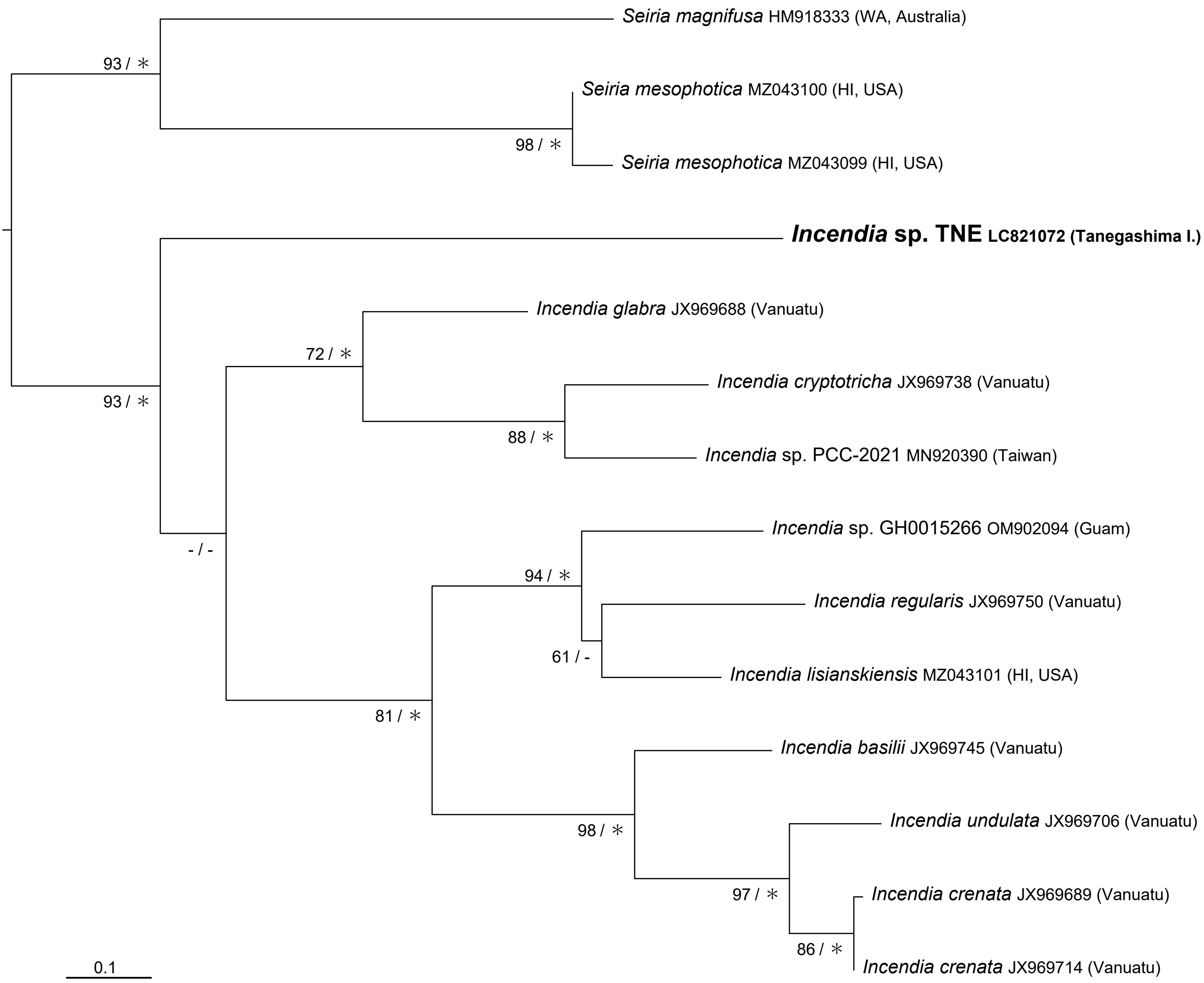

S49B Fig. Maximum likelihood phylogeny for *Incendia* species based on *cox*1 DNA sequences. Values are indicated at the branches: bootstrap (BP; ≥ 50%) and Bayesian posterior probabilities (PP; ≥ 0.95). Asterisks (＊) indicate 100% BP and 1.00 PP.

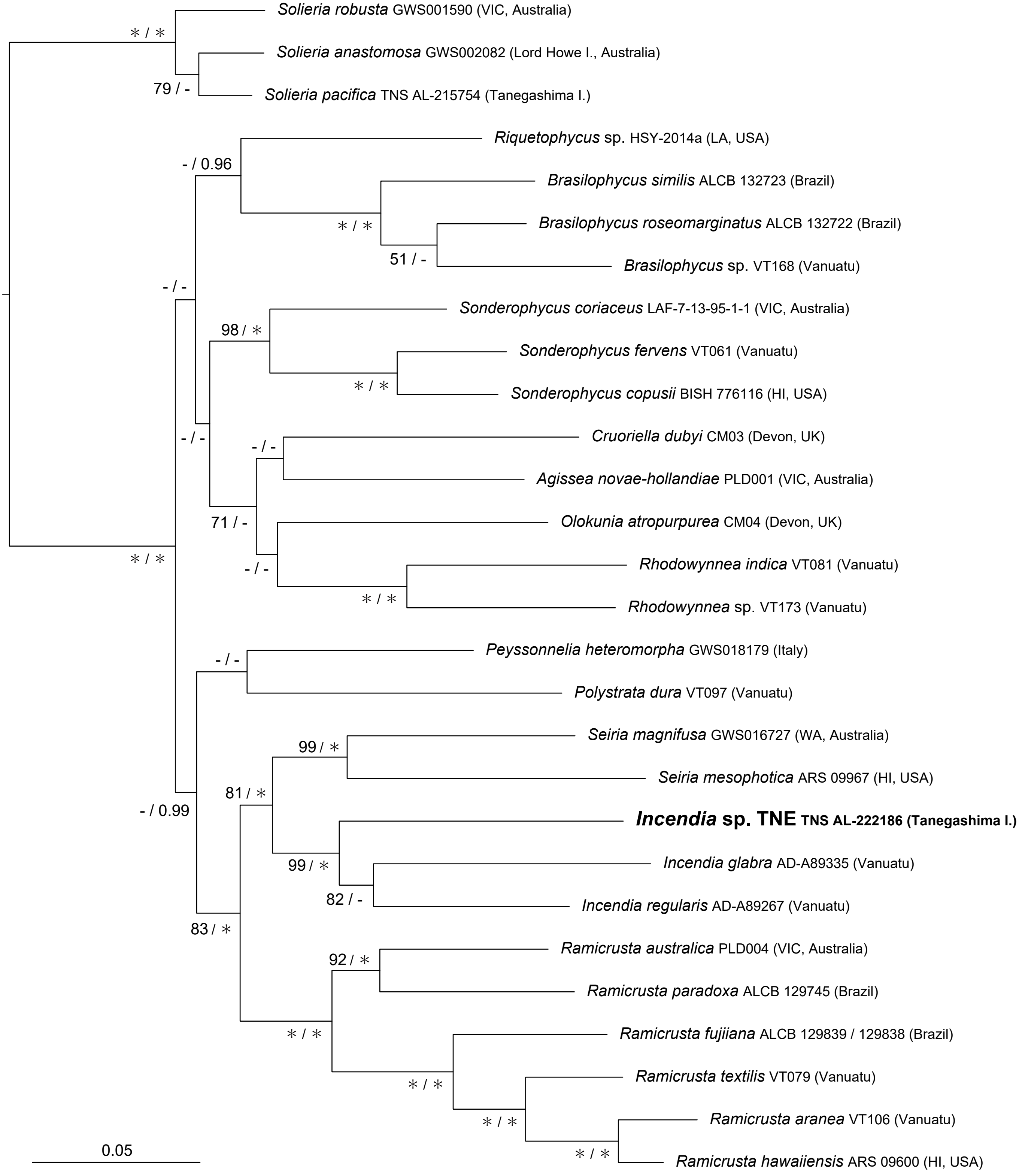

S49C Fig. Maximum likelihood phylogeny for Peyssonneliaceae species based on combined *rbc*L and *cox*1 DNA sequences. Values are indicated at the branches: bootstrap (BP; ≥ 50%) and Bayesian posterior probabilities (PP; ≥ 0.95). Asterisks (∗) indicate 100% BP and 1.00 PP.
