# Supplementary material for: DNA-based floristic survey of red algae (Rhodophyta) growing in the mesophotic coral ecosystems (MCEs) offshore of Tanegashima Island, northern Ryukyu Archipelago, Japan
Source: PLoS One. 2025 Mar 10;20(3):e0316067. doi: 10.1371/journal.pone.0316067 (PMC11893125; doi:10.1371/journal.pone.0316067)
Supplement: S5 File — Maximum likelihood phylogeny of red algae collected from offshore Tanegashima Island. (ZIP) [file pone.0316067.s005.zip › S5_File/S51_Fig.pdf]

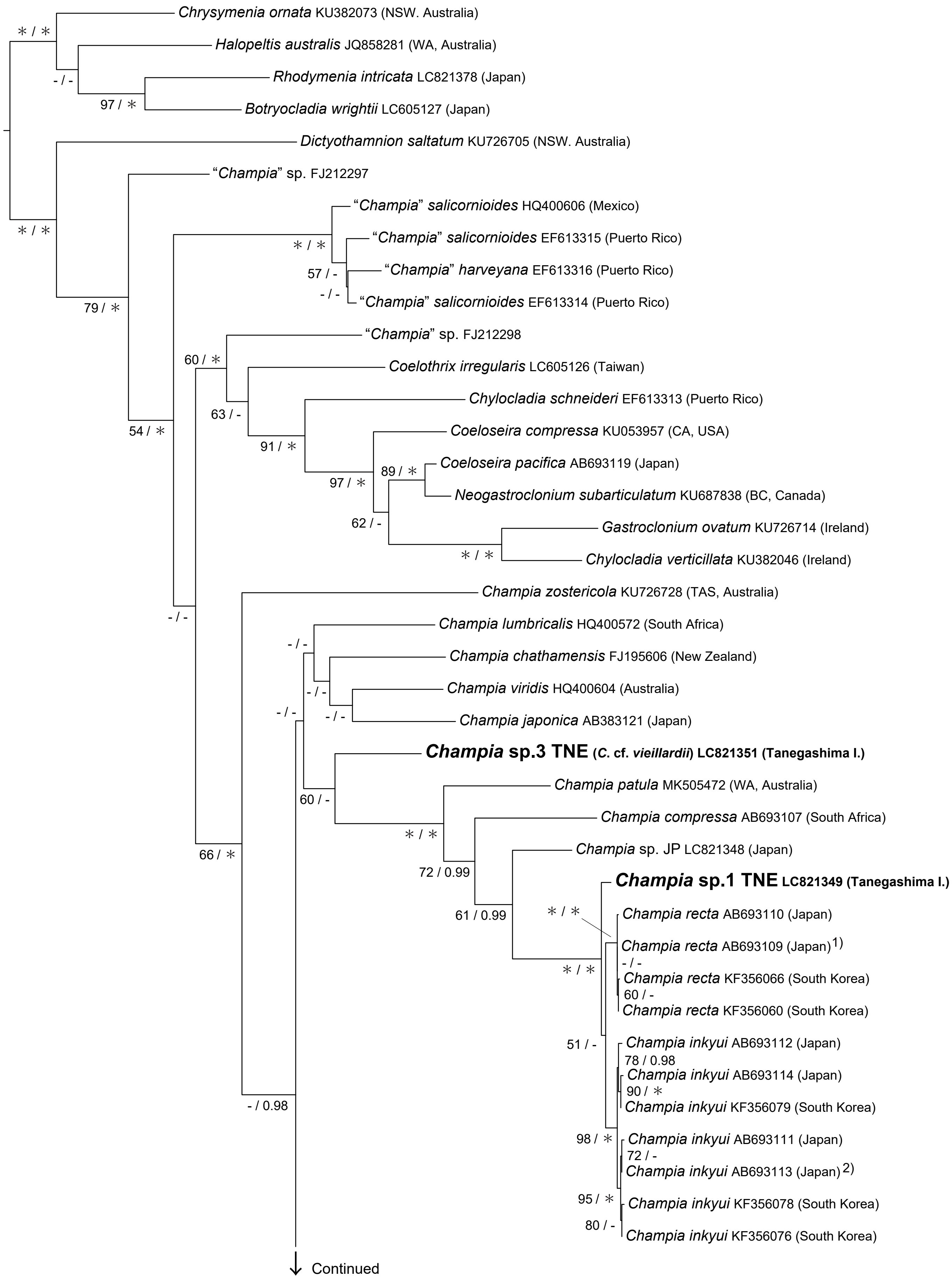
Chrysymenia ornata KU382073 (NSW. Australia)
* / *
Halopeltis australis JQ858281 (WA, Australia)
- / -
Rhodymenia intricata LC821378 (Japan)
97 / *
Botryocladia wrightii LC605127 (Japan)
Dictyothamnion saltatum KU726705 (NSW. Australia)
* / *
"Champia" sp. FJ212297
"Champia" salicornioides HQ400606 (Mexico)
* / *
"Champia" salicornioides EF613315 (Puerto Rico)
57 / -
"Champia" harveyana EF613316 (Puerto Rico)
- / -
"Champia" salicornioides EF613314 (Puerto Rico)
79 / *
"Champia" sp. FJ212298
60 / *
Coelothrix irregularis LC605126 (Taiwan)
Chylocladia schneideri EF613313 (Puerto Rico)
63 / -
54 / *
Coeloseira compressa KU053957 (CA, USA)
91 / *
Coeloseira pacifica AB693119 (Japan)
89 / *
97 / *
Neogastroclonium subarticulatum KU687838 (BC, Canada)
62 / -
Gastroclonium ovatum KU726714 (Ireland)
* / *
Chylocladia verticillata KU382046 (Ireland)
Champia zostericola KU726728 (TAS, Australia)
- / -
Champia lumbricalis HQ400572 (South Africa)
- / -
Champia chathamensis FJ195606 (New Zealand)
- / -
Champia viridis HQ400604 (Australia)
- / -
- / -
Champia japonica AB383121 (Japan)
Champia sp.3 TNE (C. cf. vieillardii) LC821351 (Tanegashima I.)
Champia patula MK505472 (WA, Australia)
60 / -
Champia compressa AB693107 (South Africa)
* / *
66 / *
Champia sp. JP LC821348 (Japan)
72 / 0.99
Champia sp.1 TNE LC821349 (Tanegashima I.)
61 / 0.99
* / *
Champia recta AB693110 (Japan)
Champia recta AB693109 (Japan)1)
- / -
* / *
Champia recta KF356066 (South Korea)
60 / -
Champia recta KF356060 (South Korea)
51 / -
Champia inkyui AB693112 (Japan)
78 / 0.98
Champia inkyui AB693114 (Japan)
90 / *
- / 0.98
Champia inkyui KF356079 (South Korea)
98 / *
Champia inkyui AB693111 (Japan)
72 / -
Champia inkyui AB693113 (Japan)2)
95 / *
Champia inkyui KF356078 (South Korea)
80 / -
Champia inkyui KF356076 (South Korea)
Continued

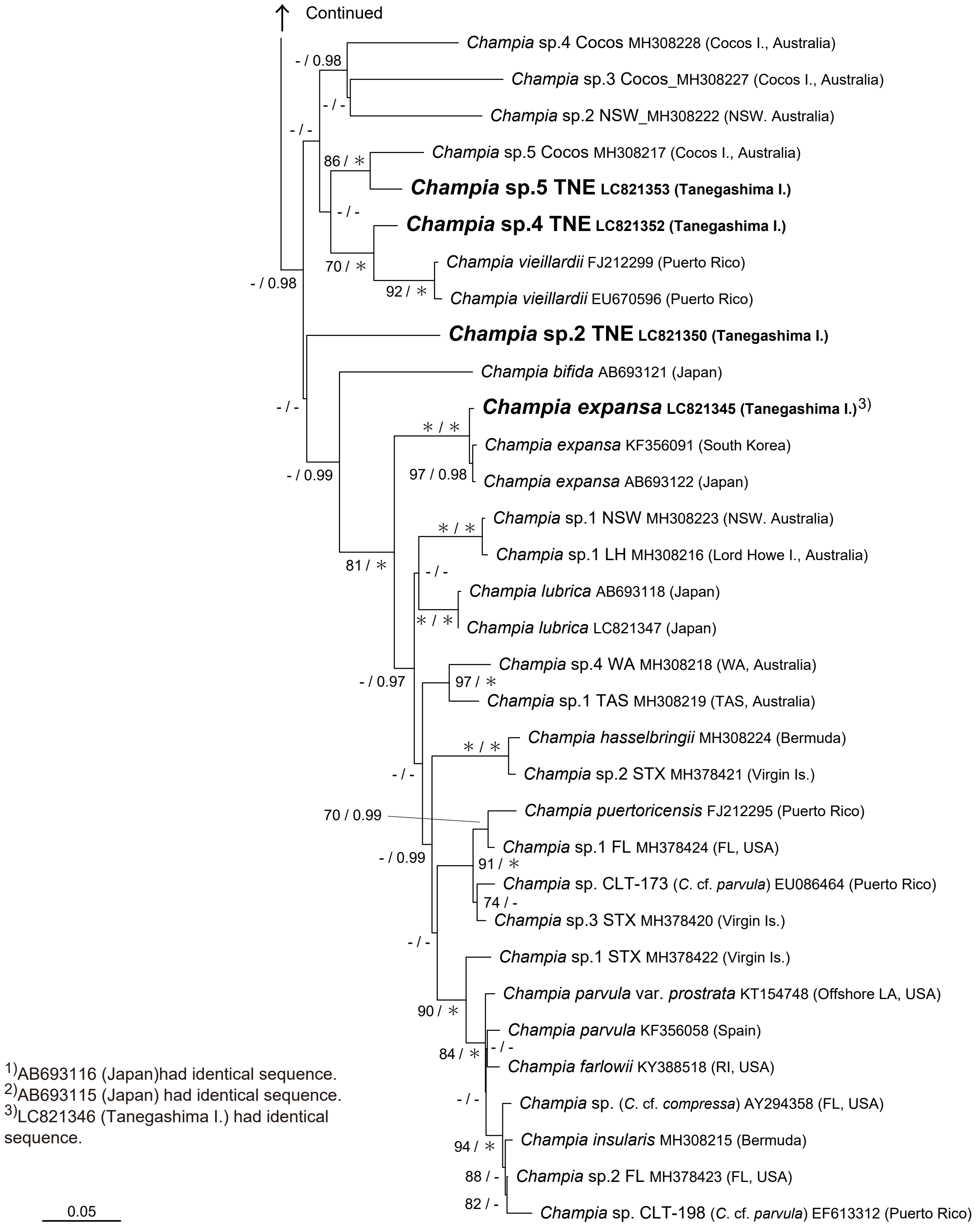

S51A Fig. Maximum likelihood phylogeny for Champiaceae species based on *rbc*L DNA sequences.
Values are indicated at the branches: bootstrap (BP; ≥ 50%) and Bayesian posterior probabilities (PP; ≥ 0.95). Asterisks (∗) indicate 100% BP and 1.00 PP.

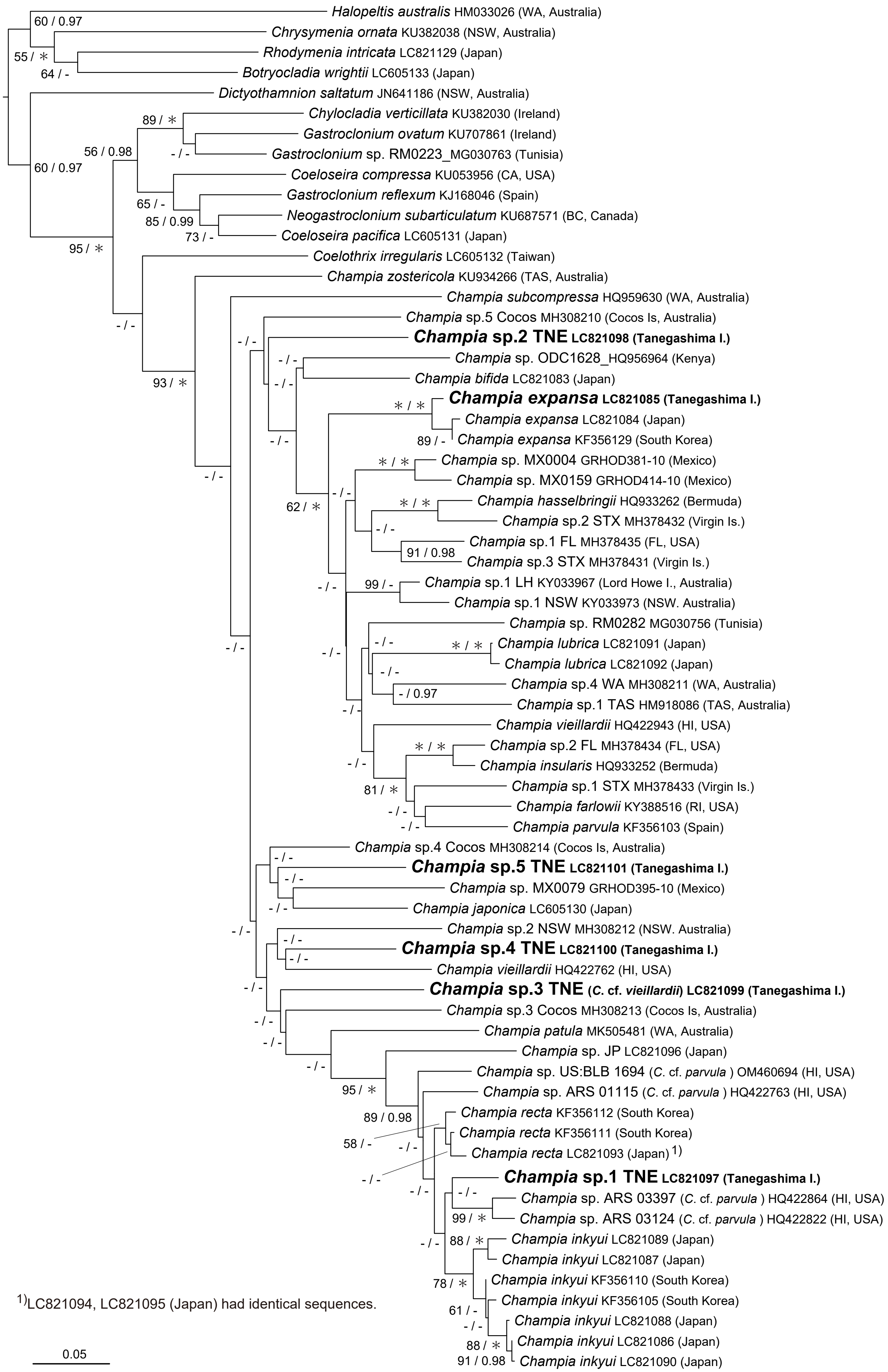

S51B Fig. Maximum likelihood phylogeny for Champiaceae species based on *cox*1 DNA sequences.
Values are indicated at the branches: bootstrap (BP; ≥ 50%) and Bayesian posterior probabilities (PP; ≥ 0.95). Asterisks (∗) indicate 100% BP and 1.00 PP.

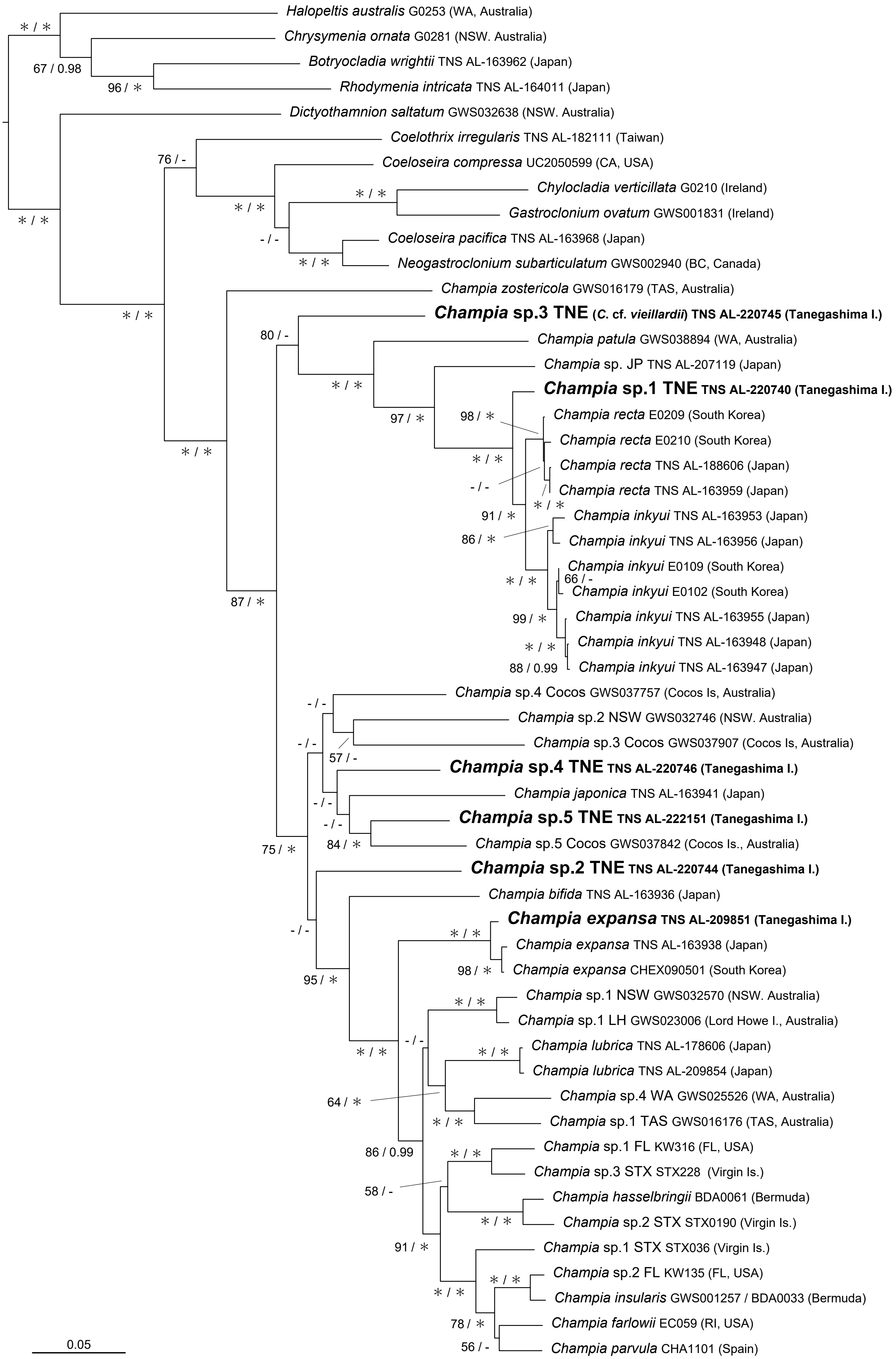

S51C Fig. Maximum likelihood phylogeny for Champiaceae species based on combined *rbc*L and *cox*1 DNA sequences. Values are indicated at the branches: bootstrap (BP; ≥ 50%) and Bayesian posterior probabilities (PP; ≥ 0.95). Asterisks (∗) indicate 100% BP and 1.00 PP.
