# Supplementary material for: DNA-based floristic survey of red algae (Rhodophyta) growing in the mesophotic coral ecosystems (MCEs) offshore of Tanegashima Island, northern Ryukyu Archipelago, Japan
Source: PLoS One. 2025 Mar 10;20(3):e0316067. doi: 10.1371/journal.pone.0316067 (PMC11893125; doi:10.1371/journal.pone.0316067)
Supplement: S5 File — Maximum likelihood phylogeny of red algae collected from offshore Tanegashima Island. (ZIP) [file pone.0316067.s005.zip › S5_File/S54_Fig.pdf]

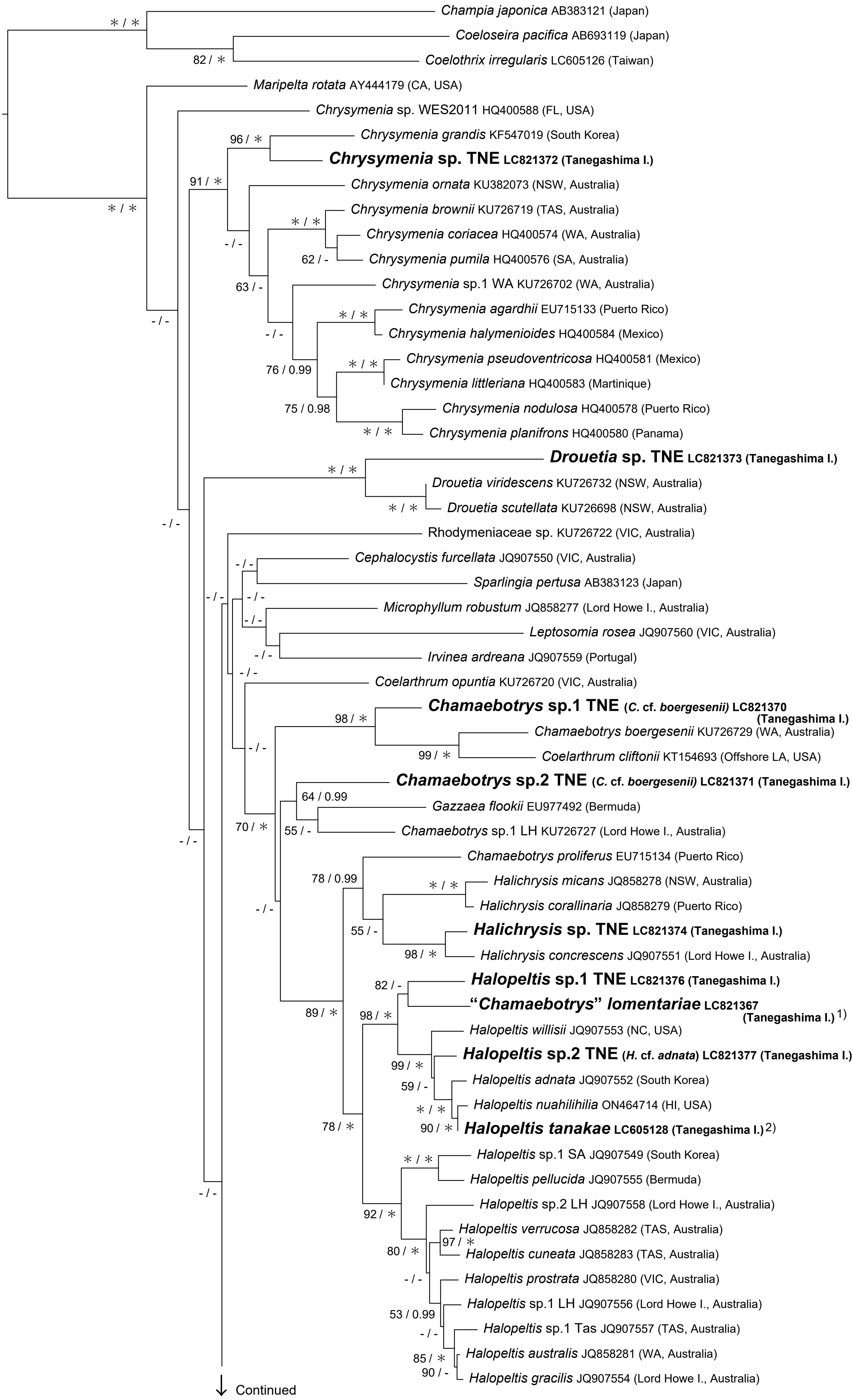

Champia japonica AB383121 (Japan)
Coeloseira pacifica AB693119 (Japan)
Coelothrix irregularis LC605126 (Taiwan)
Maripelta rotata AY444179 (CA, USA)
Chrysymenia sp. WES2011 HQ400588 (FL, USA)
Chrysymenia grandis KF547019 (South Korea)
Chrysymenia sp. TNE LC821372 (Tanegashima I.)
Chrysymenia ornata KU382073 (NSW, Australia)
Chrysymenia brownii KU726719 (TAS, Australia)
Chrysymenia coriacea HQ400574 (WA, Australia)
Chrysymenia pumila HQ400576 (SA, Australia)
Chrysymenia sp.1 WA KU726702 (WA, Australia)
Chrysymenia agardhii EU715133 (Puerto Rico)
Chrysymenia halymenioides HQ400584 (Mexico)
Chrysymenia pseudoventricosa HQ400581 (Mexico)
Chrysymenia littleriana HQ400583 (Martinique)
Chrysymenia nodulosa HQ400578 (Puerto Rico)
Chrysymenia planifrons HQ400580 (Panama)
Drouetia sp. TNE LC821373 (Tanegashima I.)
Drouetia viridescens KU726732 (NSW, Australia)
Drouetia scutellata KU726698 (NSW, Australia)
Rhodymeniaceae sp. KU726722 (VIC, Australia)
Cephalocystis furcellata JQ907550 (VIC, Australia)
Sparlingia pertusa AB383123 (Japan)
Microphyllum robustum JQ858277 (Lord Howe I., Australia)
Leptosomia rosea JQ907560 (VIC, Australia)
Irvinea ardreana JQ907559 (Portugal)
Coelarthrum opuntia KU726720 (VIC, Australia)
Chamaebotrys sp.1 TNE (C. cf. boergesenii) LC821370 (Tanegashima I.)
Chamaebotrys boergesenii KU726729 (WA, Australia)
Coelarthrum cliftonii KT154693 (Offshore LA, USA)
Chamaebotrys sp.2 TNE (C. cf. boergesenii) LC821371 (Tanegashima I.)
Gazzaea flookii EU977492 (Bermuda)
Chamaebotrys sp.1 LH KU726727 (Lord Howe I., Australia)
Chamaebotrys proliferus EU715134 (Puerto Rico)
Halichrysis micans JQ858278 (NSW, Australia)
Halichrysis corallinaria JQ858279 (Puerto Rico)
Halichrysis sp. TNE LC821374 (Tanegashima I.)
Halichrysis concrescens JQ907551 (Lord Howe I., Australia)
Halopeltis sp.1 TNE LC821376 (Tanegashima I.)
"Chamaebotrys" lomentariae LC821367 (Tanegashima I.) 1)
Halopeltis willisii JQ907553 (NC, USA)
Halopeltis sp.2 TNE (H. cf. adnata) LC821377 (Tanegashima I.)
Halopeltis adnata JQ907552 (South Korea)
Halopeltis nuahilihilia ON464714 (HI, USA)
Halopeltis tanakae LC605128 (Tanegashima I.) 2)
Halopeltis sp.1 SA JQ907549 (South Korea)
Halopeltis pellucida JQ907555 (Bermuda)
Halopeltis sp.2 LH JQ907558 (Lord Howe I., Australia)
Halopeltis verrucosa JQ858282 (TAS, Australia)
Halopeltis cuneata JQ858283 (TAS, Australia)
Halopeltis prostrata JQ858280 (VIC, Australia)
Halopeltis sp.1 LH JQ907556 (Lord Howe I., Australia)
Halopeltis sp.1 Tas JQ907557 (TAS, Australia)
Halopeltis australis JQ858281 (WA, Australia)
Halopeltis gracilis JQ907554 (Lord Howe I., Australia)
/
82/*
/
96/*
91/*
/
-/-
62/-
63/-
/
-/-
76/0.99
/
75/0.98
/
-/-
/
/
-/-
-/-
-/-
-/-
-/-
-/-
98/*
99/*
-/-
64/0.99
70/*
55/-
78/0.99
/
-/-
55/-
98/*
82/-
89/*
98/*
99/*
59/-
/
78/*
90/*
/
-/-
92/*
97/*
80/*
-/-
53/0.99
-/-
85/*
90/-
Continued

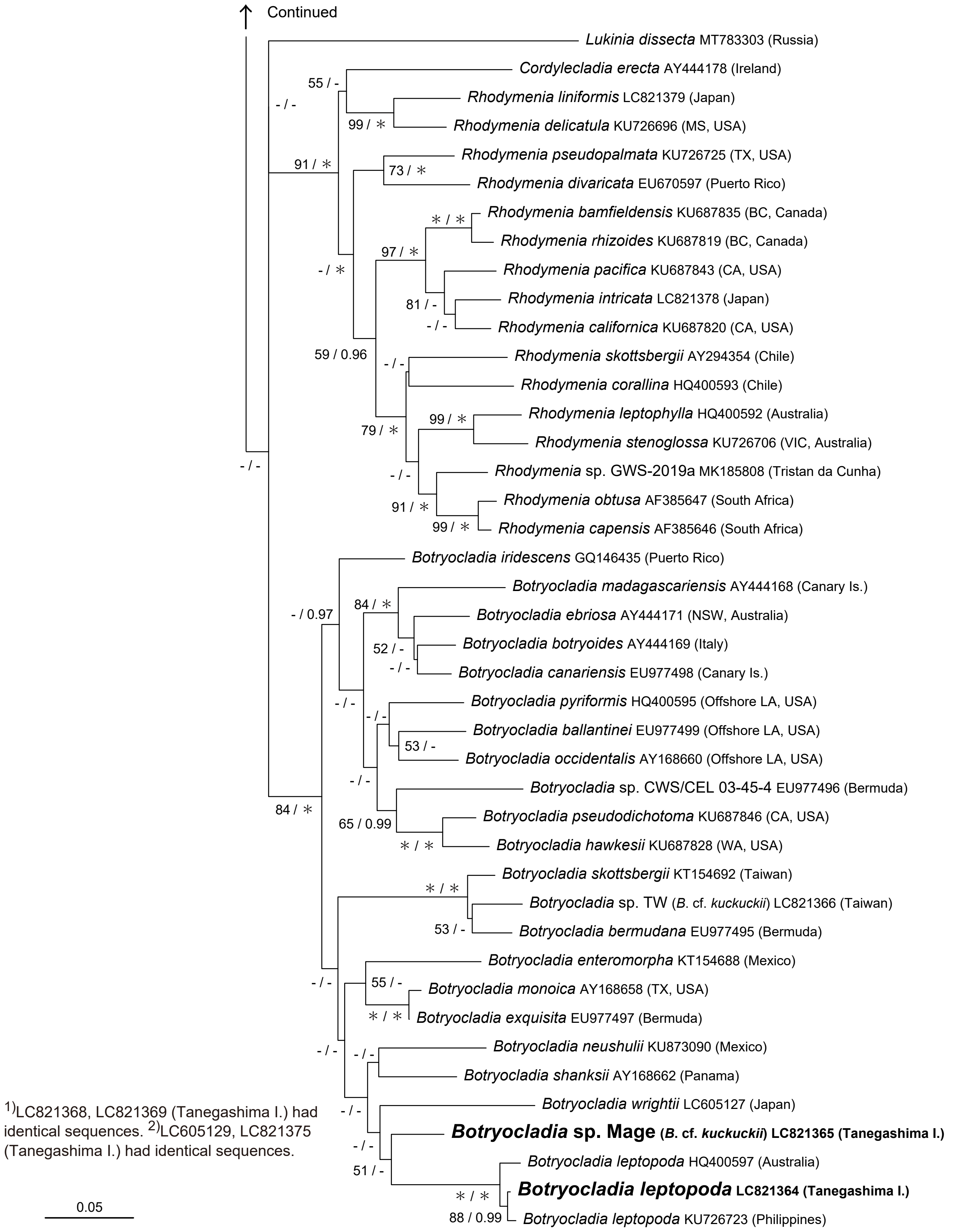

S54A Fig. Maximum likelihood phylogeny for Rhodymeniaceae species based on *rbc*L DNA sequences.

Values are indicated at the branches: bootstrap (BP; ≥ 50%) and Bayesian posterior probabilities (PP; ≥ 0.95). Asterisks (∗) indicate 100% BP and 1.00 PP.

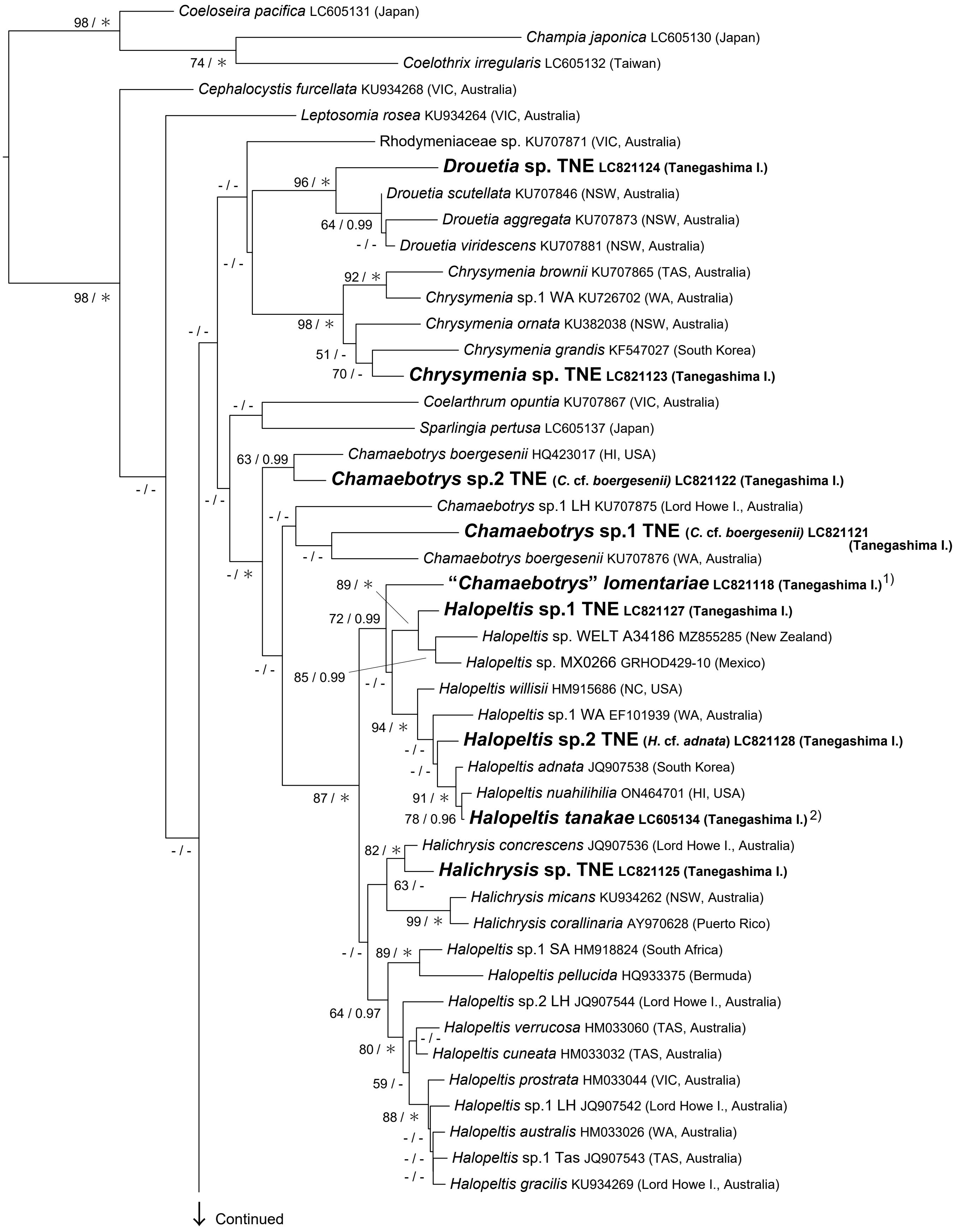

Coeloseira pacifica LC605131 (Japan)
Champia japonica LC605130 (Japan)
Coelothrix irregularis LC605132 (Taiwan)
Cephalocystis furcellata KU934268 (VIC, Australia)
Leptosomia rosea KU934264 (VIC, Australia)
Rhodymeniaceae sp. KU707871 (VIC, Australia)
Drouetia sp. TNE LC821124 (Tanegashima I.)
Drouetia scutellata KU707846 (NSW, Australia)
Drouetia aggregata KU707873 (NSW, Australia)
Drouetia viridescens KU707881 (NSW, Australia)
Chrysymenia brownii KU707865 (TAS, Australia)
Chrysymenia sp.1 WA KU726702 (WA, Australia)
Chrysymenia ornata KU382038 (NSW, Australia)
Chrysymenia grandis KF547027 (South Korea)
Chrysymenia sp. TNE LC821123 (Tanegashima I.)
Coelarthrum opuntia KU707867 (VIC, Australia)
Sparlingia pertusa LC605137 (Japan)
Chamaebotrys boergesenii HQ423017 (HI, USA)
Chamaebotrys sp.2 TNE (C. cf. boergesenii) LC821122 (Tanegashima I.)
Chamaebotrys sp.1 LH KU707875 (Lord Howe I., Australia)
Chamaebotrys sp.1 TNE (C. cf. boergesenii) LC821121 (Tanegashima I.)
Chamaebotrys boergesenii KU707876 (WA, Australia)
"Chamaebotrys" lomentariae LC821118 (Tanegashima I.)1)
Halopeltis sp.1 TNE LC821127 (Tanegashima I.)
Halopeltis sp. WELT A34186 MZ855285 (New Zealand)
Halopeltis sp. MX0266 GRHOD429-10 (Mexico)
Halopeltis willisii HM915686 (NC, USA)
Halopeltis sp.1 WA EF101939 (WA, Australia)
Halopeltis sp.2 TNE (H. cf. adnata) LC821128 (Tanegashima I.)
Halopeltis adnata JQ907538 (South Korea)
Halopeltis nuahilihilia ON464701 (HI, USA)
Halopeltis tanakae LC605134 (Tanegashima I.)2)
Halichrysis concrescens JQ907536 (Lord Howe I., Australia)
Halichrysis sp. TNE LC821125 (Tanegashima I.)
Halichrysis micans KU934262 (NSW, Australia)
Halichrysis corallinaria AY970628 (Puerto Rico)
Halopeltis sp.1 SA HM918824 (South Africa)
Halopeltis pellucida HQ933375 (Bermuda)
Halopeltis sp.2 LH JQ907544 (Lord Howe I., Australia)
Halopeltis verrucosa HM033060 (TAS, Australia)
Halopeltis cuneata HM033032 (TAS, Australia)
Halopeltis prostrata HM033044 (VIC, Australia)
Halopeltis sp.1 LH JQ907542 (Lord Howe I., Australia)
Halopeltis australis HM033026 (WA, Australia)
Halopeltis sp.1 Tas JQ907543 (TAS, Australia)
Halopeltis gracilis KU934269 (Lord Howe I., Australia)
98 / *
74 / *
98 / *
- / -
96 / *
64 / 0.99
- / -
- / -
92 / *
98 / *
51 / -
70 / -
- / -
- / -
63 / 0.99
- / -
- / -
- / -
- / *
89 / *
72 / 0.99
85 / 0.99
- / -
- / -
94 / *
- / -
- / -
91 / *
78 / 0.96
87 / *
82 / *
63 / -
99 / *
- / -
89 / *
64 / 0.97
80 / *
- / -
59 / -
88 / *
- / -
- / -
- / -
- / -
Continued

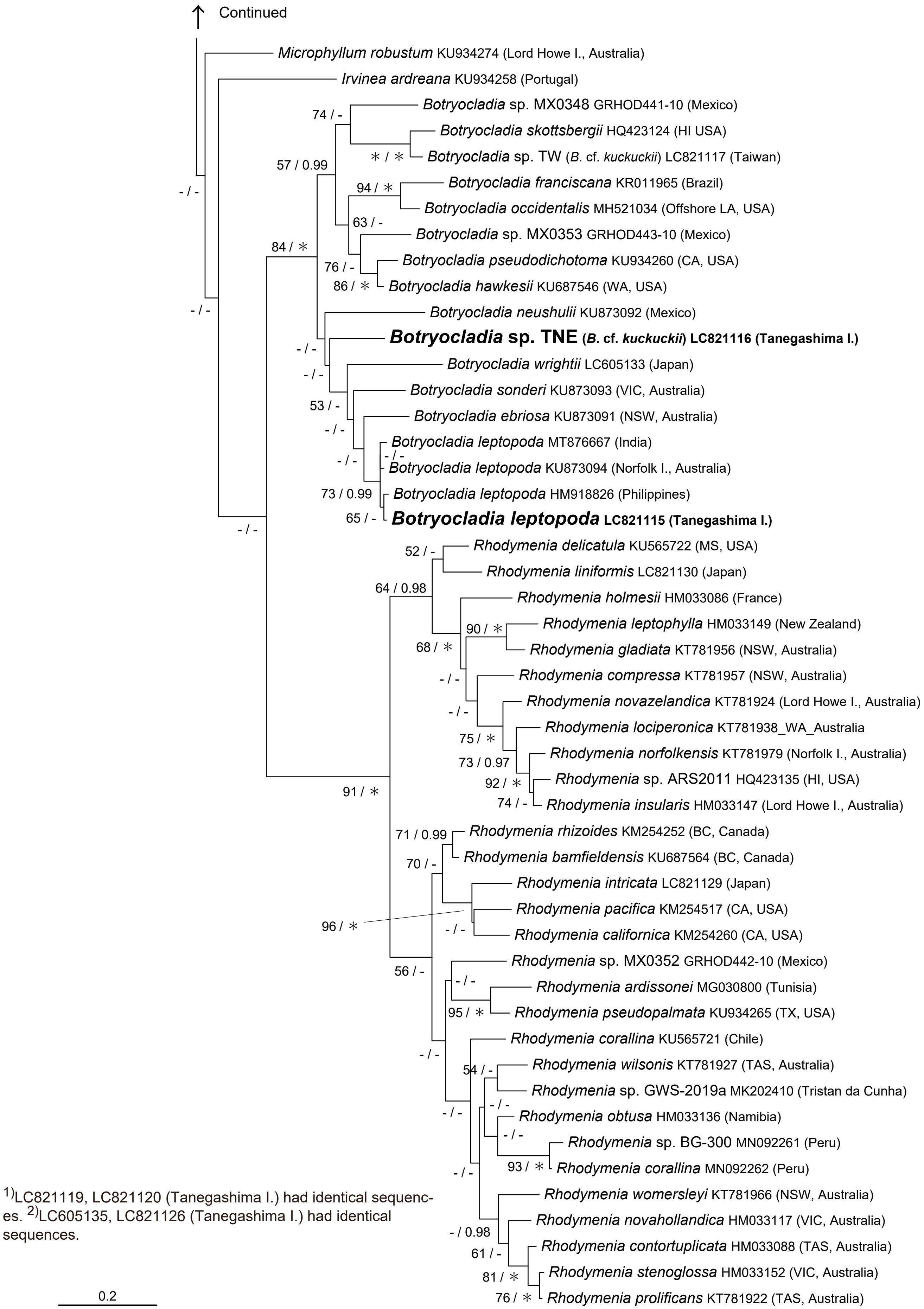

[1]LC821119, LC821120 (Tanegashima I.) had identical sequences. [2]LC605135, LC821126 (Tanegashima I.) had identical sequences.

S54B Fig. Maximum likelihood phylogeny for Rhodymeniaceae species based on *cox*1 DNA sequences. Values are indicated at the branches: bootstrap (BP; ≥ 50%) and Bayesian posterior probabilities (PP; ≥ 0.95). Asterisks (*) indicate 100% BP and 1.00 PP.

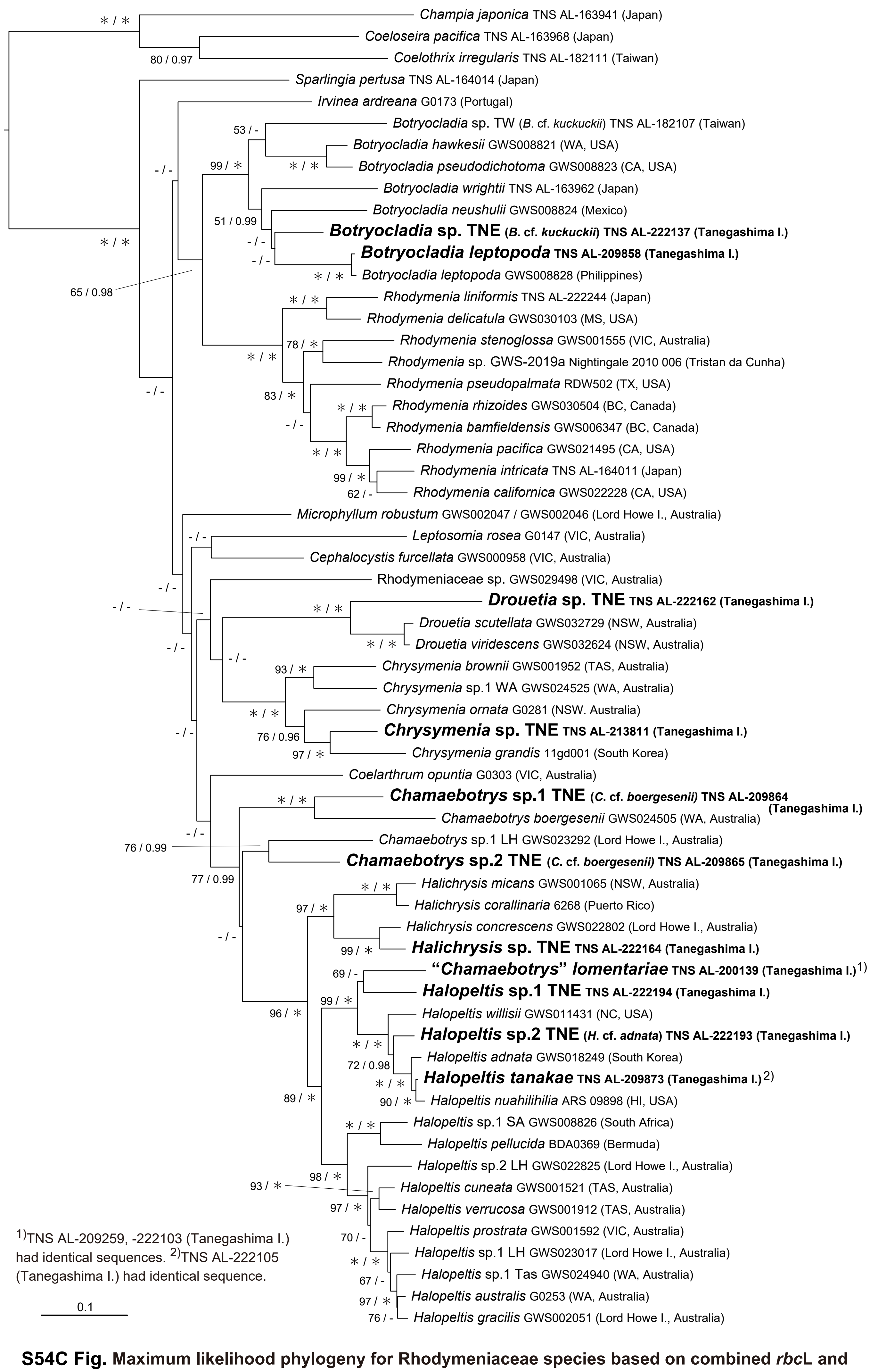

S54C Fig. Maximum likelihood phylogeny for Rhodymeniaceae species based on combined *rbc*L and *cox*1 DNA sequences. Values are indicated at the branches: bootstrap (BP; ≥ 50%) and Bayesian posterior probabilities (PP; ≥ 0.95). Asterisks (∗) indicate 100% BP and 1.00 PP.
