# Supplementary material for: DNA-based floristic survey of red algae (Rhodophyta) growing in the mesophotic coral ecosystems (MCEs) offshore of Tanegashima Island, northern Ryukyu Archipelago, Japan
Source: PLoS One. 2025 Mar 10;20(3):e0316067. doi: 10.1371/journal.pone.0316067 (PMC11893125; doi:10.1371/journal.pone.0316067)
Supplement: S6 File — (DOCX) [file pone.0316067.s006.docx]

**S6 File. Taxonomic information and morpho-anatomical observations of newly recorded species for Japan.**

All Japanese specimens were collected from offshore Tanegashima Island.

*Pseudopolyneura hyacinthina* (J.C.Kang & M.S.Kim) M.J.Wynne 2016: 1. [1] (S4 Fig. in S4 File)

Basionym: *Erythroglossum hyacinthinum* J.C.Kang & M.S.Kim 2014: 3. Figs 1, 2. [2]

Type locality: Chujado, Jeju province, South Korea.

Holotype: JNUB; JN130604-1 (35º58'04.28"N, 126º17'08.64E; 4 June 2013; 12 m depth)

Isotypes: JNUB; JN130604-3, JN130604-5 to -9. KB; NIBRAL0000137942-3.

Japanese name: Toge-hasujiginu (new name).

Specimens examined: TNS AL-222139 (3 October 2021; *R*. *Terada*); TNS AL-222180 (15 May 2022; *R*. *Terada*); TNS AL-222210 (4 June 2022; *R*. *Terada*).

Morpho-anatomical observation: Thalli were erect, 4.0–7.0 cm in height, flat, membranous, pale to rose-red in color, and attached by small discoid holdfast. The blades were elliptical, oblong to lanceolate, 4.0–12 mm wide, dichotomous, or irregularly divided, often producing marginal bladelets (S4 Fig. A–C). The midrib was conspicuous except for the marginal bladelets and the apical part of the blades (S4 Fig. D). Microscopic dentations were produced from the margins of the blades (S4 Fig. E). The blades were monostromatic except for the midrib part (S4 Fig. E–G). The reproductive organ was not observed.

Remarks: The *rbc*L sequences of Japanese specimens are identical to the holotype of *P*. *hyacinthina* ([KF305299](https://www.ncbi.nlm.nih.gov/nuccore/KF305299)). The blades of Japanese specimens are oblong to lanceolate with many marginal bladelets, whereas those of Korean specimens are elliptical to obovate without marginal bladelets [2]. The margins of the blades with microscopic dentations and monostromatic thalli, except for the midrib of Japanese specimens, were essentially in accordance with those of Korean *P*. *hyacinthina* [2]. Although the habits of Japanese specimens are different from those of Korean specimens, we identified the *Pseudopolyneura* specimens from Japan as *P*. *hyacinthina* based on the absence of divergence of *rbc*L sequences between Japanese and Korean specimens and characteristics of vegetative anatomy.

*Acanthophora dendroides* Harvey 1855: 538. [3] (S5 Fig. in S4 File)

Type locality: Rottnest Island, Western Australia, Australia.

Lectotype: TCD; Herb. Harvey; Trav. Set 224.

Japanese name: Beni-togenori (new name).

Specimens examined: TNS AL-222201 (15 May 2022; *R*. *Terada*).

Morpho-anatomical observation: The thallus was erect, 8.5 cm in height, rose-red in color, and attached by discoid holdfast (S5 Fig. A, F). Stoloniferous axes were produced from the basal part of the thallus (S5 Fig. C). The axes were cylindrical with diameters up to 1.0 mm. The lateral branches were similar to axes, bearing short spines, and were not constricted at the base of branches (S5 Fig. D). The thallus was solid, uniaxial, with 5 pericentral cells surrounded by the pseudoparenchymatous medulla and cortex (S5 Fig. E, F). The reproductive organ was not observed.

Remarks: The *rbc*L sequence of the Japanese specimen is 0.4–0.9% divergent from *A*. *dendroides* from India, Philippines, and Florida, U.S.A. ([MH388514](https://www.ncbi.nlm.nih.gov/nuccore/MH388514), [MH388560](https://www.ncbi.nlm.nih.gov/nuccore/MH388560), [MH388561](https://www.ncbi.nlm.nih.gov/nuccore/MH388561), [MT876666](https://www.ncbi.nlm.nih.gov/nuccore/MT876666)), while the *cox*1 sequence is 1.0–2.4% divergent from *A*. *dendroides* ([MH388702](https://www.ncbi.nlm.nih.gov/nuccore/MH388702), [MH388737](https://www.ncbi.nlm.nih.gov/nuccore/MH388737), [MH388739](https://www.ncbi.nlm.nih.gov/nuccore/MH388739), [MT876665](https://www.ncbi.nlm.nih.gov/nuccore/MT876665)). The stoloniferous axes and branches bearing short spines in the Japanese specimen are essentially consistent with those of Australian *A*. *dendroides* [4-6]. The basal constriction of branches is a key feature of *A*. *dendroides* [4,6,7]. However, Japanese specimen lacks a conspicuous constriction at the base of branches. Womersley [5] suggested that Australian *A*. *dendroides* lacks basal branches constriction. It seems that the degree of basal constriction of branches varies, as Huisman [6] pointed out. Although DNA sequences of *A*. *dendroides* from its type locality (Rottnest Island) are not available, we identified the *Acanthophora* specimen from Japan as *A*. *dendroides* based on the low divergence of *rbc*L and *cox*1 sequences between Japanese and other specimens from India, Philippines, and U.S.A. and the characteristics of habit except for basal constriction of branches.

*Calliblepharis yasutakei* Paiano & A.R.Sherwood *in* Paiano *et al*. 2022: 79. Fig. 3 A–H. [8] (S9 Fig. in S4 File)

Type locality: Kapou (Lisianski), Papahânaumokuâkea Marine National Monument, Hawai'i, U.S.A.

Holotype: BISH; BISH 783229 (26.08363°N, 174.16647°W; 30 July 2019; 98 m depth; *R. Kosaki*)

Japanese name: Yasutake-ibara (new name).

Specimens examined: TNS AL-220756 (25 May 2018; *R*. *Terada & M. Suzuki*); TNS AL-220757 (16 May 2021; *R*. *Terada*).

Morpho-anatomical observation: Thalli were entangled and attached by small holdfasts, rose-red in color, and 3.0 cm high (S9 Fig. A, B). Some axes and branches were secondarily attached to the substratum or adjacent branches by adventitious pads. The axes were flattened to slightly compressed, irregularly, or dichotomously branched, 2.0–2.5 mm in width. The branches were curved and tapered to acute apices, up to 1.0 mm in width (S9 Fig. A, B). In the surface view, the cortical cells were irregular to polygonal, forming incipient rosettes (S9 Fig. C). Thalli were solid, uniaxial, and composed of a thin outer cortex and inner medulla. The cortex was composed of one layer of rounded or cuboidal cells irregularly surrounded by small cells. The medulla was composed of two to four layers of large cells (S9 Fig. D, F). Lenticular thickenings were frequently observed in cortical and medullary cells (S9 Fig. E). In the longitudinal section, an axial filament was surrounded by elongated medullary cells (S9 Fig. G–I). The reproductive organ was not observed.

Remarks: The *rbc*L sequences of Japanese specimens were 0.4% divergent from the holotype of *C*. *yasutakei* ([OL795916](https://www.ncbi.nlm.nih.gov/nuccore/OL795916)), whereas the *cox*1 sequences were 0.2%–1.4% divergent from the holotype and South African specimens ([OL795915](https://www.ncbi.nlm.nih.gov/nuccore/OL795915), [HQ956841](https://www.ncbi.nlm.nih.gov/nuccore/HQ956841.1?report=genbank)). Japanese specimens form many axes and are more branched than Hawaiian *C*. *yasutakei*. The slightly compressed thalli, incipient rosettes in surface view, frequently produce lenticular thickenings in cortical and medullary cells, and the axial filament surrounded by elongated medullary cells in Japanese specimens are essentially consistent with Hawaiian *C*. *yasutakei* [8]. Although the habits of Japanese specimens are different from those of Hawaiian specimens, we identified the *Calliblepharis* specimens from Japan as *C*. *yasutakei* based on the low divergence of *rbc*L and *cox*1 sequences between Japanese and Hawaiian specimens and the characteristics of vegetative anatomy.

*Croisettea kalaukapuae* F.P.Cabrera & A.R.Sherwood *in* Cabrera *et al*. 2022: 574. Figs 2–13. [9] (S11 Fig. in S4 File)

Type locality: Kapou (Lisianski), Papahânaumokuâkea Marine National Monument, Hawai'i, U.S.A.

Holotype: BISH; BISH 780911 (25°52.94'N, 173°57,73'W; 15 September 2014; 84 m depth; *R. Pyle & S. Wagner*)

Isotypes: BISH; BISH 780912, 780913.

Japanese name: Kalaukapu-nori (new name).

Specimens examined: TNS AL-220761 (30 May 2019; *R*. *Terada*); TNS AL-220766 (30 May 2019; *R*. *Terada*; female plant).

Morpho-anatomical observation: Thalli were foliose, 10–10.5 cm in height, 6.0–6.5 cm in width, and rose-red in color, attached by small discoid holdfast with short stipe (S11 Fig. A, B). The blades were elliptical to obovate in shape. The blade margins were slightly undulated. The medulla blade was elongated and ruptured (S11 Fig. C). The cortex was composed of an outer cortex of compact cells composed of two small ellipsoidal cells and an inner cortex composed of two to three periclinally compressed cells (S11 Fig. D). Cystocarps were scattered over the blade surface (Fig. S11E). Mature cystocarps were loosely immersed within the medulla, slightly protruding through the blade, and bearing a thick pericarp penetrated by an ostiole (S11 Fig. F, G).

Remarks: The *rbc*L sequences of Japanese specimens are 0.2% divergent from the holotype of *C*. *kalaukapuae* from Hawai'i, U.S.A. ([OM621858](https://www.ncbi.nlm.nih.gov/nuccore/OM621858)), whereas the *cox*1 sequences are 1.2–1.5% divergent from the holotype and other specimens collected from Hawaii, U.S.A. ([OM509716](https://www.ncbi.nlm.nih.gov/nuccore/OM509716), [OM509718](https://www.ncbi.nlm.nih.gov/nuccore/OM509718)). We could not observe the structure of the medulla because the medulla was elongated and ruptured due to cystocarp production. Therefore, filamentous medulla and medullary stellate cells were not detected. The structures of cortex and cystocarps in Japanese specimens are essentially in accordance with those of Hawaiian *C*. *kalaukapuae* [9]. Although we could not observe the vegetative structure of the medulla, we identified these *Croisettea* specimens from Japan as *C*. *kalaukapuae* based on the low divergence of the *rbc*L and *cox*1 sequences between Japanese and Hawaiian specimens.

*Stenogramma guleopoense* M.S.Calderón & S.M.Boo 2014: 345. Figs 1–13. [10] (S12 Fig. in S4 File)

Type locality: Guleopdo Isle, Dukjeokdo Islands, South Korea.

Holotype: CNU; CNU014891 (37°11'20"N, 125°58'45" E; 4 August 1993; subtidal zone (depth of 5-10 m); *S.M. Boo*)

Isotypes: CNU; CNU014888-9, CNU14891-1, 2.

Specimens examined: TNS AL-209830 (18 May 2016; *R. Terada*; female plant); TNS AL-209831 (19 May 2016; *R. Terada*; female plant); TNS AL-209832, 209833 (20 July 2017; *R. Terada*; tetrasporophyte); TNS AL- 209834 (21 July 2017; *R*. *Terada*; tetrasporophyte); TNS AL-209835 (28 September 2017; *R. Terada*; female plant); TNS AL-209836 (25 May 2018; *R. Terada & M. Suzuki*); TNS AL-209837 (25 May 2018; *R. Terada & M. Suzuki*; tetrasporophyte); TNS AL-209838 (26 May 2018; *R. Terada & M. Suzuki*); TNS AL-215751 (15 May 2021; *R. Terada*); TNS AL-215752 (16 May 2021; *R. Terada*); TNS AL-222119 (2 October 2021; *R. Terada*); TNS AL-220768 (16 May 2022; *R. Terada*; tetrasporophyte).

Morpho-anatomical observation: The gametophytes and tetrasporophytes were isomorphic. Thalli arose from small discoid holdfasts, flat, membranous, and rose-red or bright-red in color, up to 13 cm in height (S12 Fig. A, B). The blades were dichotomously or subdichotomously branched five to seven times, with flabellate upper potions, and 2.0–7.0 mm in width (S12 Fig. A, B). Proliferations were abundantly produced from the margins of the blades. The thallus was solid, multiaxial, and composed of a thin outer cortex and inner medullary layers. The cortex was composed of one to two continuous cell layers. The medulla was composed of two to three incomplete layers of large cells, with one to two layers of smaller cells present between the cortex and medulla (S12 Fig. C, D). The cystocarps were developing inwardly, forming an interrupted, median thickened line (S12 Fig. E). The procarp consisted of a three-celled carpogonial branch with a sterile lateral from the basal cell (S12 Fig. F, H). The supporting cell bore a six-celled sterile branch. The postfertilization stage and mature carposporophyte was not observed. Male thalli were not observed. Tetrasporangial nemathecia were scattered as wart-like blotches over both blade surfaces (S12 Fig. G). The outer cortical cells produced rows of tetrasporangia (S12 Fig. I, J). Division of tetrasporangia were not observed.

Remarks: The *rbc*L sequences of Japanese specimens are 0.2% divergent from those of *S.* *guleopoense* from South Korea ([MF576328](https://www.ncbi.nlm.nih.gov/nuccore/MF576328)). Japanese specimens often produce many proliferations, whereas Korean *S*. *guleopoense* rarely or occasionally produces proliferations [10,11]. The basal cell of the carpogonial branch cuts off a one-celled sterile lateral in the Japanese specimen, whereas that of the Korean *S*. *guleopoense* cuts off a two-celled sterile lateral [10]. The relatively small size, slender blades, one to two layers of cortex, two to three layers of medulla, and one to two layers of small cells between the cortex and medulla in Japanese specimens are essentially in accordance with the Korean *S*. *guleopoense* [10,11]. Although the habits of Japanese specimens are somewhat different from those of Korean specimens, we identified these *Stenogramma* specimens from Japan as *S*. *guleopoense* based on the low divergence of *rbc*L sequences between Japanese and Korean specimens and characteristics of vegetative anatomy.

The Japanese *S*. *guleopoense* has been identified as *S*. *interruptum* (C. Agardh) Montagne. Molecular studies suggested that *S*. *interruptum* reported from Pacific Ocean was suggested to be segregated from the species [10-12]. Cho *et al*. [11] pointed out that *S*. *interruptum* in Japanese is similar to *S*. *coreanum* G.Y. Cho, I.K. Hwang, M.S. Calderon & S.M. Boo in size, number of dichotomous branches, and structure of cystocarps and nemathecia. Further investigations, including specimens collected from Honshu region, Japan, are required to identify ‘*S*. *interruptum*’ reported in Japan.

*Stenogramma lamyi* L.Le Gall *in* Le Gall *et al*. 2015: 196, 197. Figs 3–6. [13] (S13 Fig. in S4 File)

Type locality: Manantenina, Anosy Region, Madagascar.

Holotype: PC; PC 0142763 (24.76S, 47.2067E; 3 June 2010; 6 m depth; *L. Le Gall*)

Japanese name: Nankai-hasujigusa (new name).

Specimens examined: TNS AL-209842 (28 September 2017; *R. Terada*); TNS AL-215753 (26 June 2021; *R. Terada*); TNS AL-222091 (25 June 2021; *R. Terada*); TNS AL-220769, 222146 (16 May 2022; *R. Terada*); TNS AL-222097 (3 October 2021; *R. Terada*); TNS AL-222120 (2 October 2021; *R. Terada*); TNS AL-222185 (16 May 2022; *R. Terada*; tetrasporophyte); TNS AL-222217 (29 September 2022; *R. Terada*).

Morpho-anatomical observation: Thallus was arising from small discoid holdfasts, flat, membranous, and bright red in color, up to 10 cm in height (S13 Fig. A, B). The blades were dichotomously or subdichotomously branched two to five times, with flabellate upper potions, and 3.0–10 mm in width (S13 Fig. A, B). Proliferations were abundantly produced from the margins of the blades. The thallus was multiaxial, solid, and composed of a thin outer cortex and inner medullary layers. The cortex was composed of one to two continuous cell layers. The medulla was composed of one to three incomplete layers of large cells, with one to two layers of smaller cells present between the cortex and medulla (S13 Fig. C, D). The tetrasporangial nemathecia were scattered as wart-like blotches over both blade surfaces (Fig. S13E). The outer cortical cells produced rows of tetrasporangia (S13 Fig. F, G). Division of tetrasporangia were not observed.

Remarks: The *rbc*L sequences of Japanese specimens were identical to the holotype of *S*. *lamyi* from Madagascar ([KR733113](https://www.ncbi.nlm.nih.gov/nuccore/KR733113)), whereas the *cox*1 sequence was 0.6% divergent from the holotype ([KR733111](https://www.ncbi.nlm.nih.gov/nuccore/KR733111)). The Japanese specimens were larger than those of Malagasy *S*. *lamyi* [13]. The cortex of the Japanese specimens is composed of one to two layers of small cells, whereas the cortex of *S*. *lamyi* comprises one layer [11]. The medulla of the Japanese specimens is composed of one to three layers of large cells, whereas the medulla of *S*. *lamyi* comprises one layer [13]. Although the habits and vegetative anatomy of Japanese specimens are different from those of Malagasy specimens, we identified these *Stenogramma* specimens from Japan as *S*. *lamyi* based on the low divergence of *rbc*L and *cox*1 sequences between Japanese and Malagasy specimens. *Stenogramma lamyi* was described based on two collections and has not been recorded since its original description [13]. Further investigations, including more specimens from Madagascar and other regions in the Indian Ocean, are required to clarify the morphological gaps among the specimens. Herein, we provide the first record of a tetrasporophyte in this species. The structure of tetrasporangial nemathecia is essentially in accordance with that of other *Stenogramma* species [11,12,14,15].

*Yonagunia taiwani-borealis* Showe M.Lin, Y.C.Chuang & De Clerck *in* Lin *et al*. 2020: 1550. Figs 6, 7. [16] (S15 Fig. in S4 File)

Type locality: Chaojing, Keelung City, Taiwan.

Holotype: HAST; HAST 145307 (25.14°N, 121.80°E; 8 June 2009; rocky shore; *J.J. Hsueh & L.C. Liu*)

Isotypes: National Taiwan Ocean University; NTOU000988–NTOU001015.

Japanese name: Kita-yonaguniso (new name).

Specimens examined: TNS AL-214472 (18 May 2016; *R*. *Terada*); TNS AL-214473 (25 May 2018; *R. Terada & M. Suzuki*); TNS AL-214474 (30 May 2019; *R*. *Terada*); TNS AL-215816 (16 May 2021; *R. Terada*); TNS AL-222218 (29 September 2022; *R. Terada*).

Morpho-anatomical observation: Thalli were erect, 3.0–9.0 cm in height, rose-red in color, and cartilaginous in texture, (S15 Fig. A, B). Midribs were conspicuous in middle to basal part of axes (S15 Fig. C). The axes were flattened, subdichotomously branched, 2.0–3.0 mm wide, arising from short stipes (S15 Fig. C, D). Thalli were multiaxial and composed of cortex and inner medullary filaments. Cortex comprised four to five layers of oval to oblong cells, and medullary filaments were compactly arranged (S15 Fig. E–I). The cortex was unilaterally thickened in the middle to under part of thalli (S15 Fig. F, G). The reproductive organ was not observed.

Remarks: The *rbc*L sequences of Japanese specimens were identical to the holotype of *Y*. *taiwani-borealis* from Taiwan ([MT501503](https://www.ncbi.nlm.nih.gov/nuccore/MT501503)) and 0.1% divergent from *Y*. *taiwani-borealis* from Taiwan ([MT501502](https://www.ncbi.nlm.nih.gov/nuccore/MT501502)). The relatively long stipe, conspicuous midrib, subdichotomously branched axes, and thallus structures in Japanese specimens are essentially consistent with the Taiwanese *Y*. *taiwani-borealis* [16]. We identified these *Yonagunia* specimens from Japan as *Y*. *taiwani-borealis* based on the low divergence of *rbc*L sequences between Japanese and Taiwanese specimens and characteristics of habit and vegetative anatomy.

*Amalthea rubida* H.W.Lee & M.S.Kim *in* Lee *et al*. 2016: 343. Fig. 2 a–m. [17] (S16 Fig. in S4 File)

Type locality: Udo, Jeju Island, South Korea.

Holotype: JNUB; MSK150624-42 (25.14°N, 121.80°E; 24 June 2015; 17 m depth; tetrasporophyte)

Isotype: JNUB; MSK150624-40, NIBR; NIBRRD0000000184 (MSK150624-41).

Japanese name: Numeriginu (new name).

Specimens examined: TNS AL-220703 (20 July 2017; *R*. *Terada*); TNS AL-220704 (30 May 2019; *R. Terada*); TNS AL-222065 (16 May 2021; *R. Terada*).

Morpho-anatomical observation: Thalli were erect, solitary, foliose, 19–28 cm in height, 12–16.5 cm in width, pale-red or yellowish-red in color, and gelatinous in texture, attached by small discoid holdfast (S16 Fig. A, B). The blades were obovate with short, cuneate stipes. The blade margins were entire or slightly undulated. Thalli were multiaxial, composed of cortex and inner medullary filaments (S16 Fig. C, F). Cortex were composed of one-layered rounded cells and one- to two-layered subcortical cells, including stellate cells. The medulla was composed of loosely arranged anticlinal medullary filaments surrounded by medullary stellate cells (S16 Fig. D–F). The reproductive organ was not observed.

Remarks: The *rbc*L sequences of Japanese specimens were 0.2% divergent from the isotype of *A*. *rubida* from South Korea ([KX879776](https://www.ncbi.nlm.nih.gov/nuccore/KX879776)). The habit and loose arrangement of anticlinal filaments with medullary stellate cells in the medulla of Japanese specimens are consistent with the Korean *A*. *rubida* [17]. We identified these *Amalthea* specimens from Japan as *A*. *rubida* based on the low divergence of *rbc*L sequences between Japanese and Korean specimens and characteristics of habit and vegetative anatomy.

*Plocamium brasiliense* (Greville) M.Howe & W.R.Taylor 1931: 14. Figs 7, 8. [18] (S19 Fig. in S4 File)

Basionym: *Thamnophora brasiliensis* Greville *in* Saint-Hilaire 1833: 448. [19]

Type locality: Brazil.

Type: no information

Japanese name: Nankai-yukari (new name).

Specimens examined: TNS AL-209776 (18 May 2016; *R*. *Terada*); TNS AL-215770 (15 May 2021; *R. Terada*).

Morpho-anatomical observation: Thalli were erect (4.5–6.0 cm in height) and rose-red in color (S19 Fig. A, B). The axes were membranous, flattened, subdichotomously branched, up to 2.0 mm in width (S19 Fig. C). Upper branches produced alternating pairs of 2–3 ramuli (S19 Fig. D). Thalli were solid, uniaxial, and composed of a thin outer cortex and inner medulla. The cortex was composed of one layer of rounded or cuboidal cells. The medulla was composed of two to three layers of large cells (S19 Fig. E–K). The reproductive organ was not observed.

Remarks: The *rbc*L sequences of Japanese specimens are 0.6% divergent from *P*. *brasiliense* from Brazil ([KM974718](https://www.ncbi.nlm.nih.gov/nuccore/KM974718)). The habits of Japanese specimens are essentially in accordance with Brazilian *P*. *brasiliense* [18,20,21]. We identified these *Plocamium* specimens from Japan as *P*. *brasiliense* based on the low divergence of *rbc*L sequences between Japanese and Brazilian specimens and their habit characteristics.

Although the *rbc*L and *cox*1 analyses suggest that *P*. *brasiliense* is distinct from *P*. *luculentum* M.Y.Yang & M.S.Kim, the habit and vegetative anatomies of these species are similar to each other [22,this study]. According to Joly [21], the shapes of the tetrasporangial stichidia of *P*. *brasiliense* are similar to those of *P*. *luculentum* [22]. Both species were collected from offshore Tanegashima Island. Therefore, identification of Japanese *P*. *brasiliense* based on the morpho-anatomical characters typically used to distinguish species would be difficult.

References

1. Wynne MJ. The transfer of *Erythroglossum hyacinthinum* J.C.Kang & M.S.Kim to *Pseudopolyneura* (Delesseriaceae, Rhodophyta). Notulae Algarum 2016; 15: 1–2.
2. Kang JC, Kim MS. New red algal species, *Erythroglossum hyacinthinum* (Delesseriaceae, Rhodophyta) from Korea. Algae. 2014; 29: 1–13.
3. Harvey WH. Some account of the marine botany of the colony of western Australia. Trans R Irish Acad. 1855; 22: 525–566.
4. De Jong YSDM, Hitipeuw C, Prud'Homme van Reine WF. A taxonomic, phylogenetic and biogeographic study of the genus *Acanthophora* (Rhodomelaceae, Rhodophyta). Blumea. 1999; 44: 217–249.
5. Womersley HBS. The marine benthic flora of southern Australia Part IIID Ceramiales- Delesseriaceae, Sarcomeniaceae, Rhodomelaceae. Canberra & Adelaide: Australian Biological Resources Study & State Herbarium of South Australia; 2003.
6. Huisman JM. Algae of Australia. Marine Benthic Algae of North-western Australia, 2. Red Algae. Canberra & Melbourne: ABRS & CSIRO Publishing; 2018.
7. Perrone C, Cecere E, Furnari G. Growth pattern assessment in the genus *Acanthophora* (Rhodophyta, Ceramiales). Phycologia. 2006; 45: 37–43.
8. Paiano MO, Fumo JT, Cabrera FP, Kosaki RK, Spalding HL, Sherwood AR. *Calliblepharis yasutakei* sp. nov. and *Hypnea tsudae* sp. nov. (Cystocloniaceae, Rhodophyta): novel diversity from the Hawaiian Islands. Phytotaxa. 2022; 572: 74–86.
9. Cabrera FP, Huisman JM, Spalding HL, Kosaki RK, Smith CM, Sherwood AR Cryptic diversity in the genus *Croisettea* (Kallymeniaceae, Rhodophyta) from Hawaiian mesophotic reefs. Phycologia. 2022; 61: 572–583.
10. Calderón MS, Boo SM. A new species of phyllophoracean red algae (Gigartinales, Rhodophyta) from Korea: *Stenogramma guleopensis* sp. nov. Bot Mar. 2014; 57: 343–349.
11. Cho GY, Hwang IK, Calderon MS, Boo SM. 2018. Phylogenetic relationships of *Stenogramma* (Gigartinales, Rhodophyta) with a description of *S*. *coreanum* sp. nov.. Phycologia. 2018; 57: 243–250.
12. Le Gall L, Saunders GW. DNA barcoding is a powerful tool to uncover algal diversity: a case study of the Phyllophoraceae (Gigartinales, Rhodophyta) in the Canadian flora. J Phycol. 2010; 46: 374–389.
13. Le Gall L, Peña V, Gey D, Manghisi A, Dennetiere B, Reviers B de et al. A new species of *Stenogramma* was uncovered [in] Indian Ocean during the Mozangascar expedition Atimo Vatae: *Stenogramma lamyi* sp. nov.. Cryptogamie Algol. 2015; 36: 189–198.
14. Mikami H. A systematic study of the Phyllophoraceae and Gigartinaceae from Japan and its vicinity. Mem Fac Fish Hokkaido Univ. 1965; 5: 181–285.
15. Dixon P S, Irvine LM. Seaweeds of the British Isles. Volume 1. Rhodophyta. Part 1. Introduction, Nemaliales, Gigartinales. London: British Museum (Natural History); 1977.
16. Lin S-M, De Cleck O, Leliaert F, Chuang Y-C. Systematics and biogeography of the red algal genus *Yonagunia* (Halymeniaceae, Rhodophyta) from the Indo-Pacific including the description of two new species from Taiwan. J Phycol. 2020; 56: 1542–1556.
17. Lee HW, Yang MY, Kim MY. 2016. Verifying a new distribution of the genus *Amalthea* (Halymeniales, Rhodophyta) with description of *A*. *rubida* sp. nov. from Korea. Algae. 2016; 31: 341–349.
18. Howe MA, Taylor WR. Notes on new or little-known marine algae from Brazil. Brittonia 1931; 1: 7–33.
19. Saint-Hilaire A de. Voyage dans le district des diamans et sur le littoral du Brésil, suivi de note sur quelques plantes caractéristiques et d'un précis de l'historie des révolutions de l'empire brésilien, depuis le commencement du règne de Jean vi jusqu'à l'abdication de D. Pedro. Seconde Partie. Paris: Librarie-Gide; 1833.
20. Taylor WR. Marine algae of the eastern tropical and subtropical coasts of the Americas. Ann Arbor: The University of Michigan Press; 1960.
21. Joly AB. Flora marinha do litoral norte do estado de Saõ Paulo e regiões circunvizinhas. Bol Fac Filos Ciênc Let Univ São Paulo, Bot. 1965; 21: 5–393.
22. Yang MY, Kim MS. Cryptic diversity and phylogeographic patterns of *Plocamium telfairiae* and *P*. *cartilagineum* (Plocamiales, Rhodophyta) in the Northwest Pacific. Algae 2023; 38: 159–172.
