# Supplementary material for: DNA-based floristic survey of red algae (Rhodophyta) growing in the mesophotic coral ecosystems (MCEs) offshore of Tanegashima Island, northern Ryukyu Archipelago, Japan
Source: PLoS One. 2025 Mar 10;20(3):e0316067. doi: 10.1371/journal.pone.0316067 (PMC11893125; doi:10.1371/journal.pone.0316067)
Supplement: S7 File — (DOCX) [file pone.0316067.s007.docx]

**S48 Table. List of red algae from offshoreTanegashima Island and identification details.** C1: completely identified. The identification of species was supported by both molecular and morphological data. C2: completely identified. The identification of species was supported only by morphological data. C3: completely identified. The identification of species was supported only by molecular data. T: tentatively identified. The species was morphologically identified; however, molecular data revealed that it includes several cryptic species. U1: unidentified. The DNA sequences did not closely match the INSD data or matched sequences that were unidentified at the species or genus level. In addition, the specimens did not morphologically match any species recorded in Japan or its vicinity. U2: unidentified. The specimen was morphologically similar to a known species; however, molecular data indicated that it was distinct from a known species. U3: unidentified. The DNA sequences did not closely match the INSD data and did not exhibit distinguishable or reliable morphological characteristics.

| Species | Collection data (date; collector; voucher) and accession No. | Morpholoigcal identification | Top BLAST matches | Genetic identification | Accurate application of the species name; Remarks; Identification pattern (Id) |
| --- | --- | --- | --- | --- | --- |
| Nemaliophycidae, Nemaliales, Galaxauraceae | | | | | |
| *Dichotomaria latifolia* (Tak.Tanaka) S.Fontana, W.L.Wang & Sh.L.Liu (S1 Fig. in S4 File) | 15 May 2021; R. Terada; TNS AL-215728  *rbc*L: [LC821133](https://www.ncbi.nlm.nih.gov/nuccore/LC821133)  *cox*1: [LC820897](https://www.ncbi.nlm.nih.gov/nuccore/LC820897) | The habit and vegetative anatomy of this specimen were in accordance with the descriptions of *D*. *latifolia* in Japan and Taiwan [1,2]. | *rbc*L: *Dichotomaria* *latifolia* ([AY688021](https://www.ncbi.nlm.nih.gov/nuccore/AY688021), [PP003485](https://www.ncbi.nlm.nih.gov/nuccore/PP003485), [PP003486](https://www.ncbi.nlm.nih.gov/nuccore/PP003486); 99.9%)  *cox*1: *Dichotomaria* *latifolia* ([PP003717](https://www.ncbi.nlm.nih.gov/nuccore/PP003717); 99.4%) | The *rbc*L analyses suggested that this specimen forms a clade with a Taiwanese *D*. *latifolia* with full statistical supports (S23A Fig. in S5 File). The *rbc*L sequence was 0.1% divergent from Taiwanese *D*. *latifolia* ([AY688021](https://www.ncbi.nlm.nih.gov/nuccore/AY688021), [PP003485](https://www.ncbi.nlm.nih.gov/nuccore/PP003485), [PP003486](https://www.ncbi.nlm.nih.gov/nuccore/PP003486)). The *cox*1 analyses suggested that this specimen forms a clade with Taiwanese *D*. *latifolia* with full statistical supports (S23B Fig. in S5 File). The *cox*1 sequence was 0.6% divergent from *D*. *latifolia* ([PP003715](https://www.ncbi.nlm.nih.gov/nuccore/PP003715)–[PP003718](https://www.ncbi.nlm.nih.gov/nuccore/PP003718)). | This specimen was identified as *D*. *latifolia*. The identification was supported by both morphological and genetic identification.  Id: C1 |
| *Dichotomaria* sp. TNE (S1 Fig. in S4 File) | 15 May 2022; R. Terada; TNS AL-222169  *cox*1: [LC820898](https://www.ncbi.nlm.nih.gov/nuccore/LC820898) | The habit of this specimen was similar to those of *Dichotomaria* species with flattened thalli, such as *D*. *apiculata*, *D*. *elegans*, *D*. *falcata*, *D*. *latifolia*, and *D*. *marginata* that have been recorded in Japan, South Korea, Taiwan, Hawaii, and Australia [1-7]. | *cox*1: *Dichotomaria* “*marginata*” ([HQ422611](https://www.ncbi.nlm.nih.gov/nucleotide/HQ422611); 94.6%) | The *cox*1 sequence did not closely match the INSD data and was distant from various other *Dichotomaria* species, including *D*. *apiculata*, *D*. *elegans*, *D*. *falcata*, *D*. *latifolia*, and *D*. *marginata*. (S23B Fig. in S5 File). | We could not identify this specimen. Although this specimen was morphologically similar to *Dichotomaria* species with flattened thalli, the *cox*1 sequence was distant from all flattened species recorded in the Pacific.  Id: U3 |
| Nemaliophycidae, Nemaliales, Scinaiaceae | | | | | |
| *Scinaia hormoides* Setchell (S1 Fig. in S4 File) | 25 May 2018; R. Terada & M. Suzuki; TNS AL-209756  *rbc*L: [LC821135](https://www.ncbi.nlm.nih.gov/nuccore/LC821135)  *cox*1: [LC820900](https://www.ncbi.nlm.nih.gov/nuccore/LC820900) | The branches of this specimen were deeply constricted into ovoid segments. This characteristics are in accordance with *S*. *moniliformis* recorded in Japan and Australia [3, 6]. This specimen was also similar to *S*. *hormoides*; however it deffrent from *S*. *hormoides* which has globular segments [3, 5]. | *rbc*L: *Scinaia hormoides* ([KP238507](https://www.ncbi.nlm.nih.gov/nucleotide/KP238507); 98.8%)  *cox*1: *Scinaia hormoides* ([HQ422966](https://www.ncbi.nlm.nih.gov/nuccore/HQ422966); 99.1%) | The *rbc*L analyses suggested that this specimen forms a clade with *S*. *hormoides* from Taiwan with moderate to high statistical supports (77% BP and 1.00 PP: S24A Fig. in S5 File). The *rbc*L sequence was 1.2% divergent from Taiwanese *S*. *hormoides* ([KP238507](https://www.ncbi.nlm.nih.gov/nucleotide/KP238507), [LC821134](https://www.ncbi.nlm.nih.gov/nuccore/LC821134)). The *cox*1 analyses suggested that this specimen forms a clade with *S*. *hormoides* from Taiwan and Hawaii, U.S.A. with high statistical supports (98% BP and 1.00 PP; S24B Fig. in S5 File). The *cox*1 sequence was 0.9–1.1% divergent from the other two taxa ([HQ422966](https://www.ncbi.nlm.nih.gov/nuccore/HQ422966), [LC820899](https://www.ncbi.nlm.nih.gov/nuccore/LC820899)). | Although the morphology of this specimen similar to *S*. *moniliformis*, we identified it as *S*. *hormoides* based on the low divergence of the *rbc*L and *cox*1 sequences between Japanese and other specimens from Taiwan and Hawaii, U.S.A. Further morphological and molecular analyses, including the type specimen of *S*. *moniliformis* or with samples from the type locality (Victoria, Australia) are needed to clarify the identification of *S*. *hormoides* and *S*. *moniliformis* in Pacific.  Id: C3 |
| *Scinaia* sp.1 TNE (*S*. cf. *latifrons* M.Howe; S1 Fig. in S4 File) | 25 May 2018; R. Terada & M. Suzuki; TNS AL-209755  *rbc*L: [LC821137](https://www.ncbi.nlm.nih.gov/nuccore/LC821137)  *cox*1: [[LC820902](https://www.ncbi.nlm.nih.gov/nuccore/LC820902)](https://www.ncbi.nlm.nih.gov/nuccore/LC820902) | The blades of this specimen were flattened. This characteristics are in accordance with *S*. *latifrons* recorded in Japan and South Korea [3, 8]. | *rbc*L: *Scinaia* *latifrons* ([AB258449](https://www.ncbi.nlm.nih.gov/nuccore/AB258449); 94.7%)  *cox*1: *Scinaia japonica* ([LC820901](https://www.ncbi.nlm.nih.gov/nuccore/LC820901); 90.7%) | The *rbc*L analyses suggested that this specimen forms a clade with *S*. *latifrons* from Oita, Japan, with moderate to high statistical supports (83% BP and 1.00 PP; S24A Fig. in S5 File). The *rbc*L sequence was 5.3% divergent from the Japanese sequence assigned to *S*. *latifrons* ([AB258449](https://www.ncbi.nlm.nih.gov/nuccore/AB258449)). The *cox*1 sequence did not closely match the INSD data and was distant from various other *Scinaia* species. (S24B Fig. in S5 File). | We tentatively identified this specimen as *S*. cf. *latifrons*. Morphologically, the specimen was identified as *S*. *latifrons*. However, the *rbc*L analyses appeared that *S*. *latifrons* recorded in Japan includes two cryptic species. Further morpho-anatomical and molecular analyses, including the type specimen of *S*. *latifrons* or samples from a type locality (Baja California, México) are needed to clarify its identification.  Id: T |
| *Scinaia* sp.2 TNE (S1 Fig. in S4 File) | 25 May 2018; R. Terada & M. Suzuki; TNS AL-209760  *rbc*L: [LC821138](https://www.ncbi.nlm.nih.gov/nuccore/LC821138)  *cox*1: [LC820903](https://www.ncbi.nlm.nih.gov/nuccore/LC820903) | This specimen was morphologically similar to *Scinaia* species with cylindrical and small thalli, such as *S*. *chinensis*, *S*. *johnstoniae*, *S*. *okiensis*, and *S*. *pseudojaponica* recorded in the northwestern Pacific [3, 8-10]. The habits and vegetative anatomy of these species are similar to each other and *S*. *johnstoniae* and *S*. *okiensis* are distinguished by their male reproductive organs. However, our specimen did not have a reproductive organ. | *rbc*L: *Scinaia* *undulata* ([LT622873](https://www.ncbi.nlm.nih.gov/nuccore/LT622873); 90.6%)  *cox*1: *Scinaia moniliformis* ([MT946307](https://www.ncbi.nlm.nih.gov/nuccore/MT946307); 93.3%) | The *rbc*L and *cox*1 sequences did not closely match the INSD data and were distant from various other *Scinaia* species, including *S*. *chinensis*. (S24 Fig. in S5 File). | We could not identify this specimen because of a lack of close sequence data in INSD and lacked morphological key character. Further morpho-anatomical and molecular analyses, including additional specimens that produced reproductive organs and available sequences for *S*. *johnstoniae*, *S*. *okiensis*, and *S*. *pseudojaponica* are needed to clarify its identification.  Id: U3 |
| Rhodymeniophycidae, Bonnemaisoniales, Bonnemaisoniaceae | | | | | |
| *Delisea japonica* Okamura (S1 Fig. in S4 File) | 16 May 2021; R. Terada; TNS AL-215738  *rbc*L: [LC821142](https://www.ncbi.nlm.nih.gov/nuccore/LC821142)  *cox*1: [LC820907](https://www.ncbi.nlm.nih.gov/nuccore/LC820907) | The habit and vegetative anatomy of this specimen was in accordance with descriptions of *D*. *japonica* recorded in Japan and South Korea [3,8,11,12]. | *rbc*L: *Delisea japonica* ([LC821141](https://www.ncbi.nlm.nih.gov/nuccore/LC821141); 99.8%)  *cox*1: *Delisea japonica* ([LC820906](https://www.ncbi.nlm.nih.gov/nuccore/LC820906); 99.7%) | The *rbc*L sequence was 0.2% divergent from the Japanese sequence assigned to this species ([LC821141](https://www.ncbi.nlm.nih.gov/nuccore/LC821141)), whereas the *cox*1 sequence was 0.3% divergent from the Japanese sequence assigned to this species ([LC820906](https://www.ncbi.nlm.nih.gov/nuccore/LC820906)). (S25 Fig. in S5 File). | This specimen was identified as *D*. *japonica*. Morphological and genetic identifications support this identification. Lee [13] treated *D*. *japonica* as a synonym of *D*. *pulchra*. However, the *rbc*L and *cox*1 analyses suggested that *D*. *japonica* is distinct from *D*. *pulchra* from Taiwan and the Antarctic Peninsula ([U26812](https://www.ncbi.nlm.nih.gov/nuccore/U26812), [KY559726](https://www.ncbi.nlm.nih.gov/nuccore/KY559726)) and distant from various *Delisea* species  Id: C1 |
| Rhodymeniophycidae, Ceramiales, Callithamniaceae | | | | | |
| *Euptilota* sp. JP (S1 Fig. in S4 File) | 21 July 2017; R. Terada; TNS AL-209879  *rbc*L: [LC821143](https://www.ncbi.nlm.nih.gov/nuccore/LC821143)  *cox*1: [LC820908](https://www.ncbi.nlm.nih.gov/nuccore/LC820908) | The habit and vegetative anatomy of this specimen were in accordance with descriptions of *E*. *articulata* recorded in Japan and Australia [3, 14]. | *rbc*L: *Euptilota articulata* ([DQ022826](https://www.ncbi.nlm.nih.gov/nuccore/DQ022826); 94.6%)  *cox*1: *Aglaothamnion callophyllidicola* ([EU194955](https://www.ncbi.nlm.nih.gov/nuccore/EU194955); 88.2%) | The *rbc*L and *cox*1 sequences were 100% identical to *E*. *articulata* collected from Tokunoshima Island, Japan ([LC821144](https://www.ncbi.nlm.nih.gov/nuccore/LC821144), [LC820909](https://www.ncbi.nlm.nih.gov/nuccore/LC820909)). The *rbc*L analyses suggested that this specimen forms a clade with *E*. *articulata* from Victoria, Australia, with high statistical supports (95% BP and 1.00 PP; S26A Fig. in S5 File). The *rbc*L sequence was 5.4% divergent from Australian *E*. *articulata* ([DQ022826](https://www.ncbi.nlm.nih.gov/nuccore/DQ022826)). The *cox*1 sequence did not closely match the INSD data and was distant from various other callithamniacean genera (S26B Fig. in S5 File). | We could not identify this specimen. Morphologically, this specimen was identified as *E*. *articulata*. However, the *rbc*L analyses revealed that the Japanese specimens were distinct from the Australian *E*. *articulata*, suggesting *E*. *articulata* recorded in Japan appear to be different species. Further morpho-anatomical and molecular analyses, including more specimens from Japan, Australia, and the other parts of the Pacific, are needed to clarify its identification.  Id: U2 |
| Rhodymeniophycidae, Ceramiales, Ceramiaceae | | | | | |
| “*Ceramium*” *nakamurae* E.Y.Dawason (S1 Fig. in S4 File) | 20 July 2017; R. Terada; TNS AL-220685  *rbc*L: [LC821154](https://www.ncbi.nlm.nih.gov/nuccore/LC821154) | The branching pattern, and shape of branches and cortification of this specimen were similar to *Ceramium nakamurae* recorded in Japan, South Korea, and Taiwan [3, 15, 16]. | *rbc*L: *Ceramothamnion* sp. JFC-2019 ([MK125366](https://www.ncbi.nlm.nih.gov/nuccore/MK125366) as *Ceramium* sp.3; 95.3%) | The *rbc*L analyses suggested that this specimen is included in *Ceramothamnon* clade and distant from various other *Ceramothamnion* species. (S27A Fig. in S5 File). | Although the *rbc*L sequence did not closely match in INSD data, we identified this specimen as *C*. *nakamurae* because it was morphologically similar to *C*. *nakamurae*. This species is currently classified under *Ceramium*. However, *rbc*L analyses revealed that this species is included in the *Ceramothamnion* clade. Further morphological and molecular analyses are needed to clarify the genetic status.  Id: C2 |
| *Delesseriopsis elegans* Okamura (S1 Fig. in S4 File) | 26 May 2015; R. Terada; TNS AL-200146  *rbc*L: [LC821155](https://www.ncbi.nlm.nih.gov/nuccore/LC821155)  *cox*1: [LC820918](https://www.ncbi.nlm.nih.gov/nuccore/LC820918) | The habit and vegetative structure of this specimen were in accordance with descriptions of *D*. *elegans* recorded on offshore of Tanegashima Island and in Japan [3,15,17,18]. | *rbc*L: *Antithamnionella spirographidis* ([LC821147](https://www.ncbi.nlm.nih.gov/nuccore/LC821147); 88.1%)  *cox*1: *Pterothamnion crispum* ([KJ961054](https://www.ncbi.nlm.nih.gov/nuccore/KJ961054); 84.3%) | The *rbc*L and *cox*1 sequences did not closely match the INSD data and were distant from various other ceramiacean genera. (S27 Fig. in S5 File). | Although the *rbc*L and *cox*1 sequences did not closely match in INSD data, we identified this specimen as *D*. *elegans* because it was morphologically similar to *D*. *elegans*.  Id: C2 |
| *Pterothamnion* sp. TNE (*P*. cf. *yezoense*; S1 Fig. in S4 File) | 19 May 2016; R. Terada; TNS AL-220687  *rbc*L: [LC821159](https://www.ncbi.nlm.nih.gov/nuccore/LC821159)  *cox*1: [LC820921](https://www.ncbi.nlm.nih.gov/nuccore/LC820921) | The size, branching pattern, and shape of the branches of this specimen were in accordance with *P*. *yezoense* recorded in Japan and South Korea [3,15,19]. | *rbc*L: *Pterothamnion yezoense* ([GQ252494](https://www.ncbi.nlm.nih.gov/nuccore/GQ252494); 99.1%)  *cox*1: *Pterothamnion yezoense* ([EU194974](https://www.ncbi.nlm.nih.gov/nuccore/EU194974); 93.0%) | The *rbc*L analyses suggested that this specimen forms a clade with *P*. *pectinatum* from Washington, U.S.A., *P*. cf. *yezoense* from South Korea and *P*. sp. JP (*P*. cf. *yezoense*) from Kamakura, Japan, with high statistical supports (98% BP and 1.00 PP; S27A Fig. in S5 File). The *rbc*L sequence was 0.9–2.2% divergent from the other three taxa ([DQ787574](https://www.ncbi.nlm.nih.gov/nuccore/DQ787574), [GQ252494](https://www.ncbi.nlm.nih.gov/nuccore/GQ252494), [LC821158](https://www.ncbi.nlm.nih.gov/nuccore/LC821158)). The combined *rbc*L and *cox*1 analyses suggested that this specimen forms a clade with *P*. sp. JP (*P*. cf. *yezoense*) with full statistical supports (S27B Fig. in S5 File). The *cox*1 sequence is 8.4% divergent from *P*. sp. JP ([LC820920](https://www.ncbi.nlm.nih.gov/nuccore/LC820920)). The *cox*1 sequence (227 bp) was 7.0% divergent from Korean *P*. *yezoense* ([EU194974](https://www.ncbi.nlm.nih.gov/nuccore/EU194974)). | We tentatively identified this specimen as *P*. cf. *yezoense*. Morphologically, the specimen was identified as *P*. *yezoense*. However, *rbc*L and *cox*1 analyses revealed that *P*. *yezoense* recorded in Japan and South Korea includes two to three cryptic species. Further morphological and molecular analyses, including the type specimen of *P*. *yezoense* or samples from a type locality (Hokkaido, Japan) are needed to clarify its identification.  Id: T |
| Rhodymeniophycidae, Ceramiales, Delesseriaceae | | | | | |
| *Dasya* sp. TNE (S2 Fig. in S4 File) | 16 May 2022; R. Terada; TNS AL-222154  *rbc*L: [LC821165](https://www.ncbi.nlm.nih.gov/nuccore/LC821165)  *cox*1: [LC820926](https://www.ncbi.nlm.nih.gov/nuccore/LC820926) | The size and branching pattern of this specimen were similar to Dasyoideae species such as *Dasysiphonia sessilis* and *Dasya* spp. bearling short branches recorded in Japan, South Korea, and Australia [3, 7, 14, 16]; however, it lacked tetrasporangial stichidia, which is the key morphological characteristic of Dasyoideae species. | *rbc*L: *Dasya japonovillosa* ([LC635850](https://www.ncbi.nlm.nih.gov/nuccore/LC635850); 93.8%)  *cox*1: *Dasya pedicellata* ([ON002454](https://www.ncbi.nlm.nih.gov/nuccore/ON002454); 90.1%) | The *rbc*L and *cox*1 sequences did not closely match the INSD data and were distant from various other *Dasya* species (S28 and S29 Figs in S5 File). Further morphological and molecular analyses are needed to clarify the nomenclatural identity. | We could not identify this specimen because of the lack of tetrasporangial stichidia, and the sequences closely matched the INSD data. Further morpho-anatomical and molecular analyses, including additional specimens produced tetrasporangia, are needed to clarify its identification.  Id: U3 |
| Delesseriaceae sp.1 TNE (S2 Fig. in S4 File) | 16 May 2021; R. Terada; TNS AL-220666  *cox*1: [LC820928](https://www.ncbi.nlm.nih.gov/nuccore/LC820928) | The size and shape of blades of this specimen were similar to *Hypoglossum* species such as *H*. *barbatum* and *H*. *sagamianum* recorded in Japan, South Korea, and Hawaii [3, 5, 20]; however, it was different from them by the constrictions at the base of blades. | *cox*1: *Dasya tenuis* ([MW699764](https://www.ncbi.nlm.nih.gov/nuccore/MW699764); 88.7%) | The *cox*1 sequence did not closely match the INSD data and was distant from various other delesseriacean genera (S29B Fig. in S5 File). | We could not identify this specimen because of a lack of morphologically similar species, and *cox*1 analyses indicated that it did not belong to any delesseriacean genera available in INSD.  Id: U1 |
| Delesseriaceae sp.2 TNE (S2 Fig. in S4 File) | 26 June 2021; R. Terada; TNS AL-220667  *cox*1: [LC820929](https://www.ncbi.nlm.nih.gov/nuccore/LC820929) | This specimen was most morphologically similar to Delesseriaceae sp.1 TNE collected offshore Tanegashima Island; however it was larger and much branched. | *cox*1: *Phrix spatulata* ([LC311655](https://www.ncbi.nlm.nih.gov/nuccore/LC311655); 89.2%) | The *cox*1 analyses suggested that this specimen forms a clade with Delesseriaceae sp.1 TNE with full statistical supports (S29B Fig. in S5 File). The *cox*1 sequence was 2.8% divergent from Delesseriaceae sp.1 TNE ([LC820928](https://www.ncbi.nlm.nih.gov/nuccore/LC820928)). | We could not identify this specimen. Both the morphological characteristics and *cox*1 analyses indicated that this specimen is closely related to Delesseriaceae sp.1 TNE; however, the *cox*1 sequence divergence indicated that it is distinct from Delesseriaceae sp.1 TNE.  Id: U1 |
| “*Hypoglossum*” *nipponicum* Yamada (S3 Fig. in S4 File) | 15 May 2022; R. Terada; TNS AL-222181  *rbc*L: [LC821188](https://www.ncbi.nlm.nih.gov/nuccore/LC821188)  *cox*1: [LC820948](https://www.ncbi.nlm.nih.gov/nuccore/LC820948) | The entangled thallus and linear blades produced from the midrib and the position of the tetrasporangial sori on the blades of this specimen were similar to those of *Hypoglossum* *nipponicum* recorded in Japan [3,27]. | *rbc*L: *Hypoglossum sabahense* ([LC541575](https://www.ncbi.nlm.nih.gov/nuccore/LC541575); 92.3%)  *cox*1: *"Dasya" binghamiae* ([KX247283](https://www.ncbi.nlm.nih.gov/nuccore/KX247283); 87.5%) | The *rbc*L and *cox*1 sequences did not closely match the INSD data. The combined *rbc*L and *cox*1 analyses suggested that this specimen is included in the Sarcomenioideae clade with low to high statistical supports (less than 50% BP and 1.00 PP) and distant from various other delesseriacean genera (S29C Fig. in S5 File). | Although the *rbc*L and *cox*1 sequences did not closely match in the INSD data, we identified this specimen as *H*. *nipponicum* because it is morphologically in accordance with *H*. *nipponicum*. This species is currently classified under *Hypoglossum*. However, *rbc*L and *cox*1 analyses revealed that this species is not included in the *Hypoglossum* clade and is distant from various other delesseriacean genera. Further morphological and molecular analyses are needed to clarify the genetic status.  Id: C2 |
| “*Hypoglossum*” *serratifolium* Okamura (S2 Fig. in S4 File) | 25 May 2018; R. Terada & M. Suzuki; TNS AL-215842  *rbc*L: [LC821170](https://www.ncbi.nlm.nih.gov/nuccore/LC821170)  *cox*1: [LC820933](https://www.ncbi.nlm.nih.gov/nuccore/LC820933) | The linear blades and dentated margins of these specimens were in accordance with descriptions of *H*. *serratifolium* described in Japan [3,23] | *rbc*L: *Hypoglossum rhizophorum* ([MZ442337](https://www.ncbi.nlm.nih.gov/nuccore/MZ442337); 95.1%)  *cox*1: *Chauviniella coriifolia* ([JX111856](https://www.ncbi.nlm.nih.gov/nuccore/JX111856); 90.1%) | The *rbc*L and *cox*1 sequences did not closely match the INSD data and were distant from various other delesseriacean genera (S29 Fig. in S5 File). | Although the *rbc*L and *cox*1 sequences did not closely match in the INSD data, we identified this specimen as *H*. *serratifolium* because it is morphologically in accordance with *H*. *serratifolium*. This species is currently classified under *Hypoglossum*. However, *rbc*L and *cox*1 analyses revealed that this species is not included in the *Hypoglossum* clade and is distant from various other delesseriacean genera. Further morphological and molecular analyses are needed to clarify the genetic status.  Id: C2 |
|  | 16 May 2021; R. Terada; TNS AL-215844  *rbc*L: [LC821171](https://www.ncbi.nlm.nih.gov/nuccore/LC821171)  *cox*1: [LC820934](https://www.ncbi.nlm.nih.gov/nuccore/LC820934) |  |  |  |  |
| *Martensia* sp.1 TNE (S2 Fig. in S4 File) | 15 May 2021; R. Terada; TNS AL-222060  *rbc*L: [LC821173](https://www.ncbi.nlm.nih.gov/nuccore/LC821173)  *cox*1: [LC820936](https://www.ncbi.nlm.nih.gov/nuccore/LC820936) | The size and shape of the blade and single band of the networks of the specimen were similar to those of *M*. *tsudae* described in Hawaii [25]. | *rbc*L: *Martensia tsudae* ([MN164736](https://www.ncbi.nlm.nih.gov/nuccore/MN164736); 98.9%)  *cox*1: *Martensia jejuensis* ([LC821172](https://www.ncbi.nlm.nih.gov/nuccore/LC821172); 95.6%) | The *rbc*L analyses suggested that this specimen forms a clade with *M*. *jejuensis*, *M*. *lauhiekoeloa*, *M*. *tsudae*, and *M*. sp.2 TNE, with high statistical supports (91% BP and 1.00 PP; S30A Fig. in S5 File). The *rbc*L sequence was 1.1–1.6% divergent from the other four taxa ([MN164752](https://www.ncbi.nlm.nih.gov/nuccore/MN164752), [MN164736](https://www.ncbi.nlm.nih.gov/nuccore/MN164736), [LC821172](https://www.ncbi.nlm.nih.gov/nuccore/LC821172), [LC821174](https://www.ncbi.nlm.nih.gov/nuccore/LC821174)) The *cox*1 analyses suggested that this specimen forms a clade with *M*. *jejuensis*, *M*. *tsudae*, and *M*. sp.2 TNE with moderate to high statistical supports (93% BP and 0.98 PP; S30B Fig. in S5 File). The *cox*1 sequence was 4.6–5.9% divergent from the other three taxa ([MN164727](https://www.ncbi.nlm.nih.gov/nuccore/MN164727), [LC820935](https://www.ncbi.nlm.nih.gov/nuccore/LC820935), [LC820937](https://www.ncbi.nlm.nih.gov/nuccore/LC820937)). | We could not identify this specimen. Although the morphology and *rbc*L sequence of this species appear to be closely related to those of *M. tsudae*, the *cox*1 sequence appeared to be distant from *M*. *tsudae* and other *Martensia* species recorded in the northern Pacific.  Id: U2 |
| *Martensia* sp.2 TNE (S2 Fig. in S4 File) | 16 May 2021; R. Terada; TNS AL-222068  *rbc*L: [LC821174](https://www.ncbi.nlm.nih.gov/nuccore/LC821174)  *cox*1: [LC820937](https://www.ncbi.nlm.nih.gov/nuccore/LC820937) | This specimen was morphologically similar to *Martensia* sp.1 TNE collected offshore Tanegashima Island. | *rbc*L: *Martensia tsudae* ([MN164736](https://www.ncbi.nlm.nih.gov/nuccore/MN164736); 98.7%)  *cox*1: *Martensia tsudae* ([MN164727](https://www.ncbi.nlm.nih.gov/nuccore/MN164727); 94.6%) | The *rbc*L analyses suggested that this specimen forms a clade with *M*. *jejuensis*, *M*. *lauhiekoeloa*, *M*. *tsudae*, and *M*. sp.1 TNE with high statistical supports (91% BP and 1.00 PP; S30A Fig. in S5 File). The *rbc*L sequence is 1.3–1.9% divergent from the other four taxa ([MN164752](https://www.ncbi.nlm.nih.gov/nuccore/MN164752), [MN164736](https://www.ncbi.nlm.nih.gov/nuccore/MN164736), [LC821172](https://www.ncbi.nlm.nih.gov/nuccore/LC821172), [LC821173](https://www.ncbi.nlm.nih.gov/nuccore/LC821173)). The *cox*1 analyses suggested that this specimen forms a clade with *M*. *jejuensis*, *M*. *tsudae*, and *M*. sp.1 TNE with moderate to high statistical supports (93% BP and 0.98 PP; S30B Fig. in S5 File). The *cox*1 sequence is 5.4–6.9% divergent from the other three taxa ([MN164727](https://www.ncbi.nlm.nih.gov/nuccore/MN164727), [LC820935](https://www.ncbi.nlm.nih.gov/nuccore/LC820935), [LC820936](https://www.ncbi.nlm.nih.gov/nuccore/LC820936)). | We could not identify this specimen. Both the morphological characteristics and molecular analyses indicated that this specimen was closely related to *M*. sp.1 TNE. The *cox*1 sequence appeared to be distant from *M*. sp.1 TNE and other *Martensia* species recorded in the northern Pacific.  Id: U2 |
| Nitophylloideae sp.1 TNE (S2 Fig. in S4 File) | 16 May 2022; R. Terada; TNS AL-222145  *rbc*L: [LC821175](https://www.ncbi.nlm.nih.gov/nuccore/LC821175)  *cox*1: [LC820938](https://www.ncbi.nlm.nih.gov/nuccore/LC820938) | The membranous, roundish blades with an entire margin and lack of conspicuous stipe in this specimen were not similar to any delesseriacean species recorded in the Pacific [3,5,7,20,24]. | *rbc*L: *Nitophyllum adhaerens* ([AF257399](https://www.ncbi.nlm.nih.gov/nuccore/AF257399); 90.9%)  *cox*1: *Nitophyllum adhaerens* ([HQ422841](https://www.ncbi.nlm.nih.gov/nuccore/HQ422841); 87.0%) | The *rbc*L analyses suggested that this specimen forms a clade with “*Nitophyllum*” *adhaerens* M.J.Wynne with full statistical supports (S30A Fig. in S5 File). The *rbc*L sequence was 8.9% divergent from “*N*.” *adhaerens* from Texas, U.S.A. ([AF257399](https://www.ncbi.nlm.nih.gov/nuccore/AF257399)). The *cox*1 analyses suggested that this specimen forms a clade with “*N*.” *adhaerens* with low to high statistical supports (100% BP and 0.94 PP; S30B Fig. in S5 File). The *cox*1 sequence was 12.9% divergent from “*N*.” *adhaerens* from Hawaii, U.S.A. ([HQ422841](https://www.ncbi.nlm.nih.gov/nuccore/HQ422841)). | We could not identify this specimen because of a lack of morphologically similar species, and molecular analyses indicated that it did not belong to any delesseriacean genera available in INSD.  Id: U1 |
| Nitophylloideae sp.2 TNE (S2 Fig. in S4 File) | 25 June 2021; R. Terada; TNS AL-222090  *rbc*L: [LC821176](https://www.ncbi.nlm.nih.gov/nuccore/LC821176)  *cox*1: [LC820939](https://www.ncbi.nlm.nih.gov/nuccore/LC820939) | This specimen was morphologically similar to Nitophylloideae sp.1 TNE collected from offshore Tanegashima Island; however, it is larger than Nitophylloideae sp.1 TNE. | *rbc*L: *Nitophyllum adhaerens* ([AF257399](https://www.ncbi.nlm.nih.gov/nuccore/AF257399); 91.4%)  *cox*1: *Nitophyllum adhaerens* ([HQ422841](https://www.ncbi.nlm.nih.gov/nuccore/HQ422841); 86.8%) | The combined *rbc*L and *cox*1 analyses suggested that this specimen forms a clade with Nitophylloideae sp.1 TNE with full statistical supports (S29C Fig. in S5 File). The *rbc*L sequence was 1.0% divergent from Nitophylloideae sp.1 TNE ([LC821175](https://www.ncbi.nlm.nih.gov/nuccore/LC821175)), whereas the *cox*1 sequence was 5.4% divergent from Nitophylloideae sp.1 TNE ([LC820938](https://www.ncbi.nlm.nih.gov/nuccore/LC820938)). | We could not identify this specimen. Both the morphological characteristics and molecular analyses indicated that this specimen is closely related to Nitophylloideae sp.1 TNE; however, the *cox*1 sequence divergence indicated that it is distinct from Nitophylloideae sp.1 TNE.  Id: U1 |
| *Nitophyllum* sp. TNE (S2 Fig. in S4 File) | 25 June 2021; R. Terada; TNS AL-222074  *rbc*L: [LC821177](https://www.ncbi.nlm.nih.gov/nuccore/LC821177)  *cox*1: [LC820940](https://www.ncbi.nlm.nih.gov/nuccore/LC820940) | This specimen was membranous, subdivided into three blades, and produced proliferations from the margins. These characteristics are not similar to those of any delesseriacean species recorded in the Pacific [3,5,7,20,24]. | *rbc*L: *Nitophyllum hommersandii* ([AY118270](https://www.ncbi.nlm.nih.gov/nuccore/AY118270); 94.6%)  *cox*1: *Nitophyllum* sp. MSK-2015 ([KP019299](https://www.ncbi.nlm.nih.gov/nuccore/KP019299); 86.1%) | The *rbc*L analyses suggested that this specimen forms a clade with *N*. *hommersandii* with high statistical supports (95% BP and 1.00 PP; S30A Fig. in S5 File). The *rbc*L sequence was 5.4% divergent from *N*. *hommersandii* from Taiwan ([AY118270](https://www.ncbi.nlm.nih.gov/nuccore/AY118270)). The *cox*1 analyses suggested that this specimen forms a clade with *N*. *punctatum* and *N*. sp. MSK-2015 from South Korea with moderate statistical supports (75% BP and 0.98 PP; S30B Fig. in S5 File). The *cox*1 sequence was 13.7–15.6% divergent from the other two taxa ([KY682898](https://www.ncbi.nlm.nih.gov/nuccore/KY682898), [KP019299](https://www.ncbi.nlm.nih.gov/nuccore/KP019299)). | We could not identify this specimen because of the lack of similar morphological species and close sequence data in INSD. The molecular analyses indicated that this species belongs to *Nitophyllum*; however, it morphologically does not match any *Nitophyllum* species recorded in the Pacific.  Id: U1 |
| Phycodryoideae sp.1 TNE (S3 Fig. in S4 File) | 4 June 2022; R. Terada; TNS AL-222212  *rbc*L: [LC821179](https://www.ncbi.nlm.nih.gov/nuccore/LC821179)  *cox*1: [LC820942](https://www.ncbi.nlm.nih.gov/nuccore/LC820942) | The membranous and slightly conspicuous veins on the blade of this specimen were morphologically similar to those of *Schizoseris* species recorded in the Pacific [3,20,24] and most similar to *S*. *hymenena* recorded in Tasmania, Australia [24]. | *rbc*L: *Neuroglossum binderianum* ([AF257395](https://www.ncbi.nlm.nih.gov/nuccore/AF257395); 91.5%)  *cox*1: Delesseriaceae sp. LLG-2014 ([KJ960650](https://www.ncbi.nlm.nih.gov/nuccore/KJ960650); 89.0%) | The *rbc*L and *cox*1 sequences did not closely match the INSD data. The *rbc*L analyses suggested that this specimen forms a large clade comprising *Drachiella* spp., *Myriogramme multinervis*, Phycodryoideae spp., and *Schizoseris* spp. with high statistical supports (82% BP and 1.00 PP; S31A Fig. in S5 File). The *cox*1 analyses suggested that this specimen forms a large clade comprising Delesseriaceae sp. LLG-2014, *M*. *smithii*, Phycodryoideae spp., and *Schizoseris* spp. with low to high statistical supports (59% BP and 1.00 PP; S31B Fig. in S5 File). | Although this specimen was morphologically similar to *S. hymenena*, we could not identify it as *S. hymenena* because of the lack of available sequence data for *S*. *hymenena* in the INSD, no records of this species have been found in the northern Pacific, and molecular analyses have revealed that its genetic status in the Phycodryoideae is uncertain.  Id: U3. |
| Phycodryoideae sp.2 TNE (S3 Fig. in S4 File) | 3 October 2021; R. Terada; TNS AL-222110  *rbc*L: [LC821180](https://www.ncbi.nlm.nih.gov/nuccore/LC821180) | The membranous and veins on the blade of this specimen were morphologically similar to those of *Acrosorium* species recorded in the Pacific [3,20,24] and most similar to *A*. *flabellatum* or *A*. *polyneurum* recorded in Japan and South Korea [3,20]. | *rbc*L: *Drachiella liaoi* ([AF257448](https://www.ncbi.nlm.nih.gov/nuccore/AF257448); 91.1%) | However, *rbc*L analyses suggested that this specimen is excluded from the *Acrosorium* clade and forms a clade with Phycodryoideae sp.1 TNE collected from offshore Tanegashima Island with high statistical supports (91% BP and 1.00 PP; S31A Fig. in S5 File). The *rbc*L sequences was 7.6% divergent from Phycodryoideae sp.1 TNE ([LC821179](https://www.ncbi.nlm.nih.gov/nuccore/LC821179)). Further morphological and molecular analyses are needed to clarify the nomenclatural identity. | We could not identify this specimen. Although it was morphologically similar to *Acrosorium*, the *rbc*L analyses appeared that it is excluded from the *Acrosorium* clade, including *A*. *flabellatum* and *A*. *polyneurum*, and is distant from the other genera.  Id: U3. |
| Phycodryoideae sp.3 TNE (S3 Fig. in S4 File) | 16 May 2021; R. Terada; TNS AL-220684  *rbc*L: [LC821181](https://www.ncbi.nlm.nih.gov/nuccore/LC821181) | The decumbent membranous blades of this specimen were most similar to *Drachiella* *liaoi*, the only species recorded in the northwestern Pacific [26]. | *rbc*L: *Drachiella liaoi* ([AF257448](https://www.ncbi.nlm.nih.gov/nuccore/AF257448); 96.4%) | The *rbc*L analyses suggested that this specimen forms a clade with *D*. *liaoi* and Phycodoryoideae sp.5 TNE collected from offshore Tanegashima Island with full statistical supports (100% BP and 1.00 PP), whereas this clade is distinct from *D*. *spectabilis*, which is the type species of the genus *Drachiella* (S31A Fig. in S5 File). The *rbc*L sequence was 1.1–3.6% divergent from the other two taxa ([AF257448](https://www.ncbi.nlm.nih.gov/nuccore/AF257448), [LC821183](https://www.ncbi.nlm.nih.gov/nuccore/LC821183)). | We could not identify this specimen. Although the morphology of this specimen was similar to *D*. *liaoi*, *rbc*L sequence divergence indicated that it is distinct from *D*. *liaoi*.  Id: U2 |
| Phycodryoideae sp.4 TNE (S3 Fig. in S4 File) | 16 May 2022; R. Terada; TNS AL-222160  *rbc*L: [LC821182](https://www.ncbi.nlm.nih.gov/nuccore/LC821182)  *cox*1: [LC820943](https://www.ncbi.nlm.nih.gov/nuccore/LC820943) | This specimen was morphologically similar to Phycodryoideae sp.3 TNE collected from offshore Tanegashima Island. | *rbc*L: *Drachiella liaoi* ([AF257448](https://www.ncbi.nlm.nih.gov/nuccore/AF257448); 92.7%)  *cox*1: *Myriogramme smithii* ([KY559926](https://www.ncbi.nlm.nih.gov/nuccore/KY559926); 92.4%) | The *rbc*L analyses suggested that this specimen forms a clade with Phycodryoideae spp. and *D*. *liaoi* with high statistical supports (96% BP and 1.00 PP; S31A Fig. in S5 File). The *rbc*L sequence was 7.3–9.9% divergent from the other five taxa ([AF257448](https://www.ncbi.nlm.nih.gov/nuccore/AF257448), [LC821179](https://www.ncbi.nlm.nih.gov/nuccore/LC821179), [LC821180](https://www.ncbi.nlm.nih.gov/nuccore/LC821180), [LC821181](https://www.ncbi.nlm.nih.gov/nuccore/LC821181), [LC821183](https://www.ncbi.nlm.nih.gov/nuccore/LC821183)). The *cox*1 analyses suggested that this specimen forms a clade with Phycodryoideae sp. 1 TNE with high statistical supports (84% BP and 1.00 PP; S31B Fig. in S5 File). The *cox*1 sequence was 8.3% divergent from Phycodryoideae sp.1 TNE ([LC820942](https://www.ncbi.nlm.nih.gov/nuccore/LC820942)). | We could not identify this specimen. Both the morphological characteristics and molecular analyses indicated that this specimen is closely related to *D*. *liaoi* and Phycodryoideae sp.3 TNE; however, the *rbc*L sequence divergence indicated that it is distinct from *D*. *liaoi* and Phycodryoideae sp.3 TNE.  Id: U2 |
| Phycodryoideae sp.5 TNE (S3 Fig. in S4 File) | 5 June 2022; R. Terada; TNS AL-222166  *rbc*L: [LC821183](https://www.ncbi.nlm.nih.gov/nuccore/LC821183) | This specimen was morphologically similar to Phycodryoideae sp.3 TNE collected from offshore Tanegashima Island. | *rbc*L: *Drachiella liaoi* ([AF257448](https://www.ncbi.nlm.nih.gov/nuccore/AF257448); 96.5%) | The *rbc*L analyses suggested that this specimen forms a clade with Phycodryoideae sp. 3 TNE with full statistical supports (S31A Fig. in S5 File). The *rbc*L sequence is 1.1% divergent from Phycodryoideae sp.3 TNE ([LC821181](https://www.ncbi.nlm.nih.gov/nuccore/LC821181)). | We could not identify this specimen. Both the morphological characteristics and molecular analyses indicated that this specimen is closely related to *D*. *liaoi* and Phycodryoideae sp.3 TNE; however, the *rbc*L sequence divergence indicated that it is distinct from *D*. *liaoi* and Phycodryoideae sp.3 TNE.  Id: U2 |
| *Pseudopolyneura hyacinthina* (J.C.Kang & M.S.Kim) M.J.Wynne (S4 Fig. in S4 File and S6 File) | 3 October 2021; R. Terada; TNS AL-222139  *rbc*L: [LC821184](https://www.ncbi.nlm.nih.gov/nuccore/LC821184)  *cox*1: [LC820944](https://www.ncbi.nlm.nih.gov/nuccore/LC820944) | This species is a new record for Japan. The details of the morpho-anatomical observations and identification of these specimens are presented in S6 File. | *rbc*L: *Pseudopolyneura hyacinthina* ([KF305299](https://www.ncbi.nlm.nih.gov/nuccore/KF305299); 100%)  *cox*1: *Polyneura latissima* ([HQ544165](https://www.ncbi.nlm.nih.gov/nuccore/HQ544165); 96.5%) | The *rbc*L sequences were 100% identical to the holotype of *P*. *hyacinthina* collected from South Korea ([KF305299](https://www.ncbi.nlm.nih.gov/nuccore/KF305299); S31A Fig. in S5 File). The *cox*1 sequences did not closely match the INSD data. | This specimen was identified as *P*. *hyacinthina*. The identification was supported by both morphological and genetic identification.  Id: C1 |
|  | 15 May 2022; R. Terada; TNS AL-222180  *rbc*L: [LC821185](https://www.ncbi.nlm.nih.gov/nuccore/LC821185)  *cox*1: [LC820945](https://www.ncbi.nlm.nih.gov/nuccore/LC820945) |  |  |  |  |
|  | 4 June 2022; R. Terada; TNS AL-222210  *rbc*L: [LC821186](https://www.ncbi.nlm.nih.gov/nuccore/LC821186)  *cox*1: [LC820946](https://www.ncbi.nlm.nih.gov/nuccore/LC820946) |  |  |  |  |
| “*Sorella*” *pulchra* (Yamada) T.Yoshida & Mikami (S2 Fig. in S4 File) | 18 May 2016; R. Terada; TNS AL-220665  *rbc*L: [LC821168](https://www.ncbi.nlm.nih.gov/nuccore/LC821168)  *cox*1: [LC820931](https://www.ncbi.nlm.nih.gov/nuccore/LC820931) | The size, branching pattern, and shape of branches were in accordance with *Sorella pulchra* described in Japan [3, 21] | *rbc*L: *Erythroglossum minimum* ([KF305282](https://www.ncbi.nlm.nih.gov/nuccore/KF305282); 99.1%)  *cox*1: *Erythroglossum latum* ([LC820930](https://www.ncbi.nlm.nih.gov/nuccore/LC820930); 97.7%) | The *rbc*L analyses suggested that this specimen forms a clade with *E*. *minimum* from South Korea with full statistical supports (S31A Fig. in S5 File). The *rbc*L sequence is 0.9% divergent from *E*. *minimum* ([KF305282](https://www.ncbi.nlm.nih.gov/nuccore/KF305282)). The *cox*1 analyses suggested that this specimen forms a clade with *E*. *latum* with moderate statistical supports (73% BP and 0.96 PP; S31B Fig. in S5 File). The *cox*1 sequence is 2.3% divergent from *E*. *latum* ([LC820930](https://www.ncbi.nlm.nih.gov/nuccore/LC820930)). | Although the *rbc*L and *cox*1 sequences did not closely match in the INSD data, we identified this specimen as *S*. *pulchra* because it is morphologically in accordance with *S*. *pulchra*. This species is currently classified under *Sorella*. However, *rbc*L and *cox*1 analyses revealed that this species is not included in the *Erythroglossum* clade. The *rbc*L analyses indicated that it is conspecific with Korean *E*. *minimum*. However, this specimen was morphologically different from *E*. *minimum*. This specimen branched more than three times and was less than 1.5 cm high, whereas *E*. *minimum*, branched one to two times, and reached 2.5 cm high [3,22]. Further morphological and molecular analyses are needed to clarify the genetic status.  Id: C2 |
| *Sympodothamnion leptophyllum* (Tak.Tanaka) Itono (S3 Fig. in S4 File) | 26 May 2015; R. Terada; TNS AL-200169  *rbc*L: [LC821189](https://www.ncbi.nlm.nih.gov/nuccore/LC821189)  *cox*1: [LC820949](https://www.ncbi.nlm.nih.gov/nuccore/LC820949) | The habit and vegetative structure of this specimen were in accordance with descriptions of *S*. *leptophyllum* recorded on offshore of Tanegashima Island, Japan [3,18]. | *rbc*L: *Acrosorium ciliolatum* ([MF093964](https://www.ncbi.nlm.nih.gov/nuccore/MF093964); 90.1%)  *cox*1: *Hemineura frondosa* ([HM918210](https://www.ncbi.nlm.nih.gov/nuccore/HM918210); 89.5%) | The *rbc*L and *cox*1 sequences did not closely match the INSD data. The combined *rbc*L and *cox*1 analyses suggested that this specimen is positioned sister to the Phycodryoideae clade, with moderate to high statistical supports (83% BP and 1.00 PP; S29 A, C Fig. in S5 File). | Although the *rbc*L and *cox*1 sequences did not closely match the INSD data, we identified this specimen as *S*. *leptophyllum* morphologically in accordance with the *S*. *leptophyllum* was described in the offshore Tanegashima Islands. This species was described on offshore Tanegashima Island (as Mageshima Island) and has not been recorded since its original description [3, 18]. This species is currently classified as Dasyoideae [3 as Dasyaceae]; however, molecular analyses revealed that it is related to the Phycodryoideae.  Id: C2 |
| “*Vanvoorstia*” *coccinea* Harvey ex J.Agardh (S3 Fig. in S4 File) | 19 May 2016; R. Terada; TNS AL-209889  *rbc*L: [LC821190](https://www.ncbi.nlm.nih.gov/nuccore/LC821190)  *cox*1: [LC820950](https://www.ncbi.nlm.nih.gov/nuccore/LC820950) | The net-like blades and corticated axes of this specimen were in accordance with *V*. *coccinea* recorded in Japan and Hawaii [3,5] | *rbc*L: *Hypoglossum rhizophorum* ([MZ442337](https://www.ncbi.nlm.nih.gov/nuccore/MZ442337); 92.3%)  *cox*1: *Dasya pedicellata* ([ON002454](https://www.ncbi.nlm.nih.gov/nuccore/ON002454); 87.7%) | The *rbc*L and *cox*1 sequences did not closely match the INSD data and were distant from various other delesseriacean genera (S29 Fig. in S5 File). | Although the *rbc*L and *cox*1 sequences did not closely match in the INSD data, we identified this specimen as *V*. *coccinea* because it is morphologically in accordance with *V*. *coccinea*. This species is currently classified under *Vanvoorstia*. However, *rbc*L analyses revealed that this species is distant from *V*. *spectabilis*, the type species of *Vanvoorstia* and various other delesseriacean genera. Further morphological and molecular analyses are needed to clarify the genetic status.  Id: C2 |
| *Yoshidaphycus ciliatus* (Okamura) Mikami (S3 Fig. in S4 File) | 28 September 2017; R. Terada; TNS AL-215852  *rbc*L: [LC821191](https://www.ncbi.nlm.nih.gov/nuccore/LC821191)  *cox*1: [LC820951](https://www.ncbi.nlm.nih.gov/nuccore/LC820951) | The membranous and linear blades, cortificated midrib of these specimens were in accordance with descriptions of *Y*. *ciliates* recorded in Japan [3,28 as *Branchioglossum ciliatum*]. | *rbc*L: *Sarcomenia delesserioides* ([AF257443](https://www.ncbi.nlm.nih.gov/nuccore/AF257443); 90.6%)  *cox*1: *Delesseria sanguinea* ([JX111865](https://www.ncbi.nlm.nih.gov/nuccore/JX111865); 88.7%) | The *rbc*L and *cox*1 sequences did not closely match the INSD data and were distant from various other delesseriacean genera (S29 Fig. in S5 File). | Although the *rbc*L and *cox*1 sequences did not closely match the INSD data, we identified this specimen as *Y*. *ciliates* morphologically in accordance with the *Y*. *ciliates* was described in Japan. Tanegashima Island. This species is currently classified as Delleserioideae [29]; however, molecular analyses revealed that it is related to the Sarcomenioideae.  Id: C2 |
|  | 25 June 2021; R. Terada; TNS AL-222089  *rbc*L: [LC821192](https://www.ncbi.nlm.nih.gov/nuccore/LC821192)  *cox*1: [LC820952](https://www.ncbi.nlm.nih.gov/nuccore/LC820952) |  |  |  |  |
| Zinovaeeae sp. TNE (S3 Fig. in S4 File) | 25 June 2021; R. Terada; TNS AL-220678  *rbc*L: [LC821193](https://www.ncbi.nlm.nih.gov/nuccore/LC821193)  *cox*1: [LC820953](https://www.ncbi.nlm.nih.gov/nuccore/LC820953) | This specimen produced stichidium-like bladelets, which are characteristic of *Kurogia pulchra* and *Zinovaea acanthocarpa* belonging to the tribe Zinovaeeae. Both species are distributed in the subarctic region, and their habits differ from those of this specimen. The habits and stichidium-like bladeles of this specimen are not identical to those of any delesseriacean species recorded in the Pacific [3,5,7,20,24]. | *rbc*L: *Vanvoorstia spectabilis* ([AF257456](https://www.ncbi.nlm.nih.gov/nuccore/AF257456); 93.4%)  *cox*1: *Chauviniella coriifolia* ([JX111856](https://www.ncbi.nlm.nih.gov/nuccore/JX111856); 89.7%) | The *rbc*L and *cox*1 sequences did not closely match the INSD data and were distant from various other delesseriacean genera (S29 Fig. in S5 File). | We could not identify this specimen because of the lack of similar morphological species and close sequence data in INSD.  Id: U1 |
| Rhodymeniophycidae, Ceramiales, Rhodomelaceae | | | | | |
| Acanthophora dendroides Harvey (S5 Fig. in S4 File and S6 File) | 15 May 2022; R. Terada; TNS AL-222201  *rbc*L: [LC821194](https://www.ncbi.nlm.nih.gov/nuccore/LC821194)  *cox*1: [LC820954](https://www.ncbi.nlm.nih.gov/nuccore/LC820954) | This species is a new record for Japan. The details of the morpho-anatomical observations and identification of this specimen is presented in S7 File. | *rbc*L: Acanthophora dendroides ([MT876666](https://www.ncbi.nlm.nih.gov/nuccore/MT876666); 99.6%)  *cox*1: Acanthophora dendroides ([MH388739](https://www.ncbi.nlm.nih.gov/nuccore/MH388739); 99.0%) | The *rbc*L analyses suggested that this specimen forms a clade with *A*. *dendroides* from India, with moderate to high statistical supports (61% BP and 1.00 PP; S33A Fig. in S5 File). The *rbc*L sequence was 0.4–0.9% divergent from *A*. *dendroides* from India, Philippines, and Florida, U.S.A. ([MH388514](https://www.ncbi.nlm.nih.gov/nuccore/MH388514), [MH388560](https://www.ncbi.nlm.nih.gov/nuccore/MH388560), [MH388561](https://www.ncbi.nlm.nih.gov/nuccore/MH388561), [MT876666](https://www.ncbi.nlm.nih.gov/nuccore/MT876666)). The *cox*1 analyses suggested that these specimens form a clade with *A*. *dendroides* from India, Philippines, and Florida, U.S.A. with high statistical supports (86% BP and 1.00 PP; S33B Fig. in S5 File). The *cox*1 sequence is 1.0–2.4% divergent from the other four taxa ([MH388702](https://www.ncbi.nlm.nih.gov/nuccore/MH388702), [MH388737](https://www.ncbi.nlm.nih.gov/nuccore/MH388737), [MH388739](https://www.ncbi.nlm.nih.gov/nuccore/MH388739), [MT876665](https://www.ncbi.nlm.nih.gov/nuccore/MT876665)). | This specimen was identified as *A*. *dendroides*. The identification was supported by both morphological and genetic identification.  Id: C1 |
| Amansieae sp. TNE (S6 Fig. in S4 File) | 18 May 2016; R. Terada; TNS AL-222045  *cox*1: [LC820956](https://www.ncbi.nlm.nih.gov/nuccore/LC820956) | This specimen was morphologically similar to *Enantiocladia okamurae* described in Japan [3,27]; however, it differs from *E*. *okamurae* are reddish-colored and lack incurved blades at the upper part of the thallus. | *cox*1: Amansia glomerata ([HM582891](https://www.ncbi.nlm.nih.gov/nuccore/HM582891); 87.8%) | The *cox*1 sequence did not closely match the INSD data and was distant from various other amansieaean genera (S32B Fig. in S5 File). | We could not identify this specimen because of a lack of morphologically similar species, and molecular analyses indicated that it did not belong to any amansieaean genera available in INSD.  Id: U1 |
| *Aneurianna lorentzii* (Weber-van Bosse) L.E.Phillips (S6 Fig. in S4 File) | 25 May 2018; R. Terada & M. Suzuki; TNS AL-209898  *rbc*L: [LC821196](https://www.ncbi.nlm.nih.gov/nuccore/LC821196)  *cox*1: [LC820957](https://www.ncbi.nlm.nih.gov/nuccore/LC820957) | The habit and vegetative structure of this specimen were in accordance with descriptions of *A*. *lorentzii* recorded in Japan and Australia [3,30]. | *rbc*L: Epiglossum smithiae ([MH388544](https://www.ncbi.nlm.nih.gov/nuccore/MH388544); 92.7%)  *cox*1: Neurymenia fraxinifolia ([MH388724](https://www.ncbi.nlm.nih.gov/nuccore/MH388724); 88.2%) | The *rbc*L and *cox*1 sequences did not closely match the INSD data and were distant from various other amansieaean genera (S32 Fig. in S5 File). | Although the *rbc*L and *cox*1 sequences did not closely match the INSD data, we identified this specimen as *A*. *lorentzii* morphologically in accordance with the *lorentzii* recorded in Japan and Australia [3,30].  Id: C2 |
| *Aneurianna* sp. TNE (S6 Fig. in S4 File) | 30 May 2019; R. Terada; TNS AL-220645  *rbc*L: [LC821197](https://www.ncbi.nlm.nih.gov/nuccore/LC821197)  *cox*1: [LC820958](https://www.ncbi.nlm.nih.gov/nuccore/LC820958) | This specimen was morphologically similar to *A*. *lorentzii* [3,30,this study]; however, it is smaller and thiner than *A*. *lorentzii*. | *rbc*L: Epiglossum smithiae ([MH388544](https://www.ncbi.nlm.nih.gov/nuccore/MH388544); 92.5%)  *cox*1: Amansia glomerata ([HM582891](https://www.ncbi.nlm.nih.gov/nuccore/HM582891); 88.7%) | The *rbc*L and *cox*1 analyses suggested that this specimen forms a clade with *A*. *lorentzii* with low to high statistical supports (68–99% BP and 0.98–1.00 PP; S32 Fig. in S5 File). The *rbc*L sequence was 1.5% divergent from *A*. *lorentzii* ([LC821196](https://www.ncbi.nlm.nih.gov/nuccore/LC821196)), whereas the *cox*1 sequence is 7.0% divergent from *A*. *lorentzii* ([LC820957](https://www.ncbi.nlm.nih.gov/nuccore/LC820957)), suggesting that this specimen is distinct from Japanese *A*. *lorentzii*. | We could not identify this specimen. This specimen was morphologically identified as *A*. *lorentzii* offshore Tanegashima Island; however, *rbc*L and *cox*1 analyses revealed that this specimen was distinct from *A*. *lorentzii*. We identified the specimen with large and thick blades as *A*. *lorentzii* and treated this specimen as *A*. sp.  Id: U2 |
| *Chondria intertexta* P.C.Silva (S6 Fig. in S4 File) | 26 May 2015; R. Terada; TNS AL-213816  *rbc*L: [LC821198](https://www.ncbi.nlm.nih.gov/nuccore/LC821198)  *cox*1: [LC820960](https://www.ncbi.nlm.nih.gov/nuccore/LC820960) | The habit and vegetative structure of this specimen were in accordance with descriptions of *C*. *intertexta* described in Japan and South Korea [3,31 as *C*. *intricata*,34]. | *rbc*L: *Chondria intertexta* ([MG255059](https://www.ncbi.nlm.nih.gov/nuccore/MG255059); 99.8%)  *cox*1: *Chondria* sp. ARS 01780 ([HQ423002](https://www.ncbi.nlm.nih.gov/nuccore/HQ423002); 94.2%) | The *rbc*L analyses suggested that these specimens form a clade with *C*. *intertexta* from Hachijo Island, Japan, with full statistical supports (S33A Fig. in S5 File). The *rbc*L sequences were 0.2–0.3% divergent from *C*. *intertexta* collected from Japan ([MG255059](https://www.ncbi.nlm.nih.gov/nuccore/MG255059)). The *cox*1 analyses suggested that *C*. *intertexta* forms a clade with *C*. sp. ARS 01780 from Hawaii, U.S.A. with high statistical supports (95% BP and 1.00 PP; S33B Fig. in S5 File). The *cox*1 sequences were 5.8% divergent from *C*. sp. ARS 01780 ([HQ423002](https://www.ncbi.nlm.nih.gov/nuccore/HQ423002)). | This specimen was identified as *C*. *intertexta*. The identification was supported by both morphological and genetic identification.  Id: C1 |
|  | 18 May 2017; R. Terada; TNS AL-213817  *rbc*L: [LC821198](https://www.ncbi.nlm.nih.gov/nuccore/LC821198)  *cox*1: [LC820961](https://www.ncbi.nlm.nih.gov/nuccore/LC820961) |  |  |  |  |
| *Chondria mageshimensis* Tak.Tanaka & K.Nozawa (S6 Fig. in S4 File) | 18 May 2016; R. Terada; TNS AL-209902  *rbc*L: [LC821200](https://www.ncbi.nlm.nih.gov/nuccore/LC821200)  *cox*1: [LC820962](https://www.ncbi.nlm.nih.gov/nuccore/LC820962) | The habit and vegetative structure of this specimen were in accordance with descriptions of *C*. *mageshimensis* described in Japan [3,18]. | *rbc*L: *Chondria* cf. *mageshimensis* ([MG255060](https://www.ncbi.nlm.nih.gov/nuccore/MG255060); 98.1%)  *cox*1: *Chondria dangeardii* ([GU223879](https://www.ncbi.nlm.nih.gov/nuccore/GU223879); 91.8%) | The *rbc*L analyses suggested that *C*. *mageshimensis* forms a clade with *C*. cf. *mageshimensis* from Hiroshima, Japan ([MG255060](https://www.ncbi.nlm.nih.gov/nuccore/MG255060)), and *C*. sp.1 TNE ([LC821202](https://www.ncbi.nlm.nih.gov/nuccore/LC821202)) collected from offshore Tanegasima Island with full statistical supports (S33A Fig. in S5 File). The *rbc*L sequences were 1.2–1.9% divergent from the other two taxa. The *cox*1 analyses suggested that *C*. *mageshimensis* forms a clade with *C*. sp.1 TNE ([LC820966](https://www.ncbi.nlm.nih.gov/nuccore/LC820966)) with high statistical supports (94%BP and 1.00 PP; S33B Fig. in S5 File). The *cox*1 sequences were 5.3–5.5% divergent from *C*. sp.1 TNE. | Although the *rbc*L sequences of these specimens were 1.9% divergent from that of *C.* *mageshimensis* from Hiroshima, Japan, we identified them as *C*. *mageshimensis* morphologically in accordance with *C*. *mageshimensis* was described in offshore Tanegashima Island. This species was described on offshore Tanegashima Island (as Mageshima Island) [3, 18].  Id: C2 |
|  | 18 May 2017; R. Terada; TNS AL-209400  *cox*1: [LC820963](https://www.ncbi.nlm.nih.gov/nuccore/LC820963) |  |  |  |  |
|  | 20 July 2017; R. Terada; TNS AL-209904  *cox*1: [LC820964](https://www.ncbi.nlm.nih.gov/nuccore/LC820964) |  |  |  |  |
|  | 16 May 2021; R. Terada; TNS AL-220651  *rbc*L: [LC821201](https://www.ncbi.nlm.nih.gov/nuccore/LC821201)  *cox*1: [LC820965](https://www.ncbi.nlm.nih.gov/nuccore/LC820965) |  |  |  |  |
| *Chondria* sp.1 TNE (S6 Fig. in S4 File) | 19 May 2016; R. Terada; TNS AL-209903  *rbc*L: [LC821202](https://www.ncbi.nlm.nih.gov/nuccore/LC821202)  *cox*1: [LC820966](https://www.ncbi.nlm.nih.gov/nuccore/LC820966) | This specimen was morphologically similar to *C*. *mageshimensis* [3,18,this study]; however, it is smaller than *C*. *mageshimensis*. | *rbc*L: *Chondria* cf. *mageshimensis* ([MG255060](https://www.ncbi.nlm.nih.gov/nuccore/MG255060); 98.1%)  *cox*1: *Chondria dangeardii* ([GU223879](https://www.ncbi.nlm.nih.gov/nuccore/GU223879); 92.6%) | The *rbc*L analyses suggested that this specimen forms a clade with *C*. *mageshimensis* ([LC821200](https://www.ncbi.nlm.nih.gov/nuccore/LC821200)) and *C*. cf. *mageshimensis* from Hiroshima, Japan ([MG255060](https://www.ncbi.nlm.nih.gov/nuccore/MG255060)) with full statistical supports (S33A Fig. in S5 File). The *rbc*L sequence was 1.2–1.9% divergent from the other two taxa. The *cox*1 analyses suggested that this specimen forms a clade with *C*. *mageshimensis* ([LC820962](https://www.ncbi.nlm.nih.gov/nuccore/LC820962)) with high statistical supports (94% BP and 1.00 PP; S33B Fig. in S5 File). The *cox*1 sequence was 5.3–5.5% divergent from *C*. *mageshimensis*. | We could not identify this specimen. This specimen was morphologically identified as *C*. *mageshimensis* offshore Tanegashima Island; however, *rbc*L and *cox*1 analyses revealed that this specimen was distinct from *C*. *mageshimensis*. We identified the specimen with large thalli as *C*. *mageshimensis* and treated this specimen as *C*. sp.  Id: U2 |
| *Chondria* sp.2 TNE (S6 Fig. in S4 File) | 21 July 2017; R. Terada; TNS AL-220649  *rbc*L: [LC821203](https://www.ncbi.nlm.nih.gov/nuccore/LC821203)  *cox*1: [LC820967](https://www.ncbi.nlm.nih.gov/nuccore/LC820967) | The cylindrical and entangled thallus of this specimen were similar to *Chondria* species with filamentous thalli, such as *C*. *expansa* recorded in Japan and South Korea [3,34]. | *rbc*L: *Chondria* sp. A11 ([MF094049](https://www.ncbi.nlm.nih.gov/nuccore/MF094049); 94.9%)  *cox*1: *Chondria dasyphylla* ([MH388700](https://www.ncbi.nlm.nih.gov/nuccore/MH388700); 91.1%) | The *rbc*L analyses suggested that this specimen forms a clade with *C*. sp. A11 from Queensland, Australia, with high statistical supports (99% BP and 1.00 PP; S33A Fig. in S5 File). The *rbc*L sequence is 5.1% divergent from *C*. sp. A11 ([MF094049](https://www.ncbi.nlm.nih.gov/nuccore/MF094049)). The *cox*1 sequence did not closely match the INSD data and was distant from various other *Chondria* species (S33B Fig. in S5 File). | We could not identify this specimen. This specimen is morphologically similar to *C*. *expansa* recorded in the Pacific; however, *rbc*L and *cox*1 analyses suggested that it is distant from various *Chondria* species, including *C*. *expansa*.  Id: U2 |
| *Chondria* sp.3 TNE (S6 Fig. in S4 File) | 15 May 2021; R. Terada; TNS AL-222062  *rbc*L: [LC821204](https://www.ncbi.nlm.nih.gov/nuccore/LC821204)  *cox*1: [LC820968](https://www.ncbi.nlm.nih.gov/nuccore/LC820968) | The flattened and lanceolate blades of this specimen were not identical to those of flattened *Chondria* species recorded in the Pacific [3,5,24,34]. | *rbc*L: *Chondria ryukyuensis* ([MG255061](https://www.ncbi.nlm.nih.gov/nuccore/MG255061); 90.9%)  *cox*1: *Chondria dangeardii* ([GU223879](https://www.ncbi.nlm.nih.gov/nuccore/GU223879); 90.0%) | The *rbc*L and *cox*1 sequences did not closely match the INSD data and were distant from various other *Chondria* species (S33 Fig. in S5 File). | We could not identify this specimen because of the lack of similar morphological species and close sequence data in INSD.  Id: U1 |
| *Chondria* sp.4 TNE (S6 Fig. in S4 File) | 18 May 2017; R. Terada; TNS AL-220650  *rbc*L: [LC821205](https://www.ncbi.nlm.nih.gov/nuccore/LC821205)  *cox*1: [LC820969](https://www.ncbi.nlm.nih.gov/nuccore/LC820969) | The cylindrical thalli with acute apices and incurved branches of this specimen were similar to. *C*. *arcuata* recorded in South Korea, Hawaii, and Australia [5,24,34]. | *rbc*L: *Chondria ryukyuensis* ([MG255061](https://www.ncbi.nlm.nih.gov/nuccore/MG255061); 94.7%)  *cox*1: *Chondria decipiens* ([MG272232](https://www.ncbi.nlm.nih.gov/nuccore/MG272232); 89.3%) | The *rbc*L and *cox*1 sequences did not closely match the INSD data and were distant from various other *Chondria* species (S33 Fig. in S5 File). | We could not identify this specimen. This specimen is morphologically similar to *C*. *arcuata* recorded in the Pacific; however, *rbc*L and *cox*1 analyses indicated that it is distant from various *Chondria* species, including *C*. *arcuata*.  Id: U2 |
| *Chondrophycus* sp.1 TNE (S6 Fig. in S4 File) | 18 May 2016; R. Terada; TNS AL-220652  *cox*1: [LC820972](https://www.ncbi.nlm.nih.gov/nuccore/LC820972) | Morphologically, this specimen was similar to *Chondrophycus dotyi* and *C*. *succisus* recorded in Hawaii [5]; however, it differs from these species in that its thallus is flattened, except for the basal part. | *cox*1: *Chondrophycus* sp. ARS 00734 ([HQ423056](https://www.ncbi.nlm.nih.gov/nuccore/HQ423056); 92.4%) | The *cox*1 sequence did not closely match the INSD data and was distant from various *Chondrophycus* species, including *C*. *dotyi* and *C*. *succisus*. (S34B Fig. in S5 File). | We could not identify this specimen because of the lack of similar morphological species recorded in the Pacific and close sequence data in INSD.  Id: U1 |
| *Chondrophycus* sp.2 TNE (S7 Fig in S4 File) | 18 May 2016; R. Terada; TNS AL-220653  *cox*1: [LC820973](https://www.ncbi.nlm.nih.gov/nuccore/LC820973) | This specimen was morphologically similar to *Chondrophycus* sp.1 TNE collected from offshore Tanegashima Island; however, it is smaller than *C*. sp.1 TNE. | *cox*1: *Chondrophycus dotyi* ([HQ423050](https://www.ncbi.nlm.nih.gov/nuccore/HQ423050); 96.1%) | The *cox*1 sequence did not closely match the INSD data and was distant from various other *Chondrophycus* species. (S34B Fig. in S5 File). | We could not identify this specimen. Morphologically, this specimen did not match any *Chondrophycus* species recorded in the Pacific. The *cox*1 analyses indicated that it is distant from various *Chondrophycus* species, including *C*. sp.1 TNE.  Id: U1 |
| *Laurencia* sp. TNE (S7 Fig. in S4 File) | 16 May 2021; R. Terada; TNS AL-220657  *rbc*L: [LC821210](https://www.ncbi.nlm.nih.gov/nuccore/LC821210)  *cox*1: [LC820976](https://www.ncbi.nlm.nih.gov/nuccore/LC820976) | The flattend, cartilaginous, and linear thallil of this specimen were not identical to those of  *Laurencia* species recorded in Japan, South Korea, Hawaii, and Australia [3,5,24,34]. | *rbc*L: *Laurencia flexuosa* ([KX146179](https://www.ncbi.nlm.nih.gov/nuccore/KX146179); 98.1%)  *cox*1: *Laurencia flexuosa* ([KX258821](https://www.ncbi.nlm.nih.gov/nuccore/KX258821); 94.5%) | The *rbc*L analyses suggested that this specimen forms a clade with *L*. *flexuosa* from South Africa with high statistical supports (88% BP and 1.00 PP; S34A Fig. in S5 File). The *cox*1 analyses suggested that this specimen forms a clade with *L*. *flexuosa* from South Africa without statistical supports (less than 50% BP and 0.95 PP; S34B Fig. in S5 File). The *rbc*L sequence was 1.9% divergent from *L*. *flexuosa* ([KX146179](https://www.ncbi.nlm.nih.gov/nuccore/KX146179)), whereas the *cox*1 sequence is 5.5% divergent from *L*. *flexuosa* ([KX258821](https://www.ncbi.nlm.nih.gov/nuccore/KX258821)). | We could not identify this specimen because of the lack of similar morphological species recorded in the Pacific and close sequence data in INSD.  Id: U1 |
| *Lophocladia japonica* Yamada (S7 Fig. in S4 File) | 21 July 2017; R. Terada; TNS AL-220660  *rbc*L: [LC821211](https://www.ncbi.nlm.nih.gov/nuccore/LC821211)  *cox*1: [LC820977](https://www.ncbi.nlm.nih.gov/nuccore/LC820977) | The size, shape of branches of this specimen was in accordance with descriptions of *L*. *japonica* recorded in Japan and South Korea [3,8,35]. | *rbc*L: *Lophocladia kuetzingii* ([OQ970075](https://www.ncbi.nlm.nih.gov/nuccore/OQ970075); 97.7%)  *cox*1: *Lophocladia* sp. ARS 00772 ([HQ422836](https://www.ncbi.nlm.nih.gov/nuccore/HQ422836); 96.0%) | The *rbc*L and *cox*1 sequences did not closely match the INSD data and were distant from various other *Lophocladia* species (S35 Fig. in S5 File). | Although the *rbc*L and *cox*1 sequences did not closely match the INSD data, we identified this specimen as *L*. *japonica* morphologically in accordance with the *L*. *japonica* was described in Japan.  Id: C2 |
| *Neurymenia nigricans* Tak.Tanaka & Itono (S7 Fig. in S4 File) | 16 May 2021; R. Terada; TNS AL-215872  *rbc*L: [LC821213](https://www.ncbi.nlm.nih.gov/nuccore/LC821213)  *cox*1: [LC820979](https://www.ncbi.nlm.nih.gov/nuccore/LC820979) | The shape of blades and inconspicous veins of this specimen was in accordance with descriptions of *N*. *nigricans* recorded in Japan [3,36]. | *rbc*L: Neurymenia sp. UA0004 ([MH388534](https://www.ncbi.nlm.nih.gov/nuccore/MH388534); 95.3%)  *cox*1: Neurymenia fraxinifolia ([MH388724](https://www.ncbi.nlm.nih.gov/nuccore/MH388724); 91.2%) | The *rbc*L and *cox*1 sequences did not closely match the INSD data and were distant from various other *Neurymenia* species (S32 Fig. in S5 File). | Although the *rbc*L and *cox*1 sequences did not closely match the INSD data, we identified this specimen as *N*. *nigricans* morphologically in accordance with the *N*. *nigricans* was described in Japan.  Id: C2 |
| *Tolypiocladia* sp. TNE (S22 Fig. in S4 File) | 28 September 2017; R. Terada; TNS AL-213823  *rbc*L: [LC821218](https://www.ncbi.nlm.nih.gov/nuccore/LC821218) | The shap of branches and branching pattern of this specimen were similar to those of *Tolypiocladia* *glomerulata* recorded in Japan, Hawaii, and Australia [3,5,7]. | *rbc*L: *Tolypiocladia* *glomerulata* ([MF101467](https://www.ncbi.nlm.nih.gov/nuccore/MF101467); 92.6%) | The *rbc*L sequence was 6.7–7.4% divergent from *T*. *glomerulata* from Taiwan and Western Australia ([MN920294](https://www.ncbi.nlm.nih.gov/nuccore/MN920294), [MN920305](https://www.ncbi.nlm.nih.gov/nuccore/MN920305), [MN920332](https://www.ncbi.nlm.nih.gov/nuccore/MN920305), [MF101467](https://www.ncbi.nlm.nih.gov/nuccore/MF101467); S36 Fig. in S5 File). | We could not identify this specimen. Morphologically, this specimen was identified as *T*. *glomerulata*. However, the *rbc*L analyses revealed that the Japanese specimen was distinct from the Australian *T*. *glomerulata*, suggesting *T*. *glomerulata* recorded in Japan appear to be different species. Further morpho-anatomical and molecular analyses, including additional specimens from Japan, Australia, and the other parts of the Pacific, are needed to clarify its identification.  Id: U2 |
| *Wrightiella* sp. TNE (S7 Fig. in S4 File) | 18 May 2016; R. Terada; TNS AL-220662  *rbc*L: [LC821219](https://www.ncbi.nlm.nih.gov/nuccore/LC821219)  *cox*1: [LC820983](https://www.ncbi.nlm.nih.gov/nuccore/LC820983) | Morphologically, this specimen did not match any rhodomelacean species recorded in the Pacific [3,5,24,34,37]. The habit of this specimen is similar to that of *Wrightiella tumanowiczi* recorded in the Caribbean [38,39]; however, it much smaller than *W*. *tumanowiczii*. | *rbc*L: *Wrightiella tumanowiczii* ([EU492922](https://www.ncbi.nlm.nih.gov/nuccore/EU492922); 97.0%)  *cox*1: *Laurencia snackeyi* ([MF093985](https://www.ncbi.nlm.nih.gov/nuccore/MF093985); 86.7%) | The *rbc*L analyses suggested that this specimen forms a clade with *W*. *tumanowiczii* with full statistical supports (S35A Fig. in S5 File). The *rbc*L sequence was 3.0% divergent from *W*. *tumanowiczii* ([EU492922](https://www.ncbi.nlm.nih.gov/nuccore/EU492922)). The *cox*1 analyses suggested that this specimen forms a clade with *W*. *blodgettii* with high statistical supports (99% BP and 0.99 PP; S35B Fig. in S5 File). The *cox*1 sequence was 10.5% divergent from *W*. *blogettii* ([ALSEU247-16](https://www.boldsystems.org/index.php/Public_RecordView?processid=ALSEU247-16)). | We could not identify this specimen. Morphologically, this specimen did not match any rhodomelacean species recorded in the Pacific. Both the morphological characteristics and molecular analyses indicated that this specimen is related to *Wrightiella*; however, *rbc*L and *cox*1 sequences are distant from *W*. *tumanowiczii* and *W*. *blodgettii*.  Id: U1 |
| Rhodymeniophycidae, Ceramiales, Wrangeliaceae | | | | | |
| *Anotrichium* sp. TNE (S7 Fig. in S4 File) | 3 October 2021; R. Terada; TNS AL-222107  *rbc*L: [LC821223](https://www.ncbi.nlm.nih.gov/nuccore/LC821223)  *cox*1: [LC820987](https://www.ncbi.nlm.nih.gov/nuccore/LC820987) | The vegetative structures of this specimen were in accordance with those of *Anotrichium* species with tufted thalli, such as *A*. *furcellatum*, *A*. *subtile*, *A*. *tenue*, and *A*. *yagii* recorded in the Pacific [3,5,7,14,19]; however, it lacks tetrasporangia, which is the key morphological characteristic of *Anotrichium* species. | *rbc*L: *Anotrichium yagii* ([LC821220](https://www.ncbi.nlm.nih.gov/nuccore/LC821220); 98.2%)  *cox*1: *Anotrichium* sp. ARS 01533 ([HQ423101](https://www.ncbi.nlm.nih.gov/nuccore/HQ423101)as Ceramiales sp.; 97.2%) | The *rbc*L analyses suggested that this specimen forms a clade with *A*. *yagii* with moderate to high statistical supports (82% BP, 1.00 PP; S37A Fig. in S5 File). The *rbc*L sequence was 1.8–2.1% divergent from *A*. *yagii* ([LC821220](https://www.ncbi.nlm.nih.gov/nuccore/LC821220), [LC821221](https://www.ncbi.nlm.nih.gov/nuccore/LC821221)). The *cox*1 analyses suggested that this specimen forms a clade with *A*. *yagii* and *A*. sp. ARS 01533 from Hawaii, U.S.A. with high statistical supports (94% BP, 1.00 PP; S37B Fig. in S5) File). The *cox*1 sequence was 2.8–6.2% divergent from the other two taxa ([HQ423101](https://www.ncbi.nlm.nih.gov/nuccore/HQ423101), [LC820984](https://www.ncbi.nlm.nih.gov/nuccore/LC820984), [LC820985](https://www.ncbi.nlm.nih.gov/nuccore/LC820985)). | We could not identify this specimen because of the lack of tetrasporangia, and the sequences closely matched the INSD data. The *rbc*L and *cox*1 analyses indicated that the specimen was distant from *A*. *furcellatum*, *A*. *tenue*, and *A*. *yagii*. Further morpho-anatomical and molecular analyses, including additional specimens produced tetrasporangia, are needed to clarify its identification.  Id: U3 |
| *Griffithsia venusta* Yamada (S7 Fig. in S4 File) | 26 May 2015; R. Terada; TNS AL-200149  *rbc*L: [LC821226](https://www.ncbi.nlm.nih.gov/nuccore/LC821226)  *cox*1: [LC820990](https://www.ncbi.nlm.nih.gov/nuccore/LC820990) | The subdichotomously branched thallus composed of spherical cells was observed in this specimen, in accordance with the description of *G*. *venusta* were recorded in Japan and South Korea [3,8,19,40]. | *rbc*L: *Griffithsia tomo-yamadae* ([AY295171](https://www.ncbi.nlm.nih.gov/nuccore/AY295171); 99.8%)  *cox*1: *Griffithsia* sp. ARS 01645 ([HQ423085](https://www.ncbi.nlm.nih.gov/nuccore/HQ423085); 92.5%) | The *rbc*L analyses suggested that this specimen forms a clade with *G*. *tomo-yamadae* Okamura from South Korea with full statistical supports (S37A Fig. in S5 File). The *rbc*L sequence was 0.2% divergent from *G*. *tomo-yamadae* ([AY295171](https://www.ncbi.nlm.nih.gov/nuccore/AY295171)). The *cox*1 analyses suggested that this specimen forms a clade with *G*. sp. ARS 01645 from Hawaii, U.S.A. with full statistical supports (S37B Fig. in S5 File). The *cox*1 sequence was 7.5% divergent from *G*. sp. ARS 01645 ([HQ423085](https://www.ncbi.nlm.nih.gov/nuccore/HQ423085)). | We identified this specimen as *G*. *venusta* morphologically in accordance with the *G*. *venusta* has been recorded in Japan and South Korea. The *rbc*L analyses indicated that it is conspecific with Korean *G*. *tomo-yamadae*. However, *G*. *venusta* is morphologically different from *G*. *tomo-yamadae*. *Griffithsia venusta* is less than 2.0 cm high and is composed of spherical cells, whereas *G*. *tomo-yamadae* is more than 5.0 cm high and composed of cylindrical cells [3,40]. Further morpho-anatomical analyses, including additional specimens and type specimens of *G*. *venusta* and *G*. *tomo-yamadae* or samples from their type localities (Kanagawa, Japan; Mie, Japan) are needed to clarify their identification.  Id: C2 |
| “*Griffithsia*” sp.1 TNE (*G*. cf. *subcylindrica*; S7 Fig. in S4 File) | 3 October 2021; R. Terada; TNS AL-222106  *rbc*L: [LC821227](https://www.ncbi.nlm.nih.gov/nuccore/LC821227)  *cox*1: [LC820991](https://www.ncbi.nlm.nih.gov/nuccore/LC820991) | The tufted and filamentous thalli of these specimens were similar to those of *G*. *subcylindrica* has been recorded in Japan and Hawaii [3,5,15,41]. This specimen is also similar to *G*. *coacta* was recorded in Japan [3,41]; however, it lacks tetrasporangia, which is the key morphological characteristic of *G*. *coacta* and *G*. *subcylindrica*. Although these specimens lacked tetrasporangia, the size of thalli (3.0 to 9.0 cm high) were similar to those of *G.subcylindrica* rather than *G*. *coacta*. *Griffithsia coacta* is 2.0 to 3.0 cm high, whereas *G*. *subcylindrica* is more than 5.0 cm high [3,41]. | *rbc*L: *"Griffithsia"* sp.1 JFC-2019 ([MK125386](https://www.ncbi.nlm.nih.gov/nuccore/MK125386); 92.2%)  *cox*1: *Griffithsia subcylindrica* ([HQ422664](https://www.ncbi.nlm.nih.gov/nuccore/HQ422664); 93.4%) | The *rbc*L and *cox*1 sequences did not closely match the INSD data and were distant from various *Griffithsia* species. The *rbc*L analyses suggested that this specimen is not included in *Griffithsia* clade (S37A Fig. in S5 File). The *cox*1 analyses suggested that this specimen forms a clade with *G*. *subcylindrica* from Hawaii, U.S.A. and *G*. sp. ODC1630 from Kenya with full statistical supports (S36B Fig. in S5 File). The *cox*1 sequence was 4.8–6.6% divergent from the other two taxa ([HQ422664](https://www.ncbi.nlm.nih.gov/nuccore/HQ422664), [HQ956966](https://www.ncbi.nlm.nih.gov/nuccore/HQ956966.1?report=genbank)). | We tentatively identified these specimens as *G*. cf. *subcylindrica*. Morphologically, these specimens were similar to *G*. *subcylindrica*. However, we must observe tetrasporangial fascicles to distinguish them from *G*. *coacta* clearly. The *cox*1 analyses indicated that Hawaiian *G*. *subcylindrica* is distinct from our specimens. Further morpho-anatomical and molecular analyses, including additional specimens that produced tetrasporangia and type specimens of *G*. *subcylindrica* and *G*. *coacta* or samples from their type localities (Hachijo Island, Japan) are needed to clarify their identification.  Id: T |
|  | 5 June 2022; R. Terada; TNS AL-222167  *rbc*L: [LC821228](https://www.ncbi.nlm.nih.gov/nuccore/LC821228) |  |  |  |  |
| “*Griffithsia*” sp.2 TNE (S7 Fig. in S4 File) | 3 October 2021; R. Terada; TNS AL-222108  *rbc*L: [LC821229](https://www.ncbi.nlm.nih.gov/nuccore/LC821229)  *cox*1: [LC820992](https://www.ncbi.nlm.nih.gov/nuccore/LC820992) | The dichotomously branched thallus composed of cylindrical cells was observed in this specimen, in accordance with the description of *G*. *japonica* and *G*. *okiensis* were recorded in Japan and South Korea [3,8,15,19,42-44]. | *rbc*L: *Griffithsia okiensis* ([AY604865](https://www.ncbi.nlm.nih.gov/nuccore/AY604865); 97.1%)  *cox*1: *Griffithsia okiensis* ([EU194973](https://www.ncbi.nlm.nih.gov/nuccore/EU194973); 95.0%) | The *rbc*L analyses suggested that this specimen forms a clade with *G*. *japonica* and *G*. *okiensis* with moderate to high statistical supports (77% BP and 1.00 PP; S37A Fig. in S5 File). The *rbc*L sequence was 2.9–3.3% divergent from the other two taxa ([AY295165](https://www.ncbi.nlm.nih.gov/nuccore/34100811), [AY604865](https://www.ncbi.nlm.nih.gov/nuccore/AY604865), [LC821224](https://www.ncbi.nlm.nih.gov/nuccore/LC821224), [LC821225](https://www.ncbi.nlm.nih.gov/nuccore/LC821225)). The *cox*1 analyses suggested that this specimen forms a clade with *G*. *okiensis* with low statistical supports (63% BP and less than 0.95 PP; S37B Fig. in S5 File). The *cox*1 sequence was 5.0–5.6% divergent from *G*. *okiensis* ([EU194973](https://www.ncbi.nlm.nih.gov/nuccore/EU194973), [LC820988](https://www.ncbi.nlm.nih.gov/nuccore/LC820988), [LC820989](https://www.ncbi.nlm.nih.gov/nuccore/LC820989)). | We could not identify this specimen. This specimen was morphologically similar to *G*. *japonica* and *G*. *okiensis*; however, *rbc*L and *cox*1 analyses revealed that this specimen was distinct from *G*. *japonica* and *G*. *okiensis*.  Id: U2 |
| *Pleonosporium* sp. TNE (S7 Fig. in S4 File) | 25 May 2018; R. Terada & M. Suzuki; TNS AL-220689  *rbc*L: [LC821231](https://www.ncbi.nlm.nih.gov/nuccore/LC821231)  *cox*1: [LC820993](https://www.ncbi.nlm.nih.gov/nuccore/LC820993) | The vegetative structures of this specimen were in accordance with those of *Pleonosporium* species recorded in the northern Pacific [3,5,15,19]; however, it lacks tetrasporangia or polysporangia, which are the key morphological characteristics of *Pleonosporium* species. | *rbc*L: *Pleonosporium ricksearlesii* ([OR336112](https://www.ncbi.nlm.nih.gov/nuccore/OR336112); 94.7%)  *cox*1: *Pleonosporium australicum* ([OR336105](https://www.ncbi.nlm.nih.gov/nuccore/OR336105); 87.7%) | The *rbc*L analyses suggested that this specimen forms a clade with *P*. *borreri* with moderate to high statistical supports (81% BP and 1.00 PP; S37A Fig. in S5 File). The *rbc*L sequence was 5.6% divergent from *P*. *borreri* ([MK814701](https://www.ncbi.nlm.nih.gov/nuccore/1631824018)). The *cox*1 sequence did not closely match the INSD data and was distant from various other *Pleonosporium* species. (S37B Fig. in S5 File). | We could not identify this specimen because of the lack of tetrasporangia or polysporaniga, and the sequences closely matched the INSD data. Further morpho-anatomical and molecular analyses, including additional specimens produced tetrasporangia or polysporangia, are needed to clarify its identification.  Id: U3 |
| *Wrangelia tagoi* (Okamura) Okamura & Segawa (S8 Fig. in S4 File) | 28 September 2017; R. Terada; TNS AL-209907  *rbc*L: [LC821233](https://www.ncbi.nlm.nih.gov/nuccore/LC821233)  *cox*1: [LC820995](https://www.ncbi.nlm.nih.gov/nuccore/LC820995) | The cylindrical to slightly flattened, and pinnately branched thalli of these specimens were in accordance with descriptions of *W*. *tagoi* recorded in Japan [3,15,45]. | *rbc*L: *Wrangelia abscondita* ([OQ561907](https://www.ncbi.nlm.nih.gov/nuccore/OQ561907); 93.4%)  *cox*1: *Wrangelia elegantissima* ([OM460678](https://www.ncbi.nlm.nih.gov/nuccore/OM460678); 86.6%) | The *rbc*L and *cox*1 sequences did not closely match the INSD data and were distant from various other *Wrangelia* species (S37 Fig. in S5 File). | Although the *rbc*L and *cox*1 sequences did not closely match the INSD data, we identified this specimen as *W*. *tagoi* morphologically in accordance with the *W*. *tagoi* was described in Japan.  Id: C2 |
|  | 29 September 2022; R. Terada; TNS AL-222220  *rbc*L: [LC821234](https://www.ncbi.nlm.nih.gov/nuccore/LC821234)  *cox*1: [LC820996](https://www.ncbi.nlm.nih.gov/nuccore/LC820996) |  |  |  |  |
| *Wrangelia* sp. TNE (*W*. cf. *tanegana*; S8 Fig. in S4 File) | 15 May 2022; R. Terada; TNS AL-222176  *rbc*L: [LC821236](https://www.ncbi.nlm.nih.gov/nuccore/LC821236)  *cox*1: [LC820997](https://www.ncbi.nlm.nih.gov/nuccore/LC820997) | The filamentous thalli producing four whorled laterals from main axis of this specimen were in accordance with those of *W*. *tanegana* recorded in Japan and South Korea [3,8,15 as *W*. *tayloriana*,16]. | *rbc*L: *Wrangelia argus* ([OQ561903](https://www.ncbi.nlm.nih.gov/nuccore/OQ561903); 97.5%)  *cox*1: *Wrangelia* sp. US:BLB 1688 ([OM460693](https://www.ncbi.nlm.nih.gov/nuccore/OM460693); 92.9%) | The *rbc*L analyses suggested that this specimen forms a clade with *W*. *argus* from Bermuda, *W*. *plumosa* and *W*. spp. from Australia, and *W*. sp.1 JP (*W*. cf. *tanegana*) from Japan with high statistical supports (99% BP and 1.00 PP; S37A Fig. in S5 File). The *rbc*L sequence is 2.5–3.9% divergent from the other seven taxa ([KY682905](https://www.ncbi.nlm.nih.gov/nuccore/KY682905), [LC821235](https://www.ncbi.nlm.nih.gov/nuccore/LC821235), [MK814740](https://www.ncbi.nlm.nih.gov/nuccore/MK814740), [OQ561898](https://www.ncbi.nlm.nih.gov/nuccore/OQ561898), [OQ561903](https://www.ncbi.nlm.nih.gov/nuccore/OQ561903), [OQ561904](https://www.ncbi.nlm.nih.gov/nuccore/OQ561904), [OQ561911](https://www.ncbi.nlm.nih.gov/nuccore/OQ561911)). The *cox*1 sequence did not closely match the INSD data and was distant from various other *Wrangelia* species (S37B Fig. in S5 File). | We tentatively identified this specimen as *W*. cf. *tanegana*. Morphologically, the specimen was identified as *W*. *tanegana*. However, *rbc*L analyses revealed that *W*. *tanegana* recorded in Japan comprises two cryptic species. Tanegashima Island is a type locality of *W*. *tanegana*; however, the lectotype specimen of *W*. *tanegana* is tufted and more than 2 cm high [46], whereas our specimen is epiphytic on *Laurencia* sp. and is less than 1 cm high. Therefore, additional specimens, including collections from shallow subtidal on Tanegashima Island are need to confirm the identificaiton of our specimen.  Id: T |
| Rhodymeniophycidae, Gigartinales, Cystocloniaceae | | | | | |
| *Calliblepharis saidana* (Holmes) M.Y.Yang & M.S.Kim (S8 Fig. in S4 File) | 26 June 2021; R. Terada; TNS AL-220759  *rbc*L: [LC821238](https://www.ncbi.nlm.nih.gov/nuccore/LC821238)  *cox*1: [LC820999](https://www.ncbi.nlm.nih.gov/nuccore/LC820999) | The entangled, flattened, and curved blades of this specimen were in accordance with those of *C*. *saidana* recorded in Japan and South kore [3,8,47-50]. | *rbc*L: *Calliblepharis saidana* ([LC821237](https://www.ncbi.nlm.nih.gov/nuccore/LC821237); 99.6%)  *cox*1: *Calliblepharis saidana* ([LC820998](https://www.ncbi.nlm.nih.gov/nuccore/LC820998); 98.3%) | The *rbc*L analyses suggested that this specimen forms a clade with *C*. *saidana* from Japan, South Korea, North Carolina, U.S.A., and Brazil, with full statistical supports (S38A Fig. in S5 File). The *rbc*L sequence was 0.4–0.9% divergent from the other three taxa ([MK614693](https://www.ncbi.nlm.nih.gov/nuccore/MK614693), [MZ303644](https://www.ncbi.nlm.nih.gov/nuccore/MZ303644), [LC821237](https://www.ncbi.nlm.nih.gov/nuccore/LC821237)). The combined *rbc*L and *cox*1 analyses suggested that this specimen forms a clade with *C*. *saidana* from Japan and North Carolina, U.S.A. with full statistical supports (S38C Fig. in S5 File). The *cox*1 sequence was 1.7–3.0% divergent from the other two taxa ([MZ303668](https://www.ncbi.nlm.nih.gov/nuccore/MZ303668), [LC820998](https://www.ncbi.nlm.nih.gov/nuccore/LC820998)). | This specimen was identified as *C*. *saidana*. The identification was supported by both morphological and genetic identification.  Id: C1 |
| *Calliblepharis yasutakei* Paiano & A.R.Sherwood (S9 Fig in S4 File and S6 File) | 25 May 2018; R. Terada & M. Suzuki; TNS AL-220756  *rbc*L: [LC821239](https://www.ncbi.nlm.nih.gov/nuccore/LC821239)  *cox*1: [LC821000](https://www.ncbi.nlm.nih.gov/nuccore/LC821000) | This species is a new record for Japan. The details of the morpho-anatomical observations and identification of this specimen is presented in S6 File. | *rbc*L: *Calliblepharis yasutakei* ([OL795916](https://www.ncbi.nlm.nih.gov/nuccore/OL795916); 99.6%)  *cox*1: *Calliblepharis yasutakei* ([OL795915](https://www.ncbi.nlm.nih.gov/nuccore/OL795915); 99.8%) | The combined *rbc*L and *cox*1 analyses suggested that this specimen forms a clade with *C*. *yasutakei* from Hawaii, U.S.A. with high statistical supports (94% BP and 1.00 PP; S38C Fig. in S5 File). The *rbc*L sequences were 0.4% divergent from the holotype of *C*. *yasutakei* from Hawaii, U.S.A. ([OL795916](https://www.ncbi.nlm.nih.gov/nuccore/OL795916)). The *cox*1 analyses suggested that these specimens form a clade with *C*. *yasutakei* from Hawaii, U.S.A. and South Africa (as Ceramiales sp.) with full statistical supports (S38B Fig. in S5 File). The *cox*1 sequences were 0.2–1.4% divergent from the other two taxa ([OL795915](https://www.ncbi.nlm.nih.gov/nuccore/OL795915), [HQ956841](https://www.ncbi.nlm.nih.gov/nuccore/HQ956841.1?report=genbank)). | This specimen was identified as *C*. *yasutakei*. The identification was supported by both morphological and genetic identification.  Id: C1 |
|  | 16 May 2021; R. Terada; TNS AL-220757  *rbc*L: [LC821240](https://www.ncbi.nlm.nih.gov/nuccore/LC821240)  *cox*1: [LC821001](https://www.ncbi.nlm.nih.gov/nuccore/LC821001) |  |  |  |  |
| *Calliblepharis* sp. 1 TNE (S8 Fig. in S4 File) | 18 May 2016; R. Terada; TNS AL-220755  *rbc*L: [LC821241](https://www.ncbi.nlm.nih.gov/nuccore/LC821241)  *cox*1: [LC821002](https://www.ncbi.nlm.nih.gov/nuccore/LC821002) | Morphologically, this specimen was similar to *C*. *saidana* and *C*. *yasutakei* recorded in Japan, South Korea, and Hawaii [3,8,47-51,this study]; however, it differs from these species in that its bload axis and ligulate-shape branches. | *rbc*L: *Calliblepharis fimbriata* ([AF385654](https://www.ncbi.nlm.nih.gov/nuccore/AF385654); 97.3%)  *cox*1: *Calliblepharis celatospora* ([MN941987](https://www.ncbi.nlm.nih.gov/nuccore/MN941987); 92.4%) | The *rbc*L and *cox*1 sequences did not closely match the INSD data and were distant from various other *Calliblepharis* species (S38 Fig. in S5 File). | We could not identify this specimen because of the lack of similar morphological species recorded in the Pacific and close sequence data in INSD.  Id: U1 |
| *Calliblepharis* sp. 2 TNE (S8 Fig. in S4 File) | 20 July 2017; R. Terada; TNS AL-220758  *rbc*L: [LC821242](https://www.ncbi.nlm.nih.gov/nuccore/LC821242)  *cox*1: [LC821003](https://www.ncbi.nlm.nih.gov/nuccore/LC821003) | Morphologically, this specimen was most similar to *C*. *yasutakei* recorded in Japan and Hawaii [51,this study]; however, it differs from *C*. *yasutakei* because its branches are not curved or slightly curved. | *rbc*L: *Calliblepharis celatospora* ([MN942000](https://www.ncbi.nlm.nih.gov/nuccore/MN942000); 97.0%)  *cox*1: *Calliblepharis celatospora* ([MN941987](https://www.ncbi.nlm.nih.gov/nuccore/MN941987); 93.3%) | The *rbc*L and *cox*1 sequences did not closely match the INSD data and were distant from various other *Calliblepharis* species (S38 Fig. in S5 File). | We could not identify this specimen because of the lack of similar morphological species recorded in the Pacific and close sequence data in INSD.  Id: U1 |
| *Hypnea yamadae* Tak.Tanaka (S8 Fig. in S4 File) | 20 July 2017; R. Terada; TNS AL-209788  *rbc*L: [LC821247](https://www.ncbi.nlm.nih.gov/nuccore/LC821247)  *cox*1: [LC821008](https://www.ncbi.nlm.nih.gov/nuccore/LC821008) | The bright red, cylindrical to slightly compressed thalli of these specimens were in iaccordance with descriptions of *H*. *yamadae* recorded in Japan [3,52]. | *rbc*L: *Hypnea yamadae* ([AB095916](https://www.ncbi.nlm.nih.gov/nuccore/AB095916); 99.9%)  *cox*1: *Hypnea* sp. ([MT943664](https://www.ncbi.nlm.nih.gov/nuccore/MT943664) as *H*. *musciformis*; 98.0%) | The *rbc*L analyses suggested that these specimens form a clade with *H*. *yamadae* from Nagasaki, Japan with full statistical supports (S38A Fig. in S5 File). The *rbc*L sequences were 0.1% divergent from Japanese collection assigned to this species ([AB095916](https://www.ncbi.nlm.nih.gov/nuccore/AB095916)). The combined *rbc*L and *cox*1 analyses suggested that *H*. *yamadae* forms a clade, with *H*. *rosea* Papenfuss from South Africa and *H*. sp. from South Australia with high statistical supports (96% BP and 1.00 PP; S38C Fig. in S5 File). The *rbc*L sequences were 1.9–2.0% divergent from the other two taxa ([FJ694935](https://www.ncbi.nlm.nih.gov/nuccore/FJ694935), [MT943664](https://www.ncbi.nlm.nih.gov/nuccore/MT943664)), whereas the *cox*1 sequences were 6.8–7.0% divergent from the other two taxa ([GQ141883](https://www.ncbi.nlm.nih.gov/nuccore/GQ141883), [MT943622](https://www.ncbi.nlm.nih.gov/nuccore/MT943622)). | This specimen was identified as *H*. *yamadae*. The identification was supported by both morphological and genetic identification.  Id: C1 |
|  | 16 May 2021; R. Terada; TNS AL-215742  *rbc*L: [LC821248](https://www.ncbi.nlm.nih.gov/nuccore/LC821248)  *cox*1: [LC821009](https://www.ncbi.nlm.nih.gov/nuccore/LC821009) |  |  |  |  |
| Rhodymeniophycidae, Gigartinales, Furcellariaceae | | | | | |
| *Halarachnion latissimum* Okamura (S8 Fig. in S4 File) | 30 May 2019; R. Terada; TNS AL-220760  *rbc*L: [LC821251](https://www.ncbi.nlm.nih.gov/nuccore/LC821251)  *cox*1: [LC821012](https://www.ncbi.nlm.nih.gov/nuccore/LC821012) | The membranous blade and vegetative anatomy of this specimen were in iaccordance with descriptions of *H*. *latissimum* recorded in Japan [3,53]. | *rbc*L: *Halarachnion latissimum* ([LC821250](https://www.ncbi.nlm.nih.gov/nuccore/LC821250); 99.2%)  *cox*1: *Halarachnion latissimum* ([LC821011](https://www.ncbi.nlm.nih.gov/nuccore/LC821011); 98.6%) | The *rbc*L analyses suggested that this specimen forms a clade with *H*. *latissimum* from Chiba and Awaji Island, Japan, with full statistical supports (S39A Fig. in S5 File). The *rbc*L sequence was 0.8–1.1% divergent from Japanese collections assigned to this species ([KF709207](https://www.ncbi.nlm.nih.gov/nuccore/KF709207), ([LC821250](https://www.ncbi.nlm.nih.gov/nuccore/LC821250)). The *cox*1 analyses suggested that this specimen forms a clade with *H*. *latissimum* from Awaji Island, Japan, with high statistical supports (95% BP and 1.00 PP; S39B Fig. in S5 File). The *cox*1 sequence was 1.4% divergent from Japanese collection assigned to this species ([LC821011](https://www.ncbi.nlm.nih.gov/nuccore/LC821011)). | This specimen was identified as *H*. *latissimum*. The identification was supported by both morphological and genetic identification.  Id: C1 |
| Rhodymeniophycidae, Gigartinales, Gigartinaceae | | | | | |
| *Chondracanthus saundersii* C.W.Schneider & C.E.Lane (S8 Fig. in S4 File) | 20 July 2017; R. Terada; TNS AL-209799  *rbc*L: [LC538291](https://www.ncbi.nlm.nih.gov/nuccore/LC538291)  *cox*1: [LC538298](https://www.ncbi.nlm.nih.gov/nuccore/LC538298) |  |  |  | This specimen was recorded from offshore of Tanegashima Island (as Mageshima Island) as a new record for Japan [54].  Id: C1 |
| Rhodymeniophycidae, Gigartinales, Kallymeniaceae | | | | | |
| *Austrokallymenia* sp.1 (*Kallymenia* cf. *sessilis*; S8 Fig. in S4 File) | 19 May 2016; R. Terada; TNS AL-209810  *rbc*L: [LC821253](https://www.ncbi.nlm.nih.gov/nuccore/LC821253)  *cox*1: [LC821014](https://www.ncbi.nlm.nih.gov/nuccore/LC821014) | The membranous blade and vegetative anatomy of this specimen were in iaccordance with descriptions of *Kallymenia sessilis* recorded in Japan and South Korea [3,8,55]. | *rbc*L: *Austrokallymenia roensis* ([KX783118](https://www.ncbi.nlm.nih.gov/nuccore/KX783118); 98.2%)  *cox*1: *Austrokallymenia roensis* ([KX808094](https://www.ncbi.nlm.nih.gov/nuccore/KX808094); 96.8%) | The combined *rbc*L and *cox*1 analyses suggested that specimens assigned to *K*. *sessilis* form a clade with high statistical supports (97% BP and 1.00 PP) and are included in the *Austrokallymenia* clade (S40 Fig. in S5 File). The specimens assigned to *K*. *sessilis* were divided into two lineages: *A*. sp.1 from Ehime and offshore Tanegashima Island, Japan, and *A*. sp.2 from Takeno, Japan (Fig. S40 in S5 File). The *rbc*L sequences collected from offshore of Tanegashima Island were 0.2–0.3% divergent from *A*. sp.1 from Ehime, Japan, whereas the *cox*1 sequences were 0.9–1.4% divergent from *A*. sp.1 from Ehime, Japan. The *rbc*L sequences of *A*. sp.1 were 0.9–1.1% divergent from *A*. sp.2, whereas the *cox*1 sequences were 2.4–2.9% divergent from *A*. sp.2. | We tentatively identified this specimen as *K*. cf. *sessilis*. Morphologically, the specimen was identified as *K*. *sessilis*. However, *rbc*L and *cox*1 analyses revealed that *K*. *sessilis* recorded in Japan includes two cryptic species. In addition, the specimens were assigned to *K*. *sessilis* were inlcuded in the *Austrokallymenia* clade. We could not determine which corresponds to true *K*. *sessilis*, and treated them as *A*. sp.1 (*K*. cf. *sessilis*) and *A*. sp.2 (*K*. cf. *sessilis*). Further morpho-anatomical and molecular analyses, including the type specimens of *K*. *sessilis* or samples from a type locality (Chiba, Japan) are required to clarify its identification and genetic status.  Id: T |
|  | 30 May 2019; R. Terada; TNS AL-213790  *rbc*L: [LC821254](https://www.ncbi.nlm.nih.gov/nuccore/LC821254)  *cox*1: [LC821015](https://www.ncbi.nlm.nih.gov/nuccore/LC821015) |  |  |  |  |
| *Callophyllis* sp.1 TNE (*C*. cf. *adhaerens*; S8 Fig. in S4 File) | 25 May 2018; R. Terada & M. Suzuki; TNS AL-209807  *rbc*L: [LC821257](https://www.ncbi.nlm.nih.gov/nuccore/LC821257)  *cox*1: [LC821018](https://www.ncbi.nlm.nih.gov/nuccore/LC821018) | The linear blades with small protuberances on the margins were in accordance with those of *C*. *adhaerens* recorded in Japan and South Korea [3,8,56]; however, it differs from *C*. *adhaerens* because of its reddish color and wider blades. | *rbc*L: *Callophyllis adhaerens* ([KM675239](https://www.ncbi.nlm.nih.gov/nuccore/KM675239); 100%)  *cox*1: *Callophyllis adhaerens* ([KM675337](https://www.ncbi.nlm.nih.gov/nuccore/KM675337); 99.7%) | The *rbc*L analyses suggested that this specimen forms a clade with *C*. *adhaerens* from South Korea with high statistical supports (98% BP and 1.00 PP; S41A Fig. in S5 File). The *rbc*L sequence was 0–1.0% divergent from *C*. *adhaerens* from South Korea ([KM675239](https://www.ncbi.nlm.nih.gov/nuccore/KM675239), [KX783085](https://www.ncbi.nlm.nih.gov/nuccore/KX783085)). The *cox*1 analyses suggested that this specimen forms a clade with *C*. *adhaerens* from South Korea with high statistical supports (87% BP and 1.00 PP; S41B Fig. in S5 File). The *cox*1 sequence is 0.3% divergent from *C*. *adhaerens* from South Korea ([KM675337](https://www.ncbi.nlm.nih.gov/nuccore/KM675337)) and 2.8% divergent from *C*. *adhaerens* from South Korea ([KX808040](https://www.ncbi.nlm.nih.gov/nuccore/KX808040), [KX808057](https://www.ncbi.nlm.nih.gov/nuccore/KX808057)). | We tentatively identified this specimen as *C*. cf. *adhaerens*. Morphologically, the specimen was similar to *C*. *adhaerence*. However, *cox*1 analyses revealed that *C*. *adhaerence* recorded in Japan and South Korea includes two to three cryptic species. Further morphological and molecular analyses, including the type specimen of *C*. *adhaerence* or samples from a type locality (Kanagawa, Japan) are needed to clarify its identification.  Id: T |
| *Callophyllis* sp.2 TNE (S10 Fig. in S4 File) | 30 May 2019; R. Terada; TNS AL-222049  *rbc*L: [LC821258](https://www.ncbi.nlm.nih.gov/nuccore/LC821258)  *cox*1: [LC821019](https://www.ncbi.nlm.nih.gov/nuccore/LC821019) | The creeping thallus and palmate blades of this specimen were similar to those of *C*. *adnata*, *C*. *mollitia*, and *C*. *repens* recorded in Japan and South Korea [3,8,57,58]. | *rbc*L: *Callophyllis nana* ([KX783087](https://www.ncbi.nlm.nih.gov/nuccore/KX783087); 97.1%)  *cox*1: *Callophyllis nana* ([KX808059](https://www.ncbi.nlm.nih.gov/nuccore/1199744613); 96.2%) | The *rbc*L and *cox*1 analyses suggested that this specimen forms a clade with *C*. *nana* from Norfolk Island, Australia, with high to full statistical supports (97–100% BP and 1.00 PP; S41 Fig. in S5 File). The *rbc*L sequence was 2.9% divergent from *C*. *nana* ([KX783087](https://www.ncbi.nlm.nih.gov/nuccore/KX783087)), whereas the *cox*1 sequences was 3.8% divergent from *C*. *nana* ([KX808059](https://www.ncbi.nlm.nih.gov/nuccore/1199744613)). | We could not identify this specimen. This specimen was morphologically similar to *Callophyllis* spp.; however, *rbc*L and *cox*1 analyses revealed that this specimen was distinct from *Callophyllis* species, including *C*. *adnata*, *C*. *mollitia*, and *C*. *repens*.  Id: U3 |
| *Croisettea kalaukapuae* F.P.Cabrera & A.R.Sherwood (S11 Fig. in S4 File and S6 File) | 30 May 2019; R. Terada; TNS AL-220766  *rbc*L: [LC821259](https://www.ncbi.nlm.nih.gov/nuccore/LC821259)  *cox*1: [LC821020](https://www.ncbi.nlm.nih.gov/nuccore/LC821020) | This species is a new record for Japan. The details of the morpho-anatomical observations and identification of this specimen is presented in S6 File. | *rbc*L: *Croisettea kalaukapuae* ([OM621858](https://www.ncbi.nlm.nih.gov/nuccore/OM621858); 99.8%)  *cox*1: *Croisettea kalaukapuae* ([OM509716](https://www.ncbi.nlm.nih.gov/nuccore/OM509716); 98.8%) | The *rbc*L analyses suggested that these specimens form a clade with *C*. *kalaukapuae* from Hawaii, U.S.A. with full statistical supports (S42A Fig. in S5 File). The *rbc*L sequences were 0.2% divergent from the holotype of *C*. *kalaukapuae* ([OM621858](https://www.ncbi.nlm.nih.gov/nuccore/OM621858)). The *cox*1 analyses suggested that these specimens form a clade with *C*. *kalaukapuae* from Hawaii, U.S.A. with low to high statistical supports (less than 50% BP and 1.00 PP; S42B Fig. in S5 File). The *cox*1 sequences were 1.2–1.5% divergent from the holotype and other specimens ([OM509716](https://www.ncbi.nlm.nih.gov/nuccore/OM509716), [OM509718](https://www.ncbi.nlm.nih.gov/nuccore/OM509718)). | This specimen was identified as *C*. *kalaukapuae*. The identification was supported by both morphological and genetic identification.  Id: C1 |
|  | 30 May 2019; R. Terada; TNS AL-220761  *rbc*L: [LC821260](https://www.ncbi.nlm.nih.gov/nuccore/LC821260)  *cox*1: [LC821021](https://www.ncbi.nlm.nih.gov/nuccore/LC821021) |  |  |  |  |
| *Croisettea* sp. TNE (S10 Fig. in S4 File) | 26 June 2021; R. Terada; TNS AL-222077  *rbc*L: [LC821261](https://www.ncbi.nlm.nih.gov/nuccore/LC821261)  *cox*1: [LC821022](https://www.ncbi.nlm.nih.gov/nuccore/LC821022) | The habit and vegetative anatomy of this specimen were similar to those of *C*. *kalaukapuae* [59,this study]. | *rbc*L: *Croisettea* sp.1 LH ([KF280989](https://www.ncbi.nlm.nih.gov/nuccore/KF280989); 96.3%)  *cox*1: *Croisettea* sp.3 Tas ([HM917479](https://www.ncbi.nlm.nih.gov/nuccore/HM917479); 93.2%) | The *rbc*L and *cox*1 sequences did not closely match the INSD data and were distant from various other *Croisettea* species (S42 Fig. in S5 File). | We could not identify this specimen. This specimen was morphologically identified as *C*. *kalaukapuae* offshore Tanegashima Island; however, *rbc*L and *cox*1 analyses revealed that this specimen was distinct from *C*. *kalaukapuae* and various *Croisettea* species.  Id: U2 |
| “*Kallymenia*” *perfolata* J.Agardh (S10 Fig. in S4 File) | 20 July 2017; R. Terada; TNS AL-209813  *rbc*L: [LC821263](https://www.ncbi.nlm.nih.gov/nuccore/LC821263)  *cox*1: [LC821024](https://www.ncbi.nlm.nih.gov/nuccore/LC821024) | The membranous blades with numerous perforations of this specimen were in accordance with those of *Kallymenia perfolata* recorded in Japan [3,45] | *rbc*L: *Leiomenia lacunata* ([KX783113](https://www.ncbi.nlm.nih.gov/nuccore/KX783113); 98.6%)  *cox*1: *Leiomenia lacunata* ([KX808091](https://www.ncbi.nlm.nih.gov/nuccore/KX808091); 96.4%) | The combined *rbc*L and *cox*1 analyses suggested that the specimens assigned to *K*. *perfolata* from Japan are included in the *Leiomenia* clade and form a clade with *L*. *lacunata* from Western Australia with full statistical supports (S40 Fig. in S5 File). The *rbc*L sequence was 0.3% divergent from the Japanese sequence assigned to *K*. *perfolata* ([LC486684](https://www.ncbi.nlm.nih.gov/nuccore/LC486684)), whereas the *cox*1 sequence was 0.2% divergent from the Japanese sequence assigned to *K*. *perfolata* ([LC486688](https://www.ncbi.nlm.nih.gov/nuccore/LC486688)). The *rbc*L sequence was 1.4–1.7% divergent from *L*. *lacunata* ([KX783113](https://www.ncbi.nlm.nih.gov/nuccore/KX783113)), whereas the *cox*1 sequence was 3.6–3.8% divergent from *L*. *lacunata* ([KX808091](https://www.ncbi.nlm.nih.gov/nuccore/KX808091)). | Although the *rbc*L and *cox*1 sequences did not closely match the INSD data, we identified this specimen as *K*. *perfolata* because it was morphologically similar to *K*. *perfolata*. This species is currently classified under *Kallymenia*. However, *rbc*L and *cox*1 analyses revealed that this species is included in the *Leiomenia* clade. Further morphological and molecular analyses are needed to clarify the genetic status.  Id: C2 |
| Kallymeniaceae sp. TNE (S10 Fig. in S4 File) | 28 September 2017; R. Terada; TNS AL-220767  *rbc*L: [LC821262](https://www.ncbi.nlm.nih.gov/nuccore/LC821262)  *cox*1: [LC821023](https://www.ncbi.nlm.nih.gov/nuccore/LC821023) | The membranous blade with spine-like protuberances on the margins of this specimen was similar to that of *Kallymenia sagamiana* recorded in Japan [3,20]; however, it was smaller and thinner than *K*. *sagamiana*. | *rbc*L: *Huonia sandersonii* ([KC157631](https://www.ncbi.nlm.nih.gov/nuccore/KC157631); 93.8%)  *cox*1: *Huonia sandersonii* ([HM917780](https://www.ncbi.nlm.nih.gov/nuccore/HM917780); 92.3%) | The combined *rbc*L and *cox*1 analyses suggested that this specimen forms a clade with *Huonia sandersonii* from Tasmania, Australia, with high statistical supports (94% BP and 1.00 PP; S40 Fig. in S5 File). The *rbc*L sequence was 6.2% divergent from *H*. *sandersonii* ([KC157631](https://www.ncbi.nlm.nih.gov/nuccore/KC157631)), whereas the *cox*1 sequences was 7.7% divergent from *H*. *sandersonii* ([HM917780](https://www.ncbi.nlm.nih.gov/nuccore/HM917780)). | We could not identify this specimen. Morphologically, this specimen was similar to *K*. *sagamiana*. However, the size of this specimen differed from that of *K*. *sagamiana*, and molecular analyses revealed that it was distant from various kallymeniacean genera.  Id: U1 |
| *Psaromenia* sp.1 JP (*Kallymenia* cf. *crassiuscula*; S10 Fig. in S4 File) | 20 July 2017; R. Terada; TNS AL-209816  *rbc*L: [LC821265](https://www.ncbi.nlm.nih.gov/nuccore/LC821265)  *cox*1: [LC821026](https://www.ncbi.nlm.nih.gov/nuccore/LC821026) | The habit and vegetative anatomy of this specimen is in accrdance with those of *Kallymenia crassiuscula* recorded in Japan and South Korea [3,49,55]. | *rbc*L: *Psaromenia* sp.1 Jeju ([KC157636](https://www.ncbi.nlm.nih.gov/nuccore/KC157636); 99.8%)  *cox*1: *Psaromenia* sp.1 Jeju ([KC157623](https://www.ncbi.nlm.nih.gov/nuccore/KC157623); 99.2%) | The combined *rbc*L and *cox*1 analyses suggested that specimens assigned to *K*. *crassiuscula* form a clade with *Psaromenia* sp.1 Jeju from South Korea with full statistical supports and are included in the *Psaromenia* clade (S40 Fig. in S5 File). The specimens assigned to *K*. *crassiuscula* were divided into two lineages: *P*. sp.1 from Japan and south Korea and *P*. sp.2 Tane collected from offshore Tanegashima Island. The *rbc*L sequence was 0.2% divergent from *P*. sp.1 Jeju ([KC157636](https://www.ncbi.nlm.nih.gov/nuccore/KC157636)), whereas the *cox*1 sequence was 0.8% divergent from *P*. sp.1 Jeju ([KC157623](https://www.ncbi.nlm.nih.gov/nuccore/KC157623)). The *rbc*L sequence of *P*. sp.1 was 8.2% divergent from *P*. sp.2 Tane ([LC821266](https://www.ncbi.nlm.nih.gov/nuccore/LC821266)), whereas the *cox*1 sequence was 6.3–6.6% divergent from *P*. sp.2 Tane ([LC821027](https://www.ncbi.nlm.nih.gov/nuccore/LC821027)). | We tentatively identified this specimen as *K*. cf. *crassiuscula*. Morphologically, this specimen was identified as *K*. *crassiuscula*. However, molecular analyses revealed that it was included in the *Psaromenia* clade, suggesting that *K.* *crassiuscula* is not a *Kallymenia* species or its misapplied name in Japan. The *rbc*L analyses indicated that it is conspecific with *P*. sp.1 Jeju. According to Lee & Kim [60], *P*. sp.1 Jeju is corresponding to *P*. *stipitata*; however, the *rbc*L sequences of *P*. *stipitata* used in Lee & Kim [60] were not available in INSD. Morphology of *P*. *stipitata* is also similar to *K*. *crassiuscula*. Further morpho-anatomical and molecular analyses, including the type specimens of *K*. *crassiuscula* or samples from a type locality (Mie, Japan) are needed to clarify its identification and genetic status.  Id: T |
| *Psaromenia* sp.2 TNE (*Kallymenia* cf. *crassiuscula*; S10 Fig. in S4 File) | 20 July 2017; R. Terada; TNS AL-220762  *rbc*L: [LC821266](https://www.ncbi.nlm.nih.gov/nuccore/LC821266)  *cox*1: [LC821027](https://www.ncbi.nlm.nih.gov/nuccore/LC821027) | The habit and vegetative anatomy of this specimen were similar to those of *K*. *crassiuscula* and *Psaromenia* sp.1 JP [3,49,55,this study]; however, it was smaller than *P*. sp.1 JP. | *rbc*L: *Psaromenia* sp.1 Jeju ([KC157636](https://www.ncbi.nlm.nih.gov/nuccore/KC157636); 92.1%)  *cox*1: *Psaromenia laulamaula* ([MW250210](https://www.ncbi.nlm.nih.gov/nuccore/MW250210); 93.6%) | The combined *rbc*L and *cox*1 analyses suggested that this specimen is distinct from *P*. sp.1 and distant from various other *Psaromenia* species (S40 Fig. in S5 File). | We tentatively identified this specimen as *K*. cf. *crassiuscula*, as well as *P*. sp.1 JP. Morphologically, both this specimen and *P*. sp.1 JP were identified as *K*. *crassiuscula*. However, *rbc*L and *cox*1 analyses indicated that this specimen was distinct from *P*. sp.1 JP, suggesting that *K*. *crassiuscula* includes two cryptic species. We could not determine which corresponds to true *K*. *crassiuscula*, and they were treated as *P*. sp.1 (*K*. cf. *crassiuscula*) and *P*. sp.2 (*K*. cf. *crassiuscula*). Further morpho-anatomical and molecular analyses, including the type specimens of *K*. *crassiuscula* or samples from a type locality (Mie, Japan) are required to clarify its identification and genetic status.  Id: T |
| Rhodymeniophycidae, Gigartinales, Phyllophoraceae | | | | | |
| *Stenogramma guleopoense* M.S.Calderón & S.M.Boo (S12 Fig. in S4 File and S6 File) | 19 May 2016; R. Terada; TNS AL-209831  *rbc*L: [LC473161](https://www.ncbi.nlm.nih.gov/nuccore/LC473161)  *cox*1: [LC473171](https://www.ncbi.nlm.nih.gov/nuccore/LC473171) | This species is a new record for Japan. The details of the morpho-anatomical observations and identification of this specimen is presented in S6 File. | *rbc*L: *Stenogramma guleopoense* ([MF576328](https://www.ncbi.nlm.nih.gov/nuccore/MF576328); 99.8%)  *cox*1: *Stenogramma californicum* ([GQ380375](https://www.ncbi.nlm.nih.gov/nuccore/GQ380375); 96.2%) | The *rbc*L analyses suggested that these specimens form a clade with *S*. *guleopoense* from South Korea with full statistical supports (S43A Fig. in S5 File). The *rbc*L sequences were 0.2% divergent from *S*. *guleopoense* from South Korea ([MF576328](https://www.ncbi.nlm.nih.gov/nuccore/MF576328)). The combined *rbc*L and *cox*1 analyses suggested that *S*. *guleopoense* is distant from various other *Stenogramma* species (S43B Fig. in S5 File). | This specimen was identified as *S*. *guleopoense*. The identification was supported by both morphological and genetic identification.  Id: C1 |
|  | 21 July 2017; R. Terada; TNS AL-209834  *rbc*L: [LC473162](https://www.ncbi.nlm.nih.gov/nuccore/LC473162)  *cox*1: [LC473172](https://www.ncbi.nlm.nih.gov/nuccore/LC473172) |  |  |  |  |
|  | 28 September 2017; R. Terada; TNS AL-209835  *rbc*L: [LC473163](https://www.ncbi.nlm.nih.gov/nuccore/LC473163)  *cox*1: [LC473173](https://www.ncbi.nlm.nih.gov/nuccore/LC473173) |  |  |  |  |
|  | 18 May 2016; R. Terada; TNS AL-209830  *rbc*L: [LC473164](https://www.ncbi.nlm.nih.gov/nuccore/LC473164)  *cox*1: [LC821033](https://www.ncbi.nlm.nih.gov/nuccore/LC821033) |  |  |  |  |
|  | 20 July 2017; R. Terada; TNS AL-209832  *rbc*L: [LC473165](https://www.ncbi.nlm.nih.gov/nuccore/LC473165)  *cox*1: [LC821034](https://www.ncbi.nlm.nih.gov/nuccore/LC821034) |  |  |  |  |
| *Stenogramma lamyi* L.LeGall (S13 Fig. in S4 File and S6 File) | 28 September 2017; R. Terada; TNS AL-209842  *rbc*L: [LC473167](https://www.ncbi.nlm.nih.gov/nuccore/LC473167)  *cox*1: [LC473175](https://www.ncbi.nlm.nih.gov/nuccore/LC473175) | This species is a new record for Japan. The details of the morpho-anatomical observations and identification of this specimen is presented in S6 File. | *rbc*L: *Stenogramma lamyi* ([KR733113](https://www.ncbi.nlm.nih.gov/nuccore/KR733113); 100%)  *cox*1: *Stenogramma lamyi* ([KR733111](https://www.ncbi.nlm.nih.gov/nuccore/KR733111); 99.4%) | The *rbc*L sequences were 100% identical to the holotype of *S*. *lamyi* from Madagascar ([KR733113](https://www.ncbi.nlm.nih.gov/nuccore/KR733113)), whereas the *cox*1 sequence was 0.6% divergent from the holotype ([KR733111](https://www.ncbi.nlm.nih.gov/nuccore/KR733111)). The combined *rbc*L and *cox*1 analyses suggested that *S*. *lamyi* is distant from various other *Stenogramma* species (S43B Fig. in S5 File). | Although the habits and vegetative anatomy of Japanese specimens are different from those of Malagasy specimens, we identified these *Stenogramma* specimens from Japan as *S*. *lamyi* based on the low divergence of *rbc*L and *cox*1 sequences between Japanese and Malagasy specimens.  Id: C3 |
|  | 25 June 2021; R. Terada; TNS AL-222091  *rbc*L: [LC821272](https://www.ncbi.nlm.nih.gov/nuccore/LC821272) |  |  |  |  |
|  | 26 June 2021; R. Terada; TNS AL-215753  *rbc*L: [LC821273](https://www.ncbi.nlm.nih.gov/nuccore/LC821273) |  |  |  |  |
|  | 3 October 2021; R. Terada; TNS AL-222097  *rbc*L: [LC821274](https://www.ncbi.nlm.nih.gov/nuccore/LC821274) |  |  |  |  |
|  | 2 October 2021; R. Terada; TNS AL-222120  *rbc*L: [LC821275](https://www.ncbi.nlm.nih.gov/nuccore/LC821275) |  |  |  |  |
|  | 16 May 2022; R. Terada; TNS AL-222185  *rbc*L: [LC821276](https://www.ncbi.nlm.nih.gov/nuccore/LC821276) |  |  |  |  |
| Rhodymeniophycidae, Gigartinales, Solieriaceae | | | | | |
| *Solieria pacifica* (Yamada) T.Yoshida (S10 Fig. in S4 File) | 15 May 2021; R. Terada; TNS AL-215754  *rbc*L: [LC821278](https://www.ncbi.nlm.nih.gov/nuccore/LC821278)  *cox*1: [LC821036](https://www.ncbi.nlm.nih.gov/nuccore/LC821036) | The habit and vegetative anatomy of this specimen were in accordance with those of *S*. *pacifica* recorded in Japan [3,61 as *Chrysymenia pacifica*]. | *rbc*L: *Solieria pacifica* ([LC217307](https://www.ncbi.nlm.nih.gov/nuccore/LC217307); 100%)  *cox*1: *Solieria pacifica* ([LC821035](https://www.ncbi.nlm.nih.gov/nuccore/LC821035); 98.4%) | The combined *rbc*L and *cox*1 analyses suggested that this specimen forms a clade with *S*. *pacifica* from Chiba, Japan, with full statistical supports (S44 Fig. in S5 File). The *rbc*L sequence was 100% identical to *S*. *pacifica* ([LC217307](https://www.ncbi.nlm.nih.gov/nuccore/LC217307)), whereas the *cox*1 sequences was 1.6% divergent from *S*. *pacifica* ([LC821035](https://www.ncbi.nlm.nih.gov/nuccore/LC821035)). | This specimen was identified as *S*. *pacifica*. The identification was supported by both morphological and genetic identification.  Id: C1 |
| Rhodymeniophycidae, Gracilariales, Gracilariaceae | | | | | |
| *Gracilaria punctata* (Okamura) Yamada (S10 Fig. in S4 File) | 18 May 2016; R. Terada; TNS AL-209781  *rbc*L: [LC589292](https://www.ncbi.nlm.nih.gov/nuccore/LC589292)  *cox*1: [LC589307](https://www.ncbi.nlm.nih.gov/nuccore/LC589307) | The habit and vegetative anatomy of this specimen were in accordance with those of *G*. *punctata* recorded in Japan and Taiwan [3,62 as *Rhodymenia punctata*,63]. | *rbc*L: *Gracilaria punctata* ([AY737447](https://www.ncbi.nlm.nih.gov/nuccore/AY737447); 99.6%)  *cox*1: *Gracilaria caudata* ([MZ336078](https://www.ncbi.nlm.nih.gov/nuccore/MZ336078); 90.2%) | The *rbc*L sequence was 0.4% divergent from *G*. *punctata* from Taiwan ([AY737447](https://www.ncbi.nlm.nih.gov/nuccore/AY737447)). The *cox*1 sequence did not closely match the INSD data and was distant from various other *Gracilaria* species (S45B, C Fig. in S5 File). | This specimen was identified as *G*. *punctata*. The identification was supported by both morphological and genetic identification.  Id: C1 |
|  | 29 September 2022; R. Terada; TNS AL-222219  *cox*1: [LC821038](https://www.ncbi.nlm.nih.gov/nuccore/LC821038) |  |  |  |  |
| *Gracilaria sublittoralis* Yamada & Segawa ex H.Yamamoto (S10 Fig. in S4 File) | 20 July 2017; R. Terada; TNS AL-220696  *rbc*L: [LC821287](https://www.ncbi.nlm.nih.gov/nuccore/LC821287)  *cox*1: [LC821045](https://www.ncbi.nlm.nih.gov/nuccore/LC821045) | The flattened blade, large medullary cells, and deep spermatangial conceptacles of this specimen were in accordance with those of *G*. sublittoralis recorded in Japan [3,64,65]. | *rbc*L: *Gracilaria* sp. ([AY049384](https://www.ncbi.nlm.nih.gov/nuccore/AY049384); 96.9%)  *cox*1: *Gracilaria* sp. DBP1 ([MG199582](https://www.ncbi.nlm.nih.gov/nuccore/MG199582); 94.2%) | The *rbc*L analyses suggested that this specimen forms a clade with *G*. sp. from Philippines and *G*. sp. CUPVOUCHER-Gr-2020-1 from India, with full statistical supports (S45A Fig. in S5 File). The *rbc*L sequence was 3.1–3.5% divergent from the other two taxa ([AY049384](https://www.ncbi.nlm.nih.gov/nuccore/AY049384), [MT939894](https://www.ncbi.nlm.nih.gov/nuccore/MT939894)). The *cox*1 analyses suggested that this specimen forms a clade with *G*. sp. DBP1 from Philippines and *G*. sp. CUPVOUCHER-Gr-2020-1 from India, with high statistical supports (88% BP and 1.00 PP; S45B Fig. in S5 File). The *cox*1 sequence was 5.8–6.1% divergent from the other two taxa ([MG199582](https://www.ncbi.nlm.nih.gov/nuccore/MG199582), [MT939889](https://www.ncbi.nlm.nih.gov/nuccore/MT939889)). | Although the *rbc*L and *cox*1 sequences did not closely match the INSD data, we identified this specimen as *G*. sublittoralis morphologically in accordance with the *G*. sublittoralis was described in Japan.  Id: C2 |
| *Gracilaria* sp.1 TNE (S10 Fig. in S4 File) | 20 July 2017; R. Terada; TNS AL-220692  *rbc*L: [LC821286](https://www.ncbi.nlm.nih.gov/nuccore/LC821286)  *cox*1: [LC821044](https://www.ncbi.nlm.nih.gov/nuccore/LC821044) | The flattened and linear blades of this specimen were similar to those of *G*. cuneifolia recorded in Japan and South Korea [3,66], and also *G*. webervanbosseae recorded in Australia [7]. | *rbc*L: *Gracilaria webervanbosseae* ([KY783427](https://www.ncbi.nlm.nih.gov/nuccore/KY783427); 98.6%)  *cox*1: *Gracilaria parva* ([KY656553](https://www.ncbi.nlm.nih.gov/nuccore/KY656553); 91.8%) | The *rbc*L analyses suggested that this specimen forms a clade with *G*. *webervanbosseae* Huisman & G.W.Saunders from Western Australia, with high statistical supports (97% BP and 1.00 PP; S45A Fig. in S5 File). The *rbc*L sequences were 1.4% divergent from *G*. *webervanbosseae* ([KY783427](https://www.ncbi.nlm.nih.gov/nuccore/KY783427)). The *cox*1 sequence did not closely match the INSD data and was distant from various other *Gracilaria* species (S45B, C Fig. in S5 File). | We could not identify this specimen. This specimen was morphologically similar to *Gracilaria* spp.; however, *rbc*L and *cox*1 analyses revealed that this specimen was distinct from *Gracilaria* species, including *G*. cf. cuneifolia, and *G*. webervanbosseae.  Id: U3 |
| *Gracilaria* sp.2 TNE (S14 Fig. in S4 File) | 18 May 2016; R. Terada; TNS AL-220698  *rbc*L: [LC821288](https://www.ncbi.nlm.nih.gov/nuccore/LC821288)  *cox*1: [LC821046](https://www.ncbi.nlm.nih.gov/nuccore/LC821046) | The small, flattened, and linear blades of this specimen were similar to those of *G*. *canaliculata* recorded in Taiwan, Hawaii, and Australia [5,7,63]; however, it differed from *G*. *canaliculata* by few branching. | *rbc*L: *Gracilaria cearensis* ([MZ336048](https://www.ncbi.nlm.nih.gov/nuccore/MZ336048); 94.2%)  *cox*1: *Gracilaria* sp. ARS 03323 ([HQ422773](https://www.ncbi.nlm.nih.gov/nuccore/HQ422773); 99.5%) | The *rbc*L sequence did not closely match the INSD data and was distant from various other *Gracilaria* species (S45A Fig. in S5 File). The *cox*1 sequence was 99.5% identical to *G*. sp. ARS 03323 from Hawaii, U.S.A. ([HQ422773](https://www.ncbi.nlm.nih.gov/nuccore/HQ422773)). | We could not identify this specimen because of the lack of similar morphological species recorded in the Pacific and close sequence data in INSD. The *cox*1 analyses indicated that it is conspecific with *G*. sp. ARS 03323.  Id: U1 |
| *Gracilaria* sp.3 TNE (*G*. cf. *articulata*; S14 Fig. in S4 File) | 21 July 2017; R. Terada; TNS AL-209783  *rbc*L: [LC821289](https://www.ncbi.nlm.nih.gov/nuccore/LC821289)  *cox*1: [LC821047](https://www.ncbi.nlm.nih.gov/nuccore/LC821047) | The cylindrical thalli and constriction at the base of branches, and presence of articulation of this specimen were in accordance with those of *G*. *articulata* recorded in Japan and China [67,68]. | *rbc*L: *Gracilaria articulata* ([AY769259](https://www.ncbi.nlm.nih.gov/nuccore/AY769259); 97.0%)  *cox*1: *Gracilaria* shimodensis ([LC821039](https://www.ncbi.nlm.nih.gov/nuccore/LC821039); 92.8%) | The *rbc*L analyses suggested that this specimen forms a clade with *G*. *articulata* from Malaysia with full statistical supports (S45A Fig. in S5 File). The *rbc*L sequences were 3.0% divergent from *G*. *articulata* ([AY769259](https://www.ncbi.nlm.nih.gov/nuccore/AY769259)), suggesting that this specimen is distinct from Malaysian *G*. *articulata*. The *cox*1 sequence lacked a close match to data in INSD and was distant from various other *Gracilaria* species (S45B Fig. in S5 File). | We tentatively identified this specimen as *G*. cf. *articulata*. Morphologically, the specimen was identified as *G*. *articulata*. However, *rbc*L analyses revealed that *G*. *articulata* recorded in Japan is distinct from Malaysian *G*. *articulata*, suggesting that *G*. *articulata* includes two cryptic species. The *rbc*L sequence of *G*. *articulata* from type locality (China) is available ([OP669555](https://www.ncbi.nlm.nih.gov/nuccore/OP669555)); however, the sequence length is short (121 bp), and 100% identical with Japanese and Malaysian *G*. *articulata*. Further morphological and molecular analyses, including the type specimen of *G*. *articulata* or additional samples from a type locality are needed to clarify its identification.  Id: T |
| *Gracilaria* sp.4 TNE (S14 Fig. in S4 File) | 30 May 2019; R. Terada; TNS AL-222050  *rbc*L: [LC821290](https://www.ncbi.nlm.nih.gov/nuccore/LC821290)  *cox*1: [LC821048](https://www.ncbi.nlm.nih.gov/nuccore/LC821048) | The flattened blade and short stalk of this specimen were similar to those of *G*. *huangi*, *G*. *taiwanensis*, and *G*. *vieillardii*, recorded in Japan, Taiwan, and Australia [3,63,69]; however, it differs from *Gracilaria* species recorded in the Pacific because of the absence of marginal spines. | *rbc*L: *Gracilaria textorii* ([LC821281](https://www.ncbi.nlm.nih.gov/nuccore/LC821281); 97.6%)  *cox*1: *Gracilaria* textorii ([KF214690](https://www.ncbi.nlm.nih.gov/nuccore/KF214690); 93.3%) | The *rbc*L and *cox*1 sequences did not closely match the INSD data and were distant from various other *Gracilaria* species (S45 Fig. in S5 File). | We could not identify this specimen because of the lack of similar morphological species recorded in the Pacific and close sequence data in INSD.  Id: U1 |
| *Gracilariopsis mageshimensis* Mas.Suzuki & R.Terada (S14 Fig. in S4 File) | 26 May 2018; R. Terada & M. Suzuki; TNS AL-213913  *rbc*L: [LC589289](https://www.ncbi.nlm.nih.gov/nuccore/LC589289)  *cox*1: [LC589300](https://www.ncbi.nlm.nih.gov/nuccore/LC589300) |  |  | All sequences were identical. | This species was described from offshore of Tanegashima Island (as Mageshima Island) [70].  Id: C1 |
|  | 20 July 2017; R. Terada; TNS AL-213795  *rbc*L: [LC589285](https://www.ncbi.nlm.nih.gov/nuccore/LC589285)  *cox*1: [LC589296](https://www.ncbi.nlm.nih.gov/nuccore/LC589296) |  |  |  |  |
|  | 20 July 2017; R. Terada; TNS AL-213796  *rbc*L: [LC589286](https://www.ncbi.nlm.nih.gov/nuccore/LC589286)  *cox*1: [LC589297](https://www.ncbi.nlm.nih.gov/nuccore/LC589297) |  |  |  |  |
|  | 20 July 2017; R. Terada; TNS AL-213797  *rbc*L: [LC589287](https://www.ncbi.nlm.nih.gov/nuccore/LC589287)  *cox*1: [LC589298](https://www.ncbi.nlm.nih.gov/nuccore/LC589298) |  |  |  |  |
|  | 20 July 2017; R. Terada; TNS AL-213798  *rbc*L: [LC589288](https://www.ncbi.nlm.nih.gov/nuccore/LC589288)  *cox*1: [LC589299](https://www.ncbi.nlm.nih.gov/nuccore/LC589299) |  |  |  |  |
|  | 20 July 2017; R. Terada; TNS AL-214466  *rbc*L: [LC821292](https://www.ncbi.nlm.nih.gov/nuccore/LC821292)  *cox*1: [LC821049](https://www.ncbi.nlm.nih.gov/nuccore/LC821049) |  |  |  |  |
|  | 30 May 2019; R. Terada; TNS AL-213799  *rbc*L: [LC589283](https://www.ncbi.nlm.nih.gov/nuccore/LC589283)  *cox*1: [LC589294](https://www.ncbi.nlm.nih.gov/nuccore/LC589294) |  |  |  |  |
|  | 30 May 2019; R. Terada; TNS AL-213800  *rbc*L: [LC589284](https://www.ncbi.nlm.nih.gov/nuccore/LC589284)  *cox*1: [LC589295](https://www.ncbi.nlm.nih.gov/nuccore/LC589295) |  |  |  |  |
|  | 16 May 2021; R. Terada; TNS AL-215792  *cox*1: [LC821050](https://www.ncbi.nlm.nih.gov/nuccore/LC821050) |  |  |  |  |
|  | 26 June 2021; R. Terada; TNS AL-215793  *rbc*L: [LC821293](https://www.ncbi.nlm.nih.gov/nuccore/LC821293)  *cox*1: [LC821051](https://www.ncbi.nlm.nih.gov/nuccore/LC821051) |  |  |  |  |
|  | 26 June 2021; R. Terada; TNS AL-215794  *rbc*L: [LC821294](https://www.ncbi.nlm.nih.gov/nuccore/LC821294) |  |  |  |  |
|  | 3 October 2021; R. Terada; TNS AL-222100  *rbc*L: [LC821295](https://www.ncbi.nlm.nih.gov/nuccore/LC821295) |  |  |  |  |
| Rhodymeniophycidae, Halymeniales, Grateloupiaceae | | | | | |
| *Yonagunia taiwani-borealis* ShoweM.Lin, Y.C.Chuang & De Clerck (S15 Fig. in S4 File and S6 File) | 18 May 2016; R. Terada; TNS AL-214472  *rbc*L: [LC821303](https://www.ncbi.nlm.nih.gov/nuccore/LC821303) | This species is a new record for Japan. The details of the morpho-anatomical observations and identification of this specimen is presented in S6 File. | *rbc*L: *Yonagunia taiwani-borealis* ([MT501503](https://www.ncbi.nlm.nih.gov/nuccore/MT501503); 100%)  *cox*1: *Yonagunia zollingeri* ([JX627438](https://www.ncbi.nlm.nih.gov/nuccore/JX627438); 96.7%) | The *rbc*L analyses suggested that these specimens form a clade with *Y*. *taiwani-borealis* from Taiwan with moderate to high statistical supports (87% BP and 0.98 PP; S46 Fig. in S5 File). The *rbc*L sequences were identical to the holotype of *Y*. *taiwani-borealis* ([MT501503](https://www.ncbi.nlm.nih.gov/nuccore/MT501503)) and 0.1% divergent from other specimen collected from Taiwan ([MT501502](https://www.ncbi.nlm.nih.gov/nuccore/MT501502)). The *cox*1 sequence lacked a close match to data in INSD and was distant from various other *Yonagunia* species (S47C Fig. in S5 File). | This specimen was identified as *Y*. *taiwani-borealis*. The identification was supported by both morphological and genetic identification.  Id: C1 |
|  | 25 May 2018; R. Terada & M. Suzuki; TNS AL-214473  *rbc*L: [LC821304](https://www.ncbi.nlm.nih.gov/nuccore/LC821304)  *cox*1: [LC821057](https://www.ncbi.nlm.nih.gov/nuccore/LC821057) |  |  |  |  |
|  | 30 May 2019; R. Terada; TNS AL-214474  *rbc*L: [LC821305](https://www.ncbi.nlm.nih.gov/nuccore/LC821305) |  |  |  |  |
|  | 16 May 2021; R. Terada; TNS AL-215816  *rbc*L: [LC821306](https://www.ncbi.nlm.nih.gov/nuccore/LC821306)  *cox*1: [LC821058](https://www.ncbi.nlm.nih.gov/nuccore/LC821058) |  |  |  |  |
|  | 29 September 2022; R. Terada; TNS AL-222218  *rbc*L: [LC821307](https://www.ncbi.nlm.nih.gov/nuccore/LC821307) |  |  |  |  |
| *Yonagunia* sp. TNE (S14 Fig. in S4 File) | 15 May 2021; R. Terada; TNS AL-220700  *rbc*L: [LC821308](https://www.ncbi.nlm.nih.gov/nuccore/LC821308)  *cox*1: [LC821059](https://www.ncbi.nlm.nih.gov/nuccore/LC821059) | The flattened, subdichotomously branched axes of this specimen were similar to those of *Y*. *taiwani-borealis* [71,this study]; however it differed from *Y*. *taiwani-borealis* by the relatively short stipe and inconspicuous midrib. | *rbc*L: *Yonagunia taiwani-borealis* ([MT501503](https://www.ncbi.nlm.nih.gov/nuccore/MT501503); 99.2%)  *cox*1: *Yonagunia zollingeri* ([JX627438](https://www.ncbi.nlm.nih.gov/nuccore/JX627438); 96.1%) | The combined *rbc*L and *cox*1 analyses suggested that this specimen forms a clade with *Y*. *taiwani-borealis* from Japan and Taiwan, and *Y*. *zollingeri* from Indonesia, with high statistical supports (89% BP and 1.00 PP; S47C Fig. in S5 File). The *rbc*L sequence was 0.8–1.5% divergent from the other two taxa ([JX627434](https://www.ncbi.nlm.nih.gov/nuccore/JX627434), [MT501502](https://www.ncbi.nlm.nih.gov/nuccore/MT501502), [MT501503](https://www.ncbi.nlm.nih.gov/nuccore/MT501503), [LC821303](https://www.ncbi.nlm.nih.gov/nuccore/LC821303)– [LC821307](https://www.ncbi.nlm.nih.gov/nuccore/LC821307)), whereas the *cox*1 sequence was 3.9–4.1% divergent from the other two taxa ([JX627438](https://www.ncbi.nlm.nih.gov/nuccore/JX627438), [LC821057](https://www.ncbi.nlm.nih.gov/nuccore/LC821057), [LC821058](https://www.ncbi.nlm.nih.gov/nuccore/LC821058)). | We could not identify this specimen. Although the morphology and *rbc*L sequence of this species appear to be closely related to those of *Y. taiwani-borealis*, the *cox*1 sequence appeared to be distant from *Y. taiwani-borealis* and other *Yonagunia* species.  Id: U2 |
| Rhodymeniophycidae, Halymeniales, Halymeniaceae | | | | | |
| *Amalthea rubida* H.W.Lee & M.S.Kim (S16 Fig. in S4 File and S6 File) | 20 July 2017; R. Terada; TNS AL-220703  *rbc*L: [LC821309](https://www.ncbi.nlm.nih.gov/nuccore/LC821309) | This species is a new record for Japan. The details of the morpho-anatomical observations and identification of this specimen is presented in S6 File. | *rbc*L: *Amalthea rubida* ([KX879776](https://www.ncbi.nlm.nih.gov/nuccore/KX879776); 99.8%) | The *rbc*L analyses suggested that these specimens form a clade with *A*. *rubida* from South Korea with full statistical supports (S47A Fig. in S5 File). The *rbc*L sequences were 0.2% divergent from the isotype of *A*. *rubida* ([KX879776](https://www.ncbi.nlm.nih.gov/nuccore/KX879776)). | This specimen was identified as *A*. *rubida*. The identification was supported by both morphological and genetic identification.  Id: C1 |
|  | 30 May 2019; R. Terada; TNS AL-220704  *rbc*L: [LC821310](https://www.ncbi.nlm.nih.gov/nuccore/LC821310) |  |  |  |  |
|  | 16 May 2021; R. Terada; TNS AL-222065  *rbc*L: [LC821311](https://www.ncbi.nlm.nih.gov/nuccore/LC821311) |  |  |  |  |
| *Amalthea* sp.1 TNE (S14 Fig. in S4 File) | 20 July 2017; R. Terada; TNS AL-220705  *rbc*L: [LC821312](https://www.ncbi.nlm.nih.gov/nuccore/LC821312)  *cox*1: [LC821060](https://www.ncbi.nlm.nih.gov/nuccore/LC821060) | The habit and vegetative anatomy of this specimen is similar to those of *A*. *rubida* [72,this study]. | *rbc*L: *Amalthea rubida* ([KX879776](https://www.ncbi.nlm.nih.gov/nuccore/KX879776); 96.8%)  *cox*1: *Amalthea rubida* ([MW691060](https://www.ncbi.nlm.nih.gov/nuccore/MW691060); 91.3%) | The combined *rbc*L and *cox*1 analyses suggested that this specimen forms a clade with *A*. *rubida* with full statistical supports (S47C Fig. in S5 File). The *rbc*L sequence was 3.2% divergent from *A*. *rubida* ([KX879776](https://www.ncbi.nlm.nih.gov/nuccore/KX879776), [LC821309](https://www.ncbi.nlm.nih.gov/nuccore/LC821309)), whereas the *cox*1 sequence is 8.7% divergent from *A*. *rubida* ([MW691060](https://www.ncbi.nlm.nih.gov/nuccore/MW691060)). | We could not identify this specimen. This specimen was morphologically similar to *A*. *rubida*; however, *rbc*L and *cox*1 analyses revealed that this specimen was distinct from *A*. *rubida* and various *Amalthea* species.  Id: U2 |
| *Amalthea* sp.2 TNE (S14 Fig. in S4 File) | 20 July 2017; R. Terada; TNS AL-220711  *rbc*L: [LC821313](https://www.ncbi.nlm.nih.gov/nuccore/LC821313)  *cox*1: [LC821061](https://www.ncbi.nlm.nih.gov/nuccore/LC821061) | The habit and vegetative anatomy of this specimen were similar to those of *Amalthea* species recorded in the Pacific [72,73,this study]; however, it is redder than the other species. | *rbc*L: *Amalthea rubida* ([KX879776](https://www.ncbi.nlm.nih.gov/nuccore/KX879776); 94.5%)  *cox*1: *Halymenia californica* ([HQ544224](https://www.ncbi.nlm.nih.gov/nuccore/HQ544224); 90.8%) | The *rbc*L and *cox*1 sequences did not closely match the INSD data and were distant from various other *Amalthea* species (S47 Fig. in S5 File). | We could not identify this specimen. This specimen was morphologically similar to *Amalthea* species; however, *rbc*L and *cox*1 analyses revealed that this specimen was distinct from various *Amalthea* species.  Id: U2 |
| *Amalthea* sp.3 TNE (S14 Fig. in S4 File) | 25 June 2021; R. Terada; TNS AL-220724  *rbc*L: [LC821314](https://www.ncbi.nlm.nih.gov/nuccore/LC821314)  *cox*1: [LC821062](https://www.ncbi.nlm.nih.gov/nuccore/LC821062) | The habit and vegetative anatomy of this specimen were similar to those of *A*. sp.2. TNE [this study]. | *rbc*L: *Amalthea freemaniae* ([KJ606651](https://www.ncbi.nlm.nih.gov/nuccore/KJ606651); 97.1%)  *cox*1: *Halymenia californica* ([HQ544224](https://www.ncbi.nlm.nih.gov/nuccore/HQ544224); 92.6%) | The *rbc*L analyses suggested that this specimen forms a clade with *A*. *freemaniae* with high statistical supports (92% BP and 1.00 PP; S47A Fig. in S5 File). The *rbc*L sequence was 2.9% divergent from *A*. *freemaniae* ([KJ606651](https://www.ncbi.nlm.nih.gov/nuccore/KJ606651)). The *cox*1 sequence lacked a close match to data in INSD and was distant from various other *Amalthea* species (S47B, C Fig. in S5 File). | We could not identify this specimen. This specimen was morphologically similar to *Amalthea* species; however, *rbc*L and *cox*1 analyses revealed that this specimen was distinct from various *Amalthea* species.  Id: U2 |
| *Amalthea* sp.4 TNE (S14 Fig. in S4 File) | 26 June 2021; R. Terada; TNS AL-222081  *rbc*L: [LC821315](https://www.ncbi.nlm.nih.gov/nuccore/LC821315) | The habit and vegetative anatomy of this specimen were similar to those of *Amalthea* species recorded in the Pacific [72,73,this study]; however, it differed from the other species by the presence of undulated blades. | *rbc*L: *Amalthea rubida* ([KX879776](https://www.ncbi.nlm.nih.gov/nuccore/KX879776); 95.8%) | The *rbc*L analyses suggested that this specimen forms a clade with *A*. *rubida* and *A*. sp.1 TNE with high statistical supports (93% BP and 1.00 PP; S47A Fig. in S5 File). The *rbc*L sequence was 3.7–4.2% divergent from the other two taxa ([KX879776](https://www.ncbi.nlm.nih.gov/nuccore/KX879776), [LC821309](https://www.ncbi.nlm.nih.gov/nuccore/LC821309), [LC821312](https://www.ncbi.nlm.nih.gov/nuccore/LC821312)). | We could not identify this specimen because of the lack of similar morphological species recorded in the Pacific and close sequence data in INSD.  Id: U1 |
| *Cryptonemia semiprocumbens* Tak.Tanaka (S14 Fig. in S4 File) | 18 May 2016; R. Terada; TNS AL-213807  *rbc*L: [LC821316](https://www.ncbi.nlm.nih.gov/nuccore/LC821316) | The lack of conspicuous midrib of this specimen was in accordance with *C*. *semiprocumbens* recorded in Japan [3,74]. | *rbc*L: *Cryptonemia asiatica* ([KM272322](https://www.ncbi.nlm.nih.gov/nuccore/KM272322); 99.3%) | The *rbc*L analyses suggested that these specimens form a clade with *C*. *antricola* from Bermuda, *C*. *asiatica* from Japan and South Korea, and *C*. sp. TNE from offshore Tanegashima Island with high statistical supports (99% BP and 1.00 PP; S47A Fig. in S5 File). The *rbc*L sequences were 0.7–1.4% divergent from the other four taxa ([MF782466](https://www.ncbi.nlm.nih.gov/nuccore/MF782466), [KM272322](https://www.ncbi.nlm.nih.gov/nuccore/KM272322), [KM272326](https://www.ncbi.nlm.nih.gov/nuccore/KM272326), [LC821319](https://www.ncbi.nlm.nih.gov/nuccore/LC821319)). | Although the *rbc*L sequences did not closely match the INSD data, we identified this specimen as *C*. *semiprocumbens* morphologically in accordance with the *C*. *semiprocumbens* was described in Japan.  Id: C2 |
|  | 30 May 2019; R. Terada; TNS AL-213356  *rbc*L: [LC821317](https://www.ncbi.nlm.nih.gov/nuccore/LC821317) |  |  |  |  |
|  | 16 May 2022; R. Terada; TNS AL-222172  *rbc*L: [LC821318](https://www.ncbi.nlm.nih.gov/nuccore/LC821318) |  |  |  |  |
| *Cryptonemia* sp. TNE (S17 Fig. in S4 File) | 16 May 2021; R. Terada; TNS AL-215796  *rbc*L: [LC821319](https://www.ncbi.nlm.nih.gov/nuccore/LC821319) | The presence of conspicuous midrib in the blades of this specimen was in accordance with that of *C*. *asiatica* recorded in Japan and South Korea [3,74,75]. | *rbc*L: *Cryptonemia asiatica* ([KM272322](https://www.ncbi.nlm.nih.gov/nuccore/KM272322); 99.5%) | The *rbc*L analyses suggested that this specimen forms a clade with *C*. *antricola* from Bermuda, *C*. *asiatica* from Japan and South Korea, and *C*. *semiprocumbens* from offshore Tanegashima Island with high statistical supports (99% BP and 1.00 PP; S47A Fig. in S5 File). The *rbc*L sequence was 0.5–0.9% divergent the other four taxa ([MF782466](https://www.ncbi.nlm.nih.gov/nuccore/MF782466), [KM272322](https://www.ncbi.nlm.nih.gov/nuccore/KM272322), [KM272326](https://www.ncbi.nlm.nih.gov/nuccore/KM272326), [LC821318](https://www.ncbi.nlm.nih.gov/nuccore/LC821318)). | We could not identify this specimen. This specimen was morphologically similar to *C*. *asiatica*; however, *rbc*L analyses revealed that this specimen was distinct from *C*. *asiatica* and various *Cryptonemia* species.  Id: U2 |
| *Galene* sp.1 TNE (S17 Fig. in S4 File) | 26 May 2018; R. Terada & M. Suzuki; TNS AL-214462  *rbc*L: [LC821320](https://www.ncbi.nlm.nih.gov/nuccore/LC821320)  *cox*1: [LC821063](https://www.ncbi.nlm.nih.gov/nuccore/LC821063) | The vegetative anatomy of this specimen were in accordance with *Galene* species [3,60,73,76]; however, it differed from other species by producing marginal bladelets. | *rbc*L: *Galene* sp.1 LH ([MK812809](https://www.ncbi.nlm.nih.gov/nuccore/MK812809); 97.5%)  *cox*1: *Galene* sp.2 LH ([MK812801](https://www.ncbi.nlm.nih.gov/nuccore/MK812801); 95.2%) | The *rbc*L analyses suggested that this specimen forms a clade with *G*. sp. 1 LH from Lord Howe Island, Australia, with moderate statistical supports (74% BP and 0.99 PP; S47A Fig. in S5 File). The *rbc*L sequence was 2.5% divergent from *G*. sp. 1 LH ([MK812809](https://www.ncbi.nlm.nih.gov/nuccore/MK812809)). The *cox*1 sequence lacked a close match to data in INSD and was distant from various other *Galene* species (S47 Fig in S5 File). | We could not identify this specimen because of the lack of similar morphological species recorded in the Pacific and close sequence data in INSD.  Id: U1 |
| *Galene* sp.2 TNE (S17 Fig. in S4 File) | 15 May 2021; R. Terada; TNS AL-220729  *rbc*L: [LC821321](https://www.ncbi.nlm.nih.gov/nuccore/LC821321) | The round blades of this specimen were similar to those of *G*. *profundae*, *G*. *rotunda*, and *G*. *scutae* recorded in the Pacific [3,22,60,73]. | *rbc*L: *Galene* sp.2 LH ([MK812812](https://www.ncbi.nlm.nih.gov/nuccore/MK812812); 99.2%) | The *rbc*L analyses suggested that this specimen is distinct from *G*. *rotunda* from Japan ([AB061375](https://www.ncbi.nlm.nih.gov/nuccore/AB061375)) and forms a clade with *G*. sp. 2 LH from Lord Howe Island, Australia, with moderate statistical supports (72% BP and 0.99 PP; S47A Fig. in S5 File). The *rbc*L sequence was 0.8% divergent from *G*. sp. 2 LH ([MK812812](https://www.ncbi.nlm.nih.gov/nuccore/MK812812)). | We could not identify this specimen. This specimen was morphologically similar to *Galene* species; however, *rbc*L analyses revealed that this specimen was distinct from various *Galene* species. The *rbc*L analyses indicated that it is conspecific with *G*. sp.2 LH.  Id: U3 |
| *Galene* sp.3 TNE (S17 Fig. in S4 File) | 3 October 2021; R. Terada; TNS AL-222143  *rbc*L: [LC821322](https://www.ncbi.nlm.nih.gov/nuccore/LC821322)  *cox*1: [LC821064](https://www.ncbi.nlm.nih.gov/nuccore/LC821064) | The vegetative anatomy of this specimen were in accordance with *Galene* species [3,60,73,76]; however, it differed from other species by producing many ligulate bladelets. | *rbc*L: *Galene* sp.1 WA ([MK812824](https://www.ncbi.nlm.nih.gov/nuccore/MK812824); 99.0%)  *cox*1: *Galene* sp.1 WA ([MK812804](https://www.ncbi.nlm.nih.gov/nuccore/MK812804); 97.7%) | The combined *rbc*L and *cox*1 analyses suggested that this specimen forms a clade with *G*. sp. 1 WA from Western Australia, Australia, with full statistical supports (S47C Fig. in S5 File). The *rbc*L sequence was 1.0% divergent from *G*. sp. 1 WA ([MK812824](https://www.ncbi.nlm.nih.gov/nuccore/MK812824)), whereas the *cox*1 sequence was 2.3% divergent from *G*. sp. 1 WA ([MK812804](https://www.ncbi.nlm.nih.gov/nuccore/MK812804)). | We could not identify this specimen because of the lack of similar morphological species recorded in the Pacific and close sequence data in INSD. The *rbc*L analyses indicated that it is conspecific with *G*. sp.1 WA.  Id: U1 |
| *Halymenia* sp. TNE (*H*. cf. *durvillei*; S17 Fig. in S4 File) | 20 July 2017; R. Terada; TNS AL-215802  *rbc*L: [LC821323](https://www.ncbi.nlm.nih.gov/nuccore/LC821323)  *cox*1: [LC821065](https://www.ncbi.nlm.nih.gov/nuccore/LC821065) | The habit and vegetative anatomy of this specimen is in accrdance with those of *H*. *durvillei* recorded in Japan [3 as *H*. *floresii*,77 as *H*. *formosa*]. | *rbc*L: *Halymenia plana* ([KU726713](https://www.ncbi.nlm.nih.gov/nuccore/KU726713); 96.4%)  *cox*1: *Halymenia plana* ([MK812804](https://www.ncbi.nlm.nih.gov/nuccore/MK812804); 92.9%) | The *rbc*L analyses suggested that this specimen forms a clade with “*Cryptonemia*” *kallymenioides*, *H*. *johorensis*, and *H*. *plana*, with high statistical supports (85% BP and 1.00 PP; S47A Fig. in S5 File).The *rbc*L sequence was 3.6–4.1% divergent from the other three taxa ([MH311934](https://www.ncbi.nlm.nih.gov/nuccore/MH311934), [KX958402](https://www.ncbi.nlm.nih.gov/nuccore/KX958402), [KU726713](https://www.ncbi.nlm.nih.gov/nuccore/KU726713)). The *cox*1 sequence lacked a close match to data in INSD and was distant from various other *Halymenia* species (S47B, C Fig. in S5 File). | We tentatively identified this specimen as *H*. cf. *durvillei*. Morphologically, the specimen was identified as *H*. *durvillei*. However, *rbc*L and *cox*1analyses revealed that *H*. *durvillei* recorded in Japan is distinct from Filipino and South African *H*. *durvillei* ([MK812821](https://www.ncbi.nlm.nih.gov/nuccore/MK812821), [ABMMC1356-07](https://v3.boldsystems.org/index.php/Public_RecordView?processid=ABMMC1356-07)), suggesting that *H*. *durvillei* includes two to three cryptic species. Further morphological and molecular analyses, including the type specimen of *H*. *durvillei* or samples from a type locality (Papua New Guinea) are needed to clarify its identification.  Id: T |
| Halymeniaceae sp.1 TNE (S17 Fig. in S4 File) | 4 June 2022; R. Terada; TNS AL-222205  *rbc*L: [LC821324](https://www.ncbi.nlm.nih.gov/nuccore/LC821324) | The vegetative anatomy of this specimen were in accordance with *Halymenia* species [3,5-7]; however, it differed from other species by marginal dentations and lack of conspicuous stipe. | *rbc*L: *Felicinia marginata* ([KJ594954](https://www.ncbi.nlm.nih.gov/nuccore/KJ594954); 94.6%) | The *rbc*L sequence lacked a close match to data in INSD and was distant from various other halymeniacean genera (S47A Fig. in S5 File). | We could not identify this specimen because of the lack of similar morphological species recorded in the Pacific and close sequence data in INSD.  Id: U1 |
| Halymeniaceae sp.2 TNE (S17 Fig. in S4 File) | 2 October 2021; R. Terada; TNS AL-222124  *rbc*L: [LC821325](https://www.ncbi.nlm.nih.gov/nuccore/LC821325) | This specimen was morphologically similar to halymeniacean species with membranous thalli recorded in the Pacific [3,5-7,78]. | *rbc*L: “*Halymenia*” sp. IBC 2165 ([MK919027](https://www.ncbi.nlm.nih.gov/nuccore/MK919027); 94.4%) | The *rbc*L sequence lacked a close match to data in INSD and was distant from various other halymeniacean genera (S47A Fig. in S5 File). | We could not identify this specimen. Morphologically, this specimen is similar to membranous halymeniacean species; however, *rbc*L analyses indicated that it is distant from various halymeniacean genera.  Id: U3 |
| Halymeniaceae sp.3 TNE (S17 Fig. in S4 File) | 16 May 2021; R. Terada; TNS AL-220737  *rbc*L: [LC821326](https://www.ncbi.nlm.nih.gov/nuccore/LC821326)  *cox*1: [LC821066](https://www.ncbi.nlm.nih.gov/nuccore/LC821066) | The gelatinous, linear, and dichotomously branched thallus of this specimen did not match any halymeniacean species recorded in the Pacific [3,5-7,78]. | *rbc*L: “*Halymenia*” *elongata* ([KX586168](https://www.ncbi.nlm.nih.gov/nuccore/KX586168); 98.2%)  *cox*1: “*Halymenia*” *elongata* ([KX586156](https://www.ncbi.nlm.nih.gov/nuccore/KX586156); 93.8%) | The combined *rbc*L and *cox*1 analyses suggested that this specimen forms a clade with “*Halymenia*” *elongata* from Brazil, with full statistical supports (S47C Fig. in S5 File). The *rbc*L sequence was 1.8% divergent from “*H*”. *elongata* ([KX586168](https://www.ncbi.nlm.nih.gov/nuccore/KX586168)), whereas the *cox*1 sequence was 6.2% divergent from “*H*”. *elongata* ([KX586156](https://www.ncbi.nlm.nih.gov/nuccore/KX586156)). | We could not identify this specimen because of the lack of similar morphological species recorded in the Pacific and close sequence data in INSD.  Id: U1 |
| Rhodymeniophycidae, Nemastomatales, Schizymeniaceae | | | | | |
| *Platoma* sp. TNE (S18 Fig. in S4 File) | 15 May 2021; R. Terada; TNS AL-220735  *rbc*L: [LC821331](https://www.ncbi.nlm.nih.gov/nuccore/LC821331)  *cox*1: [LC821070](https://www.ncbi.nlm.nih.gov/nuccore/LC821070) | The membranous and unbranched blade of this specimen was similar to those of *P*. *fanii* recorded in Pacific Mexico [79] ; however, it differed from *P*. *fanii* by its thinner thallus and presence of short stipe. | *rbc*L: *Platoma novae-caledoniae* ([KP267708](https://www.ncbi.nlm.nih.gov/nuccore/KP267708); 98.3%)  *cox*1: *Platoma ardreanum* ([HQ422694](https://www.ncbi.nlm.nih.gov/nuccore/HQ422694); 93.0%) | The *rbc*L analyses suggested that this specimen forms a clade with *P*. *novae-caledoniae* with full statistical supports (S48A Fig. in S5 File). The *rbc*L sequence is 1.7% divergent from *P*. *novae-caledoniae* ([KP267708](https://www.ncbi.nlm.nih.gov/nuccore/KP267708)). The *cox*1 sequence lacked a close match to data in INSD and was distant from various other *Platoma* species (S48B, C Fig. in S5 File). | We could not identify this specimen because of the lack of similar morphological species recorded in the Pacific and close sequence data in INSD.  Id: U1 |
| Rhodymeniophycidae, Peyssonneliales, Peyssonneliaceae | | | | | |
| *Agissea* sp.1 TNE (S18 Fig. in S4 File) | 2 October 2021; R. Terada; TNS AL-222096  *rbc*L: [LC821332](https://www.ncbi.nlm.nih.gov/nuccore/LC821332) | The color, crustose, and fan-shaped thallus of this specimen were similar to those of *Peyssonnelia conchicola* recorded in Japan and Hawaii [3,5,80]. | *rbc*L: *Agissea harveyana* ([AB325859](https://www.ncbi.nlm.nih.gov/nuccore/AB325859); 91.5%) | The *rbc*L sequence lacked a close match to data in INSD and was distant from various other *Agissea* species (S49A Fig. in S5 File). | We could not identify this specimen. Morphologically, this specimen is similar to *P*. *conchicola*; however, *rbc*L analyses indicated that it belongs to *Agissea* and distant from Mexican *P*. *conchicola*.  Id: U2 |
| *Agissea* sp.2 TNE (*A*. cf. *orientalis*; S18 Fig. in S4 File) | 16 May 2022; R. Terada; TNS AL-222187  *rbc*L: [LC821333](https://www.ncbi.nlm.nih.gov/nuccore/LC821333) | The non-crustose and fa-shaped thallus of this specimen were in accordance with those of *A*. *orientalis* recorded in Japan [3,80 as *Peyssonnelia orientalis*]. | *rbc*L: *Agissea orientalis* ([AB325865](https://www.ncbi.nlm.nih.gov/nuccore/AB325865); 96.8%) | The *rbc*L analyses suggested that this specimen forms a clade with *A*. *orientalis* from Japan and Hawaii, U.S.A. with high statistical supports (92% BP and 1.00 PP; S49A Fig. in S5 File). The *rbc*L sequence was 3.2–3.5% divergent from the other two taxa ([AB325865](https://www.ncbi.nlm.nih.gov/nuccore/AB325865), [MZ047755](https://www.ncbi.nlm.nih.gov/nuccore/MZ047755)). | We tentatively identified this specimen as *A*. cf. *orientalis*. Morphologically, the specimen was identified as *A*. *orientalis*. However, *rbc*L and *cox*1 analyses revealed that *A*. *orientalis* recorded in Japan and Hawaii includes three cryptic species. Further morphological and molecular analyses, including the type specimen of *A*. *orientalis* or samples from the syntype localities (Philippine Islands; various in Indonesia) are needed to clarify its identification.  Id: T |
| *Incendia* sp. TNE (S18 Fig. in S4 File) | 16 May 2022; R. Terada; TNS AL-222186  *rbc*L: [LC821334](https://www.ncbi.nlm.nih.gov/nuccore/LC821334)  *cox*1: [LC821072](https://www.ncbi.nlm.nih.gov/nuccore/LC821072) | This specimen is morphologically similar to peyssonneliacean species with non-crustose thalli recorded in the Pacific such as *Agissea orientalis* and *Sonderophycus cauliferus* [3,7,8,80]. | *rbc*L: *Incendia regularis* ([JX969803](https://www.ncbi.nlm.nih.gov/nuccore/JX969803); 88.9%)  *cox*1: *Incendia crenata* ([JX969714](https://www.ncbi.nlm.nih.gov/nuccore/JX969714); 87.8%) | The *rbc*L and *cox*1 analyses suggested that this specimen is included in *Incendia* clade (S49 Fig. in S5 File). The *rbc*L and *cox*1 sequences did not closely match the INSD data and were distant from various other *Incendia* species (S49 Fig. in S5 File). | We could not identify this specimen. Morphologically, this specimen is similar to *A*. *orientalis* and *S*. *cauliferus*; however, molecular analyses indicated that it belongs to *Incendia*.  Id: U3 |
| Rhodymeniophycidae, Plocamiales, Plocamiaceae | | | | | |
| *Plocamium brasiliense* (Greville) M.Howe & W.R.Taylor (S19 Fig. in S4 File and S6 File) | 18 May 2016; R. Terada; TNS AL-209776  *rbc*L: [LC821335](https://www.ncbi.nlm.nih.gov/nuccore/LC821335)  *cox*1: [LC821073](https://www.ncbi.nlm.nih.gov/nuccore/LC821073) | This species is a new record for Japan. The details of the morpho-anatomical observations and identification of this specimen is presented in S6 File. | *rbc*L: *Plocamium brasiliense* ([KM974718](https://www.ncbi.nlm.nih.gov/nuccore/KM974718); 99.4%)  *cox*1: *Plocamium nanum* ([JF271605](https://www.ncbi.nlm.nih.gov/nuccore/JF271605); 93.5%) | The *rbc*L sequences were 0.6% divergent from *P*. *brasiliense* from Brazil ([KM974718](https://www.ncbi.nlm.nih.gov/nuccore/KM974718)). The *cox*1 analyses suggested that these specimens form a clade with *P*. sp. Natal1 from South Africa with high statistical supports (97% BP and 1.00 PP; S50B Fig. in S5 File). The *cox*1 sequences were 4.4% divergent from *P*. sp. Natal1 ([ABMMC780-06](https://boldsystems.org/index.php/Public_RecordView?processid=ABMMC780-06)). | This specimen was identified as *P*. *brasiliense*. The identification was supported by both morphological and genetic identification.  Id: C1 |
|  | 15 May 2021; R. Terada; TNS AL-215770  *rbc*L: [LC821336](https://www.ncbi.nlm.nih.gov/nuccore/LC821336)  *cox*1: [LC821074](https://www.ncbi.nlm.nih.gov/nuccore/LC821074) |  |  |  |  |
| Plocamium luculentum M.Y.Yang & M.S.Kim (S18 Fig. in S4 File) | 20 July 2017; R. Terada; TNS AL-209777  *rbc*L: [LC821338](https://www.ncbi.nlm.nih.gov/nuccore/LC821338)  *cox*1: [LC821076](https://www.ncbi.nlm.nih.gov/nuccore/LC821076) | The upper branches produced alternating pairs of 2 ramuli of this specimen were in accordance with those of *P*. luculentum recorded in Japan and South Korea [3,8,66 as *P*. *telfairiae*,81]. | *rbc*L: *Plocamium* luculentum ([KX284727](https://www.ncbi.nlm.nih.gov/nuccore/KX284727); 99.8%)  *cox*1: *Plocamium* luculentum ([KJ398160](https://www.ncbi.nlm.nih.gov/nuccore/KJ398160); 99.6%) | The *rbc*L analyses suggested that these specimens form a clade with *P*. luculentum from Japan and South Korea with full statistical supports (S50A Fig. in S5 File). The *rbc*L sequences were 0.2–0.4% divergent from *P*. luculentum ([KX284727](https://www.ncbi.nlm.nih.gov/nuccore/KX284727), [LC821337](https://www.ncbi.nlm.nih.gov/nuccore/LC821337)). The *cox*1 analyses suggested that these specimens form a clade with *P*. luculentum from Japan and South Korea, with moderate statistical supports (75% BP and 0.99 PP; S50B Fig. in S5 File). The *cox*1 sequences were 0.4–1.7% divergent from *P*. luculentum ([KJ398160](https://www.ncbi.nlm.nih.gov/nuccore/KJ398160), [LC821075](https://www.ncbi.nlm.nih.gov/nuccore/LC821075)). | This specimen was identified as *P*. luculentum. The identification was supported by both morphological and genetic identification.  Id: C1 |
|  | 15 May 2022; R. Terada; TNS AL-222170  *rbc*L: [LC821339](https://www.ncbi.nlm.nih.gov/nuccore/LC821339)  *cox*1: [LC821077](https://www.ncbi.nlm.nih.gov/nuccore/LC821077) |  |  |  |  |
| *Plocamium ovicorne* Okamura (S18 Fig. in S4 File) | 15 May 2021; R. Terada; TNS AL-215771  *rbc*L: [LC821340](https://www.ncbi.nlm.nih.gov/nuccore/LC821340)  *cox*1: [LC821078](https://www.ncbi.nlm.nih.gov/nuccore/LC821078) | The presence of serrate branchlets of this specimen was in accordance with those of *P*. *ovicorne* recorded in Japan and South Korea [3,8,66,82]. | *rbc*L: *Plocamium* ovicorne ([LC821341](https://www.ncbi.nlm.nih.gov/nuccore/LC821341); 99.7%)  *cox*1: *Plocamium* ovicorne ([LC821079](https://www.ncbi.nlm.nih.gov/nuccore/LC821079); 98.3%) | The *rbc*L analyses suggested that this specimen forms a clade with *P*. *ovicorne* from Awaji Island, Japan, with full statistical supports (S50A Fig. in S5 File). The *rbc*L sequence was 0.3% divergent from Japanese collection assigned to this species ([LC821341](https://www.ncbi.nlm.nih.gov/nuccore/LC821341)). The *cox*1 analyses suggested that this specimen forms a clade with *P*. *ovicorne* from Japan with full statistical supports (S50B Fig. in S5 File). The *cox*1 sequence was 1.7% divergent from Japanese collection assigned to this species ([LC821079](https://www.ncbi.nlm.nih.gov/nuccore/LC821079)). | This specimen was identified as *P*. *ovicorne*. The identification was supported by both morphological and genetic identification.  Id: C1 |
| Plocamium sp. TNE (S18 Fig. in S4 File) | 25 June 2021; R. Terada; TNS AL-222095  *rbc*L: [LC821343](https://www.ncbi.nlm.nih.gov/nuccore/LC821343)  *cox*1: [LC821081](https://www.ncbi.nlm.nih.gov/nuccore/LC821081) | The upper branches produced alternating pairs of 2 ramuli of this specimen were in accordance with those of *P*. luculentum recorded in Japan and South Korea [3,8,66 as *P*. *telfairiae*,81,this study]. | *rbc*L: *Plocamium* sp. Asia ([MW770788](https://www.ncbi.nlm.nih.gov/nuccore/MW770788); 99.3%)  *cox*1: *Plocamium* sp. Asia ([JF271631](https://www.ncbi.nlm.nih.gov/nuccore/JF271631) as *P*. cf. *telfairiae*; 98.3%) | The *rbc*L analyses suggested that this specimen is distinct from *P*. *luculentum* and forms a clade with *P*. *fimbriatum* from Oman and *P*. sp. Asia from Japan and South Korea with full statistical supports (S50A Fig. in S5 File). The *rbc*L sequence was 0.7–1.6% divergent from the other three taxa ([MW770786](https://www.ncbi.nlm.nih.gov/nuccore/MW770786), [MW770788](https://www.ncbi.nlm.nih.gov/nuccore/MW770788), [LC821342](https://www.ncbi.nlm.nih.gov/nuccore/LC821342)). The *cox*1 analyses suggested that this specimen forms a clade with *P*. *fimbriatum* from Oman, *P*. *sandvicense* from Hawaii, U.S.A., *P*. sp. Asia from Japan, and *P*. cf. *telfairiae* from South Africa with moderate to high statistical supports (79% BP and 1.00 PP; S50B Fig. in S5 File). The *cox*1 sequence was 1.3–1.6% divergent from the other four taxa ([HQ422693](https://www.ncbi.nlm.nih.gov/nuccore/HQ422693), [JF271587](https://www.ncbi.nlm.nih.gov/nuccore/JF271587), [JF271631](https://www.ncbi.nlm.nih.gov/nuccore/JF271631), [LC821080](https://www.ncbi.nlm.nih.gov/nuccore/LC821080)). | We could not identify this specimen. Although the morphology of this species is similar to that of *P*. *luculentum*, the *rbc*L and *cox*1 sequences appeared to be distant from *P. luculentum* and is closely related to *P*. *fimbriatum* and *P*. sp. Asia.  Id: U3 |
| Sarcodia sp. JP1 (S18 Fig. in S4 File) | 16 May 2022; R. Terada; TNS AL-222148  *rbc*L: [LC821344](https://www.ncbi.nlm.nih.gov/nuccore/LC821344)  *cox*1: [LC821082](https://www.ncbi.nlm.nih.gov/nuccore/LC821082) | The habit and vegetative anatomy of this specimen were in accordance with those of *S*. ceylanica recorded in Japan [3,83 as *S*. *montagneana*]. | *rbc*L: Sarcodia sp. JP1 ([KX451172](https://www.ncbi.nlm.nih.gov/nuccore/KX451172); 99.2%)  *cox*1: *Sarcodia ciliata* ([HM915894](https://www.ncbi.nlm.nih.gov/nuccore/HM915894); 94.8%) | The *rbc*L analyses suggested that this specimen forms a clade with *S*. sp. JP1 from Chiba, Japan, with full statistical supports (S50A Fig. in S5 File). The *rbc*L sequence was 0.8% divergent from *S*. sp. JP1 ([KX451172](https://www.ncbi.nlm.nih.gov/nuccore/KX451172)). The *cox*1 sequence lacked a close match to data in INSD and was distant from *S*. *ciliata* and *S*. sp. HEC15899 ([HM915894](https://www.ncbi.nlm.nih.gov/nuccore/HM915894), [HQ956910](https://www.ncbi.nlm.nih.gov/nuccore/HQ956910.1?report=genbank); S50B Fig. in S5 File). | We could not identify this specimen. This specimen has been identified as *S*. ceylanica. According to Rodríguez-Prieto et al. [84], “*S*. ceylanica” recorded in Japan is different from *S*. *ceylanica* from Sri Lanka and is divided into two lineages. Our specimen is corresponding to *S*. sp. JP1 in Rodríguez-Prieto et al. [84].  Id: U2 |
| Rhodymeniophycidae, Rhodymeniales, Champiaceae | | | | | |
| *Champia expansa* Yendo (S18 Fig. in S4 File) | 19 May 2016; R. Terada; TNS AL-209851  *rbc*L: [LC821345](https://www.ncbi.nlm.nih.gov/nuccore/LC821345)  *cox*1: [LC821085](https://www.ncbi.nlm.nih.gov/nuccore/LC821085) | The flattened blades of these specimens were in accordance with those of *C*. *expansa* recorded in Japan and South Korea [3,8,12,85,86]; however, this specimen was pinnately branched, whereas C. expansa branched dichotomously or subdichotomously [3,8,12,85,86]. | *rbc*L: *Champia expansa* ([AB693122](https://www.ncbi.nlm.nih.gov/nuccore/AB693122); 99.4%)  *cox*1: *Champia expansa* ([KF356129](https://www.ncbi.nlm.nih.gov/nuccore/KF356129); 98.0%) | The *rbc*L and *cox*1 analyses suggested that these specimens form a clade with *C*. expansa from Japan and South Korea with full statistical supports (S51 Fig. in S5 File). The *rbc*L sequence was 0.6–0.7% divergent from Japanese and Korean collections assigned to *C*. expansa ([KF356092](https://www.ncbi.nlm.nih.gov/nuccore/KF356092), [AB693122](https://www.ncbi.nlm.nih.gov/nuccore/AB693122)), whereas the *cox*1 sequence was 2.0–2.4% divergent from Japanese and Korean collections assigned to *C*. expansa ([KF356129](https://www.ncbi.nlm.nih.gov/nuccore/KF356129), [LC821084](https://www.ncbi.nlm.nih.gov/nuccore/LC821084)). | Although the branching pattern of our specimens differed from that of *C*. *expansa* were identified as *C*. *expansa* based on the low divergence of the *rbc*L and *cox*1 sequences among the specimens assigned to *C*. *expansa*.  Id: C3 |
|  | 18 May 2017; R. Terada; TNS AL-209394  *rbc*L: [LC821346](https://www.ncbi.nlm.nih.gov/nuccore/LC821346) |  |  |  |  |
| *Champia* sp.1 TNE (S18 Fig. in S4 File) | 18 May 2016; R. Terada; TNS AL-220739  *rbc*L: [LC821349](https://www.ncbi.nlm.nih.gov/nuccore/LC821349)  *cox*1: [LC821097](https://www.ncbi.nlm.nih.gov/nuccore/LC821097) | The cylindrical thallus without curbed branches or hooked branch tips of this specimen was similar to those of *C*. *recta* recorded in Japan and South Korea [3 as *C*. *parvula*,8,12,86]; however, the branches of it was narrower than that of *C*. *recta*. | *rbc*L: *Champia recta* ([AB693109](https://www.ncbi.nlm.nih.gov/nuccore/AB693109); 98.4%)  *cox*1: *Champia recta* ([KF356110](https://www.ncbi.nlm.nih.gov/nuccore/KF356110); 95.6%) | The *rbc*L analyses suggested that this specimen forms a clade with *C*. *inkyui* and *C*. recta from Japan and South Korea with full statistical supports (S51A Fig. in S5 File). The *rbc*L sequence was 1.6–2.0% divergent from the other two taxa ([AB693109](https://www.ncbi.nlm.nih.gov/nuccore/AB693109)–[AB693116](https://www.ncbi.nlm.nih.gov/nuccore/AB693116), [KF356064](https://www.ncbi.nlm.nih.gov/nuccore/KF356064), [KF356065](https://www.ncbi.nlm.nih.gov/nuccore/KF356065), [KF356076](https://www.ncbi.nlm.nih.gov/nuccore/KF356076), [KF356078](https://www.ncbi.nlm.nih.gov/nuccore/KF356078)). The *cox*1 analyses suggested that this specimen forms a clade with *C*. *inkyui* and *C*. recta from Japan and South Korea, and four taxa of *C*. spp. from Hawaii, U.S.A. with moderate to high statistical supports (89% BP and 0.98 PP; S51B Fig. in S5 File). The *cox*1 sequence is 4.4–6.4% divergent from the other six taxa ([HQ422763](https://www.ncbi.nlm.nih.gov/nuccore/HQ422763), [HQ422822](https://www.ncbi.nlm.nih.gov/nuccore/HQ422822), [HQ422864](https://www.ncbi.nlm.nih.gov/nuccore/HQ422864), [KF356105](https://www.ncbi.nlm.nih.gov/nuccore/KF356105), [KF356110](https://www.ncbi.nlm.nih.gov/nuccore/KF356110)–[KF356112](https://www.ncbi.nlm.nih.gov/nuccore/KF356112), [OM460694](https://www.ncbi.nlm.nih.gov/nuccore/OM460694), [LC821086](https://www.ncbi.nlm.nih.gov/nuccore/LC821086)– [LC821090](https://www.ncbi.nlm.nih.gov/nuccore/LC821090), [LC821093](https://www.ncbi.nlm.nih.gov/nuccore/LC821093)– [LC821095](https://www.ncbi.nlm.nih.gov/nuccore/LC821095)). | We could not identify this specimen. Although the morphology of this species is similar to that of *C*. *recta*, the *rbc*L and *cox*1 sequences appeared to be distant from *C. recta* and the other *Champia* species.  Id: U2 |
| *Champia* sp.2 TNE (S20 Fig. in S4 File) | 19 May 2016; R. Terada; TNS AL-220743  *rbc*L: [LC821350](https://www.ncbi.nlm.nih.gov/nuccore/LC821350)  *cox*1: [LC821098](https://www.ncbi.nlm.nih.gov/nuccore/LC821098) | The flattened and irregularly branched thallus of this specimen were similar to those of *C*. *somalensis* recorded in India [87]. | *rbc*L: *Champia vieillardii* ([EU670596](https://www.ncbi.nlm.nih.gov/nuccore/EU670596); 93.4%)  *cox*1: *Champia* sp.4 Cocos ([MH308214](https://www.ncbi.nlm.nih.gov/nuccore/MH308214); 88.9%) | The *rbc*L and *cox*1 sequence did not closely match the INSD data and were distant from variou other *Champia* species (S51 Fig. in S5 File). | Although this specimen was morphologically similar to *C*. *somalensis*, we could not identify it as *C*. *somalensis* because of the lack of available sequence data for *C*. *somalensis* in the INSD, and no records of this species have been found in the Pacific.  Id: U3. |
| *Champia* sp.3 TNE (*C*. cf. *vieillardii*; S20 Fig. in S4 File) | 18 May 2016; R. Terada; TNS AL-220745  *rbc*L: [LC821351](https://www.ncbi.nlm.nih.gov/nuccore/LC821351)  *cox*1: [LC821099](https://www.ncbi.nlm.nih.gov/nuccore/LC821099) | The flattened and pinnately branched thallus of this specimen were most similar to those of *C*. *vieillardii* recorded in Hawaii and Malaysia [5,88]. | *rbc*L: *Champia vieillardii* ([FJ212299](https://www.ncbi.nlm.nih.gov/nuccore/FJ212299); 93.2%)  *cox*1: *Champia* sp.4 Cocos ([MH308214](https://www.ncbi.nlm.nih.gov/nuccore/MH308214); 87.9%) | The *rbc*L sequence did not closely match the INSD data and was distant from various *Champia* species, including *C*. *vieillardii* from Puerto Rico ([EU670596](https://www.ncbi.nlm.nih.gov/nuccore/EU670596), [FJ212299](https://www.ncbi.nlm.nih.gov/nuccore/FJ212299); S51A Fig. in S5 File). The *cox*1 sequences did not closely match the INSD data and was distant from various *Champia* species, including *C*. *vieillardii* from Hawaii, U.S.A. ([HQ422943](https://www.ncbi.nlm.nih.gov/nuccore/HQ422943), [HQ422762](https://www.ncbi.nlm.nih.gov/nuccore/HQ422762)) (S51B Fig. in S5 File). | We tentatively identified this specimen as *C*. cf. *vieillardii*. Morphologically, the specimen was identified as *C*. *vieillardii*. However, *rbc*L and *cox*1 analyses revealed that *C*. *vieillardii* recorded in Japan, Hawaii, and Puerto Rico include two to three cryptic species. Further morphological and molecular analyses, including the type specimen of *C*. *vieillardii* or samples from a type locality (New Caledonia) are needed to clarify its identification.  Id: T |
| *Champia* sp.4 TNE (S20 Fig. in S4 File) | 18 May 2017; R. Terada; TNS AL-220746  *rbc*L: [LC821352](https://www.ncbi.nlm.nih.gov/nuccore/LC821352)  *cox*1: [LC821100](https://www.ncbi.nlm.nih.gov/nuccore/LC821100) | This specimen was morphologically similar to *Champia* sp.2 TNE collected from offshore Tanegashima Island [this study]. | *rbc*L: *Champia vieillardii* ([FJ212299](https://www.ncbi.nlm.nih.gov/nuccore/FJ212299); 96.6%)  *cox*1: *Champia* sp.4 Cocos ([MH308214](https://www.ncbi.nlm.nih.gov/nuccore/MH308214); 90.6%) | The *rbc*L analyses suggested that this specimen forms a clade with *C*. *vieillardii* from Puerto Rico with moderate to high statistical supports (70% BP and 1.00 PP; S51A Fig. in S5 File). The *rbc*L sequence was 3.4–3.5% divergent from the other two taxa ([EU670596](https://www.ncbi.nlm.nih.gov/nuccore/EU670596), [FJ212299](https://www.ncbi.nlm.nih.gov/nuccore/FJ212299)). The *cox*1 sequences did not closely match the INSD data and was distant from various other *Champia* species (S51B Fig. in S5 File). | We could not identify this specimen. Morphological characteristics of this specimen is similar to those of *C*. sp.2 TNE; however, molecular analyses indicated that it is distinct from *C*. sp.2 TNE.  Id: U3 |
| *Champia* sp.5 TNE (S20 Fig. in S4 File) | 16 May 2022; R. Terada; TNS AL-222151  *rbc*L: [LC821353](https://www.ncbi.nlm.nih.gov/nuccore/LC821353)  *cox*1: [LC821101](https://www.ncbi.nlm.nih.gov/nuccore/LC821101) | The entangled, flattened, linear, and irregularly branched thallus of this specimen did not match any *Champia* species recorded in the Pacific [3,5,7,8,12,89]. | *rbc*L: *Champia* sp.5 Cocos ([MH308217](https://www.ncbi.nlm.nih.gov/nuccore/MH308217); 96.6%)  *cox*1: *Champia* sp.4 Cocos ([MH308214](https://www.ncbi.nlm.nih.gov/nuccore/MH308214); 89.5%) | The *rbc*L analyses suggested that this specimen forms a clade with *C*. sp. 5 Cocos from Cocos Island, Australia, with high statistical supports (86% BP and 1.00 PP; S51A Fig. in S5 File). The *rbc*L sequence was 3.4% divergent from *C*. sp. 5 Cocos ([MH308217](https://www.ncbi.nlm.nih.gov/nuccore/MH308217)). The *cox*1 sequences did not closely match the INSD data and was distant from various other *Champia* species (S51B Fig. in S5 File). | We could not identify this specimen because of the lack of similar morphological species recorded in the Pacific and close sequence data in INSD.  Id: U1 |
| Rhodymeniophycidae, Rhodymeniales, Faucheaceae | | | | | |
| *Gloiocladia* sp.1 TNE (S20 Fig. in S4 File) | 4 June 2022; R. Terada; TNS AL-222213  *rbc*L: [LC821354](https://www.ncbi.nlm.nih.gov/nuccore/LC821354)  *cox*1: [LC821102](https://www.ncbi.nlm.nih.gov/nuccore/LC821102) | The flattened, subdichotomously or irregularly branched thallus with marginal proliferations of this specimen was similar to that of *G*. *polycarpa* recorded in Australia [89]; however, its height was much smaller than that of *G*. *polycarpa*. | *rbc*L: *Gloiocladia spinulosa* ([KF547023](https://www.ncbi.nlm.nih.gov/nuccore/KF547023); 98.8%)  *cox*1: *Gloiocladia spinulosa* ([KF547031](https://www.ncbi.nlm.nih.gov/nuccore/KF547031); 95.5%) | The combined *rbc*L and *cox*1 analyses suggested that this specimen forms a clade with *G*. *spinulosa* from South Korea with full statistical supports (S52C Fig. in S5 File). The *rbc*L sequence was 1.2% divergent from Korean *G*. *spinulosa* ([KF547023](https://www.ncbi.nlm.nih.gov/nuccore/KF547023)), whereas the *cox*1 sequence was 4.5% divergent from Korean *G*. *spinulosa* ([KF547031](https://www.ncbi.nlm.nih.gov/nuccore/KF547031)). | Although this specimen was morphologically similar to *G*. *polycarpa*, we could not identify it as *G*. *polycarpa* because of the lack of available sequence data for *G*. *polycarpa* in the INSD, and no records of this species have been found in the northern Pacific.  Id: U2. |
| *Gloiocladia* sp.2 TNE (S20 Fig. in S4 File) | 16 May 2021; R. Terada; TNS AL-222066  *rbc*L: [LC821355](https://www.ncbi.nlm.nih.gov/nuccore/LC821355)  *cox*1: [LC821103](https://www.ncbi.nlm.nih.gov/nuccore/LC821103) | The linear and pinnately branched thallus of this specimen is similar to those of *G*. *iyosensis* recorded in Japan, South Korea, Hawaii, and Australia [3,5,7,12,55]. | *rbc*L: *Gloiocladia* sp. GiyoGF2005 ([OR881954](https://www.ncbi.nlm.nih.gov/nuccore/OR881954) as *G*. *iyoensis*; 99.3%)  *cox*1: *Gloiocladia* sp. ARS 10720 [OR881946](https://www.ncbi.nlm.nih.gov/nuccore/OR881946) as *G*. *iyoensis*; 97.7%) | The *rbc*L and *cox*1 analyses suggested that this specimen is distant from *G*. *iyoensis* collected from Uwajima, Ehime Prefecture, Japan, near its type locality (S52C Fig. in S5 File). The *rbc*L analyses suggested that this specimen forms a clade with *G*. sp. GiyoGF2005 (as *G*. *iyoensis*) from Australia and *G*. sp. ARS 10720 from Hawaii, U.S.A. with high statistical supports (96% BP and 1.00 PP; S52A Fig. in S5 File). The *rbc*L sequence was 0.7–0.9% divergent from the other two taxa ([FJ713142](https://www.ncbi.nlm.nih.gov/nuccore/FJ713142), [OR881954](https://www.ncbi.nlm.nih.gov/nuccore/OR881954)). The *cox*1 analyses suggested that this specimen forms a clade with *G*. sp. ARS 10720 (as *G*. *iyoensis*) from Hawaii, U.S.A. with high statistical supports (97% BP and 0.99 PP; S52B Fig. in S6 File). The *cox*L sequence was 2.3% divergent from *G*. sp. ARS 10720 ([OR881946](https://www.ncbi.nlm.nih.gov/nuccore/OR881946)). | We could not identify this specimen. Although the morphology of this species is similar to that of *G*. *iyosensis*, the *rbc*L and *cox*1 sequences appeared to be distant from *G*. *iyosensis* and the other *Gloiocladia* species. Molecular analyses indicated that this specimen is closely related to *Gloiocladia* spp. labeled “*G*. *iyoensis*” from Hawaii and Australia; however, both *rbc*L and *cox*1 analyses indicated that these sequences are distinct from *G*. *iyoensis*.  Id: U2 |
| Rhodymeniophycidae, Rhodymeniales, Rhodymeniaceae | | | | | |
| Lomentariaceae sp. TNE (S20 Fig. in S4 File) | 18 May 2016; R. Terada; TNS AL-220748  *rbc*L: [LC821362](https://www.ncbi.nlm.nih.gov/nuccore/LC821362)  *cox*1: [LC821112](https://www.ncbi.nlm.nih.gov/nuccore/LC821112) | This specimen was morphologically similar to lomentariacean species with cylindrical thalli, such as *Yendoa hakodatensis*, which were recorded in Japan, South Korea, and Hawaii [3,5,12 as *Lomentaria hakodatensis*]. However, the branching pattern of this specimen is alternate, whereas that of *Y*. *hakodatensis* is usually the opposite. | *rbc*L: *Fushitsunagia catenata* ([LC821359](https://www.ncbi.nlm.nih.gov/nuccore/LC821359); 95.0%)  *cox*1: *Ceratodictyon variabile* ([OK641550](https://www.ncbi.nlm.nih.gov/nuccore/OK641550); 91.0%) | The *rbc*L and *cox*1 sequences did not closely match the INSD data and were distant from various other lomentariacean genera (S51 Fig. in S5 File). | We could not identify this specimen because of the lack of similar morphological species recorded in the Pacific and close sequence data in INSD.  Id: U1 |
| Rhodymeniophycidae, Rhodymeniales, Rhodymeniaceae | | | | | |
| *Botryocladia* *leptopoda* (J.Agardh) Kylin (S20 Fig. in S4 File) | 20 July 2017; R. Terada; TNS AL-209858  *rbc*L: [LC821364](https://www.ncbi.nlm.nih.gov/nuccore/LC821364)  *cox*1: [LC821115](https://www.ncbi.nlm.nih.gov/nuccore/LC821115) | The large thallus with numerous vesicles of this specimen was in accordance with that of *B*. *leptopoda* recorded in Japan and Australia [3,7,90 as *Chrysymenia uvaria*]. | *rbc*L: *Botryocladia leptopoda* ([KU726723](https://www.ncbi.nlm.nih.gov/nuccore/KU726723); 99.3%)  *cox*1: *Botryocladia leptopoda* ([MT876667](https://www.ncbi.nlm.nih.gov/nuccore/MT876667); 98.7%) | The *rbc*L analyses suggested that this specimen forms a clade with *B*. *leptopoda* from Australia and Philippines, with full statistical supports (S54A Fig. in S5 File). The *rbc*L sequence was 0.7–1.7% divergent from the other two taxa ([HQ400597](https://www.ncbi.nlm.nih.gov/nuccore/HQ400597), [KU726723](https://www.ncbi.nlm.nih.gov/nuccore/KU726723)). The *cox*1 analyses suggested that this specimen forms a clade with *B*. *leptopoda* from India, Norfolk Island, Australia, and Philippines, with moderate statistical supports (73% BP and 0.99 PP; S54B Fig. in S5 File). The *cox*1 sequence was 1.3–1.9% divergent from the other three taxa ([HM918826](https://www.ncbi.nlm.nih.gov/nuccore/HM918826.1?report=genbank), [KU873094](https://www.ncbi.nlm.nih.gov/nuccore/KU873094), [MT876667](https://www.ncbi.nlm.nih.gov/nuccore/MT876667)). | This specimen was identified as *B*. *leptopoda*. The identification was supported by both morphological and genetic identification.  Id: C1 |
| *Botryocladia* sp. TNE (*B*. cf. *kuckuckii*; S20 Fig. in S4 File) | 3 October 2021; R. Terada; TNS AL-222137  *rbc*L: [LC821365](https://www.ncbi.nlm.nih.gov/nuccore/LC821365)  *cox*1: [LC821116](https://www.ncbi.nlm.nih.gov/nuccore/LC821116) | The small thallus with four vesicles, and two vesicles produced on each branch of this specimen was similar to that of *B*. *kuckuckii* recorded in Japan and Indonesia [3 as *B*. *skottsbergii*,91]. | *rbc*L: *Botryocladia iridescens* ([GQ146435](https://www.ncbi.nlm.nih.gov/nuccore/GQ146435); 93.8%)  *cox*1: *Botryocladia sonderi* ([KU873093](https://www.ncbi.nlm.nih.gov/nuccore/KU873093); 90.3%) | The *rbc*L and *cox*1 sequences did not closely match the INSD data and were distant from variou other *Botryocladia* species, including *B*. cf. *kuckuckii* from Taiwan (S54 Fig. in S5 File). | We tentatively identified this specimen as *B*. cf. *kuckuckii*. Morphologically, this specimen was identified as *B*. *kuckuckii*. However, *rbc*L and *cox*1 analyses revealed that *B*. *kuckuckii* recorded in Japan and Taiwan include two cryptic species. We could not determine which corresponds to true *B*. *kuckuckii*, and treated them as *B*. sp. TNE (*B*. cf. *kuckuckii*) and *B*. sp. TW (*B*. cf. *kuckuckii*). Further morphological and molecular analyses, including the type specimen of *B*. *kuckuckii* or samples from a type locality (Indonesia) are needed to clarify its identification.  Id: T |
| "*Chamaebotrys*" *lomentariae* (Tak.Tanaka & K.Nozawa) Huisman (S20 Fig. in S4 File)^b^ | 26 May 2015; R. Terada; TNS AL-200139  *rbc*L: [LC821367](https://www.ncbi.nlm.nih.gov/nuccore/LC821367)  *cox*1: [LC821118](https://www.ncbi.nlm.nih.gov/nuccore/LC821118) | The habit and vegetative structure of this specimen were in accordance with descriptions of *C*. *lomentariae* recorded on offshore of Tanegashima Island, Japan [3,65 as *Coelarthrum lomentariae*,92]. | *rbc*L: *Halopeltis tanakae* ([LC605128](https://www.ncbi.nlm.nih.gov/nuccore/LC605128); 94.5%)  *cox*1: *Halopeltis willisii* ([HM915686](https://www.ncbi.nlm.nih.gov/nuccore/HM915686); 90.1%) | The *rbc*L and *cox*1 sequences did not closely match the INSD data and suggested that *C*. *lomentariae* is included in the *Halopeltis* clade (S54 Fig. in S5 File). | Although the *rbc*L and *cox*1 sequences did not closely match the INSD data, we identified this specimen as *C*. *lomentariae* morphologically in accordance with the *C*. *lomentariae* was described in the offshore Tanegashima Islands. This species is currently classified as *Chamaebotrys* [3,93]; however, molecular analyses revealed that it is related to the *Halopeltis*.  Id: C2 |
|  | 18 May 2017; R. Terada; TNS AL-209259  *rbc*L: [LC821368](https://www.ncbi.nlm.nih.gov/nuccore/LC821368)  *cox*1: [LC821119](https://www.ncbi.nlm.nih.gov/nuccore/LC821119) |  |  |  |  |
|  | 3 October 2021; R. Terada; TNS AL-222103  *rbc*L: [LC821369](https://www.ncbi.nlm.nih.gov/nuccore/LC821369)  *cox*1: [LC821120](https://www.ncbi.nlm.nih.gov/nuccore/LC821120) |  |  |  |  |
| *Chamaebotrys* sp.1 TNE (*C.* cf. *boergesenii*; S21 Fig. in S4 File) | 26 May 2015; R. Terada; TNS AL-209864  *rbc*L: [LC821370](https://www.ncbi.nlm.nih.gov/nuccore/LC821370)  *cox*1: [LC821121](https://www.ncbi.nlm.nih.gov/nuccore/LC821121) | The decumbent, regularly segmented thallus of this specimen was similar to those of *C*. *boergesenii* recorded in Japan, Hawaii, and Australia [3,5,7,93] | *rbc*L: *Chamaebotrys boergesenii* ([KU726729](https://www.ncbi.nlm.nih.gov/nuccore/KU726729); 92.0%)  *cox*1: *Halichrysis concrescens* ([JQ907536](https://www.ncbi.nlm.nih.gov/nuccore/JQ907536); 88.6%) | The combined *rbc*L and *cox*1 analyses suggested that this specimen forms a clade with *C*. *boergesenii* from Western Australia, Australia with full statistical supports (S54C Fig. in S5 File). The *rbc*L sequence was 8.0% divergent from *C*. *boergesenii* from Australia ([KU726729](https://www.ncbi.nlm.nih.gov/nuccore/KU726729)), whereas the *cox*1 sequence was 11.8% divergent from *C*. *boergesenii* from Australia ([KU707876](https://www.ncbi.nlm.nih.gov/nuccore/KU707876)). | We tentatively identified this specimen as *C.* cf. *boergesenii*. Morphologically, this specimen was identified as *C.* *boergesenii*. However, molecular analyses revealed that it was distinct from Australian *C.* *boergesenii* and *C*. sp.2 (*C.* cf. *boergesenii*) from offshore Tanegashima Island, suggesting that *C.* *boergesenii* recorded in the Pacific includes two to three cryptic species. Further morpho-anatomical and molecular analyses, including the type specimens of *C*. *boergesenii* or samples from a type locality (Indonesia) are needed to clarify its identification.  Id: T |
| *Chamaebotrys* sp.2 TNE (*C.* cf. *boergesenii*; S21 Fig. in S4 File) | 19 May 2016; R. Terada; TNS AL-209865  *rbc*L: [LC821371](https://www.ncbi.nlm.nih.gov/nuccore/LC821371)  *cox*1: [LC821122](https://www.ncbi.nlm.nih.gov/nuccore/LC821122) | The habit and vegetative anatomy of this specimen were similar to those of *C*. *boergesenii* and *C*. sp.1TNE [3,5,7,93,this study]; however, it is much more branched than *C*. sp.1 JP. | *rbc*L: “*Botryocladia*” sp. 1 SV-2018 ([LT969687](https://www.ncbi.nlm.nih.gov/nuccore/LT969687); 97.1%)  *cox*1: *Chamaebotrys boergesenii* ([HQ423017](https://www.ncbi.nlm.nih.gov/nuccore/HQ423017); 92.2%) | The *rbc*L sequence did not closely match the INSD data and was distant from various other rhodymeniacean genera (S54A Fig. in S5 File). The *cox*1 analyses suggested that this specimen forms a clade with *C*. *boergesenii* from Hawaii, U.S.A. with low to moderate statistical supports (63% BP and 0.99 PP; S54B Fig. in S5 File). The *cox*1 sequence was 7.8% divergent from *C*. *boergesenii* from Hawaii, U.S.A. ([HQ423017](https://www.ncbi.nlm.nih.gov/nuccore/HQ423017)). | We tentatively identified this specimen as *C.* cf. *boergesenii*, as well as *C*. sp.1 TNE. Morphologically, both this specimen and *C*. sp.1 TNE were identified as *C*. *boergesenii*. However, *rbc*L and *cox*1 analyses indicated that this specimen was distinct from *C*. sp.1 TNE and Australian *C*. *boergesenii*, suggesting that *C*. *boergesenii* includes two to three cryptic species. We could not determine which corresponds to true *C*. *boergesenii*, and they were treated as *C*. sp.1 TNE (*C.* cf. *boergesenii*) and *C*. sp.2 TNE (*C.* cf. *boergesenii*). Further morpho-anatomical and molecular analyses, including the type specimens of *C*. *boergesenii* or samples from a type locality (Indonesia) are needed to clarify its identification.  Id: T1 |
| *Chrysymenia* sp. TNE (S21 Fig. in S4 File) | 26 May 2018; R. Terada & M. Suzuki; TNS AL-213811  *rbc*L: [LC821372](https://www.ncbi.nlm.nih.gov/nuccore/LC821372)  *cox*1: [LC821123](https://www.ncbi.nlm.nih.gov/nuccore/LC821123) | The saccate thallus of this specimen was similar to that of C. grandis recorded in Japan and South Korea [3,8,12,94]; however, it differed from *C*. *grandis* by producing many marginal proliferations. | *rbc*L: *Chrysymenia grandis* ([KF547019](https://www.ncbi.nlm.nih.gov/nuccore/KF547019); 93.7%)  *cox*1: *Chrysymenia grandis* ([KF547027](https://www.ncbi.nlm.nih.gov/nuccore/KF547027); 91.1%) | The combined *rbc*L and *cox*1 analyses suggested that this specimen forms a clade with *C*. *grandis* from South Korea with high statistical supports (97% BP and 1.00 PP; S54C Fig. in S5 File). The *rbc*L sequence was 6.3% divergent from *C*. *grandis* ([KF547019](https://www.ncbi.nlm.nih.gov/nuccore/KF547019)), whereas the *cox*1 sequence was 8.9% divergent from *C*. *grandis* ([KF547027](https://www.ncbi.nlm.nih.gov/nuccore/KF547027)). | We could not identify this specimen because of the lack of similar morphological species recorded in the Pacific and close sequence data in INSD.  Id: U1 |
| *Drouetia* sp. TNE (S21 Fig. in S4 File) | 5 June 2022; R. Terada; TNS AL-222162  *rbc*L: [LC821373](https://www.ncbi.nlm.nih.gov/nuccore/LC821373)  *cox*1: [LC821124](https://www.ncbi.nlm.nih.gov/nuccore/LC821124) | The short stipe and anastomosing thallus of this specimen were similar to those of *D*. *viridescens* [95]. | *rbc*L: *Drouetia*　*viridescens* ([KU726732](https://www.ncbi.nlm.nih.gov/nuccore/KU726732); 91.4%)  *cox*1: *Drouetia scutellata* ([KU707846](https://www.ncbi.nlm.nih.gov/nuccore/KU707846); 92.0%) | The *rbc*L analyses suggested that this specimen forms a clade with *D*. *scutellata* and *D*. *viridescens* from New South Wales, Australia, with full statistical supports (S54A Fig. in S5 File). The *rbc*L sequence was 8.6–8.8% divergent from the other two taxa ([KU726698](https://www.ncbi.nlm.nih.gov/nuccore/KU726698), [KU726732](https://www.ncbi.nlm.nih.gov/nuccore/KU726732)). The *cox*1 analyses suggested that this specimen forms a clade with *D*. *aggregata*, *D*. *scutellata*, and *D*. *viridescens* from New South Wales, Australia, with high statistical supports (96% BP and 1.00 PP; S54B Fig. in S5 File). The *cox*1 sequence was 8.0–9.2% divergent from the other three taxa ([KU707873](https://www.ncbi.nlm.nih.gov/nuccore/KU707873), [KU707846](https://www.ncbi.nlm.nih.gov/nuccore/KU707846), [KU707881](https://www.ncbi.nlm.nih.gov/nuccore/KU707881)). | We could not identify this specimen. Although the morphology of this species is similar to that of *D*. *viridescens*, the *rbc*L and *cox*1 sequences appeared to be distant from *D*. *viridescens* and the other *Drouetia* species. This specimen was the first *Drouetia* species reported from the northwestern Pacific [29].  Id: U2 |
| *Halichrysis* sp. TNE (S21 Fig. in S4 File) | 5 June 2022; R. Terada; TNS AL-222164  *rbc*L: [LC821374](https://www.ncbi.nlm.nih.gov/nuccore/LC821374)  *cox*1: [LC821125](https://www.ncbi.nlm.nih.gov/nuccore/LC821125) | The habit and vegetative anatomy of this specimen were similar to those of *H*. *concrescens* and *H*. *micans* recorded in Australia [96]. | *rbc*L: *Halichrysis concrescens* ([JQ907551](https://www.ncbi.nlm.nih.gov/nuccore/JQ907551); 97.3%)  *cox*1: *Halichrysis concrescens* ([JQ907536](https://www.ncbi.nlm.nih.gov/nuccore/JQ907536); 93.4%) | The combined *rbc*L and *cox*1 analyses suggested that this specimen forms a clade with *H*. *concrescens* from Lord Howe Island, Australia, with high statistical supports (99% BP and 1.00 PP; S54C Fig. in S5 File). The *rbc*L sequence was 2.7% divergent from *H*. *concrescens* ([JQ907551](https://www.ncbi.nlm.nih.gov/nuccore/JQ907551)), whereas the *cox*1 sequence was 6.6% divergent from *H*. *concrescens* ([JQ907536](https://www.ncbi.nlm.nih.gov/nuccore/JQ907536)). | We could not identify this specimen. Although the morphology of this species is similar to that of *H*. *concrescens* and *H*. *micans*, the *rbc*L and *cox*1 sequences appeared to be distant from *H*. *concrescens* and *H*. *micans* and the other *Halichrysis* species.  Id: U3 |
| *Halopeltis tanakae* Mas.Suzuki & R.Terada (S21 Fig. in S4 File) | 18 May 2016; R. Terada; TNS AL-209873  *rbc*L: [LC605128](https://www.ncbi.nlm.nih.gov/nuccore/LC605128)  *cox*1: [LC605134](https://www.ncbi.nlm.nih.gov/nuccore/LC605134) | The habit and vegetative structure of this specimen were in accordance with descriptions of *H*. *tanakae* recorded on offshore of Tanegashima Island, Japan [3,18 as *Rhodymenia prostrata*,97]. | *rbc*L: *Halopeltis tanakae* ([LC605128](https://www.ncbi.nlm.nih.gov/nuccore/LC605128); 100%)  *cox*1: *Halopeltis tanakae* ([LC605134](https://www.ncbi.nlm.nih.gov/nuccore/LC605134); 100%) | The combined *rbc*L and *cox*1 analyses suggested that these specimens form a clade with *H*. *adnata* from South Korea and *H*. *nuahilihilia* from Hawaii, U.S.A. with full statistical supports (S54C Fig. in S5 File). The *rbc*L sequence was 0.6–1.1% divergent from the other two taxa ([JQ907552](https://www.ncbi.nlm.nih.gov/nuccore/JQ907552), [ON464714](https://www.ncbi.nlm.nih.gov/nuccore/ON464714)), whereas the *cox*1 sequence was 2.4–2.9% divergent from the other two taxa ([JQ907538](https://www.ncbi.nlm.nih.gov/nuccore/JQ907538), [ON464701](https://www.ncbi.nlm.nih.gov/nuccore/ON464701)). | This specimen was identified as *H*. *tanakae*. The identification was supported by both morphological and genetic identification.  Id: C1 |
|  | 20 July 2017; R. Terada; TNS AL-209875  *rbc*L: [LC605129](https://www.ncbi.nlm.nih.gov/nuccore/LC605129)  *cox*1: [LC605135](https://www.ncbi.nlm.nih.gov/nuccore/LC605135) |  |  |  |  |
|  | 3 October 2021; R. Terada; TNS AL-222105  *rbc*L: [LC821375](https://www.ncbi.nlm.nih.gov/nuccore/LC821375)  *cox*1: [LC821126](https://www.ncbi.nlm.nih.gov/nuccore/LC821126) |  |  |  |  |
| *Halopeltis* sp.1 TNE (S21 Fig. in S4 File) | 16 May 2022; R. Terada; TNS AL-222194  *rbc*L: [LC821376](https://www.ncbi.nlm.nih.gov/nuccore/LC821376)  *cox*1: [LC821127](https://www.ncbi.nlm.nih.gov/nuccore/LC821127) | The habit and vegetative anatomy of this specimen were similar to those of *H*. *tanakae* recorded in Japan [3,18 as *Rhodymenia prostrata*,97,this study]. This specimen adhared to the substratum as well as *H*. *tanakae*; however, the blades were free and loosened compared to those of *H*. *tanakae*. | *rbc*L: *Halopeltis tanakae* ([LC605128](https://www.ncbi.nlm.nih.gov/nuccore/LC605128); 94.0%)  *cox*1: *Halopeltis* sp. WELT A34186 ([MZ855285](https://www.ncbi.nlm.nih.gov/nuccore/MZ855285); 92.6%) | The *rbc*L and *cox*1 sequences did not closely match the INSD data and were distant from various other *Halopeltis* species (S54 Fig. in S5 File). | We could not identify this specimen. Although the morphology of this species is similar to that of *H*. *tanakae*, the *rbc*L and *cox*1 sequences appeared to be distant from *H*. *tanakae* and the other *Halopeltis* species.  Id: U2 |
| *Halopeltis* sp.2 TNE (*H*. cf. *adnata*; S21 Fig. in S4 File) | 16 May 2022; R. Terada; TNS AL-222193  *rbc*L: [LC821377](https://www.ncbi.nlm.nih.gov/nuccore/LC821377)  *cox*1: [LC821128](https://www.ncbi.nlm.nih.gov/nuccore/LC821128) | The membranous, partialy decumbent, and flabellate thallus of this specimen was similar to those of *H*. *adnata* recodied in Japan and South Korea [3 as *Rhodymenia adnata*,12 as *H*. cf. *adnata*,98 as *R*. *adnata*,99]. | *rbc*L: *Halopeltis tanakae* ([LC605128](https://www.ncbi.nlm.nih.gov/nuccore/LC605128); 97.5%)  *cox*1: *Halopeltis adnata* ([JQ907538](https://www.ncbi.nlm.nih.gov/nuccore/JQ907538); 93.7%) | The *rbc*L and *cox*1 sequences did not closely match the INSD data and were distant from various other *Halopeltis* species, including *H*. *adnata* from South Korea (S54 Figs in S5 File). | We tentatively identified this specimen as *H*. cf. *adnata*. Morphologically, this specimen was identified as *H*. *adnata*. However, molecular analyses revealed that it was distinct from Korean *H*. *adnata*, suggesting that *H*. *adnata* recorded in the Pacific includes two cryptic species. Further morpho-anatomical and molecular analyses, including the type specimens of *H*. *adnata* or samples from a type locality (Tateyama, Japan) are needed to clarify its identification.  Id: T |
| Rhodymeniophycidae, Sebdeniales, Sebdeniaceae | | | | | |
| *Sebdenia* sp. TNE (*S*. cf. *flabellata*; S22 Fig. in S4 File) | 15 May 2021; R. Terada; TNS AL-215817  *rbc*L: [LC821380](https://www.ncbi.nlm.nih.gov/nuccore/LC821380)  *cox*1: [LC821131](https://www.ncbi.nlm.nih.gov/nuccore/LC821131) | The habit and vegetative anatomy of this specimen were similar to those of *S*. *flabellata* recorded in Japan, South Korea [3,8,12] and *S*. *polydactyla* recorded in Australia [7]. | *rbc*L: *Sebdenia flabellata* ([KU726730](https://www.ncbi.nlm.nih.gov/nuccore/KU726730); 97.2%)  *cox*1: *Sebdenia flabellata* ([KF761650](https://www.ncbi.nlm.nih.gov/nuccore/KF761650); 95.4%) | The *rbc*L analyses suggested that this specimen forms a clade with *S*. *flabellata* from Bermuda with full statistical supports (S55A Fig. in S5 File). The *rbc*L sequence was 2.8% divergent from *S*. *flabellata* ([KU726730](https://www.ncbi.nlm.nih.gov/nuccore/KU726730)). The *cox*1 analyses suggested that this specimen forms a clade with *S*. *flabellata* from Bermuda, *S*. sp.1 flabellata from Lord Howe Island, Australia, and *S*. sp. from Mexico, with high statistical supports (94% BP and 1.00 PP; S55B Fig. in S5 File). The *cox*1 sequence was 4.6–5.4% divergent from the other four taxa ([JN641198](https://www.ncbi.nlm.nih.gov/nuccore/JN641198), [KF761649](https://www.ncbi.nlm.nih.gov/nuccore/KF761649), [KF761650](https://www.ncbi.nlm.nih.gov/nuccore/KF761650), [GRHOD446-10](https://v3.boldsystems.org/index.php/Public_RecordView?processid=GRHOD446-10)). | We tentatively identified this specimen as *S*. cf. *flabellata*. Morphologically, this specimen was identified as *S*. *flabellata*. However, molecular analyses revealed that it was distinct from *S*. *flabellata* from Bermuda and Australia, suggesting that *S*. *flabellata* recorded in the Pacific includes two to three cryptic species. In addition, Australian *S*. *flabellata* is currently treated as a misapplied name for *S*. *polydactyla* [7]. Further morpho-anatomical analyses, including the type specimens of *S*. *flabellata* and *S*. *polydactyla* or samples from their type localities (Guadeloupe; India) are needed to clarify its identification.  Id: T |
| Sebdeniaceae sp.1 TNE (S22 Fig. in S4 File) | 3 October 2021; R. Terada; TNS AL-222135  *rbc*L: [LC821381](https://www.ncbi.nlm.nih.gov/nuccore/LC821381)  *cox*1: [LC821132](https://www.ncbi.nlm.nih.gov/nuccore/LC821132) | The flattened and subdichotomouslly branched thallus of this specimen did not match any sebdeniacean species recorded in the Pacific [3,7,8,12]. | *rbc*L: Sebdeniaceae sp.1 GWS-2011 ([KU382048](https://www.ncbi.nlm.nih.gov/nuccore/KU382048); 99.8%)  *cox*1: Sebdeniaceae sp.1 GWS-2011 ([JN641193](https://www.ncbi.nlm.nih.gov/nuccore/JN641193); 98.9%) | The *rbc*L analyses suggested that this specimen forms a clade with Sebdeniaceae sp.1 GWS-2011 from Lord Howe Island, Australia, with full statistical supports (S55A Fig. in S5 File). The *rbc*L sequence was 0.2% divergent from Sebdeniaceae sp.1 GWS-2011 ([KU382048](https://www.ncbi.nlm.nih.gov/nuccore/KU382048)). The *cox*1 analyses suggested that this specimen forms a clade with Sebdeniaceae sp.1 GWS-2011 from Western Australia, Lord Howe Island, and Norfolk Island, Australia, with high statistical supports (93% BP and 1.00 PP; S55B Fig. in S5 File). The *cox*1 sequence was 1.1–2.6% divergent from the other three taxa ([HM916189](https://www.ncbi.nlm.nih.gov/nuccore/HM916189), [JN641193](https://www.ncbi.nlm.nih.gov/nuccore/JN641193), [JN641195](https://www.ncbi.nlm.nih.gov/nuccore/JN641195)). | We could not identify this specimen because of the lack of similar morphological species recorded in the Pacific and close sequence data in INSD. The *rbc*L and *cox*1 analyses indicated that it is conspecific with Sebdeniaceae sp.1 GWS-2011.  Id: U1 |
| Rhodymeniophycidae, Ordo incertae sedis, Calosiphoniaceae | | | | | |
| *Schmitzia* sp. TNE (S22 Fig. in S4 File) | 25 May 2018; R. Terada & M. Suzuki; TNS AL-220738  *rbc*L: [LC821382](https://www.ncbi.nlm.nih.gov/nuccore/LC821382) | The flattened and linear thallus of this specimen was similar to that of *S*. *japonica* recorded in Japan [3,100 as *Platoma japonicum*]; however, it is much smaller than *S*. *japonica*. | *rbc*L: *Schmitzia* sp.1 WA ([KU382067](https://www.ncbi.nlm.nih.gov/nuccore/KU382067); 98.1%) | The *rbc*L sequence did not closely match the INSD data and was distant from various other *Schmitzia* species (S56 Fig. in S5 File). | We could not identify this specimen because of the lack of key morphological characteristics and close sequence data in INSD. Further morphological and molecular analyses, including additional specimens and the type specimen of *S*. *japonica* or samples from a type locality (Enoshima, Japan)are needed to clarify its identification.  Id: U3 |

References

1. Tanaka T. Four new species of Galaxaura from Japan. Sci Pap Inst Algol Res Fac Sci Hokkaido Imp Univ. 1935; 1: 51–57.
2. Fontana S, Wang W-L, Tseng K-Y, Draisma SGA, Dumilag RV, Hu Z-M, et al. Seaweed diversification driven by Taiwan’s emergence and the Kuroshio Current: insights from the cryptic diversity and phylogeography of *Dichotomaria* (Galaxauraceae, Rhodophyta). Front Ecol Evol. 2024; 12:1346199. https://doi.org/10.3389/fevo.2024.1346199
3. Yoshida T. Marine algae of Japan. Tokyo: Uchida Rokakuho; 1998.
4. Hwang I-K, Kim H-S Algal flora of Korea. Volume 4, Number 2. Rhodophyta: Florideophyceae: Nemaliophycidae: Acrochaetiales, Colaconematales, Palmariales, Nemaliales. Nemalion red algae. Incheon: National Institute of Biological Resources; 2011.
5. Abbott IA Marine Red Algae of the Hawaiian Islands. Honolulu: Bishop Museum Press; 1999.
6. Womersley HBS. The Marine Benthic Flora of Southern Australia Part IIIA Bangiophyceae and Florideophyceae (Acrochaetiales, Nemaliales, Gelidiales, Hildenbrandiales and Gigartinales sensu lato). Canberra: Australian Biological Resources Study & the State Herbarium of South Australia; 1994.
7. Huisman JM. Algae of Australia. Marine Benthic Algae of North-western Australia, 2. Red Algae. Canberra & Melbourne: ABRS & CSIRO Publishing; 2018.
8. Kim MS, Kang JC, Kim B, Yang MY, Lee HW. Seaweed diversity on Udo Islet, Jeju. Jeju: Research Institute of Basic Sciences, Jeju University; 2022.
9. Wang W-L, Liu S-L, Lin S-M. First description of carposporophyte development in *Scinaia chinensis* (Galaxauraceae, Rhodophyta), a species newly recorded for Taiwan. Cryptogamie Algol. 2005; 26: 155–164.
10. Lee YP, Ko YD. The red algal genus *Scinaia* (Galaxauraceae, Nemaliales) on Jeju Island, Korea. Algae. 2006; 21: 267–281. https://doi.org/10.4490/algae.2006.21.3.267
11. Okamura K. Icones of Japanese algae. Vol. I No. 6. Tokyo: Published by the author; 1908.
12. Kim H-S. Algal flora of Korea. Volume 4, Number 8. Rhodophyta: Florideophyceae: Rhodymeniales, Bonnemaisoniales, Sebdeniales, Peyssonneliales. Marine Red Algae. Incheon: National Institute of Biological Resources; 2013.
13. Lee YP. Marine algae of Jeju. Seoul: Academy Publication; 2008.
14. Womersley HBS. The marine benthic flora of southern Australia Part IIIC Ceramiales- Ceramiaceae, Dasyaceae. Canberra & Adelaide: Australian Biological Resources Study & State Herbarium of South Australia; 1998.
15. Itono H. Studies on the ceramiaceous algae (Rhodophyta) from southern parts of Japan. Bibl Phycol. 1977; 35: 1–499.
16. Kim H-S. Algal flora of Korea. Volume 4, Number 6 Rhodophyta: Florideophyceae: Ceramiales: Ceramiaceae II (Corticated Species), Dasyaceae. Incheon: National Institute of Biological Resources; 2012.
17. Okamura K. Icones of Japanese algae. Vol. VI No. 5. Tokyo: Published by the author; 1931.
18. Tanaka T. Studies on some marine algae from southern Japan, VI. Mem Fac Fish, Kagoshima Univ. 1965; 14: 52–71.
19. Kim H-S, Lee IK. Algal flora of Korea. Volume 4, Number 5. Rhodophyta: Florideophyceae, Ceramiales: Ceramiaceae I (Non-corticate species). Marine red algae. Incheon: National Institute of Biological Resources; 2012.
20. Nam KW, Kang PJ. Algal flora of Korea. Volume 4, Number 7 Rhodophyta: Florideophyceae: Ceramiales: Delesseriaceae: 22 genera including *Acrosorium*. Marine Red Algae. Incheon: National Institute of Biological Resources; 2012.
21. Yamada Y. Notes on some Japanese algae, VIII. Sci Pap Inst Algol Res, Fac Sci, Hokkaido Imp Univ. 1938; 2: 119–130.
22. Okamura K. Icones of Japanese algae. Vol. VI No. 10. Tokyo: Published by the author; 1932.
23. Okamura K. Descriptions of Japanese algae. Tokyo: Uchida Rokakuho, Tokyo; 1936.
24. Womersley HBS. The marine benthic flora of southern Australia Part IIID Ceramiales- Delesseriaceae, Sarcomeniaceae, Rhodomelaceae. Canberra & Adelaide: Australian Biological Resources Study & State Herbarium of South Australia; 2003.
25. Sherwood AR, Lin S-M, Wade RM, Spalding HL, Smith CM, Kosaki RK. Characterization of *Martensia* (Delesseriaceae; Rhodophyta) from shallow and mesophotic habitats in the Hawaiian Islands: description of four new species. Euro J Phycol. 2019; 55: 172–185. https://doi.org/10.1080/09670262.2019.1668062
26. Lin S-M, Lewis JE, Fredericq S. *Drachiella liaoii* sp. nov., a new member of the Schizoserideae (Delesseriaceae, Rhodophyta) from Taiwan and the Philippines. Euro J Phycol. 2002; 37: 93-102. https://doi.org/10.1017/S0967026201003511
27. Yamada Y. Notes on some Japanese algae, I. J Fac Sci Hokkaido Imp Univ. 1930; 1: 27–36.
28. Okamura K. Icones of Japanese algae. Vol. VI No. 6. Tokyo: Published by the author; 1931.
29. Guiry MD, Guiry GM. AlgaeBase. World-wide electronic publication. National University of Ireland, Galway; 2024 [cited 2024 Oct 24]. Available from: https://www.algaebase.org
30. Phillips LE. A re-assessment of the species previously included in *Lenormandiopsis* including the description of *Aneurianna* gen. nov. (Rhodomelaceae, Ceramiales). Cryptogamie Algol. 2006; 27: 213–232.
31. Okamura K. Icones of Japanese algae. Vol. II No. 10. Tokyo: Published by the author; 1912.
32. Lee YP, Yoon SY. Taxonomy of *Chondria* (Rhodophyta) in Korea. Algae 1996; 11: 107–139.
33. Okamura K. Icones of Japanese algae. Vol. VII No. 5. Tokyo: Published by the author; 1934.
34. Nam KW. Algal flora of Korea. Volume 4, Number 3. Rhodophyta: Florideophyceae, Ceramiales: Rhodomelaceae: *Laurencia*, *Chondrophycus*, *Palisada*, *Chondria*. Marine red algae. Incheon: National Institute of Biological Resources; 2011.
35. Yamada Y. Notes on some Japanese algae. IX. Sci Pap Inst Algol Res Fac Sci Hokkaido Imp Univ. 1941; 2: 195–215.
36. Tanaka T, Itono H. Studies on the genus *Neurymenia* (Rhodomelaceae) from southern Japan and vicinities. Mem Fac Fish Kagoshima Univ. 1969; 18: 7–27.
37. Nam KW, Kang PJ. Algal flora of Korea. Volume 4, Number 4. Rhodophyta: Florideophyceae, Ceramiales: Rhodomelaceae: 18 genera including *Herposiphonia*. Marine red algae. Incheon: National Institute of Biological Resources; 2012.
38. Taylor WR. Marine algae of the eastern tropical and subtropical coasts of the Americas. Ann Arbor: The University of Michigan Press; 1960.
39. Littler DS, Littler MM. Caribbean reef plants. An identification guide to the reef plants of the Caribbean, Bahamas, Florida and Gulf of Mexico. Washington: Offshore Graphics; 2000.
40. Yamada Y. Notes on some Japanese algae. X. Sci Pap Inst Algol Res Fac Sci Hokkaido Imp Univ. 1944; 3: 11–25.
41. Okamura K. On the algae from Island Hatidyo. Rec Oceanogr Work Jpn. 1930; 2: 92–110.
42. Okamura K. Icones of Japanese algae. Vol. VI No. 4. Tokyo: Published by the author; 1930.
43. Kajimura M. On a new species of *Griffithsia* (Rhodophyta, Ceramiaceae), *G. okiensis*, from Japan. Mem Fac Sci Shimane Univ. 1982; 16: 77–90.
44. Kim H-S, Yang EC, Boo SM. The occurrence of *Griffithsia okiensis* (Ceramiaceae, Rhodophyta) from Korea on the basis of morphology and molecular data. Algae. 2006; 21: 91–101. https://doi.org/10.4490/algae.2006.21.1.091
45. Okamura K. Icones of Japanese algae. Vol. II No. 8. Tokyo: Published by the author; 1912.
46. Masuda M, Kudo T, Kawaguchi S, Guiry MD. Lectotypification of some marine red algae described by W. H. Harvey from Japan. Phycol Res. 1995; 43: 191–202. https://doi.org/10.1111/j.1440-1835.1995.tb00025.x
47. Holmes EM. Marine algae from Japan. J Linn Soc Lond Bot. 1895; 31: 248–260.
48. Okamura K. Icones of Japanese algae. Vol. II No. 2. Tokyo: Published by the author; 1909.
49. Nam KW, Kang PJ. Algal flora of Korea. Volume 4, Number 11. Rhodophyta: Florideophyceae, Gigartinales: Gigartinaceae: Cystocloniaceae, Kallymeniaceae. Marine red algae. Incheon: National Institute of Biological Resources; 2015.
50. Yang MY, Kim MS. Molecular analyses and reproductive structure to verify the generic relationships of *Hypnea* and *Calliblepharis* (Cystocloniaceae, Gigartinales), with proposal of *C*. *saidana* comb. nov. Algae. 2017; 32: 87–100. https://doi.org/10.4490/algae.2017.32.5.15
51. Paiano MO, Fumo JT, Cabrera FP, Kosaki RK, Spalding HL, Sherwood AR. *Calliblepharis yasutakei* sp. nov. and *Hypnea tsudae* sp. nov. (Cystocloniaceae, Rhodophyta): novel diversity from the Hawaiian Islands. Phytotaxa. 2022; 572: 74–86. https://doi.org/10.11646/phytotaxa.572.1.5
52. Tanaka T. Studies on some marine algae from southern Japan, III. Mem Fac Fish Kagoshima Univ. 1960; 9: 91–105.
53. Okamura K. Icones of Japanese algae. Vol. VII No. 2. Tokyo: Published by the author; 1933.
54. Suzuki M, Terada R, Shibata K, Kawai H. New records of *Chondracanthus saundersii* and *Schottera koreana* (Gigartinales, Rhodophyta) from Japan based on molecular and morphological analyses. Phycol Res. 2021; 69: 81–87. https://doi.org/10.1111/pre.12447
55. Okamura K. Icones of Japanese algae. Vol. VII No. 3. Tokyo: Published by the author, 1934.
56. Yamada Y. Notes on some Japanese algae IV. J Fac Sci Hokkaido Imp Univ Ser 5 Bot. 1932; 2: 267–276.
57. Okamura K. Icones of Japanese algae. Vol. VI No. 8. Tokyo: Published by the author; 1932.
58. Lee HW, Kim MS. Cryptic species diversity of the red algal genus *Callophyllis* (Kallymeniaceae, Gigartinales) from Korea. J Ecol Environ. 2014; 37: 395-410. http://dx.doi.org/10.5141/ecoenv.2014.041
59. Cabrera FP, Huisman JM, Spalding HL, Kosaki RK, Smith CM, Sherwood AR. Cryptic diversity in the genus *Croisettea* (Kallymeniaceae, Rhodophyta) from Hawaiian mesophotic reefs. Phycologia 2022; 61: 572–583. https://doi.org/10.1080/00318884.2022.2096823
60. Lee HW, Kim MS. Undiscovered foliose species of Florideophyceae (Rhodophyta) from subtidal habitat along the Korean coastline. J Asia-Pac Biodivers. 2023; 16: 215-226. https://doi.org/10.1016/j.japb.2023.03.012
61. Yamada Y. Notes on some Japanese algae, V. J Fac Sci Hokkaido Imp Univ Ser 5 Bot. 1933; 2: 277–285.
62. Okamura K. Icones of Japanese algae. Vol. VI No. 2. Tokyo: Published by the author; 1929.
63. Lin SM. Marine benthic macroalgal flora of Taiwan Part I order Gracilariales (Rhodophyta). Keelung: National Taiwan Ocean University Press; 2009.
64. Yamamoto H. Review on *Gracilaria sublittoralis* Yamada et Segawa (nom. nud.), Gracilariaceae, Rhodophyta. Jpn J Phycol. 1994; 42: 421–424.
65. Tanaka T. Studies on some marine algae from southern Japan, V. Mem Fac Fish Kagoshima Univ. 1963; 12: 75–91.
66. Kim H-S, Hwang I-K. Algal flora of Korea. Volume 4, Number 10. Rhodophyta: Florideophyceae: Gelidiales, Gracilariales, Plocamiales. Marine red algae. Incheon: National Institute of Biological Resources; 2015.
67. Chang CF, Xia BM. Studies on Chinese species of *Gracilaria*. Stud Mar Sin 1976; 11: 91–166.
68. Terada R, Shimada S. Taxonomic note on *Gracilaria articulata* Chang et Xia (Gracilariales, Rhodophyta) from Okinawa, Japan. Cryptogamie Algol. 2005; 26: 77–89.
69. Lin S-M, Liu L-C, Payri C. Charcterization of *Gracialria vieillardii* (Gracilariaceae, Rhodophyta) and molecular phylogeny of foliose species from the western Pacific Ocean, including a description of *G. taiwanensis* sp. nov.. Phycologia. 2012; 51: 421–431. https://doi.org/10.2216/11-90.1
70. Suzuki M, Terada R. A new flattened species of *Gracilariopsis* (Gracilariales, Rhodophyta) from Japan. Phycologia. 2021; 60: 158–163. https://doi.org/10.1080/00318884.2021.1880755
71. Lin S-M, De Clerck O, Leliaert F, Chuang Y-C. Systematics and biogeography of the red algal genus *Yonagunia* (Halymeniaceae, Rhodophyta) from the Indo-Pacific including the description of two new species from Taiwan. J Phycol. 2020; 56: 1542–1556. https://doi.org/10.1111/jpy.13055
72. Lee HW, Yang MY, Kim MY. Verifying a new distribution of the genus *Amalthea* (Halymeniales, Rhodophyta) with description of *A*. *rubida* sp. nov. from Korea. Algae. 2016; 31: 341–349. https://doi.org/10.4490/algae.2016.31.12.8
73. D'Archino R, Nelson WA, Zuccarello GC. *Amalthea* and *Galene*, two new genera of Halymeniaceae (Rhodophyta) from New Zealand. Bot Mar. 2014; 57: 185-201. https://doi.org/10.1515/bot-2014-0008
74. Tanaka T. Studies on some marine algae from southern Japan, IV. Mem Fac Fish Kagoshima Univ. 1963; 12: 64–71.
75. Yang MY, Kim MS. *Cryptonemia asiatica* sp. nov. (Halymeniaceae, Rhodophyta), a new marine macroalgal species from Korea and Japan. J Ecol Environ. 2014; 37: 383–393. http://dx.doi.org/10.5141/ecoenv.2014.040
76. Schneider CW, Popolizio TR, Kraft LGK, Saunders GW. New species of *Galene* and *Howella gen. nov.* (Halymeniaceae, Rhodophyta) from the mesophotic zone off Bermuda. Phycologia. 2019; 58: 690-697. https://doi.org/10.1080/00318884.2019.1661158
77. Okamura K. Icones of Japanese algae. Vol. II No. 3. Tokyo: Published by the author; 1909.
78. Nam KW, Kang PJ. Algal flora of Korea. Volume 4, Number 9. Rhodophyta: Florideophyceae: Halymeniales: Halymeniaceae, Tsengiaceae. Marine red algae. Incheon: National Institute of Biological Resources; 2013.
79. Dawson EY. Marine red algae of Pacific Mexico. Part 4. Gigartinales. Pac Naturalist. 1961; 2: 191–343
80. Nozawa Y. Systematic anatomy of Squamariaceae in the southern islands of Japan (4). Jpn J Phycol. 1972; 20: 41–47.
81. Yang MY, Kim MS. Cryptic diversity and phylogeographic patterns of Plocamium telfairiae and P. cartilagineum (Plocamiales, Rhodophyta) in the Northwest Pacific. Algae. 2023; 38: 159–172. https://doi.org/10.4490/algae.2023.38.8.4
82. Okamura K. Contribution to knowledge of the marine algae of Japan, II. Bot Mag Tokyo. 1896: 10: 21–28, 33–40.
83. Okamura K. Icones of Japanese algae. Vol. IV No. 6. Tokyo: Published by the author; 1921.
84. Rodríguez-Prieto C, De Clerck O, Kitayama T, Lin S-M. Systematic revision of the widespread species Sarcodia ceylanica (Sarcodiaceae, Rhodophyta) in the Indo-Pacific Oceans, including S. suiae sp. nov. Phycologia. 2017; 56: 63–76. https://doi.org/10.2216/16-31
85. Yendo K. Three new marine algae from Japan. Bot Mag Tokyo. 1903; 17: 99–104.
86. Koh YH, Cho GY, Kim MS. Species delimitation of the genus Champia (Rhodymeniales, Rhodophyta) from Korea using DNA barcoding. J Ecol Environ. 2013; 36: 449–463. https://doi.org/10.5141/ecoenv.2013.449
87. Børgesen F. Some Indian Rhodophyceae especially from the shores of the Presidency of Bombay. III. Bull Misc Inform Kew. 1933; 1933: 113–142.
88. Masuda M, Kogame K, Kawaguchi S, Phang SM. Taxonomic notes on marine algae from Malaysia. V. Species of Rhodymeniales (Rhodophyceae). Bot Mar. 2001; 44: 81–88. https://doi.org/10.1515/BOT.2001.011
89. Womersley HBS. The Marine Benthic Flora of Southern Australia Part IIIB Gracilariales, Rhodymeniales, Corallinales and Bonnemaisoniales. Canberra: Australian Biological Resources Study & the State Herbarium of South Australia; 1996.
90. Okamura K. Icones of Japanese algae. Vol. IV No. 7. Tokyo: Published by the author; 1921.
91. Weber-van Bosse A. Liste des algues du Siboga, IV. Rhodophyceae. Troiseme partie. Gigartinales et Rhodymeniales et tableau de la distribution des Chlorophycees, Phaeophycees et Rhodophycees de l'Archipel Malaisien. Siboga Exped Monogr. 1928; 59d: 393–533.
92. Kimori M, Amano Y, Terada R. Reconfirmation of *Chamaebotrys lomentariae* (Tanaka et Nozawa) Huisman from offshore of Tanegashima, southern Japan. Nat Kagoshima. 2011; 37: 165–167.
93. Huisman JM. The red algal genus *Coelarthrum* Børgesen (Rhodymeniaceae, Rhodymeniales) in Australian seas, including the description of *Chamaebotrys* gen. nov. Phycologia. 1996; 35: 95–112. https://doi.org/10.2216/i0031-8884-35-2-95.1
94. Okamura K. Icones of Japanese algae. Vol. VII No. 1. Tokyo: Published by the author; 1933.
95. Filloramo GV, Saunders GW. Application of multigene phylogenetics and site-stripping to resolve intraordinal relationships in the Rhodymeniales (Rhodophyta). J Phycol. 2016; 52: 339–355. https://doi.org/10.1111/jpy.12418
96. Saunders GW, Lane CE, Schneider CW, Kraft GT. Unraveling the *Asteromenia peltata* species complex with clarification of the genera *Halichrysis* and *Drouetia* (Rhodymeniaceae, Rhodophyta). Can J Bot. 2006; 84: 1581–1607. https://doi.org/10.1111/jpy.12418
97. Suzuki M, Terada R. Morpho-anatomical and molecular reassessments of *Rhodymenia prostrata* (Rhodymeniaceae, Rhodophyta) from Japan support the recognition of *Halopeltis tanakae* nom. nov. Phycologia. 2021; 60: 582–588. https://doi.org/10.1080/00318884.2021.1959743
98. Okamura K. Icones of Japanese algae. Vol. VII No. 4. Tokyo: Published by the author; 1934.
99. Schneider CW, Freshwater DW., Saunders, GW. First report of *Halopeltis* (Rhodophyta, Rhodymeniaceae) from the non-tropical Northern Hemisphere: *H*. *adnata* (Okamura) comb. nov. from Korea, and *H*. *pellucida* sp. nov. and *H*. *willisii* sp. nov. from the North Atlantic. Algae. 2012; 27: 95–108.
100. Okamura K. Icones of Japanese algae. Vol. V No. 10. Tokyo: Published by the author; 1928.
